# RNA Sequencing of Intestinal Enterocytes Pre- and Post-Roux-en-Y Gastric Bypass Reveals Alteration in Gene Expression Related to Enterocyte Differentiation, Restitution, and Obesity with Regulation by Schlafen 12

**DOI:** 10.3390/cells11203283

**Published:** 2022-10-18

**Authors:** Emilie E. Vomhof-DeKrey, Sonalika Singhal, Sandeep K. Singhal, Allie D. Stover, Odele Rajpathy, Elizabeth Preszler, Luis Garcia, Marc D. Basson

**Affiliations:** 1 Department of Surgery, School of Medicine and the Health Sciences, University of North Dakota, Grand Forks, ND 58202, USA; 2 Department of Biomedical Sciences, School of Medicine and the Health Sciences, University of North Dakota, Grand Forks, ND 58202, USA; 3 Department of Pathology, School of Medicine and the Health Sciences, University of North Dakota, Grand Forks, ND 58202, USA; 4 Sanford Health Clinic, Sioux Falls, ND 57117, USA

**Keywords:** Schlafen 12 (SLFN12), laser microdissection, intestinal epithelial cells, enterocytes, obesity, weight loss

## Abstract

Background: The intestinal lining renews itself in a programmed fashion that can be affected by adaptation to surgical procedures such as gastric bypass. Methods: To assess adaptive mechanisms in the human intestine after Roux-en-Y gastric bypass (RYGB), we biopsied proximal jejunum at the anastomotic site during surgery to establish a baseline and endoscopically re-biopsied the same area 6–9 months after bypass for comparison. Laser microdissection was performed on pre- and post-RYGB biopsies to isolate enterocytes for RNA sequencing. Results: RNA sequencing suggested significant decreases in gene expression associated with G2/M DNA damage checkpoint regulation of the cell cycle pathway, and significant increases in gene expression associated with the CDP-diacylglycerol biosynthesis pathway TCA cycle II pathway, and pyrimidine ribonucleotide salvage pathway after RYGB. Since Schlafen 12 (SLFN12) is reported to influence enterocytic differentiation, we stained mucosa for SLFN12 and observed increased SLFN12 immunoreactivity. We investigated SLFN12 overexpression in HIEC-6 and FHs 74 Int intestinal epithelial cells and observed similar increased expression of the following genes that were also increased after RYGB: HES2, CARD9, SLC19A2, FBXW7, STXBP4, SPARCL1, and UTS. Conclusions: Our data suggest that RYGB promotes SLFN12 protein expression, cellular mechanism and replication pathways, and genes associated with differentiation and restitution (HES2, CARD9, SLC19A2), as well as obesity-related genes (FBXW7, STXBP4, SPARCL1, UTS).

## 1. Introduction

Patients undergoing Roux-en-Y gastric bypass (RYGB) surgery lose weight and exhibit improved glycemic and lipidemic control and other obesity-related co-morbidities [1,2,3]. Interestingly, such metabolic improvements are not entirely dependent on weight loss, as these improvements are often clinically observed before significant weight loss is even achieved [4]. It is not fully understood how bariatric surgery induces these metabolic improvements, especially since obesity-related metabolic disturbances are generated as a consequence of an interaction between lifestyle, aging, environment, and genomic and epigenetic factors [1]. Additionally, the RYGB method alters gastrointestinal continuity [5]. Within the jejunal segment that is positioned at the proximal end of the Roux-limb, the intraluminal milieu is substantially altered by its more proximal location in the chain of oral intake. This results in an augmentation in microbiota and mechanical load as well as the chemical effects of undigested food [6]. However, little is known about how enterocyte gene expression is affected by RYGB.

We previously studied the role of SLFN12 (and its mouse ortholog, Slfn3) in the regulation of enterocyte differentiation [7,8,9,10,11,12,13]. SLFN12 is one of a family of proteins that also regulate hematopoiesis, the immune response, and bone biology [14,15,16,17]. SLFN proteins are classified into three groups based on size. Group I, long SLFN proteins, have a helicase domain with a nuclear targeting sequence that allows for nuclear DNA binding and then modulation of transcription. However, intermediate Group II SLFN proteins such as SLFN12 and the short Group III SLFN proteins lack this nuclear targeting sequence [16]. Instead, SLFN12 influences enterocyte differentiation through a pathway involving SLFN12, serpinB12, and the deubiquitylases UCHL5 and USP14 that in turn alter protein levels of the transcription factor CDX2 [8,9]. In this study, we collected operative RYGB proximal jejunum tissue at the anastomotic site and endoscopically biopsied the same area 6–9 months after RYGB for comparison. After sectioning and staining, laser capture dissection isolated enterocytes along the villi and RNASeq were performed on RNA isolated from the laser capture dissected enterocytes. We stained for SLFN12 to evaluate the effects of RYGB on enterocytic SLFN12 and then investigated whether SLFN12 might influence some of the RNASeq genes that were affected by RYGB.

## 2. Materials and Methods

### 2.1. Human Samples

Human samples and non-identifying patient information were obtained under a protocol approved under IRB-201606-406 from the University of North Dakota in Grand Forks, ND, USA and by the IRB of the Sanford Heath System in Fargo, ND, USA. Patients who were already scheduled for a Roux-en-Y gastric bypass surgery for clinical indications at Sanford Health were recruited for participation in this research study. The study population thus consisted of 11 consecutive patients scheduled for RYGB who had consented to participation in the research. During the scheduled RYGB surgery, the most proximal 2 cm portion of the Roux limb (proximal jejunum) was collected and divided into 2 mL tubes for either direct freezing in liquid nitrogen, or storage in DNA/RNA shield solution (Zymo Research, Irvine, CA, USA) or 10% formalin (Sigma-Aldrich, St. Louis, MO, USA). Six to nine months after RYGB, each patient was scheduled for an upper endoscopy. Four to six endoscopic mucosal biopsies were obtained from the jejunum. Each biopsy consisted of a piece of mucosa that was approximately 0.4 cm × 0.2 cm × 0.1 cm. Biopsies were collected into 2 mL tubes and preserved in a similar way to the RYGB samples above. After all the samples had been collected and deidentified with a subject ID number, they were transported to the research laboratory at the University of North Dakota together with corresponding deidentified health information for each research subject including age, sex, race, medical conditions, current medications, weight, and BMI prior to surgery and at the time of endoscopy.

### 2.2. Tissue Sectioning, Staining, and Laser Capture Microscopy

Frozen tissue was embedded in an optimal cutting temperature compound and 8 µm sections were frozen sectioned on a microtome-cryostat. Sections were mounted onto PET membrane steel frame slides (1.4 µm, RNase-free, Leica LMD slides; NCI, Inc. Brooklyn Park, MN, USA) and sectioned slides were stored at −80 °C. Applied 50 µL of RNase Inhibitor (ProtectRNA 500x R7397, Sigma-Aldrich, St. Louis, MO, USA) was added to all staining jars (per 25 mL of the following ethanol and isopropanol jars, not in a xylene jar). Frozen slides were immediately submerged into 95% ethanol for 30 s. with frequent dipping up and down. Hematoxylin stain was directly applied to the tissue sections on the slides for 60 s. These were then de-stained in 100% isopropanol, via 10 dips for 15 s each. Eosin stain was applied directly to tissue sections on the slide for 2 s and they were then de-stained in 100% isopropanol for 10 dips for 30 s each. The slides were subsequently de-stained in 75% ethanol for five dips for 15 s, then 95% ethanol for 10 dips, then 100% ethanol for 10 dips for 10 s. Finally, the slides were submerged in anhydrous 100% ethanol for 30 s. Then the slides were repeatedly dipped in xylene for 15 s with a final submersion for 10 min. Each slide was air-dried for 2 min. Laser capture microscopy immediately followed in which two sections of about 10–30 intestinal epithelial cells along with the villi were cut and collected into the collection cap (Leica LMD 6, Deer Park, IL, USA). Sectioned cells were lysed with 5 µL of lysis buffer using the Tecan LCMSeq kit and tubes were stored at −80 °C. The RNA library was prepared using the Tecan Ovation SoLo RNA-Seq library preparation kit as per the manufacturer’s instructions. Libraries were quantified using the Quant-iT PicoGreen dsDNA assay kit and read using a BioTek FLx800 fluorescence microplate reader. Libraries were pooled at equal molarity and the sample pool was quantified using Biorad ddPCR. Libraries were sequenced using an Illumina HiSeq X Ten at Psomagen (Rockville, MD, USA).

### 2.3. Immunohistochemical Staining for Slfn12 Protein

A Leica Bondmax Stainer in modified protocol F was used to stain for Slfn12 protein in the gastric bypass and EGD tissue sections. Paraffin sections were dewaxed and antigen retrieval was performed with Leica Bond Epitope Retrieval Solution 1 for 20 min. For immunostaining, slides were sequentially washed with bond wash buffer (BWB), incubated with SLFN12 antibody (Rabbit polyclonal, LS-B4757, 1:200 dilution; LSBiosystems, Seattle, WA, USA) for 30 min, bonded after the primary antibody for 20 min, washed with BWB, bonded with polymer for 20 min, washed with BWB, bonded with peroxidase block for 10 min, washed with BWB, bonded with DAB for 10 min, washed with deionized water, bonded with hematoxylin for 5 min, and washed with BWB. The slide was dehydrated, and a coverslip was applied. Slides were then deidentified as to the peri-operative or post-operative nature of the specimens. Two blinded observers were asked to score the intensity of epithelial SLFN12 staining on a 0 to 3 scale in each image based upon a predetermined scale. These scores were then aggregated and subjected to statistical analysis by a Student t-test in a GraphPad Prism 8.

### 2.4. Cell Culture and RNA Expression Analysis

Human HIEC-6 cells (ATCC, Manassas, VA, USA) were cultured in a base medium of OptiMEM 1 Reduced Serum (Gibco, Thermofisher, Waltham, MS, USA), 20 mM HEPES (Thermofisher, Waltham, MS, USA), 10 mM GlutaMAX (Thermofisher, Waltham, MS, USA), 10 ng/mL epidermal growth factor (EGF, Biolegend, San Diego, CA, USA) and 4% fetal bovine serum (FBS, Genesee Scientific, El Cajon, CA, USA). FHS 74 Int cells (ATCC, Manassas, VA, USA) were cultured in a base medium of Hybri-Care ATCC 46-X, 30 ng/mL EGF, and 10% FBS. Both cell lines were incubated at 37 °C with 5% CO_2_. Cells in 6-well plates with 300,000 cells/well were incubated with 4000 vp/cell Slfn12 adenovirus (Adv-Slfn12) or Adv-CMV for 2 h. Media was replaced and the cells harvested 72 h later with 600 µL of RLT buffer. RNA was isolated using the RNeasy Mini Kit and Qiacube as per the manufacturer’s protocols (Qiagen, Valencia, CA, USA). A SmartScribe RT kit was used for cDNA synthesis (Takara Clontech, Mountain View, CA, USA). The BioRad CFX96 Touch Real-Time PCR Detection System and the PrimeTime Gene Expression Master Mix (IDT, Coralville, IA, USA) were utilized for qPCR analysis. Expression levels were ascertained from the threshold cycle (Ct) values using the 2^−∆∆Ct^ method, where RPLP0 was used as a housekeeping gene. The following primer/probe sets were used from BioRad (Hercules, CA, USA) and are proprietary: RPLP0 (Assay ID: qHsaCEP0041375, HEX), SPARCL1 (Assay ID: qHsaCEP0057695, TEX615), SLC19A2 (Assay ID: qHsaCIP0031109, Cy5.5), MBOAT2 (Assay ID: qHsaCEP0052422, Cy5.5), and FBXW7 (Assay ID: qHsaCIP0031037). Primer/probe sets from IDT were as follows: CARD9 forward 5′-GA CTG GAA GAT GGC TCA C-3′, reverse 5′-AGC TGC AAA GGG CTG TT-3′, probe 5′-/56-FAM/TTT GTC TGA/ZEN/GAG CTG GGT GTC CTC/3IABkFQ/-3′; STXBP4 forward 5′-CCT AAA TCC CTC TGT TCG CTT-3′, reverse 5′-AGT CTG CTT GTA CTT GCT GTC-3′, probe 5′-/56-FAM/TTG GTG TTG/ZEN/TTC CTT TGT GGG CTG/3IABkFQ/-3′; UTS2 forward 5′-CCT TCT ACA GAT ACT GCC AGA-3′, reverse 5′-AAC TTT CTC AAA TTT CCT CTT GGG-3′, probe 5′-/56-FAM/ATA TCC CCT/ZEN/CTT TCT GCA CCC AGC/3IABkFQ/-3′; HES2 forward 5′-CTG CTC GAA GCT AGA GAA GG-3′, reverse 5′-GTA GCT GTC GCA AGG CA-3′, probe 5′-/5Cy5/CGC ACG GTC ATT TCC AGG ACG T/3IAbRQSp/-3′; TRPC5 forward 5′-TCG ATG AGC CTA ACA ACT GC-3′, reverse 5-CCA AAT ACA GAC CAG AAG AGT GA-3′, probe 5′-/5Cy5/TCA AAG AGC GTG GAG AAG GCA TTG T/3IAbRQSp/-3′. qPCR cycle conditions were 1 cycle of 2 min at 95 °C, 50 cycles of 10 s at 95 °C and 45 s at the annealing temperature of 55 °C.

### 2.5. Processing of Sequence Data

RNA-Seq raw reads were stored in FASTQ format files containing the sequence data for this cohort study (11 patients). The quality of each raw FASTQ file was explored using fastQC [18]. As per the quality control report on the raw data, files were subjected to pre-processing steps including trimming, quality filtering, per-read quality pruning using a fastp [19] tool. After the data had been preprocessed, the FASTQ file reads were then aligned to a reference genome (Homo sapiens GRCh38, available on ENSEMBL [20]) using a STAR aligner v.2.5.2b to generate the read counts. The normalized distribution of the counts was checked to identify the outliers.

### 2.6. Statistical Analysis

Two sub-selections of the samples were made to classify pre-surgery and post-surgery samples and to compare the effects of gastric bypass on the gene profiles of patients. Principal component analysis (PCA) was performed on the RNA-Seq to describe the relationship between the samples by visualizing the samples and groups in two-dimensional space. The statistically significant genes that were differentially expressed in the sub-selections of the samples were identified using a two-tailed Student’s t-test. The significance level of the *p*-value < 0.05 was employed as a standard to filter genes. Further pathway and function enrichment analyses of the significant genes were performed using the Ingenuity Pathway Analysis (IPA, Ingenuity Systems; Qiagen, Valencia, CA, USA; www.qiagen.com/ingenuity, accessed on 4 June 2021) to identify the biological functions, canonical pathways, and regulatory networks of the functional mRNA target interactions. Comma delimited text files containing gene IDs, expression data (fold change), and *p*-values were uploaded into the IPA for core analysis. The top functions, pathways and gene-signaling networks were calculated using IPA-generated negative logarithm *p*-values i.e., −log10(*p*-value) and associated Z- and network scores. The investigation into the outcome of the surgery was observed by looking at the change in BMI (body mass index) of the patients. To differentiate between the gene expression among patients who were successful in weight loss and those who did not lose substantial weight, the change in BMI as a linear predictor was taken for post-surgery samples.

## 3. Results

### 3.1. Comparison between Pre- and Post-Surgery Gene Expression

**Figure 1:**
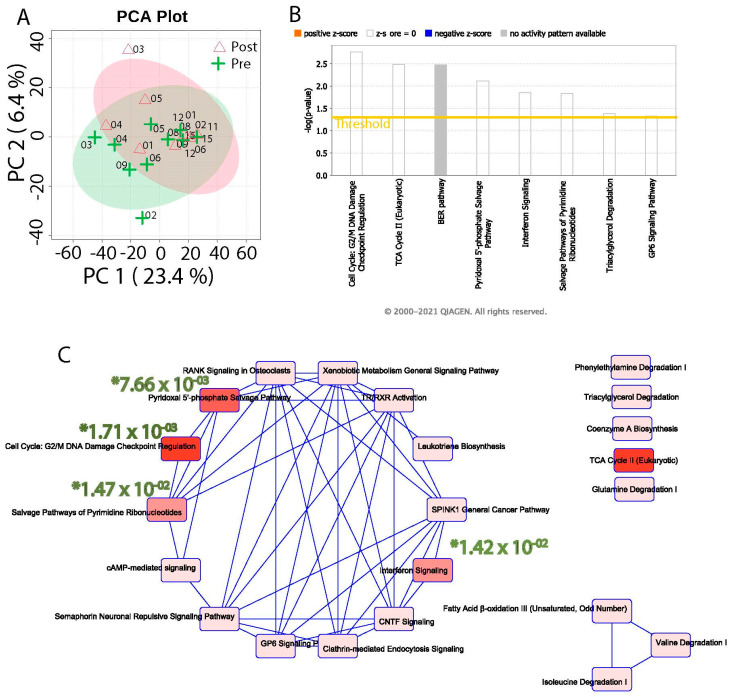
(**A**) PCA plot for pre- and post-surgery conditions. (**B**) IPA pathway enrichment showing top eight pathways for 1116 significant genes. The yellow line represents the threshold of *p*-value ≤ 0.05. (**C**) Their network of interaction of topmost pathways is shown. (**C**) Overlapping pathways: The figure shows overlapping pathways associated with differential genes. The nodes represent pathways and edges are labeled with the number of common genes connecting each node. The four most statistically significant pathways are marked with a * followed by the significant *p*-value.

The distribution of data for the gene expression levels before and after surgery was observed through the application of principal component analysis (PCA). Figure 1A shows the variation between pre- and post-RYGB samples as a group and individuals using the first two principal components. The overall gene expression of pre- and post- surgery samples did not form distinct clusters, suggesting that not all patients conformed to a similar gene expression pattern. The total variation observed between the groups was 23.4% to 6.4% for principal component-1 (PC1) and principal component-2 (PC2), respectively. The paired samples ranged in variations from very low (such as GBP11-EGD11, GBP15-EGD15) to very high (such as GBP02-EGD02, GBP03--EGD03, seen as outliers) between pre- and post-surgery on the PC1 and PC2 scales.

Differential expression analysis between Post-RYGB and Pre-RYGB samples identified 1116 (916 upregulated and 200 downregulated) genes that changed significantly to meet the threshold *p* < 0.05 (Table S1). To gain further insights into the functional association of these genes, all significant genes were investigated by IPA core analysis. This analysis identified eight pathways (Figure 1B) that attained the desired level of significance (*p* < 0.05). The interaction between these pathways suggests two clusters in which 14 pathways are functionally interlinked. The human pathways significantly enriched were Cell Cycle: G2/M DNA damage Checkpoint Regulation (7.66 × 10^−3^), pyridoxal 5′ phosphate salvage (7.66 × 10^−3^), salvage pathways of pyrimidine ribonucleotides (1.47 × 10^−2^), and interferon signaling (1.42 × 10^−2^) based on their significance value (*p*-value ≤ 0.05) (Figure 1C).

### 3.2. Changes in the Gene Profiles for Prominent Responders of Gastric Bypass

**Figure 2:**
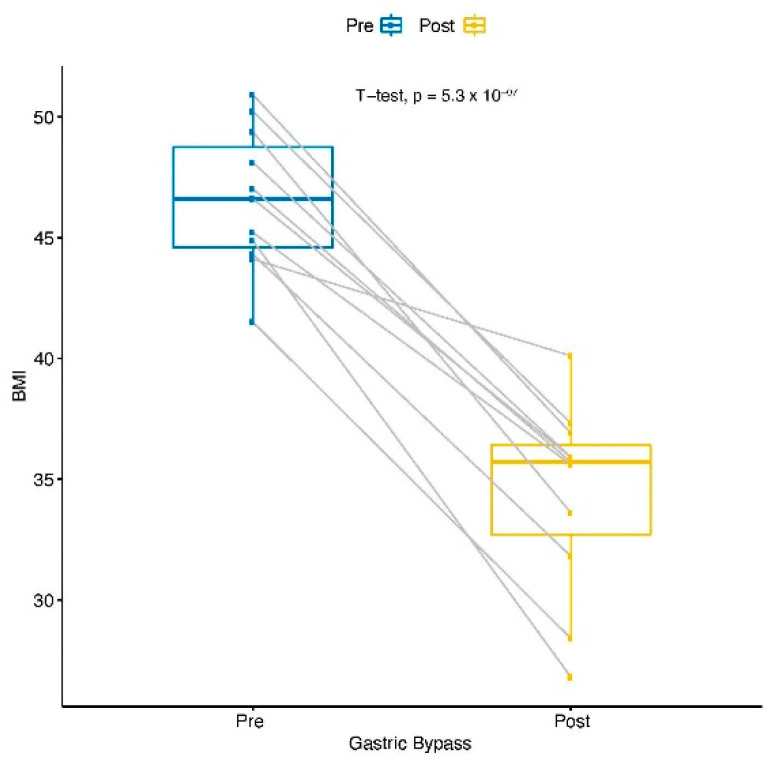
Comparison of weight loss in pre- and post-surgery conditions. For a better comparison, BMI was used to monitor changes in weight loss.

**Figure 3:**
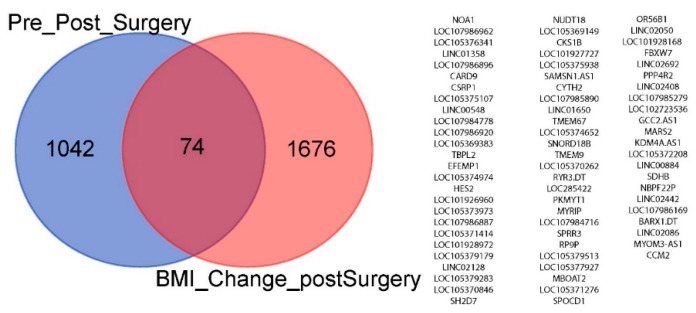
Comparison of genes changing in the post-RYGB samples with respect to the change in BMI (Table S2) vs. genes identified between the pre- vs. post-RYGB genes (Table S1).

**Figure 4:**
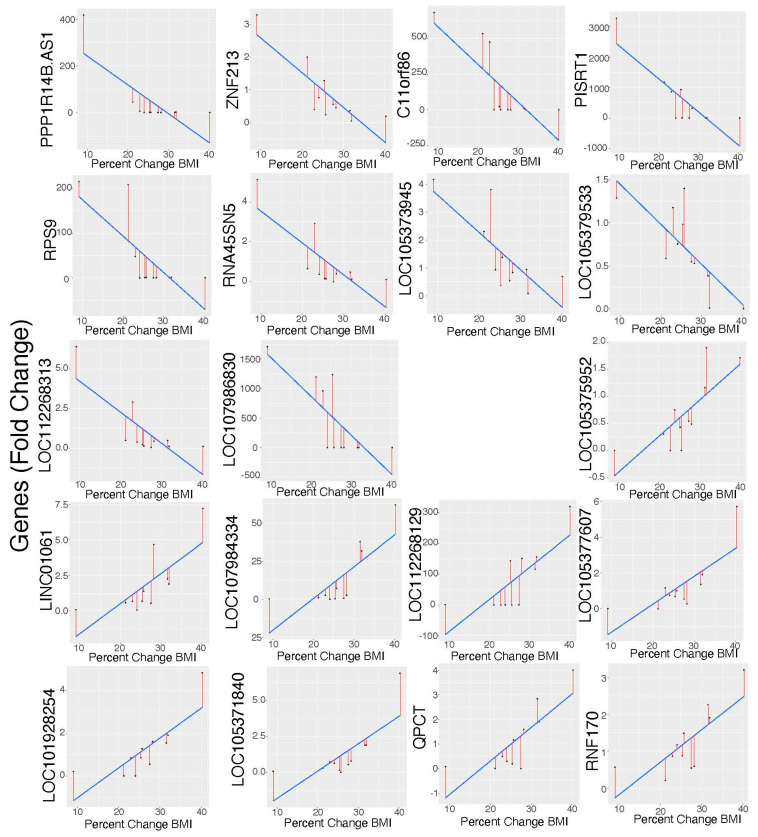
Linear relationship between gene expression fold change and percentage change in BMI. Each dot represents one patient. The blue line shows the linear relationship, and the red line shows the deviation from the blue line for each patient.

All patients lost weight after surgery, as evidenced by a substantially decreased body mass index (BMI (Figure 2). We compared the gene expression level with the BMI to find the genes most significantly associated with weight loss. Three different approaches were applied to find the most significant genes associated with the pre- and post-RYGB and BMI changes together. First, we identified the genes changing in the samples after RYGB with respect to the change in BMI (pre- vs. post-RYGB) using a regression analysis. A total of 1750 genes changed significantly with *p* < 0.05 (Table S2). We then compared these 1750 genes (Table S2) with the 1116 genes identified between the pre- and the post-RYGB in Table S1. There were 74 genes in common between the two analyses (Figure 3). Second, to test whether there was any significant statistical relationship between the BMI and pre/post-RYGB together with the changes in gene expression, we introduced an interaction term to the regression model. We found 1200 genes that had changed significantly with the combined effect of RYGB and BMI (Table S3). Out of these 1200 genes, 540 genes were upregulated and 660 were downregulated. Third, in another approach, we identified the genes whose expression changed significantly (in terms of fold change) with respect to changes in percentages of BMI when measured pre- and post-the RYGB condition. A total of 867 genes were highly correlated (Spearman correlation coefficient > 0.70) with 789 upregulated and 78 downregulated (Table S4). Figure 4 shows the changes in 19 genes from this list which significantly changed their expression with respect to the change in BMI (Table S5).

### 3.3. SLFN12 Protein Expression Is Increased after Gastric Bypass

**Figure 5:**
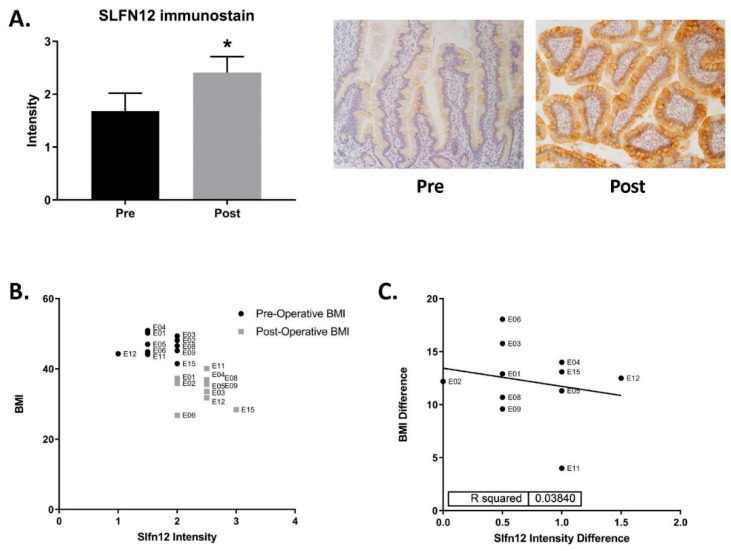
SLFN12 protein expression increases after gastric bypass. (**A**) Immunohistochemical staining in the intestinal mucosa for SLFN12 protein in pre- and post-RYGB samples and scoring by blinded observers. (Two observers each achieved similar results, * *p* ≤ 0.05.) (**B**) Comparison of patient BMI and SLFN12 protein intensity pre- and post-gastric bypass. (**C**) Correlation graph of the BMI difference vs. the SLFN12 protein intensity difference between pre- and post- gastric bypass samples. (*n* = 11).

Human enterocytic differentiation is regulated by SLFN12 via a pathway involving SerpinB12, the deubiquitylases UCHL5 and USP14, and the transcription factor, Cdx2 [9]. Indeed, SLFN12 protein immuno-reactivity was clearly greater after surgery as compared to the preoperative baseline (typical images in Figure 5a, right panel) and statistical analysis of scoring by blinded observers confirmed this difference (Figure 5a, left panel). The SLFN12 protein immunoreactivity average intensity values were subsequently compared with the pre- and post-operative BMIs (Figure 5B). Furthermore, the change in SLFN12 protein intensity after surgery in individual patients had a trending correlation that could indicate an increase in SLFN12 expression, with a decrease in BMI after surgery (Figure 5C).

### 3.4. Similar RNA Expression Change in Gastric Bypass-Specific Genes Compared to HIEC-6 or FHs 74 Int Cells Overexpressing SLFN12

**Figure 6:**
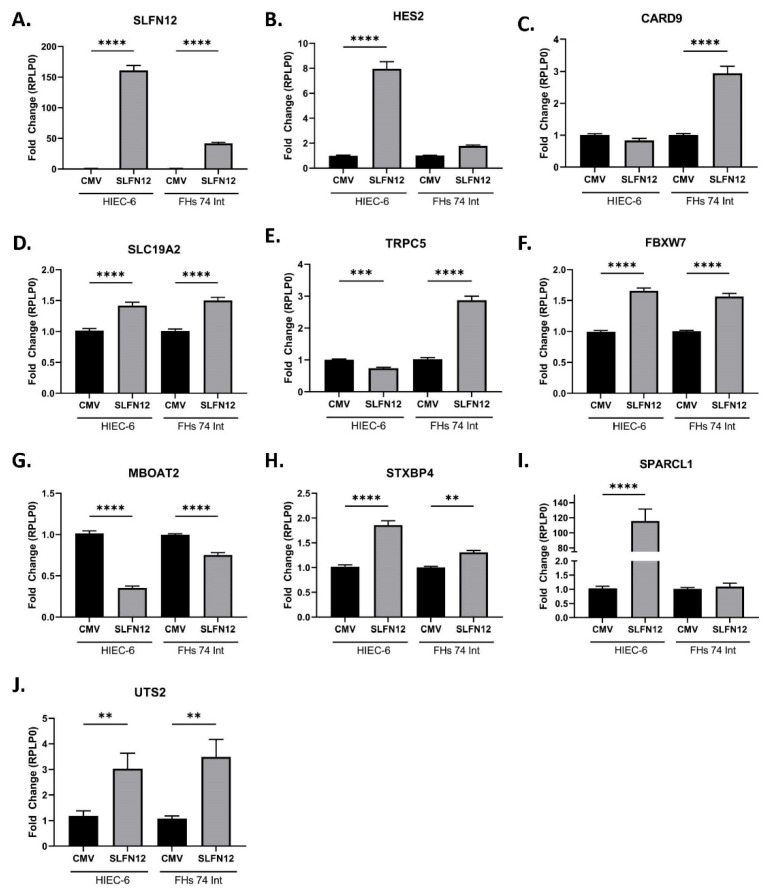
Overexpression of SLFN12 in human intestinal epithelial cell lines influences the expression of differentiation, restitution, and obesity-related genes. RNA expression of (**A**) SLFN12 was measured from either HIEC-6 or FHs 74 Int cells that were treated with either CMV adenovirus control or AdvSLFN12. The following genes are involved in differentiation and restitution of intestinal mucosa: (**B**) HES2, (**C**) CARD9, (**D**) SLC19A2, (**E**) TRPC5, while the following genes are related to obesity: (**F**) FBXW7, (**G**) MBOAT2, (**H**) STXBP4, (**I**) SPARCL1, and (**J**) UTS2. (*n* = 15–24, **** *p* < 0.0001, *** *p* < 0.001, ** *p* < 0.01).

To investigate whether this higher SLFN12 expression after RYGB could contribute to some of the post-operative gene changes that we observed, we overexpressed SLFN12 with an adenovirus (AdvSLFN12) in two human intestinal cell lines, HIEC-6 and FHs 74 Int (Figure 6A). The following genes were selected for study from among those genes identified by the RNAseq analysis (Table S1 and Figure 3) because the literature suggests that they are involved in the differentiation and restitution of intestinal mucosa: HES2, CARD9, TRPC5, and SLC19A2 [21,22,23,24,25]. We also analyzed the expression of FBXW7, MBOAT2, STXBP4, SPARCL1, and UTS after SLFN12 overexpression because these are obesity-related genes [26,27,28,29,30,31,32]. HES2 and SPARCL1 were increased in RNA expression in the HIEC-6 + AdvSLFN12 cells compared to the CMV control. However, the FHs 74 Int + AdvSLFN12 treated cells only had a modest but not significant increase (Figure 6B,I). CARD9 was only increased in the FHs 74 Int + AdvSLFN12 treated cells compared to the CMV control (Figure 6C). SLC19A2, FBXW7, STXBP4, and UTS2 all increased in RNA expression in both the HIEC-6 and FHs 74 Int + AdvSLFN12 treated cells in comparison to controls (Figure 6D,F,H,J), while MBOAT2 RNA expression decreased (Figure 6G). Interestingly, TRPC5 decreased in the HIEC-6 + AdvSLFN12 treated cells, while in the FHs 74 Int + AdvSLFN12 treated cells the TRPC5 RNA expression increased in comparison to the CMV control (Figure 6E). The changes observed in the genes above were compared to the RNA Seq expression data in Table 1. Most of the genes that we studied had similar increases or decreases in expression between the RNA Seq data and the AdvSLFN12 overexpressed intestinal cell lines, except for HES2, TRPC5, and SPARCL1 in the FHs 74 Int AdvSLFN12 treated cells, CARD9 in the HIEC-6 AdvSLFN12 treated cells, and MBOAT2, which decreased in the intestinal AdvSLFN12-treated cells compared to the RNA Seq data indicating an increase in MBOAT2 expression (Table 1).

## 4. Discussion

Instead of collecting RNA from whole intestinal mucosal tissue, we performed laser capture microscopy to collect intestinal epithelial cells only from the villi of the proximal jejunum biopsies from the Roux-en-Y gastric bypass surgery and then the EGD biopsies 6–9 months post-operatively. Patients served as their own controls for a paired analysis when examining the genes in laser-dissected enterocytes that had changed after RYGB. RNA sequencing identified 1116 statistically significant genes and pathways that changed expression in the enterocytes after RYGB (Table S1, Figure 1B). The most statistically significant canonical pathways that were affected were the cell cycle G2/M DNA damage checkpoint regulation, the CDP-diacylglycerol biosynthesis, the phosphatidylglycerol biosynthesis, the TCA cycle, and the pyrimidine ribonucleotide interconversion (derived from Figure 1 and summarized in Table 2). Jorsal et al. performed mRNA sequencing on 20 patients’ intestinal mucosal biopsies that had been collected 3 months prior to RYGB surgery and 3 months after RYGB surgery [3]. They identified 2437 differentially expressed genes after RYGB and then focused on the specific expression changes of 16 prohormones of enteroendocrine or neuroendocrine peptides and 39 G-protein coupled receptor genes [3]. They identified 16 robustly upregulated or downregulated prohormone genes. Notably, we did not observe a change in gene expression for these same genes in our study [3]. Additionally, Jorsal stated that 39 G-protein coupled receptors GPCRs had differential expression (up- or down-regulation was not noted) after RYGB [3]. Two GPCRs that were differentially expressed in both their study and ours were ADORA1 and S1PR3 [3]. We also observed expression changes in the following related but different GPCRs: GPR158, ADGRA3, ADGRG3, and ADRM1. Another study by Ben-Zvi et al. analyzed jejunal mucosa biopsies, from at the time of RYGB surgery and 1 month after RYGB [4]. They observed expression changes related to glucose, fatty acid, and cholesterol metabolism [4]. Although we did not find the same genes to change expression in our study similar to those reported by Ben-Zyi and colleagues, Ben-Zyi did observe an increase in the Paneth cell marker gene DEFA5 and the tuft cell marker gene POU2F3, while we also observed an increase in the related genes DEFB112 and POU4F2. Notably, these previous RNA sequencing studies were performed on heterogeneous mucosal populations of intestinal epithelial, immune and other cells within the mucosa, whereas our laser capture microscopy focused our analysis on the effects of RYGB, specifically on intestinal enterocytes, avoiding potential obfuscation by changes in gene expression in other cell types in the mucosa. In addition, each of these studies analyzed intestinal mucosal gene expression relatively early after surgery, while our study focused on a longer time interval by which a more steady state would have been expected to be achieved. This could have additionally contributed to differences between our observations and those of these other two studies.

The most statistically significant changing canonical pathway (Table 2), the cell cycle G2/M DNA damage checkpoint regulation, exhibited decreases in the AURKA, CKS1B, CKS2, and PLK1 genes after RYGB. The AURKA gene codes for the Aurora A protein which is involved in the disassembly of primary cilia necessary for the cell to enter into the cell cycle [33]. Dysregulation of cilium assembly/disassembly has been linked to a risk of obesity [34,35,36]. Cyclin-dependent kinases (CKs) (i.e., CKS1B, CKS2, Table S1) are also involved with cell cycle regulation, and inhibitors of CKs have drawn attention to their use in controlling diet-induced obesity [37]. Finally, Plk1 mRNA expression was upregulated in the visceral adipose tissue of obese non-diabetic patients compared to both control and obese diabetic patients [38].

The CDP-diacylglycerol biosynthesis pathway displayed increases in the SDHB and SDHC genes after RYGB surgery. CDP-diacylglycerol synthases are involved in regulating the growth in lipid droplets, fat storage, and adipocyte development [39,40]. The loss of SDHC in TH^+^ glomus cells leads to increased obesity in a mouse model. [41] However, SDHB-deficient mice were protected from obesity induced by a high fat diet [42]. These data and ours indicate that CDP-diacylglycerol synthases have a complex role in obesity that may be affected by diet.

After RYGB, the TCA cycle II pathway included increases in FAM20B and MAP3K9. FAM20B encodes for glycosaminoglycan xylosylkinase that is involved in the TCA cycle II pathway and is also involved in proteoglycan synthesis [43]. FAM20B was linked to obesity in a weight-discordant monozygotic twin study, where the adipose tissue matrisome had a positive association to the percent of liver fat, circulating triglycerides, and homeostatic model assessment of insulin resistance, each of which may contribute to metabolic dysfunction [43]. Single-nucleotide polymorphisms (SNPs) in MAP3K9 have been shown to interact with pre-menopausal obesity and diabetes [44]. Additionally, MAP3K9 is known to have a key role in regulating the JNK pathway, and is involved in the development of obesity [45]. 

Finally, the pyrimidine ribonucleotides interconversion pathway was characterized by increases in IFI35, IFNAR2, MED14, and TYK2. An interesting study involving human maternal-diet effect on offspring obesity examined the SNP interaction effect on the expression of various genes using DNA isolated from the blood buffy coat of offspring born before the mothers underwent gastrointestinal bypass surgery (BMS) or those born after bypass surgery (AMS) [46]. SNP rs35447805 had the most significant interaction with treatment for expression level of IFI35 where the IFI35 expression levels were a 0.94-fold decrease in expression for BMS heterozygous carriers and a 1.42-fold increase in expression for AMS heterozygous carriers [46]. Another study found IFI35 to have a decrease in expression from the subcutaneous adipose tissue of obese pigs [47]. MED14 has been implicated in the regulation of lipid homeostasis and adipogenesis via its interactions with the glucocorticoid receptor and PPARγ [48,49,50]. Finally, TYK2 has a role in the differentiation of Myf5^+^ brown adipocytes and when Tyk2 is lost in Tyk2^−/−^ mice they become obese with age [51]. Furthermore, diet-induced obese mice and obese humans have decreased Tyk2 expression; this can be reversed by constitutively active Stat3, which restores BAT differentiation and energy balance [51]. 

Next, we used weight loss as a measure of how well the patients responded to the gastric bypass treatment. We compared gene expression differences to weight loss based on changes in patient BMI (Figure 3 and Tables S2–S5). Additionally, we observed that the human enterocytic differentiation regulator, SLFN12, increased in protein expression in the post-gastric bypass endoscopic biopsies. We previously showed that the expression of SLFN12 immunostaining expression mirrors the mRNA expression in control vs. fasting duodenal human tissue [9] and that immunostaining for rat Slfn3 (orthologous to human SLFN12) mirrors Western blot-assayed protein and mRNA expression in rat intestinal tissue [8]. A study involving an in vitro human ulcerative colitis colon organoid model found that after 60 weeks of inflammatory stimulation the SLFN11 mRNA and protein expression was increased [52]. Additionally, the investigators described higher SLFN11 mRNA and protein expression in human endoscopic biopsy samples from patients with ulcerative colitis in comparison to endoscopic biopsies from healthy patients [52]. Given that little is known about the mechanisms that control SLFN protein expression, it is possible that similar mechanisms may influence GI mucosal changes in SLFN12 and SLFN11 in various disease states. Further analysis was performed to determine whether SLFN12 may regulate specific genes involved with differentiation and restitution of intestinal mucosa and obesity-related genes from the significant 74 genes observed above (Figure 3). The overexpression of SLFN12 in human HIEC-6 and/or FHs 74 Int cells led to a similar augmentation in the expression of the following differentiation and restitution genes: HES2, CARD9, and SLC19A2 and the following obesity-related genes: FBXW7, STXBP4, SPARCL1, and UTS in comparison with the increased expression observed in the RNA Seq data. The few genes that did not change similarly (HES2, TRPC5, SPARCL1 in FHs 74 Int, CARD9 in HIEC-6, and MBOAT5) between the RYGB RNA Seq and the HIEC-6/FHs 74 Int cell lines overexpressing SLFN12 could reflect differences in the biology of the individual cell lines or differences in the interaction between SLFN12 intracellular effects and those of other possible factors found in vivo but not in vitro (such as neuroendocrine [53], hormonal [54,55], growth factors [56,57,58,59], nutrients [60,61,62], extracellular matrix proteins [56,57,58], and physical forces [61,63,64,65,66]). Furthermore, since little is known about the factors that regulate SLFN12 expression itself, it is possible that some of the differences that we observed in vivo but not in vitro could have been upstream of SLFN12 and thus changed in association with, but not because of, SLFN12. However, most of the genes in Table 1 did change similarly between the RYGB RNA Seq and cell culture data, suggesting that SLFN12 may contribute to these gene changes that we observed after RYGB. 

While little is known about how SLFN12 itself is regulated, we have previously shown that the rodent ortholog Slfn3 is predominantly cytosolic and modestly nuclear in localization with exogenously expressed Slfn3 in IEC-6 cells. We then transfected Slfn3-null Caco2 cells with a Slfn3 plasmid containing a nuclear exclusion sequence and found that Slfn3 was confined to the cytoplasm and was still able to induce villin promoter activity [7]. SLFN12 itself is also predominantly cytosolic and only modestly nuclear in localization when exogenously adenovirally expressed in Caco-2 cells. [9] Indeed, and unlike long SLFNs such as SLFN11, SLFN12 modulates enterocytic differentiation in the cytosol by affecting the deubiquitylation and proteasomal degradation of the transcription factor CDX2, thus altering levels of newly synthesized CDX2 levels in the cytosol before that transcription factor moves back into the nucleus to bind to the chromatin [9].

Finally, with regard to genes which changed significantly in expression with respect to BMI (Figure 4, Table S5), these genes might reflect or contribute to the efficacy of the RYGB surgery. Ten of these genes are yet to be characterized and the other nine genes do not appear to be related to obesity or intestinal differentiation and restitution based on current literature review. Therefore, future studies would be warranted to determine the role of these 19 genes in adaptive mechanisms after RYGB and weight loss.

## 5. Conclusions

This study investigated the genes that specifically change only in intestinal enterocytes after human RYGB. RNA sequencing yielded canonical pathways that were affected favorably for obesity and its comorbidities. Correlating gene expression changes to the patient BMI, we were able to define gene sets associated with RYGB effectiveness. Finally, we demonstrated an increase in SLFN12 protein after RYGB and that overexpression of SLFN12 in intestinal cells leads to similar gene expression changes that were altered after RYGB.

## Figures and Tables

**Table 1:** Comparison of expression of differentiation, restitution, and obesity-related genes between RNA Seq and HIEC-6 and FHs74 Int in the in vitro data.

		RNA Seq	HIEC-6 + AdvSLFN12	FHs 74 Int + AdvSLFN12
Differentiation & Restitution genes	HES2	↑	↑	=
	CARD9	↑	=	↑
	TRPC5	↓	↓	↑
	SLC19A2	↑	↑	↑
Obesity–related genes	FBXW7	↑	↑	↑
	MBOAT2	↑	↓	↓
	STXBP4	↑	↑	↑
	SPARCL1	↑	↑	=
	UTS	↑	↑	↑

**Table 2:** Major canonical pathways affected by RYGB.

Top Canonical Pathways	*p*-Value	Overlap
Cell Cycle: G2/M DNA Damage Checkpoint Regulation	1.21 × 10^−3^	6/49 (AURKA, CKS1B, CKS2, PKMYT1, PLK1, TOP2A)
CDP-diacylglycerol Biosynthesis I	2.29 × 10^−2^	4/24 (ACO2, SDHB, SDHC, SUCLA2)
Phosphatidylglycerol Biosynthesis II (Nonplastidic)	2.50 × 10^−2^	3/12 (POLB, POLG, XRCC1)
TCA Cycle II (Eukaryotic)	2.5 × 10^−2^	6/66 (ADPGK, FAM20B, G6PC, GRK4, MAP3K9, PLK1)
Pyrimidine Ribonucleotides Interconversion	3.42 × 10^−2^	4/36 (IFI35, IFNAR2, MED14, TYK2)

## Data Availability

RNA sequencing data can be found at the following GEO link: https://www.ncbi.nlm.nih.gov/geo/info/seq.html (accessed on 1 November 2022).

## Supplementary Materials

The following supporting information can be downloaded at: https://www.mdpi.com/article/10.3390/cells11203283/s1. Table S1: Genes that changed significantly with differential expression analysis between Post-RYGB and Pre-RYGP samples. Table S2: Genes that changed significantly for post-RYGB samples with respect to the change in BMI using a regression analysis. Table S3: Genes that changed significantly with the combined effect of RYGB and BMI. Table S4: Genes that highly correlated with respect to changes in percentages of BMI when measured pre- and post- the RYGB condition. Table S5: 19 genes that changed their expression significantly from Table S4 with respect to the change in BMI.
