# Supplementary material for: RNA Sequencing of Intestinal Enterocytes Pre- and Post-Roux-en-Y Gastric Bypass Reveals Alteration in Gene Expression Related to Enterocyte Differentiation, Restitution, and Obesity with Regulation by Schlafen 12
Source: Cells. 2022 Oct 18;11(20):3283. doi: 10.3390/cells11203283 (PMC9601224; doi:10.3390/cells11203283)
Supplement: Supplementary file 1 [file cells-11-03283-s001.zip › Table S1.pdf]

Genes	mean in group	mean in group Pre	Mean_diff.PostVsPre	FC	pval
STXBP4	12.04	4.49	7.54	2.68	8.46E-04
LOC105375670	15.57	6.59	8.98	2.36	1.15E-03
TRPC5	2.79	6.89	-4.10	0.40	1.24E-03
SPARCL1	18.81	6.21	12.60	3.03	1.30E-03
LOC105378841	8.60	2.66	5.93	3.23	1.35E-03
SLC19A2	12.83	4.97	7.86	2.58	1.75E-03
UTS2	16.04	4.90	11.13	3.27	2.00E-03
CES1P1	4.79	1.05	3.74	4.55	2.19E-03
LOC105372092	5.96	1.89	4.07	3.16	2.21E-03
LOC101928978	10.75	4.35	6.39	2.47	2.22E-03
WDR37	8.06	4.51	3.55	1.79	2.23E-03
NABP1.OT1	10.33	1.01	9.33	10.26	2.39E-03
CNN1	9.51	0.97	8.54	9.81	2.41E-03
ERICH4	11.52	3.56	7.96	3.23	2.44E-03
AHSA1	10.17	2.55	7.62	3.99	2.44E-03
XPO7	12.97	4.09	8.87	3.17	2.47E-03
ZNF397	3.89	1.22	2.67	3.19	2.47E-03
LOC389705	16.92	3.12	13.80	5.42	2.54E-03
PLEKHG4	6.35	2.89	3.46	2.20	2.91E-03
LINC00678	9.26	3.33	5.93	2.78	3.08E-03
LOC107984098	20.06	1.84	18.22	10.88	3.13E-03
AKAP13	6.10	10.56	-4.46	0.58	3.21E-03
LOC105377963	11.50	1.33	10.17	8.66	3.24E-03
LOC105372725	7.53	2.38	5.15	3.17	3.32E-03
PTPRF	11.30	4.54	6.76	2.49	3.37E-03
CPNE3	5.52	14.64	-9.11	0.38	3.38E-03
ZNF417	7.33	3.54	3.78	2.07	3.49E-03
KLHL6	6.57	2.67	3.90	2.46	3.53E-03
TRNT1	6.88	3.42	3.47	2.02	3.58E-03
LOC105371155	12.43	1.62	10.81	7.69	3.98E-03
LINC00466	13.95	6.83	7.11	2.04	4.10E-03
TCTN3	3.99	17.39	-13.39	0.23	4.15E-03
C2CD4A	7.54	1.31	6.23	5.74	4.25E-03
EFCC1	5.80	2.12	3.68	2.73	4.32E-03
VPS13D	13.32	6.28	7.05	2.12	4.35E-03
PATJ	19.98	11.84	8.14	1.69	4.39E-03
LOC105378748	4.64	14.39	-9.74	0.32	4.40E-03
LOC105377927	11.87	1.34	10.53	8.84	4.52E-03
TUB	6.18	3.79	2.40	1.63	4.54E-03
EGFEM1P	11.71	6.05	5.66	1.94	4.59E-03
SLC26A6	10.11	0.72	9.39	14.07	4.62E-03
LINC01426	5.82	1.67	4.15	3.49	4.62E-03
ABLIM2	9.07	3.11	5.96	2.92	4.65E-03
GSN	7.92	4.15	3.77	1.91	4.68E-03
POFUT1	7.31	1.98	5.33	3.70	4.76E-03

LOC105379179	7.83	2.32	5.50	3.37	5.14E-03
ACOX3	8.91	2.02	6.88	4.40	5.16E-03
LOC440700	7.87	0.25	7.62	32.11	5.16E-03
LOC105379130	12.87	3.84	9.04	3.36	5.19E-03
LINC02669	9.64	1.94	7.69	4.96	5.22E-03
LOC107984880	8.94	1.22	7.72	7.34	5.23E-03
LINC01607	17.46	4.69	12.76	3.72	5.30E-03
LOC105376755	17.18	3.84	13.34	4.47	5.35E-03
BBS7	14.16	5.72	8.44	2.47	5.43E-03
LOC105375938	15.28	3.49	11.79	4.38	5.52E-03
SLC5A7	7.19	4.17	3.03	1.73	5.54E-03
LINC02774	16.16	5.78	10.38	2.79	5.81E-03
LOC105375749	9.18	2.43	6.75	3.78	6.02E-03
CFC1	3.35	0.97	2.38	3.46	6.02E-03
LOC105372877	9.91	2.92	6.99	3.39	6.27E-03
LOC285422	15.00	0.76	14.24	19.79	6.27E-03
DEF6	16.07	3.29	12.78	4.89	6.29E-03
MIATNB	8.38	1.32	7.06	6.35	6.39E-03
LOC105369890	12.28	6.96	5.32	1.76	6.42E-03
LOC107986617	9.55	2.12	7.43	4.51	6.53E-03
LOC105378128	10.78	2.00	8.78	5.39	6.59E-03
HIGD2B	7.15	1.82	5.33	3.93	6.60E-03
SLC35A3	8.65	4.92	3.73	1.76	6.61E-03
TAS2R1	10.88	3.30	7.58	3.30	6.64E-03
LOC112268086	8.95	3.48	5.46	2.57	6.70E-03
LOC339902	3.48	23.43	-19.94	0.15	6.74E-03
LOC112268467	5.41	2.54	2.88	2.13	6.78E-03
HES2	5.20	1.75	3.45	2.97	7.00E-03
CALB1	14.53	5.94	8.59	2.45	7.11E-03
DCTN1.AS1	9.00	3.98	5.02	2.26	7.21E-03
LPXN	12.28	5.04	7.24	2.44	7.23E-03
TMEM37	2.73	13.80	-11.07	0.20	7.33E-03
TMEM245	11.24	4.11	7.14	2.74	7.45E-03
LOC105376085	18.00	7.98	10.01	2.25	7.45E-03
LOC101927267	2.03	9.00	-6.97	0.23	7.49E-03
GALNT10	3.84	10.19	-6.34	0.38	7.56E-03
LOC105376817	18.92	3.40	15.52	5.57	7.64E-03
LOC101926960	8.95	1.17	7.78	7.65	7.65E-03
CNTNAP1	11.83	5.40	6.44	2.19	7.90E-03
G6PC	7.70	2.42	5.28	3.18	7.92E-03
NUDT19	5.23	12.82	-7.59	0.41	7.93E-03
NSRP1	7.43	15.50	-8.07	0.48	7.94E-03
SH3TC2	8.28	4.65	3.63	1.78	8.11E-03
REV1	11.00	4.00	7.00	2.75	8.13E-03
CDC45	13.55	4.86	8.68	2.79	8.20E-03
LOC105377562	16.37	2.97	13.41	5.52	8.23E-03
LOC112267972	13.32	3.77	9.55	3.54	8.30E-03

SPIB	8.08	1.70	6.38	4.75	8.30E-03
PRG4	14.47	6.13	8.34	2.36	8.37E-03
UTP11	9.37	2.93	6.44	3.20	8.51E-03
CNOT6L	12.21	7.72	4.50	1.58	8.64E-03
TLK2	12.48	6.27	6.21	1.99	8.79E-03
BRK1	7.75	1.79	5.96	4.34	8.81E-03
NSL1	11.20	5.07	6.13	2.21	8.86E-03
HLTF	16.59	5.56	11.03	2.99	8.87E-03
LOC105374594	18.22	4.94	13.27	3.69	8.87E-03
CENPO	7.83	2.14	5.69	3.66	8.89E-03
LOC107985241	9.22	2.32	6.90	3.97	8.93E-03
LOC101929650	4.80	1.29	3.51	3.73	9.09E-03
CREM	14.85	6.79	8.06	2.19	9.24E-03
LOC105378403	1.50	16.57	-15.06	0.09	9.32E-03
CACNA2D1.AS1	4.75	10.82	-6.07	0.44	9.43E-03
LINC00548	7.52	2.13	5.39	3.53	9.52E-03
LOC107985379	10.07	0.72	9.36	14.08	9.58E-03
SLC13A2	5.78	16.98	-11.20	0.34	9.67E-03
HSPH1	16.41	6.50	9.91	2.52	9.69E-03
LINC02542	14.92	5.03	9.89	2.97	9.73E-03
LOC107985037	6.21	14.66	-8.44	0.42	9.83E-03
LOC107986223	36.91	8.26	28.64	4.47	9.84E-03
LINC02128	8.61	0.57	8.04	15.04	9.89E-03
AP1S3	10.99	4.17	6.82	2.63	9.91E-03
LRFN1	7.66	1.26	6.40	6.06	9.94E-03
LMBRD1	8.16	15.55	-7.38	0.53	9.95E-03
DTWD2	11.02	5.82	5.20	1.89	9.97E-03
NUMB	12.28	4.54	7.74	2.71	1.00E-02
SPAG8	11.96	2.12	9.84	5.64	1.00E-02
WDR7	13.44	7.39	6.05	1.82	1.01E-02
LOC107986708	10.19	2.13	8.05	4.77	1.01E-02
DNAJA2	15.35	5.31	10.04	2.89	1.01E-02
NALCN.AS1	7.28	0.76	6.51	9.52	1.02E-02
COL5A2	20.82	9.76	11.05	2.13	1.03E-02
SLC4A8	9.22	6.47	2.75	1.43	1.03E-02
MEMO1	10.10	3.75	6.35	2.69	1.04E-02
COL22A1	14.84	5.26	9.59	2.82	1.05E-02
RPAP2	9.56	6.19	3.37	1.54	1.05E-02
CENPE	18.81	9.33	9.49	2.02	1.06E-02
LOC107984023	10.08	4.08	5.99	2.47	1.06E-02
PRSS55	0.42	4.30	-3.89	0.10	1.06E-02
EXOC5	11.83	7.05	4.78	1.68	1.07E-02
C3orf33	15.47	3.82	11.65	4.05	1.08E-02
RP9P	5.15	1.09	4.06	4.73	1.09E-02
ZNF740	3.12	7.32	-4.20	0.43	1.09E-02
ONECUT3	2.55	0.99	1.55	2.56	1.09E-02
LOC107985967	9.84	3.47	6.38	2.84	1.10E-02

PTPRG	7.38	12.76	-5.38	0.58	1.10E-02
LOC105369990	7.31	2.18	5.13	3.35	1.10E-02
RIPPLY2	3.29	0.39	2.91	8.52	1.11E-02
LOC107986367	11.08	4.06	7.02	2.73	1.12E-02
MIR3150BHG	8.39	4.08	4.31	2.06	1.13E-02
LOC105374959	14.59	2.73	11.85	5.34	1.14E-02
UVRAG.DT	6.29	1.12	5.17	5.60	1.15E-02
LOC105370175	6.28	1.25	5.03	5.02	1.15E-02
RNF157.AS1	4.91	0.97	3.94	5.08	1.15E-02
NR1H2	12.98	1.57	11.42	8.28	1.15E-02
PANX1	7.69	3.84	3.85	2.00	1.15E-02
LOC105374038	17.08	8.09	8.99	2.11	1.15E-02
PGAP1	11.94	7.00	4.94	1.71	1.16E-02
OR56B1	5.44	1.70	3.74	3.21	1.17E-02
GPN3	16.62	2.88	13.74	5.77	1.17E-02
LINC02020	18.68	2.12	16.55	8.79	1.19E-02
BAIAP2L2	6.52	2.66	3.86	2.45	1.19E-02
AUH	12.35	4.83	7.52	2.56	1.20E-02
EN2	4.95	13.20	-8.25	0.38	1.21E-02
DYRK4	4.18	9.21	-5.04	0.45	1.21E-02
LOC107984173	12.57	0.70	11.87	17.92	1.22E-02
CSMD2.AS1	15.25	2.68	12.57	5.70	1.22E-02
LPO	11.66	4.11	7.55	2.84	1.24E-02
PFDN4	9.99	3.65	6.34	2.74	1.25E-02
SGO2	11.82	6.32	5.51	1.87	1.26E-02
LOC107985188	7.00	0.41	6.59	16.96	1.27E-02
LINC02140	5.91	1.13	4.78	5.23	1.27E-02
LOC105376626	4.46	11.05	-6.59	0.40	1.27E-02
OTUD6B.AS1	11.23	4.38	6.84	2.56	1.27E-02
SNU13	6.07	1.71	4.36	3.55	1.29E-02
FBXW7	8.25	4.90	3.35	1.68	1.29E-02
SLCO2B1	8.70	1.96	6.73	4.43	1.29E-02
ATP13A4.AS1	10.25	3.62	6.63	2.83	1.30E-02
LOC100505851	10.11	2.45	7.66	4.12	1.30E-02
GOLGA6L9_1	0.84	0.03	0.82	32.12	1.30E-02
WDR1	9.33	2.37	6.96	3.93	1.30E-02
SLC4A5	10.03	5.23	4.80	1.92	1.31E-02
C1orf116	4.23	1.45	2.78	2.92	1.31E-02
TNS2	8.51	2.63	5.87	3.23	1.32E-02
S100A7A	9.27	2.19	7.08	4.24	1.32E-02
LOC105373831	11.18	4.10	7.07	2.72	1.32E-02
LOC105371899	9.32	0.50	8.82	18.57	1.32E-02
LOC107986119	8.97	1.63	7.34	5.50	1.33E-02
LOC105373234	8.81	4.98	3.83	1.77	1.34E-02
LINC00934	5.52	1.52	3.99	3.62	1.34E-02
PLAA	8.42	3.51	4.90	2.40	1.34E-02
DHRS7C	3.06	0.22	2.84	13.76	1.35E-02

TMEM131	18.54	7.96	10.58	2.33	1.35E-02
RPL23AP7	10.38	0.73	9.66	14.29	1.35E-02
MFAP5	17.62	5.45	12.17	3.23	1.36E-02
NCAPG2	9.15	17.33	-8.18	0.53	1.36E-02
TANC1	11.40	5.54	5.86	2.06	1.37E-02
CSH1	14.64	3.53	11.11	4.15	1.37E-02
HRK	4.19	8.04	-3.85	0.52	1.37E-02
ACO2	8.88	3.93	4.95	2.26	1.37E-02
LOC613266	4.03	12.27	-8.25	0.33	1.38E-02
LOC105371951	11.85	1.74	10.10	6.79	1.38E-02
LOC284379	11.16	3.20	7.96	3.48	1.39E-02
LOC107985256	7.73	2.45	5.28	3.15	1.39E-02
LINC01205	6.68	15.86	-9.18	0.42	1.39E-02
ATP11A	11.27	6.55	4.72	1.72	1.40E-02
LOC101927947	6.58	2.02	4.57	3.26	1.40E-02
LOC105373973	7.95	2.40	5.55	3.31	1.41E-02
POLD2	8.47	2.03	6.44	4.17	1.41E-02
LOC105371873	11.41	4.43	6.98	2.58	1.43E-02
TMEM147.AS1	5.13	0.85	4.27	6.00	1.44E-02
SLC26A9.AS1	2.35	0.28	2.07	8.41	1.44E-02
BICDL1	4.46	10.67	-6.21	0.42	1.44E-02
LINC01497	9.64	3.46	6.18	2.79	1.44E-02
LOC107984302	5.27	1.14	4.13	4.61	1.44E-02
TEX13C	2.54	8.21	-5.67	0.31	1.44E-02
CCDC127	6.18	2.69	3.49	2.30	1.45E-02
LOC100996574	7.19	0.73	6.46	9.83	1.46E-02
GUCY1B1	12.13	5.22	6.90	2.32	1.47E-02
FLRT1	2.71	5.54	-2.83	0.49	1.47E-02
MEOX1	8.06	0.88	7.18	9.16	1.47E-02
FOCAD	16.57	8.60	7.97	1.93	1.49E-02
DSC1	16.49	7.73	8.76	2.13	1.49E-02
OR52B4	7.52	2.00	5.52	3.76	1.49E-02
LOC102723536	13.37	2.38	10.99	5.63	1.51E-02
LOC107985584	3.25	0.00	3.25	3.25	1.51E-02
NDST1	4.81	2.05	2.76	2.35	1.53E-02
LOC105375046	9.97	1.68	8.29	5.93	1.54E-02
LOC105378464	34.89	8.18	26.71	4.26	1.54E-02
LOC105370538	14.25	4.31	9.93	3.30	1.56E-02
IGLON5	9.63	5.13	4.50	1.88	1.56E-02
PLK1	10.95	2.49	8.46	4.39	1.56E-02
C11orf53	14.19	4.44	9.75	3.20	1.56E-02
LOC105376357	17.21	0.00	17.21	17.21	1.57E-02
HSPA13	9.03	3.78	5.25	2.39	1.57E-02
PHACTR4	11.42	4.89	6.53	2.33	1.57E-02
NXPE2	8.89	5.56	3.33	1.60	1.59E-02
PEX3	12.34	5.31	7.03	2.32	1.59E-02
FAM242B	1.40	15.58	-14.18	0.09	1.59E-02

MANBAL	4.21	15.34	-11.13	0.27	1.59E-02
LOC105374981	4.99	15.66	-10.68	0.32	1.60E-02
LOC105374217	24.84	4.63	20.21	5.37	1.61E-02
LOC107986813	4.65	11.50	-6.85	0.40	1.61E-02
AAGAB	21.19	5.75	15.44	3.69	1.61E-02
LOC105370304	13.23	4.53	8.70	2.92	1.61E-02
CCDC190	9.28	4.69	4.60	1.98	1.61E-02
LOC107985541	2.60	7.67	-5.06	0.34	1.62E-02
TRAPPC12	0.97	0.12	0.85	8.22	1.62E-02
LINC01359	27.30	8.06	19.24	3.39	1.63E-02
NOA1	8.90	2.79	6.11	3.19	1.63E-02
LOC105371822	5.81	3.31	2.51	1.76	1.65E-02
PHIP	11.32	5.85	5.47	1.93	1.65E-02
AQP12B	3.31	0.13	3.18	25.72	1.65E-02
ERO1B	8.55	5.37	3.18	1.59	1.66E-02
NFKBIZ	9.55	3.58	5.96	2.66	1.66E-02
MBD3	6.69	1.86	4.83	3.60	1.66E-02
CKM	5.36	1.68	3.68	3.19	1.66E-02
LOC107986260	4.45	0.88	3.58	5.07	1.67E-02
GRK4	9.73	4.74	5.00	2.06	1.67E-02
TBC1D15	17.80	10.83	6.97	1.64	1.67E-02
DENND4B	8.83	3.63	5.20	2.43	1.67E-02
TCF7L1.IT1	15.70	1.05	14.65	14.97	1.68E-02
LOC158434	14.64	2.65	12.00	5.53	1.68E-02
PTCSC2	17.72	4.11	13.60	4.31	1.69E-02
LOC105379134	51.89	4.87	47.01	10.65	1.70E-02
LOC105372518	17.37	3.07	14.31	5.67	1.70E-02
NETO1	11.22	4.76	6.45	2.36	1.71E-02
R3HCC1	1.38	9.22	-7.84	0.15	1.71E-02
FLYWCH1	7.74	4.11	3.63	1.88	1.72E-02
DEPDC4	11.94	5.50	6.45	2.17	1.72E-02
MRPS6	4.57	0.10	4.47	44.29	1.72E-02
TTC38	7.25	4.06	3.19	1.78	1.73E-02
LOC105371449	6.06	3.15	2.91	1.92	1.73E-02
D21S2088E	4.76	26.18	-21.42	0.18	1.73E-02
FGF10	7.97	4.83	3.15	1.65	1.74E-02
LOC105374475	12.33	6.97	5.37	1.77	1.74E-02
BMPR1B.DT	21.37	2.89	18.47	7.38	1.74E-02
LOC101929498	15.16	6.80	8.36	2.23	1.75E-02
FAM218A	9.71	3.72	5.99	2.61	1.76E-02
DPH3	11.60	4.50	7.09	2.57	1.76E-02
DARS1.AS1	5.42	0.43	4.99	12.70	1.76E-02
LOC105371414	6.65	0.49	6.16	13.64	1.77E-02
MRPL51	10.48	2.16	8.32	4.86	1.77E-02
LOC105379065	12.82	5.25	7.57	2.44	1.78E-02
LOC107983961	8.86	1.27	7.58	6.95	1.79E-02
LOC105370518	6.88	4.70	2.17	1.46	1.79E-02

RASAL2	8.91	5.60	3.31	1.59	1.79E-02
SDHB	11.13	2.86	8.26	3.88	1.82E-02
LOC107986031	7.12	1.65	5.47	4.31	1.82E-02
PSMG4	8.65	4.61	4.05	1.88	1.82E-02
LOC105369709	8.09	2.16	5.94	3.75	1.83E-02
LOC112267894	21.61	2.32	19.29	9.30	1.83E-02
OR1L8	11.38	4.13	7.25	2.75	1.83E-02
ITM2B	9.72	5.04	4.68	1.93	1.84E-02
LOC105373989	2.78	0.00	2.78	2.78	1.85E-02
PPM1B	13.00	4.99	8.01	2.60	1.85E-02
ERFE	3.19	0.41	2.78	7.70	1.86E-02
LOC105372480	16.34	2.47	13.87	6.61	1.86E-02
SAMD15	11.78	7.12	4.66	1.65	1.86E-02
NFE2L3	11.93	3.84	8.09	3.11	1.86E-02
LOC107986962	9.62	1.77	7.85	5.44	1.88E-02
MADCAM1	0.35	2.92	-2.57	0.12	1.89E-02
LOC105376287	5.47	1.60	3.87	3.42	1.89E-02
LOC112267935	4.69	1.60	3.09	2.94	1.90E-02
LOC105376205	4.84	12.16	-7.32	0.40	1.91E-02
ZNF671	3.40	10.01	-6.61	0.34	1.91E-02
STARD3	2.52	6.33	-3.81	0.40	1.92E-02
LOC284412	2.33	14.57	-12.24	0.16	1.92E-02
MYO6	16.31	6.92	9.39	2.36	1.92E-02
CPO	8.37	4.38	4.00	1.91	1.93E-02
STN1	5.63	13.80	-8.17	0.41	1.94E-02
LSM5	16.96	8.23	8.73	2.06	1.94E-02
TSPY26P	3.20	0.08	3.12	40.46	1.94E-02
ASB10	8.00	1.31	6.70	6.13	1.95E-02
LOC100131532	8.30	3.07	5.23	2.70	1.95E-02
LOC105378029	11.22	4.02	7.20	2.79	1.95E-02
LOC105377152	4.58	16.92	-12.34	0.27	1.96E-02
KLHL11	9.10	3.77	5.33	2.41	1.96E-02
LINC01833	3.27	8.60	-5.33	0.38	1.96E-02
LOC100996598	16.84	1.54	15.30	10.93	1.97E-02
RPS6KA4	2.95	8.85	-5.89	0.33	1.97E-02
LOC107986514	7.46	2.28	5.19	3.28	1.99E-02
MRGPRF.AS1	11.23	2.38	8.85	4.72	2.00E-02
LINC02286	11.50	5.96	5.54	1.93	2.01E-02
SRSF9	2.87	0.33	2.54	8.61	2.02E-02
STAM.AS1	9.49	2.25	7.25	4.23	2.02E-02
FAIM2	3.03	0.86	2.18	3.54	2.03E-02
LOC107986953	18.69	1.58	17.11	11.80	2.03E-02
KIAA1522	10.55	3.08	7.47	3.43	2.03E-02
MROH5	4.60	1.62	2.98	2.85	2.03E-02
BBS5	12.38	4.36	8.03	2.84	2.04E-02
LOC105377331	9.29	0.00	9.29	9.29	2.04E-02
SLC66A2	4.65	1.51	3.15	3.09	2.04E-02

LOC105374235	8.22	4.49	3.73	1.83	2.05E-02
LOC105370531	5.70	2.58	3.12	2.21	2.05E-02
LINC02681	23.72	7.41	16.31	3.20	2.06E-02
IL26	7.66	20.27	-12.60	0.38	2.06E-02
ZNF430	10.48	5.43	5.05	1.93	2.07E-02
C1orf220	11.87	2.91	8.96	4.08	2.08E-02
MAGEF1	6.33	0.98	5.34	6.43	2.09E-02
LOC105372683	8.86	3.60	5.26	2.46	2.09E-02
LOC102723427	16.38	57.66	-41.28	0.28	2.09E-02
PYGL	16.78	6.70	10.08	2.51	2.10E-02
HAO2	6.11	9.23	-3.11	0.66	2.10E-02
LOC105371157	8.41	0.30	8.10	27.65	2.10E-02
ZNF771	0.38	5.00	-4.62	0.08	2.11E-02
LOC105379027	8.61	1.27	7.35	6.80	2.11E-02
KRT34	0.46	0.07	0.40	7.02	2.11E-02
LOC105379378	8.88	0.38	8.50	23.30	2.11E-02
HEXA.AS1	6.49	2.44	4.05	2.66	2.11E-02
FPR2	17.19	5.38	11.81	3.20	2.12E-02
LOC105370952	10.67	3.09	7.58	3.45	2.12E-02
STX10	4.98	0.63	4.35	7.92	2.12E-02
LINC02505	10.85	40.37	-29.52	0.27	2.12E-02
CCDC74B	7.76	2.02	5.74	3.85	2.13E-02
KIF3C	3.65	11.80	-8.15	0.31	2.13E-02
CDC42SE2	9.70	3.23	6.47	3.01	2.14E-02
TNFSF13	3.49	0.00	3.49	3.49	2.15E-02
LOC107986769	13.57	4.76	8.80	2.85	2.16E-02
POLG	5.72	3.53	2.19	1.62	2.16E-02
LOC107985141	8.78	3.70	5.08	2.37	2.16E-02
SLMAP	10.62	4.83	5.79	2.20	2.16E-02
CATSPERE	14.96	8.50	6.45	1.76	2.17E-02
NNT.AS1	9.23	1.69	7.53	5.45	2.17E-02
KRT71	13.94	5.08	8.86	2.74	2.18E-02
AOC3	6.07	1.82	4.24	3.33	2.18E-02
LINC02408	11.89	4.24	7.65	2.80	2.18E-02
MGST3	20.91	3.48	17.43	6.01	2.18E-02
EFEMP1	9.54	2.98	6.56	3.20	2.19E-02
ZBTB5	4.85	1.67	3.18	2.90	2.20E-02
ITGBL1	4.19	2.21	1.97	1.89	2.20E-02
LOC100128882	10.26	2.37	7.90	4.34	2.20E-02
SEC22B4P	4.09	1.81	2.28	2.26	2.20E-02
LINC01506	13.70	0.90	12.81	15.30	2.21E-02
LOC107983950	4.40	0.15	4.25	29.90	2.21E-02
LOC107986782	6.33	1.57	4.76	4.03	2.21E-02
NF1	14.74	8.71	6.04	1.69	2.22E-02
LOC105369961	8.65	0.59	8.06	14.57	2.23E-02
LOC105369408	12.51	3.41	9.10	3.67	2.24E-02
CELSR1	5.60	2.55	3.05	2.20	2.26E-02

TCEANC2	9.68	4.58	5.10	2.11	2.26E-02
DSTNP2	6.80	2.43	4.37	2.79	2.27E-02
SPOUT1	5.49	1.68	3.81	3.27	2.27E-02
LINC02724	5.20	0.89	4.31	5.86	2.28E-02
GALNTL5	10.65	4.64	6.01	2.30	2.28E-02
ZNF474.AS1	5.49	10.01	-4.52	0.55	2.28E-02
LINC02442	10.03	3.35	6.69	3.00	2.29E-02
ELL2	6.00	9.20	-3.20	0.65	2.29E-02
LOC107984387	10.72	4.69	6.03	2.28	2.29E-02
NAGS	3.74	0.67	3.06	5.54	2.30E-02
LRRC59	3.85	11.28	-7.44	0.34	2.30E-02
LOC105370739	10.26	2.59	7.67	3.96	2.30E-02
SNX7	13.87	4.39	9.48	3.16	2.31E-02
ADORA1	5.99	1.31	4.69	4.59	2.31E-02
PLPPR5	7.06	3.08	3.99	2.30	2.31E-02
LOC105370593	10.98	5.09	5.89	2.16	2.32E-02
PPP4R2	8.90	5.94	2.96	1.50	2.34E-02
MIR4453HG	3.67	1.46	2.21	2.52	2.35E-02
DEFB112	16.46	2.02	14.44	8.14	2.36E-02
CHODL	8.79	4.54	4.24	1.93	2.36E-02
MAB21L4	7.90	1.69	6.21	4.68	2.36E-02
LOC107986263	15.57	7.63	7.94	2.04	2.36E-02
LOC105374124	13.71	3.43	10.28	4.00	2.37E-02
LOC105377743	9.86	3.43	6.43	2.87	2.37E-02
PDIA5	5.54	13.82	-8.28	0.40	2.37E-02
CD2AP	19.11	7.47	11.65	2.56	2.37E-02
NDUFA12	10.86	4.34	6.53	2.51	2.37E-02
MAP11	4.75	0.28	4.47	16.76	2.37E-02
CLVS1	9.49	2.93	6.57	3.24	2.39E-02
LOC105374115	6.75	1.51	5.24	4.47	2.40E-02
BAZ2B	14.40	6.79	7.61	2.12	2.40E-02
TIAM2	11.39	5.40	5.99	2.11	2.40E-02
ETS1.AS1	2.29	12.57	-10.28	0.18	2.40E-02
LOC105372696	3.11	14.61	-11.49	0.21	2.41E-02
LOC101928972	10.08	1.20	8.88	8.39	2.42E-02
CECR3	7.85	1.24	6.62	6.36	2.42E-02
VWC2	9.13	5.01	4.12	1.82	2.44E-02
PKD2	8.66	4.02	4.65	2.16	2.44E-02
LOC105373895	20.03	3.99	16.04	5.02	2.44E-02
CMTR2_1	0.82	0.00	0.82	0.82	2.45E-02
LOC105372669	5.54	14.10	-8.56	0.39	2.45E-02
LOC107986240	46.91	3.72	43.19	12.60	2.45E-02
PLAGL1	17.77	4.77	13.00	3.73	2.45E-02
LOC101927817	9.45	2.56	6.88	3.68	2.47E-02
BRDT	19.88	8.79	11.09	2.26	2.47E-02
LOC102724475	12.92	4.33	8.60	2.99	2.48E-02
PNMA1	2.01	8.41	-6.41	0.24	2.48E-02

LINC00976	17.80	3.63	14.16	4.90	2.49E-02
MED14	7.57	4.16	3.41	1.82	2.49E-02
LOC105374796	3.98	0.51	3.47	7.84	2.51E-02
C19orf18	24.83	4.09	20.74	6.08	2.52E-02
BECN1	6.34	2.18	4.16	2.90	2.52E-02
LOC107986169	12.45	3.19	9.26	3.90	2.53E-02
ABCA6	16.59	10.89	5.70	1.52	2.53E-02
ARMC10	6.04	1.78	4.26	3.40	2.54E-02
HTR2C	7.17	3.13	4.04	2.29	2.54E-02
LYRM4.AS1	10.31	3.88	6.44	2.66	2.55E-02
LPP.AS2	3.00	0.37	2.63	8.09	2.55E-02
LOC105370462	9.00	2.72	6.28	3.31	2.56E-02
LRP2BP	4.26	8.10	-3.84	0.53	2.56E-02
EHHADH	5.84	16.36	-10.53	0.36	2.57E-02
OR2L2	5.70	18.28	-12.58	0.31	2.57E-02
SPZ1	3.61	12.25	-8.65	0.29	2.58E-02
RCL1	11.94	3.49	8.45	3.42	2.58E-02
LOC388813	11.57	2.09	9.48	5.55	2.58E-02
ZNF738	14.42	8.52	5.89	1.69	2.59E-02
HIRIP3	4.01	0.76	3.25	5.25	2.62E-02
LOC105378010	22.03	8.07	13.96	2.73	2.62E-02
CFP	5.66	1.82	3.84	3.11	2.63E-02
LOC105375742	2.04	10.74	-8.70	0.19	2.64E-02
UBE2K	12.43	3.83	8.61	3.25	2.64E-02
LINC01834	7.12	1.14	5.98	6.25	2.64E-02
ADAMTS3	15.95	7.76	8.19	2.06	2.64E-02
GLS	12.75	8.46	4.29	1.51	2.64E-02
ARCN1	5.87	17.21	-11.34	0.34	2.64E-02
TREM1	4.44	8.78	-4.34	0.51	2.64E-02
LAMTOR5.AS1	11.94	0.16	11.78	74.58	2.66E-02
SPOCD1	6.63	1.81	4.81	3.66	2.66E-02
MXD3	1.85	0.07	1.78	26.66	2.66E-02
TRPC7	8.70	3.36	5.34	2.59	2.66E-02
LRIT1	5.64	1.21	4.43	4.68	2.66E-02
LOC105374664	15.91	6.06	9.85	2.62	2.67E-02
TMEM63A	6.77	2.66	4.11	2.54	2.68E-02
DHX8	5.83	11.12	-5.29	0.52	2.68E-02
OR8S1	6.59	0.16	6.44	41.74	2.68E-02
CCDC54	20.33	3.91	16.42	5.20	2.68E-02
MEG3	12.53	5.15	7.38	2.43	2.69E-02
RNPS1	13.07	3.20	9.87	4.09	2.69E-02
GSE1	6.52	3.57	2.95	1.82	2.70E-02
LOC107984361	3.90	8.96	-5.06	0.44	2.70E-02
LOC105377085	8.19	3.87	4.32	2.12	2.70E-02
CYP7B1	8.37	5.63	2.75	1.49	2.71E-02
PLA2G2C	5.02	1.97	3.05	2.55	2.71E-02
LINC02075	9.36	1.10	8.26	8.51	2.72E-02

LOC112268016	4.43	2.41	2.02	1.84	2.72E-02
LINC00528	3.39	0.82	2.56	4.12	2.73E-02
LINC00543	5.25	1.34	3.91	3.92	2.73E-02
FAM131A	7.28	1.13	6.15	6.43	2.74E-02
OTOL1	6.27	2.41	3.86	2.60	2.74E-02
ALG10B	10.52	4.74	5.78	2.22	2.74E-02
FARP2	9.83	2.88	6.95	3.41	2.74E-02
HECW2.AS1	23.88	6.86	17.01	3.48	2.75E-02
LOC105371544	28.03	9.81	18.22	2.86	2.75E-02
LOC102723348	2.07	10.92	-8.85	0.19	2.75E-02
ABTB1	2.89	8.93	-6.04	0.32	2.76E-02
LINC01104	6.91	1.76	5.15	3.93	2.76E-02
EPX	4.24	1.40	2.84	3.03	2.76E-02
ENPP5	10.23	3.19	7.03	3.20	2.77E-02
TMEM230	12.41	5.53	6.88	2.24	2.77E-02
GLCE	7.28	12.00	-4.71	0.61	2.77E-02
EME1	8.95	2.78	6.18	3.22	2.77E-02
TRAV23DV6	9.38	0.41	8.97	22.75	2.78E-02
LOC102723370	9.15	29.63	-20.47	0.31	2.79E-02
LOC100508046	4.55	0.87	3.69	5.26	2.79E-02
VPS52_5	0.32	0.00	0.32	0.32	2.79E-02
RBM23	9.12	4.90	4.22	1.86	2.81E-02
ERC2	12.11	8.94	3.17	1.36	2.81E-02
AZI2	4.56	0.85	3.71	5.35	2.81E-02
LOC105376281	8.14	1.32	6.81	6.15	2.82E-02
LINC01415	8.26	4.47	3.79	1.85	2.82E-02
ATP8B5P	11.90	6.51	5.39	1.83	2.82E-02
LOC105370960	5.80	1.67	4.13	3.47	2.82E-02
YES1	8.77	5.43	3.34	1.61	2.83E-02
PHTF1	14.67	9.27	5.41	1.58	2.83E-02
ZDHHC2	3.31	1.45	1.86	2.28	2.83E-02
PRSS40B	0.52	0.00	0.52	0.52	2.83E-02
LOC107984270	9.80	3.78	6.02	2.59	2.84E-02
TRIM64B	8.80	28.06	-19.26	0.31	2.84E-02
GTF3A	13.52	2.85	10.67	4.74	2.84E-02
LOC105372551	4.85	14.64	-9.79	0.33	2.85E-02
KDM3A	21.25	11.28	9.96	1.88	2.85E-02
TBPL2	14.33	7.30	7.03	1.96	2.86E-02
LOC101927948	15.76	1.75	14.01	8.99	2.87E-02
AADAC	24.90	10.80	14.10	2.30	2.87E-02
CXXC4	7.62	4.56	3.06	1.67	2.88E-02
ZC3HAV1	8.62	3.78	4.84	2.28	2.88E-02
OR5B21	11.40	3.98	7.42	2.87	2.89E-02
CKS2	9.57	0.97	8.60	9.82	2.90E-02
BIRC8	10.01	2.55	7.47	3.93	2.90E-02
LOC101928168	8.80	2.44	6.37	3.61	2.90E-02
CIPC	4.36	12.73	-8.36	0.34	2.90E-02

NANOGP8	4.15	1.39	2.77	2.99	2.90E-02
COL4A3	11.75	4.31	7.44	2.73	2.90E-02
LOC105369149	4.75	0.84	3.91	5.65	2.90E-02
LOC107986887	5.04	0.67	4.37	7.50	2.91E-02
LOC107986167	12.42	8.63	3.79	1.44	2.91E-02
LOC102725148	11.33	5.26	6.07	2.15	2.92E-02
LOC105372562	3.48	16.57	-13.09	0.21	2.92E-02
TAF8	5.99	3.47	2.53	1.73	2.93E-02
MAFG.DT	2.38	0.49	1.89	4.87	2.93E-02
MIR3974	39.52	13.22	26.30	2.99	2.94E-02
CSN1S1	22.22	12.01	10.21	1.85	2.95E-02
DDHD1	12.02	6.71	5.31	1.79	2.96E-02
LOC105378337	26.83	7.02	19.81	3.82	2.96E-02
GJD2	3.33	10.89	-7.56	0.31	2.96E-02
ADGRG3	4.19	1.86	2.34	2.26	2.96E-02
IGHVII.78.1	7.65	0.00	7.65	7.65	2.97E-02
LCMT1.AS2	5.13	1.94	3.19	2.64	2.97E-02
LOC105369388	10.68	4.30	6.38	2.49	2.97E-02
LINC00444	9.72	2.82	6.89	3.44	2.97E-02
CISD2	9.65	3.67	5.98	2.63	2.98E-02
FHOD3	5.40	9.26	-3.86	0.58	2.98E-02
ACTR1A	5.76	3.03	2.74	1.90	2.99E-02
NDST4	11.89	5.51	6.39	2.16	2.99E-02
CKS1B	10.90	2.81	8.09	3.88	2.99E-02
LOC107986833	5.25	0.76	4.50	6.94	2.99E-02
LOC101928424	17.20	1.46	15.74	11.75	2.99E-02
ANKRD20A21P	18.53	10.16	8.37	1.82	3.00E-02
ATP1B3	17.66	5.68	11.98	3.11	3.00E-02
LOC105377582	16.61	4.87	11.73	3.41	3.00E-02
LOC105371203	9.96	2.78	7.18	3.58	3.01E-02
LOC105374363	8.80	0.28	8.52	31.98	3.02E-02
ENC1	7.95	4.15	3.80	1.91	3.02E-02
MYRIP	8.48	4.28	4.19	1.98	3.02E-02
TTYH3	5.09	0.75	4.34	6.82	3.03E-02
GACAT1	27.20	10.29	16.91	2.64	3.03E-02
LOC101927432	9.90	5.59	4.32	1.77	3.03E-02
SMIM23	11.06	1.81	9.25	6.10	3.04E-02
MT3	10.31	0.71	9.60	14.52	3.04E-02
LOC101928180	9.09	3.32	5.77	2.74	3.04E-02
ZNF789	12.04	6.64	5.40	1.81	3.05E-02
LINC01147	20.78	2.66	18.12	7.80	3.07E-02
PLPP4	3.68	7.18	-3.50	0.51	3.07E-02
S1PR3	10.01	2.17	7.84	4.61	3.07E-02
LINC00449	27.63	3.51	24.12	7.87	3.08E-02
NR2F1	7.14	1.86	5.29	3.85	3.08E-02
ESPN	1.94	4.92	-2.98	0.39	3.08E-02
INHBB	6.63	2.89	3.74	2.30	3.10E-02

THUMPD1	11.53	4.35	7.19	2.65	3.10E-02
LOC105374974	8.11	33.20	-25.09	0.24	3.10E-02
LOC102723862	10.22	4.44	5.78	2.30	3.11E-02
CA8	8.92	4.10	4.83	2.18	3.12E-02
LOC105372394	41.74	5.74	36.00	7.27	3.12E-02
GPX8	8.24	16.70	-8.46	0.49	3.12E-02
NIM1K	1.67	8.18	-6.51	0.20	3.13E-02
LOC105370588	13.17	5.31	7.87	2.48	3.13E-02
LINC02341	8.48	2.95	5.53	2.88	3.13E-02
ZNF70	4.26	7.85	-3.59	0.54	3.13E-02
COMMD6	9.33	3.36	5.97	2.78	3.14E-02
GUCY1B2	14.08	5.48	8.60	2.57	3.14E-02
PLEKHG3	5.91	1.41	4.50	4.20	3.15E-02
STK38L	11.93	6.92	5.01	1.72	3.15E-02
LINC02464	7.16	1.92	5.25	3.74	3.15E-02
TEX30	12.08	3.86	8.22	3.13	3.16E-02
RAD51AP1	6.94	29.69	-22.76	0.23	3.16E-02
LOC105378375	4.26	12.79	-8.52	0.33	3.16E-02
FPGT	8.85	2.65	6.19	3.34	3.16E-02
ZMYND15	6.97	2.93	4.03	2.38	3.16E-02
EXOC6B	11.72	6.83	4.89	1.72	3.17E-02
LOC105374636	15.51	7.50	8.01	2.07	3.17E-02
LPL	5.22	24.95	-19.73	0.21	3.17E-02
ADAMTS1	9.55	4.39	5.16	2.18	3.18E-02
EIF3B	15.08	6.70	8.38	2.25	3.18E-02
LOC105372026	5.10	9.13	-4.03	0.56	3.18E-02
FGF2	13.08	5.61	7.47	2.33	3.19E-02
LOC102723480	7.04	1.89	5.15	3.73	3.19E-02
LOC101929534	6.73	10.44	-3.71	0.64	3.20E-02
CFAP58.DT	12.83	2.18	10.65	5.88	3.20E-02
LINC02362	24.68	4.86	19.82	5.08	3.21E-02
B3GALT1	12.01	5.69	6.32	2.11	3.21E-02
ASPRV1	8.03	0.26	7.76	30.31	3.21E-02
LOC102724378	6.31	0.13	6.18	47.70	3.22E-02
MRPL11	10.17	1.40	8.77	7.29	3.22E-02
LINC01818	10.28	3.69	6.59	2.79	3.23E-02
TIA1	10.90	4.43	6.47	2.46	3.23E-02
LOC105377412	7.99	1.52	6.47	5.26	3.23E-02
KCNH2	3.87	8.62	-4.75	0.45	3.24E-02
LINC02068	15.11	5.00	10.11	3.02	3.24E-02
TAL2	19.40	2.21	17.18	8.76	3.24E-02
CREB3L2.AS1	5.12	25.79	-20.66	0.20	3.25E-02
IL1R2	9.96	4.84	5.12	2.06	3.25E-02
MFAP1	20.70	5.48	15.22	3.78	3.25E-02
LOC107984653	18.18	1.82	16.35	9.97	3.25E-02
UCN	5.52	0.00	5.52	5.52	3.26E-02
CNN3	18.33	5.40	12.94	3.40	3.26E-02

LINC02284	8.79	18.38	-9.59	0.48	3.26E-02
LOC107984690	2.07	6.34	-4.27	0.33	3.26E-02
KPTN	9.32	3.29	6.03	2.83	3.26E-02
TRPA1	9.38	25.65	-16.26	0.37	3.26E-02
LOC105375703	14.34	2.32	12.02	6.19	3.27E-02
LOC105374748	3.71	13.09	-9.39	0.28	3.27E-02
CALCOCO2	11.18	5.15	6.03	2.17	3.27E-02
LOC105375773	14.53	1.62	12.91	8.97	3.27E-02
LOC112267900	9.02	4.82	4.20	1.87	3.28E-02
FAM20B	10.95	4.39	6.56	2.49	3.28E-02
ACAD10	9.69	3.65	6.04	2.66	3.29E-02
LOC105375993	8.66	2.29	6.37	3.78	3.30E-02
SNRNP48	9.46	4.89	4.57	1.93	3.30E-02
LOC107984643	3.11	0.61	2.50	5.12	3.30E-02
LOC105369383	7.02	2.74	4.28	2.56	3.30E-02
TMEM117	13.32	4.39	8.93	3.03	3.31E-02
THG1L	13.36	3.83	9.53	3.49	3.31E-02
SLC6A7	8.75	2.14	6.62	4.09	3.31E-02
PRRX1	8.94	3.90	5.04	2.29	3.32E-02
LOC105375230	7.54	2.53	5.02	2.98	3.32E-02
SOBP	6.44	3.14	3.29	2.05	3.32E-02
LOC105373228	11.48	0.81	10.67	14.22	3.33E-02
CDRT7	14.29	2.46	11.84	5.82	3.34E-02
LRRC47	6.33	2.34	3.99	2.71	3.34E-02
LOC105370672	4.01	16.11	-12.09	0.25	3.35E-02
SUGCT	6.72	14.87	-8.14	0.45	3.35E-02
LOC105371235	3.67	1.07	2.60	3.42	3.36E-02
LINC01975	3.87	1.07	2.80	3.61	3.36E-02
KHK	3.31	0.72	2.59	4.60	3.37E-02
MBOAT2	10.54	4.79	5.76	2.20	3.37E-02
LOC105372679	17.16	4.29	12.88	4.00	3.37E-02
LOC105374652	20.01	4.84	15.17	4.13	3.38E-02
LOC105377410	14.01	3.53	10.48	3.96	3.38E-02
KRTAP4.4	9.01	2.82	6.18	3.19	3.38E-02
LRRC18	3.34	9.95	-6.61	0.34	3.39E-02
LOC107984546	8.14	3.22	4.92	2.52	3.39E-02
HHLA1	5.16	10.27	-5.11	0.50	3.40E-02
LOC105370071	7.91	2.16	5.75	3.66	3.41E-02
LINC01638	15.21	3.74	11.47	4.06	3.41E-02
LOC107986920	12.28	0.98	11.30	12.55	3.41E-02
CSN3	26.47	8.41	18.06	3.15	3.42E-02
RUSF1	8.09	2.12	5.97	3.82	3.43E-02
AKR1C1	9.93	3.59	6.35	2.77	3.44E-02
LOC105373182	6.30	1.61	4.70	3.93	3.45E-02
FOXF1	6.08	1.44	4.64	4.23	3.45E-02
GDI1	1.38	6.39	-5.01	0.22	3.46E-02
TRMT1L	18.20	6.87	11.33	2.65	3.46E-02

PRR20G	4.87	0.25	4.63	19.83	3.46E-02
EEA1	15.61	8.26	7.35	1.89	3.47E-02
MGME1	3.90	8.64	-4.74	0.45	3.47E-02
MIR591	44.84	1.91	42.93	23.49	3.47E-02
POP7	12.95	1.79	11.16	7.25	3.48E-02
IER3IP1	4.32	9.00	-4.68	0.48	3.48E-02
LINC02354	10.80	2.65	8.15	4.07	3.48E-02
LOC105376850	5.70	2.36	3.34	2.42	3.48E-02
LOC105373386	1.12	0.36	0.76	3.08	3.48E-02
LOC107985279	1.02	0.00	1.02	1.02	3.49E-02
LOC105376341	3.59	1.82	1.76	1.97	3.49E-02
LOC107984659	11.42	3.13	8.29	3.65	3.49E-02
LOC107986088	12.70	5.60	7.10	2.27	3.49E-02
IL6.AS1	6.70	1.01	5.69	6.66	3.50E-02
FAM53C	4.21	1.80	2.41	2.34	3.50E-02
NDP	3.18	10.31	-7.13	0.31	3.50E-02
ZC2HC1A	5.21	9.41	-4.20	0.55	3.51E-02
ERCC6L	8.99	4.47	4.52	2.01	3.51E-02
ASB15	8.72	14.65	-5.93	0.60	3.51E-02
LOC105372439	5.69	0.22	5.48	26.45	3.51E-02
LOC112267951	2.37	0.67	1.70	3.55	3.51E-02
ZBTB41	9.82	6.38	3.44	1.54	3.51E-02
LOC107984585	16.65	5.52	11.13	3.02	3.52E-02
LOC105376448	17.16	2.74	14.42	6.26	3.52E-02
ITGA11	7.68	4.18	3.49	1.84	3.52E-02
KBTBD6	8.52	4.13	4.39	2.06	3.53E-02
SHB	5.90	2.19	3.72	2.70	3.53E-02
ASB16.AS1	4.69	1.77	2.91	2.65	3.53E-02
PHYKPL	10.81	3.89	6.92	2.78	3.54E-02
RNY1	14.37	0.00	14.37	14.37	3.54E-02
RGL3	6.64	3.08	3.56	2.15	3.54E-02
LOC105377893	34.24	2.45	31.80	13.99	3.55E-02
LOC107985890	9.67	3.71	5.96	2.61	3.55E-02
LOC102723381	12.76	3.67	9.09	3.48	3.56E-02
VHL	16.94	3.14	13.80	5.40	3.56E-02
RNF125	6.64	14.09	-7.45	0.47	3.56E-02
LOC654780	3.20	1.02	2.18	3.13	3.56E-02
RAD23A	3.87	0.10	3.77	38.32	3.56E-02
EDC4	4.30	8.17	-3.88	0.53	3.57E-02
LOC105375431	2.64	9.74	-7.09	0.27	3.57E-02
LOC105374292	11.66	5.89	5.77	1.98	3.57E-02
PSTPIP2	11.36	3.44	7.92	3.30	3.57E-02
PPP6C	19.43	13.39	6.04	1.45	3.58E-02
TUBGCP5_2	0.54	0.00	0.54	0.54	3.58E-02
SEC22A	5.78	12.00	-6.22	0.48	3.58E-02
NBPF22P	3.64	11.97	-8.32	0.30	3.59E-02
MIR4738	16.93	0.00	16.93	16.93	3.59E-02

TMEM9	7.23	2.30	4.93	3.15	3.60E-02
LOC105373893	10.16	4.05	6.11	2.51	3.60E-02
LOC107985135	6.93	17.20	-10.27	0.40	3.60E-02
GYS2	9.50	4.23	5.27	2.24	3.61E-02
SCGB1D2	6.36	0.75	5.61	8.50	3.61E-02
LOC105371261	5.24	39.94	-34.70	0.13	3.61E-02
PSMD14	14.94	28.71	-13.77	0.52	3.61E-02
KRTAP4.8	5.33	0.55	4.79	9.75	3.61E-02
BDNF.AS	10.54	2.77	7.78	3.81	3.62E-02
LOC105371254	0.56	0.00	0.56	0.56	3.62E-02
COASY	6.76	1.08	5.69	6.29	3.62E-02
COMMD9	11.71	5.42	6.28	2.16	3.62E-02
MIR194.1	14.59	0.00	14.59	14.59	3.63E-02
CERS4	1.99	5.06	-3.07	0.39	3.63E-02
LOC107985324	0.49	0.00	0.49	0.49	3.64E-02
KDM4A.AS1	6.49	2.04	4.45	3.18	3.64E-02
MARS2	5.73	1.44	4.29	3.97	3.64E-02
THEGL	10.63	6.11	4.52	1.74	3.64E-02
LOC105373528	11.61	3.83	7.78	3.03	3.65E-02
LDLRAD1	9.79	2.44	7.35	4.02	3.65E-02
PSG8.AS1	10.44	2.74	7.70	3.81	3.65E-02
RBFOX3	5.90	13.77	-7.87	0.43	3.66E-02
PDE7B	10.27	5.55	4.73	1.85	3.67E-02
SYCP3	12.44	19.52	-7.08	0.64	3.68E-02
LINC02450	8.26	1.91	6.35	4.32	3.68E-02
LOC102724776	15.69	6.01	9.68	2.61	3.68E-02
LOC105371276	17.00	3.21	13.79	5.30	3.68E-02
MAS1	7.88	4.03	3.84	1.95	3.69E-02
LOC101929141	2.73	0.22	2.51	12.25	3.69E-02
KCTD10	5.58	17.58	-12.00	0.32	3.69E-02
LOC105372173	10.99	4.87	6.12	2.26	3.69E-02
LOC105372208	5.19	16.81	-11.62	0.31	3.70E-02
TMEM253	8.08	2.25	5.83	3.59	3.70E-02
LOC107984716	17.96	5.15	12.81	3.49	3.71E-02
LOC107985595	8.74	1.99	6.75	4.38	3.71E-02
TMEM39B	11.87	5.33	6.54	2.23	3.72E-02
LOC105376826	9.93	1.36	8.57	7.28	3.72E-02
ADPGK	4.54	7.91	-3.37	0.57	3.72E-02
LOC101928505	13.18	3.80	9.38	3.46	3.72E-02
LRRC17	5.06	12.12	-7.07	0.42	3.72E-02
QTRT2	9.41	6.00	3.41	1.57	3.73E-02
DENND2B	8.40	3.75	4.65	2.24	3.73E-02
MAT2A	11.32	3.86	7.46	2.93	3.73E-02
EHF	8.63	4.09	4.53	2.11	3.73E-02
ZNF286A	7.71	3.62	4.09	2.13	3.73E-02
LOC105370846	8.15	3.62	4.53	2.25	3.73E-02
LINC01405	6.02	0.34	5.68	17.78	3.73E-02

CYTH2	6.48	3.37	3.11	1.92	3.74E-02
TMEM67	14.69	9.34	5.35	1.57	3.74E-02
LINC01650	11.57	4.54	7.03	2.55	3.75E-02
DUSP29	1.96	0.03	1.92	57.76	3.75E-02
LOC107985681	1.89	8.33	-6.44	0.23	3.76E-02
LOC105379002	15.47	2.31	13.15	6.68	3.76E-02
PHLDA1	8.65	5.10	3.55	1.70	3.76E-02
LOC105370052	8.64	4.52	4.12	1.91	3.76E-02
LOC105372785	3.57	0.56	3.01	6.42	3.76E-02
ZBTB8B	8.28	5.41	2.88	1.53	3.77E-02
HOTAIRM1	10.54	3.01	7.52	3.50	3.78E-02
LOC105376587	20.89	2.91	17.98	7.17	3.78E-02
PUDP	6.91	2.13	4.79	3.25	3.78E-02
GOLGA2P6	6.88	1.28	5.60	5.38	3.78E-02
ADAP2	4.15	7.55	-3.40	0.55	3.78E-02
NIPAL2	13.04	4.78	8.26	2.73	3.79E-02
LOC102724738	2.39	0.45	1.94	5.29	3.79E-02
LINC00884	4.74	0.78	3.96	6.10	3.79E-02
AP1M2	3.19	6.93	-3.75	0.46	3.80E-02
LOC112268124	3.61	0.57	3.04	6.33	3.81E-02
GPR158	6.09	9.28	-3.19	0.66	3.82E-02
ZNF19	7.70	3.15	4.55	2.45	3.82E-02
ADGRA3	11.82	7.30	4.52	1.62	3.82E-02
LOC102724340	12.48	3.15	9.33	3.96	3.82E-02
LOC105374768	10.34	2.61	7.73	3.97	3.83E-02
LOC105375167	8.76	17.21	-8.45	0.51	3.84E-02
SEMA6A.AS2	5.51	1.43	4.08	3.86	3.84E-02
RBM39	12.44	7.79	4.65	1.60	3.84E-02
TRMT11	14.04	5.00	9.04	2.81	3.85E-02
LOC105377400	8.14	2.68	5.46	3.04	3.85E-02
RNF43	8.76	3.15	5.61	2.78	3.86E-02
FGD4	6.97	10.67	-3.71	0.65	3.87E-02
GDF15	5.70	2.12	3.57	2.68	3.87E-02
ANKRD13B	3.76	1.73	2.03	2.17	3.87E-02
LOC105375861	10.87	4.48	6.39	2.43	3.87E-02
GSAP	10.29	22.60	-12.31	0.46	3.87E-02
LOC105373225	10.78	4.67	6.10	2.31	3.87E-02
PIK3C2G	12.24	7.69	4.55	1.59	3.87E-02
STX12	14.53	6.55	7.97	2.22	3.88E-02
SUSD4	6.75	10.54	-3.79	0.64	3.88E-02
LOC105375107	6.65	1.75	4.90	3.80	3.88E-02
ACSM4	4.89	1.57	3.32	3.11	3.88E-02
CTAGE10P	12.43	4.41	8.02	2.82	3.88E-02
PRDM10	12.54	5.05	7.49	2.48	3.89E-02
USH2A.AS1	27.65	7.84	19.82	3.53	3.89E-02
CNKSR3	8.67	5.21	3.46	1.66	3.89E-02
LOC105379464	1.54	0.00	1.54	1.54	3.90E-02

LOC101929976	16.29	3.38	12.91	4.82	3.90E-02
MHENCR	7.36	0.55	6.81	13.29	3.90E-02
MR1	6.06	9.63	-3.57	0.63	3.91E-02
LOC100130701	13.74	4.82	8.92	2.85	3.91E-02
EEF2K	0.15	0.00	0.15	0.15	3.92E-02
CDH15	4.49	1.32	3.17	3.40	3.92E-02
LOC105370033	5.52	1.66	3.86	3.33	3.92E-02
LOC101929388	22.99	2.77	20.22	8.30	3.92E-02
GUK1	11.75	3.16	8.59	3.72	3.92E-02
LOC105372383	5.12	2.49	2.62	2.05	3.93E-02
FABP1	10.66	28.60	-17.94	0.37	3.93E-02
LINC00963	4.79	1.48	3.31	3.23	3.93E-02
TRPS1	11.20	4.73	6.47	2.37	3.93E-02
LOC105378890	4.53	10.04	-5.52	0.45	3.93E-02
TYK2	10.53	4.00	6.53	2.63	3.93E-02
LOC107985635	1.80	9.16	-7.36	0.20	3.94E-02
MIR9985	8.59	0.00	8.59	8.59	3.94E-02
TOMM22	2.98	1.25	1.73	2.38	3.95E-02
LINC01807	17.36	5.94	11.42	2.92	3.95E-02
LOC105378849	7.40	1.15	6.25	6.42	3.95E-02
SAMSN1.AS1	13.09	2.26	10.83	5.78	3.96E-02
TRAV10	16.50	1.12	15.37	14.72	3.97E-02
LOC107987158	2.94	8.90	-5.95	0.33	3.98E-02
NICN1	10.67	1.23	9.44	8.70	3.98E-02
LHX9	11.59	5.39	6.20	2.15	3.98E-02
GGA2	9.10	5.46	3.64	1.67	3.98E-02
LOC105373311	2.42	0.52	1.90	4.63	3.99E-02
LOC112268140	15.39	1.70	13.69	9.07	3.99E-02
LOC105378426	6.61	14.20	-7.59	0.47	3.99E-02
TMC2	5.64	16.74	-11.09	0.34	4.00E-02
GSTM5	13.82	4.87	8.95	2.84	4.00E-02
SPRR3	9.63	5.32	4.30	1.81	4.00E-02
HGH1	1.10	4.11	-3.01	0.27	4.00E-02
LOC107984525	7.94	11.22	-3.28	0.71	4.00E-02
FAM8A1	7.26	4.26	3.00	1.70	4.00E-02
STAG3L4	12.54	2.97	9.57	4.22	4.00E-02
UAP1	18.57	5.30	13.27	3.50	4.00E-02
LOC105371240	11.52	6.29	5.23	1.83	4.00E-02
TRMT2A	3.82	1.18	2.65	3.25	4.03E-02
LOC105370902	23.25	5.69	17.56	4.09	4.03E-02
MTHFD1L	9.84	5.81	4.03	1.69	4.04E-02
FLT3	15.66	8.56	7.10	1.83	4.05E-02
CSRP1	7.37	2.78	4.59	2.65	4.06E-02
ANKRD30B	16.00	25.51	-9.50	0.63	4.06E-02
LOC102723846	13.45	3.85	9.60	3.49	4.06E-02
CNTN2	5.86	3.30	2.56	1.77	4.07E-02
C5orf58	15.58	5.11	10.47	3.05	4.07E-02

PTMA	9.20	2.19	7.01	4.20	4.07E-02
TRGV3	12.91	3.23	9.68	4.00	4.08E-02
SERPINB5	12.64	2.01	10.63	6.28	4.08E-02
BAG2	5.93	2.78	3.15	2.13	4.08E-02
POLB	13.25	5.35	7.90	2.48	4.08E-02
TAB2	13.76	6.46	7.29	2.13	4.09E-02
WLS	14.00	5.64	8.36	2.48	4.09E-02
TNPO2	8.31	3.52	4.79	2.36	4.09E-02
LOC105370911	10.12	2.00	8.12	5.05	4.10E-02
LOC100270746	10.20	0.41	9.79	24.97	4.11E-02
DNLZ	1.00	6.62	-5.62	0.15	4.11E-02
LOC105372878	13.03	0.52	12.51	24.86	4.12E-02
PRAMEF9	0.52	0.00	0.52	0.52	4.13E-02
INKA2.AS1	4.01	0.00	4.01	4.01	4.13E-02
AGMO	14.17	6.71	7.46	2.11	4.14E-02
RPL19	12.95	3.49	9.46	3.71	4.14E-02
LOC107984778	3.18	14.96	-11.79	0.21	4.14E-02
LINC01543	2.68	26.77	-24.09	0.10	4.14E-02
LOC105378858	7.85	2.72	5.13	2.89	4.15E-02
MELK	15.10	10.43	4.67	1.45	4.15E-02
SDHC	9.17	5.92	3.25	1.55	4.15E-02
MRLN	22.39	2.52	19.86	8.87	4.16E-02
CCNA1	7.18	19.25	-12.07	0.37	4.16E-02
SCPEP1	7.20	3.62	3.58	1.99	4.16E-02
IFNAR2	6.35	3.19	3.16	1.99	4.16E-02
LOC102723529	7.00	16.41	-9.41	0.43	4.17E-02
LOC101927727	5.73	14.66	-8.93	0.39	4.17E-02
PTX4	10.75	1.58	9.17	6.80	4.17E-02
PFDN2	13.33	3.48	9.85	3.83	4.17E-02
LOC105373467	7.70	0.74	6.96	10.38	4.18E-02
EVADR	3.61	0.29	3.32	12.64	4.18E-02
LOC107985173	5.56	1.69	3.87	3.29	4.19E-02
LOC105374654	19.60	1.74	17.86	11.26	4.19E-02
LOC105370861	7.40	0.22	7.18	32.97	4.19E-02
GOLGA7	7.01	2.01	5.00	3.49	4.19E-02
LOC112268436	7.89	3.89	4.01	2.03	4.19E-02
LOC645177	19.51	7.69	11.82	2.54	4.21E-02
LHX3	3.24	1.80	1.44	1.80	4.21E-02
WDFY3.AS2	8.91	3.86	5.05	2.31	4.21E-02
LOC105372922	27.24	4.34	22.90	6.28	4.21E-02
TFAP2B	11.27	6.37	4.90	1.77	4.22E-02
GPATCH3	11.57	2.03	9.54	5.69	4.22E-02
FOXC2	8.90	1.47	7.43	6.05	4.22E-02
MPP3	7.44	2.91	4.53	2.56	4.22E-02
SCOC.AS1	24.88	6.97	17.91	3.57	4.23E-02
LOC107986094	10.97	2.94	8.03	3.73	4.23E-02
SEPTIN3	3.31	1.75	1.55	1.88	4.23E-02

RCAN1	3.85	9.82	-5.97	0.39	4.24E-02
HGC6.3	5.27	0.83	4.44	6.31	4.24E-02
LOC105375553	5.26	15.13	-9.86	0.35	4.25E-02
LOC102723763	4.53	9.97	-5.45	0.45	4.25E-02
LOC105374123	15.19	33.90	-18.71	0.45	4.25E-02
LINC02002	22.67	8.09	14.58	2.80	4.25E-02
IFI44L	10.80	6.30	4.50	1.71	4.27E-02
LOC105378095	12.07	4.29	7.77	2.81	4.27E-02
LMBR1	7.53	4.68	2.85	1.61	4.27E-02
SEMA6C	10.78	3.20	7.58	3.37	4.28E-02
CGB8	10.29	2.75	7.55	3.75	4.28E-02
LOC105378605	7.21	3.42	3.79	2.11	4.28E-02
GRIK1.AS1	7.44	15.72	-8.28	0.47	4.29E-02
NUDT18	3.08	0.70	2.38	4.39	4.29E-02
LOC105371983	11.34	0.62	10.72	18.18	4.29E-02
SLC5A9	6.90	3.26	3.64	2.11	4.29E-02
KRTAP4.3	7.73	2.35	5.38	3.29	4.30E-02
SPATA45	26.64	1.67	24.97	15.97	4.30E-02
ABCC13	13.39	5.68	7.71	2.36	4.30E-02
LOC105372172	20.19	5.48	14.71	3.69	4.30E-02
LOC105370282	20.23	4.81	15.42	4.21	4.31E-02
LINC02200	10.89	2.08	8.81	5.23	4.31E-02
LINC01069	12.94	4.53	8.40	2.85	4.32E-02
GLUD1P2	4.68	17.94	-13.26	0.26	4.32E-02
LOC105376137	6.91	2.36	4.55	2.93	4.32E-02
FUT2	8.55	3.66	4.89	2.34	4.33E-02
LOC105376127	34.45	3.69	30.76	9.35	4.33E-02
LOC100507516	6.24	3.20	3.04	1.95	4.33E-02
LOC105373691	25.46	1.05	24.41	24.22	4.33E-02
OR8B3	11.72	6.16	5.56	1.90	4.33E-02
LOC105369565	2.61	15.27	-12.66	0.17	4.33E-02
SNORD136	13.67	6.56	7.11	2.08	4.34E-02
LOC107986968	32.32	10.44	21.88	3.10	4.34E-02
PIK3R1	6.50	10.31	-3.81	0.63	4.35E-02
OAZ1	4.18	1.71	2.47	2.45	4.35E-02
LOC105379283	8.90	0.94	7.96	9.50	4.35E-02
ZPLD1	16.03	8.24	7.79	1.95	4.36E-02
SNORD115.5	15.61	1.83	13.78	8.53	4.36E-02
LINC02313	4.18	0.00	4.18	4.18	4.36E-02
PATE1	16.76	4.78	11.98	3.51	4.37E-02
GKAP1	24.01	9.65	14.36	2.49	4.37E-02
LOC107985792	4.12	13.28	-9.15	0.31	4.37E-02
LOC105373282	2.64	15.31	-12.67	0.17	4.38E-02
LOC102723331	10.47	6.05	4.42	1.73	4.39E-02
IL15	13.72	4.54	9.18	3.02	4.40E-02
LOC105376589	11.49	3.69	7.80	3.11	4.41E-02
RGS9BP	4.88	0.80	4.08	6.07	4.41E-02

CHI3L1	4.02	11.97	-7.95	0.34	4.42E-02
LOC105376257	5.91	10.25	-4.34	0.58	4.43E-02
GTF2F2	11.40	4.60	6.81	2.48	4.43E-02
WTAP	6.09	11.72	-5.63	0.52	4.43E-02
FNBP1	6.94	13.13	-6.20	0.53	4.43E-02
LOC105375323	15.85	4.42	11.43	3.58	4.43E-02
ARHGEF3.AS1	12.65	1.49	11.16	8.50	4.43E-02
EML5	5.07	7.69	-2.63	0.66	4.44E-02
CDS2	6.55	3.15	3.41	2.08	4.44E-02
LINC02692	6.63	0.00	6.63	6.63	4.44E-02
PNMA2	8.00	2.08	5.91	3.84	4.44E-02
CYP1B1	6.35	1.54	4.82	4.13	4.45E-02
LOC112267958	2.22	0.58	1.64	3.85	4.45E-02
ADCY10	5.82	10.93	-5.11	0.53	4.45E-02
LINC02064	18.36	2.23	16.13	8.24	4.46E-02
KRTAP5.10	8.02	2.19	5.83	3.67	4.46E-02
LOC107987107	2.42	26.98	-24.56	0.09	4.47E-02
COL9A3	13.05	4.58	8.47	2.85	4.47E-02
LINC01883	12.34	2.87	9.47	4.29	4.47E-02
ADRM1	2.46	7.84	-5.38	0.31	4.48E-02
LOC105371272	3.22	0.38	2.84	8.56	4.49E-02
LOC101928266	6.67	3.33	3.34	2.01	4.49E-02
SNORA58B	11.15	0.00	11.15	11.15	4.49E-02
LOC112268078	14.70	0.73	13.97	20.25	4.49E-02
LOC105372813	8.79	0.41	8.37	21.30	4.49E-02
WDR5	2.97	13.67	-10.71	0.22	4.49E-02
HAGLR	5.49	1.99	3.51	2.77	4.49E-02
OR5H14	14.18	8.20	5.99	1.73	4.50E-02
C19orf44	8.01	4.45	3.55	1.80	4.50E-02
LOC112268221	6.34	2.66	3.67	2.38	4.51E-02
TOP2A	7.00	14.94	-7.94	0.47	4.51E-02
NEIL2_1	1.09	0.00	1.09	1.09	4.51E-02
GID8	7.61	2.98	4.63	2.55	4.51E-02
MRC2	7.04	4.16	2.88	1.69	4.52E-02
KCND3.IT1	11.75	1.53	10.22	7.67	4.52E-02
MEIS3P1	4.79	19.04	-14.26	0.25	4.52E-02
LCE5A	9.71	0.45	9.26	21.73	4.53E-02
KLHDC10	6.26	12.78	-6.52	0.49	4.53E-02
CCDC141	7.43	9.28	-1.85	0.80	4.54E-02
LOC107986896	15.92	2.73	13.18	5.82	4.54E-02
LOC107984872	3.68	0.98	2.70	3.77	4.54E-02
SNORD18B	12.72	6.86	5.86	1.85	4.54E-02
LINC01341	4.17	0.34	3.82	12.17	4.54E-02
TPST1	7.07	3.22	3.85	2.19	4.55E-02
ACTR1B	4.27	0.76	3.51	5.62	4.55E-02
LOC105370604	8.19	22.61	-14.42	0.36	4.56E-02
PDE4B	20.23	8.43	11.80	2.40	4.57E-02

ZBTB42	4.22	1.51	2.71	2.79	4.57E-02
LOC112267966	15.61	4.78	10.84	3.27	4.57E-02
LOC105374846	10.49	3.05	7.43	3.43	4.57E-02
FBXL14	4.70	8.58	-3.88	0.55	4.58E-02
LOC105372432	1.17	0.00	1.17	1.17	4.58E-02
LOC105377659	11.09	1.70	9.39	6.51	4.59E-02
LOC105369457	9.31	0.56	8.75	16.69	4.59E-02
LINC02742	10.84	4.62	6.22	2.35	4.59E-02
LOC107986930	15.00	5.76	9.24	2.60	4.59E-02
LOC105376568	8.58	2.20	6.38	3.90	4.60E-02
CCM2	6.23	2.66	3.57	2.34	4.60E-02
PKMYT1	2.77	0.48	2.29	5.74	4.61E-02
LOC101929322	13.42	7.65	5.77	1.75	4.61E-02
LINC01358	6.03	2.24	3.79	2.69	4.61E-02
BCAR3	12.72	2.71	10.01	4.70	4.61E-02
OR10AD1	9.88	3.36	6.52	2.94	4.61E-02
MIR6127	11.98	0.00	11.98	11.98	4.62E-02
ATP13A2	5.75	2.11	3.64	2.72	4.62E-02
MGAT5	11.69	5.87	5.82	1.99	4.63E-02
LOC105371010	5.58	2.31	3.28	2.42	4.63E-02
CAGE1	9.08	2.90	6.18	3.13	4.63E-02
CTDSPL2	18.24	5.60	12.63	3.25	4.64E-02
POU4F2	8.87	2.44	6.43	3.64	4.64E-02
LAMC2	11.22	3.98	7.24	2.82	4.64E-02
LUADT1	28.30	4.31	23.99	6.57	4.64E-02
TRV.AAC1.3	82.32	5.02	77.29	16.39	4.65E-02
XKR6	1.22	0.28	0.94	4.41	4.65E-02
CARD9	4.12	1.64	2.48	2.52	4.66E-02
PSPC1	10.12	4.17	5.94	2.42	4.66E-02
KLF9	10.36	3.30	7.06	3.14	4.67E-02
LOC105370030	4.92	12.48	-7.56	0.39	4.67E-02
LOC107985217	4.36	12.19	-7.83	0.36	4.67E-02
LOC105378127	23.57	3.47	20.10	6.79	4.67E-02
LOC107987037	16.02	2.59	13.43	6.19	4.67E-02
LOC105379535	0.44	0.00	0.44	0.44	4.68E-02
WFS1	4.12	16.00	-11.88	0.26	4.68E-02
TRBV7.5_1	3.49	0.87	2.62	4.02	4.68E-02
LOC105378963	13.46	6.15	7.31	2.19	4.68E-02
LOC105369421	5.96	2.99	2.96	1.99	4.70E-02
LOC105372411	11.59	2.36	9.23	4.91	4.70E-02
MAGEH1	7.56	0.84	6.72	8.99	4.71E-02
LOC101927374	13.41	3.38	10.03	3.97	4.71E-02
FLJ20712	8.30	1.01	7.29	8.23	4.71E-02
XRCC1	10.44	4.19	6.25	2.49	4.71E-02
LOC105378269	4.94	0.00	4.94	4.94	4.71E-02
ZNRF3	7.77	3.95	3.82	1.97	4.71E-02
KRTAP13.1	9.78	3.42	6.36	2.86	4.71E-02

SNORA2B	24.35	1.10	23.25	22.23	4.72E-02
EPC1	10.38	7.28	3.10	1.43	4.72E-02
LINC02050	15.92	2.90	13.02	5.49	4.73E-02
CERK	6.46	2.53	3.93	2.55	4.73E-02
LOC105369515	3.17	10.26	-7.10	0.31	4.74E-02
ZC3H13	12.53	8.40	4.13	1.49	4.74E-02
NR1I2	5.07	8.75	-3.68	0.58	4.75E-02
KDM7A.DT	9.23	2.19	7.04	4.22	4.76E-02
ITGB4	3.92	10.16	-6.24	0.39	4.77E-02
AURKA	12.54	6.71	5.83	1.87	4.77E-02
LOC107987087	24.16	3.39	20.77	7.13	4.77E-02
CH507.9B2.8	0.31	0.00	0.31	0.31	4.77E-02
SOAT2	13.05	4.43	8.62	2.95	4.78E-02
MRPL45_1	1.10	0.12	0.98	8.92	4.78E-02
IFI35	19.34	3.52	15.82	5.50	4.79E-02
LOC105378766	6.20	24.40	-18.20	0.25	4.79E-02
RYR3.DT	16.43	2.23	14.20	7.37	4.80E-02
DGKB	11.56	7.10	4.47	1.63	4.80E-02
AKR1C8P	6.37	2.61	3.76	2.44	4.80E-02
IFNW1	7.29	0.76	6.54	9.64	4.80E-02
PRSS58_1	2.59	0.00	2.59	2.59	4.80E-02
LOC100505774	9.99	3.42	6.56	2.92	4.81E-02
APOBEC1	8.30	2.19	6.12	3.80	4.81E-02
TDP1	14.09	7.91	6.17	1.78	4.81E-02
LOC105371132	0.68	0.09	0.59	7.86	4.81E-02
LOC101927073	11.94	5.78	6.17	2.07	4.81E-02
LOC105377994	20.86	9.58	11.28	2.18	4.82E-02
PCGF3	9.24	4.31	4.93	2.14	4.82E-02
SPRY4	2.87	6.48	-3.61	0.44	4.82E-02
ANKRD26	9.15	6.69	2.46	1.37	4.83E-02
LOC107984284	6.17	16.02	-9.85	0.38	4.83E-02
D2HGDH	6.00	2.31	3.68	2.59	4.83E-02
LOC105371608	1.89	11.25	-9.36	0.17	4.84E-02
FAM83A	5.74	4.05	1.70	1.42	4.84E-02
ANKRD34C.AS1	4.05	21.77	-17.72	0.19	4.84E-02
LOC105375955	7.00	0.35	6.65	20.17	4.85E-02
LOC105371679	11.42	3.70	7.73	3.09	4.85E-02
LINC01771	11.24	0.00	11.24	11.24	4.86E-02
LOC105370262	10.75	3.50	7.25	3.07	4.87E-02
FCGR2C	18.63	6.32	12.31	2.95	4.88E-02
MRM1	0.75	0.05	0.70	15.88	4.88E-02
TRIM68	2.64	6.41	-3.76	0.41	4.88E-02
LOC107984822	21.67	4.48	17.19	4.84	4.89E-02
NMD3	14.58	6.44	8.14	2.26	4.89E-02
LINC02086	12.49	5.86	6.63	2.13	4.89E-02
RBM47	8.49	4.62	3.88	1.84	4.89E-02
MYOM3.AS1	6.12	1.27	4.84	4.80	4.89E-02

CEP68	6.29	3.41	2.88	1.84	4.90E-02
BARX1.DT	4.90	0.77	4.14	6.39	4.90E-02
SH2D7	3.09	7.68	-4.59	0.40	4.90E-02
PSLNR	8.50	26.99	-18.49	0.32	4.90E-02
LOC107984316	3.94	8.43	-4.49	0.47	4.91E-02
LOC105374362	5.17	9.75	-4.58	0.53	4.92E-02
LOC105375684	2.57	21.31	-18.73	0.12	4.92E-02
MIR6859.1	5.79	0.00	5.79	5.79	4.92E-02
VN1R1	8.69	2.36	6.33	3.68	4.92E-02
LOC105379513	1.25	0.30	0.95	4.11	4.93E-02
LOC107986970	7.20	4.21	2.99	1.71	4.93E-02
LOC107986334	7.19	3.58	3.62	2.01	4.93E-02
PRRC2C	13.08	7.73	5.34	1.69	4.93E-02
LOC105376991	8.30	1.57	6.73	5.28	4.94E-02
LOC107986440	13.42	2.86	10.56	4.69	4.94E-02
ENOSF1	6.15	3.26	2.89	1.89	4.94E-02
AKAP1	3.75	7.19	-3.44	0.52	4.95E-02
VWA5B1	7.95	4.48	3.47	1.77	4.96E-02
DPEP2	13.61	6.44	7.17	2.11	4.96E-02
LOC101929560	3.29	18.85	-15.56	0.17	4.96E-02
LOC105377088	6.79	0.97	5.82	7.00	4.97E-02
OR10Z1	9.01	3.46	5.54	2.60	4.97E-02
LRFN4	2.65	6.28	-3.63	0.42	4.97E-02
GCC2.AS1	2.09	0.22	1.87	9.61	4.97E-02
FITM2	9.01	1.76	7.25	5.12	4.98E-02
SEC61A2	16.85	6.22	10.63	2.71	4.98E-02
C16orf74	8.57	0.46	8.10	18.55	4.98E-02
ENTPD2	5.39	1.70	3.69	3.17	4.98E-02
LOC105374136	17.73	2.69	15.04	6.59	4.98E-02
MAP3K9	7.44	3.08	4.35	2.41	4.99E-02
SLC25A16	6.11	9.82	-3.71	0.62	4.99E-02
SLC22A23	9.84	4.23	5.61	2.33	4.99E-02
LINC01683	9.38	28.11	-18.73	0.33	4.99E-02
LONRF3	5.43	2.80	2.63	1.94	4.99E-02
LOC105378226	10.11	3.72	6.39	2.72	5.00E-02
SLC37A3	16.08	6.96	9.12	2.31	5.00E-02
EPM2A	10.36	5.85	4.50	1.77	5.00E-02
