# Supplementary material for: RNA Sequencing of Intestinal Enterocytes Pre- and Post-Roux-en-Y Gastric Bypass Reveals Alteration in Gene Expression Related to Enterocyte Differentiation, Restitution, and Obesity with Regulation by Schlafen 12
Source: Cells. 2022 Oct 18;11(20):3283. doi: 10.3390/cells11203283 (PMC9601224; doi:10.3390/cells11203283)
Supplement: Supplementary file 1 [file cells-11-03283-s001.zip › Table S2- Post_BMI_Linear.pdf]

	r.squared	adj.r.squared	sigma	statistic	p.value
LOC107983998	7.81E-01	7.56E-01	1.77	32.07	3.09E-04
LOC105378353	7.78E-01	7.54E-01	1.78	31.57	3.26E-04
LINC00160	7.59E-01	7.32E-01	1.86	28.27	4.83E-04
MS4A8	7.52E-01	7.24E-01	1.88	27.29	5.46E-04
ZNF239	7.49E-01	7.21E-01	1.90	26.80	5.82E-04
ADNP.AS1	7.48E-01	7.20E-01	1.90	26.67	5.92E-04
PISRT1	7.36E-01	7.06E-01	1.94	25.07	7.32E-04
LOC105374652	7.33E-01	7.03E-01	1.95	24.71	7.69E-04
RPL10A	7.33E-01	7.03E-01	1.96	24.65	7.75E-04
TBC1D29P	7.26E-01	6.95E-01	1.98	23.81	8.72E-04
SIRPB3P	7.18E-01	6.86E-01	2.01	22.88	9.96E-04
LINC00302	7.17E-01	6.85E-01	2.01	22.76	1.01E-03
TRAV36DV7	7.16E-01	6.84E-01	2.02	22.66	1.03E-03
LINC00605	7.14E-01	6.82E-01	2.02	22.46	1.06E-03
MCEMP1	7.11E-01	6.79E-01	2.03	22.18	1.11E-03
LOC105377416	7.08E-01	6.75E-01	2.05	21.79	1.17E-03
LOC105370917	7.07E-01	6.74E-01	2.05	21.68	1.19E-03
C17orf75	7.04E-01	6.71E-01	2.06	21.43	1.24E-03
HLA.F.AS1_1	7.04E-01	6.71E-01	2.06	21.39	1.25E-03
TMEM171	7.02E-01	6.69E-01	2.06	21.24	1.27E-03
ANXA2P1	7.00E-01	6.67E-01	2.07	21.05	1.31E-03
PSMD4	6.99E-01	6.66E-01	2.07	20.92	1.34E-03
LOC105379350	6.99E-01	6.65E-01	2.08	20.89	1.35E-03
MIR4303	6.98E-01	6.64E-01	2.08	20.76	1.37E-03
SPRN	6.97E-01	6.64E-01	2.08	20.72	1.38E-03
THEG	6.96E-01	6.62E-01	2.08	20.63	1.40E-03
THUMPD3.AS1	6.95E-01	6.62E-01	2.09	20.55	1.42E-03
ZSCAN10	6.95E-01	6.61E-01	2.09	20.47	1.44E-03
MTRES1	6.94E-01	6.60E-01	2.09	20.44	1.44E-03
DCLK1	6.93E-01	6.59E-01	2.10	20.32	1.47E-03
LOC107987086	6.92E-01	6.57E-01	2.10	20.19	1.50E-03
MIR1263	6.89E-01	6.55E-01	2.11	19.94	1.56E-03
LINC02237	6.88E-01	6.54E-01	2.11	19.88	1.58E-03
TPI1P3	6.88E-01	6.53E-01	2.11	19.82	1.60E-03
DPM3	6.86E-01	6.51E-01	2.12	19.67	1.64E-03
CTAG2	6.85E-01	6.50E-01	2.12	19.61	1.65E-03
CDKN2A.DT	6.85E-01	6.50E-01	2.12	19.54	1.67E-03
LOC105371729	6.84E-01	6.49E-01	2.13	19.48	1.69E-03
TYMP	6.78E-01	6.43E-01	2.15	18.98	1.83E-03
ICOSLG	6.78E-01	6.42E-01	2.15	18.95	1.84E-03

LINC02311	6.77E-01	6.41E-01	2.15	18.86	1.87E-03
LINC01050	6.75E-01	6.39E-01	2.15	18.73	1.91E-03
GJA9.MYCBP	6.75E-01	6.39E-01	2.16	18.73	1.91E-03
LOC100421746	6.75E-01	6.38E-01	2.16	18.65	1.94E-03
C9orf135.DT	6.74E-01	6.38E-01	2.16	18.63	1.94E-03
LOC105375318	6.74E-01	6.38E-01	2.16	18.60	1.95E-03
AGR3	6.74E-01	6.37E-01	2.16	18.57	1.96E-03
TRT.TGT4.1	6.73E-01	6.37E-01	2.16	18.53	1.98E-03
MIR1294	6.73E-01	6.37E-01	2.16	18.53	1.98E-03
NME1	6.73E-01	6.36E-01	2.16	18.49	1.99E-03
LOC105375072	6.72E-01	6.35E-01	2.17	18.40	2.02E-03
LOC105376184	6.70E-01	6.33E-01	2.17	18.28	2.06E-03
LOC105370450	6.69E-01	6.32E-01	2.18	18.16	2.11E-03
LINC01243	6.68E-01	6.31E-01	2.18	18.12	2.12E-03
LINC00373	6.66E-01	6.28E-01	2.19	17.91	2.20E-03
SCHIP1	6.65E-01	6.28E-01	2.19	17.86	2.22E-03
LINC02692	6.64E-01	6.27E-01	2.19	17.82	2.24E-03
LOC105374284	6.64E-01	6.27E-01	2.19	17.79	2.25E-03
H2BC6	6.64E-01	6.26E-01	2.19	17.76	2.26E-03
OR56B1	6.63E-01	6.25E-01	2.20	17.69	2.29E-03
LINC02685	6.62E-01	6.24E-01	2.20	17.63	2.31E-03
LINC01900	6.60E-01	6.23E-01	2.20	17.51	2.36E-03
SEZ6L.AS1	6.59E-01	6.22E-01	2.21	17.42	2.40E-03
TRDV2	6.59E-01	6.21E-01	2.21	17.40	2.41E-03
LOC105373411	6.59E-01	6.21E-01	2.21	17.39	2.41E-03
POU3F2	6.59E-01	6.21E-01	2.21	17.36	2.42E-03
LOC105379007	6.58E-01	6.20E-01	2.21	17.28	2.46E-03
LOC105377894	6.57E-01	6.19E-01	2.21	17.26	2.47E-03
H2BC11	6.57E-01	6.19E-01	2.22	17.24	2.48E-03
SNORD18B	6.56E-01	6.18E-01	2.22	17.15	2.52E-03
LOC107987285	6.55E-01	6.17E-01	2.22	17.09	2.54E-03
LOC105370643	6.54E-01	6.15E-01	2.23	17.00	2.59E-03
LOC105371062	6.50E-01	6.11E-01	2.24	16.70	2.73E-03
LOC105374733	6.49E-01	6.10E-01	2.24	16.67	2.74E-03
LINC02176	6.48E-01	6.09E-01	2.24	16.60	2.78E-03
UPK3A	6.48E-01	6.09E-01	2.25	16.54	2.81E-03
LOC112268110	6.47E-01	6.08E-01	2.25	16.50	2.83E-03
LOC101927756	6.46E-01	6.07E-01	2.25	16.45	2.86E-03
SERPIND1	6.46E-01	6.07E-01	2.25	16.45	2.86E-03
FAM98A	6.45E-01	6.06E-01	2.25	16.37	2.90E-03
LOC105370255	6.45E-01	6.06E-01	2.25	16.36	2.91E-03

OR5K1	6.44E-01	6.05E-01	2.26	16.31	2.93E-03
MIR1912	6.43E-01	6.03E-01	2.26	16.20	3.00E-03
TAS2R7	6.43E-01	6.03E-01	2.26	16.20	3.00E-03
LOC112268014	6.43E-01	6.03E-01	2.26	16.20	3.00E-03
NXNL1	6.43E-01	6.03E-01	2.26	16.19	3.00E-03
LOC105379444	6.41E-01	6.01E-01	2.27	16.04	3.09E-03
SLC47A2	6.40E-01	6.00E-01	2.27	16.03	3.09E-03
LOC105377183	6.40E-01	6.00E-01	2.27	16.03	3.10E-03
VAT1	6.39E-01	5.99E-01	2.27	15.95	3.14E-03
ERG28	6.39E-01	5.99E-01	2.27	15.95	3.14E-03
LOC107986716	6.39E-01	5.98E-01	2.27	15.90	3.17E-03
ZNF451.AS1	6.38E-01	5.98E-01	2.28	15.85	3.20E-03
GLDC	6.38E-01	5.97E-01	2.28	15.84	3.21E-03
MED11	6.37E-01	5.97E-01	2.28	15.79	3.23E-03
VIP	6.37E-01	5.96E-01	2.28	15.78	3.24E-03
LOC100128398	6.37E-01	5.96E-01	2.28	15.77	3.25E-03
FOXD3	6.36E-01	5.96E-01	2.28	15.74	3.27E-03
FEZF2	6.36E-01	5.95E-01	2.28	15.71	3.28E-03
MAZ	6.34E-01	5.94E-01	2.29	15.62	3.34E-03
ERP29	6.33E-01	5.93E-01	2.29	15.55	3.39E-03
TRAV9.1	6.33E-01	5.93E-01	2.29	15.54	3.39E-03
C4orf47	6.33E-01	5.92E-01	2.29	15.53	3.40E-03
LRRC37A4P_1	6.32E-01	5.92E-01	2.29	15.48	3.43E-03
CLC	6.32E-01	5.91E-01	2.29	15.46	3.45E-03
LOC105378030	6.32E-01	5.91E-01	2.29	15.45	3.45E-03
HOXC.AS3	6.32E-01	5.91E-01	2.29	15.45	3.45E-03
LMO7DN.IT1	6.31E-01	5.90E-01	2.30	15.42	3.48E-03
WHAMMP1	6.31E-01	5.90E-01	2.30	15.40	3.49E-03
SLC6A3_1	6.31E-01	5.90E-01	2.30	15.40	3.49E-03
LOC388436	6.30E-01	5.88E-01	2.30	15.30	3.56E-03
SYCE1L	6.29E-01	5.88E-01	2.30	15.28	3.57E-03
LOC107985518	6.29E-01	5.88E-01	2.30	15.28	3.57E-03
TCP11X2	6.29E-01	5.88E-01	2.30	15.27	3.58E-03
LOC112267879	6.29E-01	5.87E-01	2.30	15.24	3.60E-03
LOC105369649	6.29E-01	5.87E-01	2.30	15.24	3.60E-03
LOC107984199	6.27E-01	5.85E-01	2.31	15.10	3.70E-03
MYF6	6.26E-01	5.85E-01	2.31	15.08	3.71E-03
CH507.145C22.1	6.25E-01	5.84E-01	2.32	15.03	3.75E-03
LOC107985159	6.25E-01	5.83E-01	2.32	15.00	3.77E-03
RCAN2	6.25E-01	5.83E-01	2.32	14.99	3.78E-03
MIR1286	6.24E-01	5.82E-01	2.32	14.92	3.83E-03

ARMC2.AS1	6.24E-01	5.82E-01	2.32	14.92	3.83E-03
LOC107985342	6.24E-01	5.82E-01	2.32	14.92	3.83E-03
ANAPC2	6.23E-01	5.81E-01	2.32	14.87	3.87E-03
LOC107985151	6.22E-01	5.80E-01	2.32	14.84	3.90E-03
EVX1.AS	6.22E-01	5.80E-01	2.32	14.83	3.90E-03
MIR10527	6.22E-01	5.80E-01	2.33	14.81	3.92E-03
LOC105379368	6.22E-01	5.80E-01	2.33	14.78	3.94E-03
MORN5	6.21E-01	5.79E-01	2.33	14.76	3.95E-03
LINC02285	6.21E-01	5.79E-01	2.33	14.73	3.98E-03
LOC107985890	6.19E-01	5.77E-01	2.33	14.65	4.04E-03
FAU	6.19E-01	5.77E-01	2.33	14.64	4.05E-03
RAC3	6.19E-01	5.77E-01	2.33	14.63	4.06E-03
PRAMEF7	6.18E-01	5.76E-01	2.34	14.58	4.10E-03
LOC107984931	6.18E-01	5.75E-01	2.34	14.55	4.13E-03
ARL17B	6.18E-01	5.75E-01	2.34	14.54	4.13E-03
TRQ.CTG5.1	6.18E-01	5.75E-01	2.34	14.53	4.14E-03
LOC105372407	6.17E-01	5.75E-01	2.34	14.53	4.14E-03
LOC107985059	6.17E-01	5.74E-01	2.34	14.50	4.17E-03
LOC105378052	6.17E-01	5.74E-01	2.34	14.48	4.18E-03
LOC105378949	6.16E-01	5.73E-01	2.34	14.43	4.23E-03
LOC107986949	6.15E-01	5.72E-01	2.35	14.38	4.27E-03
SNORA74D	6.15E-01	5.72E-01	2.35	14.37	4.28E-03
LOC107985095	6.14E-01	5.71E-01	2.35	14.33	4.31E-03
TSL	6.14E-01	5.71E-01	2.35	14.33	4.31E-03
LOC105370627	6.14E-01	5.71E-01	2.35	14.32	4.32E-03
OXCT2	6.14E-01	5.71E-01	2.35	14.30	4.34E-03
LOC107987187	6.13E-01	5.70E-01	2.35	14.26	4.37E-03
OVCA2	6.13E-01	5.69E-01	2.35	14.23	4.40E-03
A1BG.AS1	6.12E-01	5.69E-01	2.36	14.22	4.41E-03
KIR3DL2_9	6.12E-01	5.69E-01	2.36	14.21	4.42E-03
ADAM18	6.12E-01	5.69E-01	2.36	14.21	4.42E-03
MGC4859	6.12E-01	5.69E-01	2.36	14.20	4.42E-03
MIR1469	6.11E-01	5.68E-01	2.36	14.16	4.47E-03
SEC23A.AS1	6.11E-01	5.68E-01	2.36	14.15	4.48E-03
IGLV3.4	6.10E-01	5.67E-01	2.36	14.10	4.52E-03
LINC01843	6.10E-01	5.67E-01	2.36	14.10	4.52E-03
LOC105375303	6.10E-01	5.66E-01	2.36	14.06	4.55E-03
LOC105378289	6.09E-01	5.66E-01	2.36	14.04	4.57E-03
LINC01976	6.09E-01	5.65E-01	2.37	14.00	4.61E-03
RAB11FIP1	6.08E-01	5.65E-01	2.37	13.99	4.63E-03
LINC01717	6.08E-01	5.65E-01	2.37	13.98	4.63E-03

MPG	6.08E-01	5.65E-01	2.37	13.98	4.63E-03
OSBPL10.AS1	6.08E-01	5.64E-01	2.37	13.96	4.66E-03
IGHV3.33_1	6.08E-01	5.64E-01	2.37	13.94	4.67E-03
LOC101929614	6.07E-01	5.64E-01	2.37	13.92	4.69E-03
FAM13C	6.07E-01	5.63E-01	2.37	13.88	4.73E-03
PARD6G	6.06E-01	5.62E-01	2.37	13.85	4.75E-03
LBHD2	6.06E-01	5.62E-01	2.37	13.85	4.76E-03
LOC105369687	6.06E-01	5.62E-01	2.38	13.82	4.79E-03
TRIM8	6.05E-01	5.61E-01	2.38	13.80	4.80E-03
LOC100505622	6.05E-01	5.61E-01	2.38	13.80	4.81E-03
ANKRD19P	6.05E-01	5.61E-01	2.38	13.79	4.82E-03
LOC105375938	6.04E-01	5.61E-01	2.38	13.75	4.86E-03
LOC114841040	6.03E-01	5.59E-01	2.38	13.70	4.91E-03
MRGPRG	6.03E-01	5.59E-01	2.38	13.69	4.92E-03
KIF2B	6.03E-01	5.59E-01	2.38	13.68	4.93E-03
ZFX.AS1	6.03E-01	5.59E-01	2.38	13.66	4.96E-03
ACP1	6.02E-01	5.57E-01	2.39	13.60	5.02E-03
LINC01100	6.01E-01	5.57E-01	2.39	13.57	5.04E-03
LOC105371716	6.01E-01	5.57E-01	2.39	13.57	5.05E-03
MIR1285.2	6.01E-01	5.56E-01	2.39	13.54	5.07E-03
LOC105369167	6.01E-01	5.56E-01	2.39	13.54	5.08E-03
ZCCHC14	6.01E-01	5.56E-01	2.39	13.53	5.09E-03
IFNA17	6.00E-01	5.56E-01	2.39	13.52	5.10E-03
OSMR.AS1	6.00E-01	5.56E-01	2.39	13.50	5.12E-03
SAMSN1.AS1	6.00E-01	5.56E-01	2.39	13.50	5.12E-03
LOC107984436	5.99E-01	5.55E-01	2.39	13.47	5.15E-03
BBC3	5.99E-01	5.54E-01	2.40	13.45	5.18E-03
LOC100287010	5.99E-01	5.54E-01	2.40	13.44	5.19E-03
LSP1P5	5.99E-01	5.54E-01	2.40	13.42	5.20E-03
LOC105370733	5.98E-01	5.54E-01	2.40	13.41	5.22E-03
TTC9B	5.98E-01	5.53E-01	2.40	13.39	5.24E-03
ELF3	5.98E-01	5.53E-01	2.40	13.37	5.27E-03
GADD45GIP1	5.98E-01	5.53E-01	2.40	13.36	5.27E-03
C19orf81	5.97E-01	5.53E-01	2.40	13.35	5.28E-03
C18orf63	5.97E-01	5.52E-01	2.40	13.32	5.32E-03
LOC105369850	5.96E-01	5.51E-01	2.40	13.28	5.37E-03
LINC02428	5.96E-01	5.51E-01	2.40	13.28	5.37E-03
CFAP157	5.96E-01	5.51E-01	2.40	13.27	5.38E-03
LYRM1	5.95E-01	5.50E-01	2.41	13.23	5.42E-03
LOC102723630	5.95E-01	5.50E-01	2.41	13.23	5.43E-03
SYNGAP1_1	5.95E-01	5.50E-01	2.41	13.23	5.43E-03

LINC02321	5.95E-01	5.50E-01	2.41	13.21	5.45E-03
LOC105372005	5.94E-01	5.49E-01	2.41	13.17	5.49E-03
LOC107984432	5.94E-01	5.49E-01	2.41	13.16	5.50E-03
LOC105374308	5.94E-01	5.49E-01	2.41	13.16	5.51E-03
OTOS	5.93E-01	5.48E-01	2.41	13.12	5.56E-03
LOC105379675	5.93E-01	5.47E-01	2.41	13.09	5.59E-03
LINC01814	5.92E-01	5.47E-01	2.42	13.06	5.63E-03
HOTTIP	5.92E-01	5.46E-01	2.42	13.05	5.64E-03
LOC107986486	5.92E-01	5.46E-01	2.42	13.05	5.64E-03
LOC105371072	5.91E-01	5.46E-01	2.42	13.02	5.68E-03
LINC02545	5.91E-01	5.46E-01	2.42	13.01	5.68E-03
MTFP1	5.91E-01	5.45E-01	2.42	13.00	5.70E-03
LOC105370055	5.90E-01	5.45E-01	2.42	12.98	5.73E-03
CDK17	5.90E-01	5.45E-01	2.42	12.97	5.73E-03
LOC107986606	5.90E-01	5.44E-01	2.42	12.95	5.76E-03
BMERB1_1	5.90E-01	5.44E-01	2.42	12.94	5.77E-03
TSPY8	5.89E-01	5.44E-01	2.42	12.92	5.80E-03
LOC105374498_1	5.89E-01	5.43E-01	2.43	12.88	5.85E-03
MFHAS1_1	5.89E-01	5.43E-01	2.43	12.88	5.85E-03
MYLK.AS1	5.88E-01	5.43E-01	2.43	12.87	5.87E-03
LOC105376828	5.88E-01	5.43E-01	2.43	12.86	5.87E-03
MIR3131	5.88E-01	5.42E-01	2.43	12.85	5.88E-03
LOC101927898	5.88E-01	5.42E-01	2.43	12.85	5.89E-03
TBX2.AS1	5.88E-01	5.42E-01	2.43	12.85	5.89E-03
MIR12124	5.88E-01	5.42E-01	2.43	12.85	5.89E-03
SNORD114.15	5.87E-01	5.41E-01	2.43	12.80	5.95E-03
OR2J1_2	5.87E-01	5.41E-01	2.43	12.79	5.96E-03
LOC105372208	5.87E-01	5.41E-01	2.43	12.78	5.97E-03
LOC105373176	5.87E-01	5.41E-01	2.43	12.77	5.99E-03
USP30.AS1	5.86E-01	5.41E-01	2.43	12.76	6.00E-03
PAQR4	5.86E-01	5.40E-01	2.43	12.73	6.04E-03
NRGN	5.86E-01	5.40E-01	2.44	12.72	6.06E-03
LOC101928069	5.85E-01	5.39E-01	2.44	12.68	6.11E-03
CNPY2	5.85E-01	5.39E-01	2.44	12.68	6.12E-03
SLC43A2	5.85E-01	5.38E-01	2.44	12.66	6.13E-03
RPH3AL_2	5.84E-01	5.38E-01	2.44	12.65	6.15E-03
LOC105377315	5.83E-01	5.37E-01	2.44	12.59	6.24E-03
LOC101928188	5.83E-01	5.36E-01	2.44	12.57	6.26E-03
MIR153.2	5.83E-01	5.36E-01	2.44	12.56	6.27E-03
NREP	5.83E-01	5.36E-01	2.44	12.56	6.28E-03
TRIM49B	5.82E-01	5.36E-01	2.44	12.55	6.29E-03

DBNDD2	5.82E-01	5.35E-01	2.45	12.51	6.35E-03
VTN	5.81E-01	5.34E-01	2.45	12.48	6.38E-03
BAALC.AS1	5.80E-01	5.34E-01	2.45	12.45	6.43E-03
LOC645188	5.80E-01	5.34E-01	2.45	12.45	6.43E-03
NPIPA8	5.80E-01	5.34E-01	2.45	12.45	6.44E-03
LOC105377094	5.80E-01	5.34E-01	2.45	12.44	6.45E-03
LOC105373334	5.80E-01	5.33E-01	2.45	12.44	6.45E-03
OR6N2	5.80E-01	5.33E-01	2.45	12.43	6.46E-03
P3R3URF	5.80E-01	5.33E-01	2.45	12.42	6.47E-03
LINC00640	5.79E-01	5.33E-01	2.45	12.40	6.51E-03
INO80B	5.79E-01	5.32E-01	2.45	12.39	6.52E-03
COG8	5.79E-01	5.32E-01	2.45	12.38	6.53E-03
LOC107986884	5.79E-01	5.32E-01	2.45	12.37	6.54E-03
RNU7.1	5.79E-01	5.32E-01	2.46	12.36	6.56E-03
CENPS	5.79E-01	5.32E-01	2.46	12.36	6.56E-03
HNF1B	5.78E-01	5.31E-01	2.46	12.30	6.64E-03
LINC00459	5.77E-01	5.30E-01	2.46	12.29	6.66E-03
MROCKI	5.77E-01	5.30E-01	2.46	12.27	6.70E-03
LOC105377498	5.77E-01	5.30E-01	2.46	12.26	6.71E-03
NRTN	5.76E-01	5.29E-01	2.46	12.25	6.73E-03
IGLV10.67	5.76E-01	5.29E-01	2.46	12.24	6.74E-03
LOC105377537	5.76E-01	5.29E-01	2.46	12.23	6.75E-03
MUC5AC	5.76E-01	5.29E-01	2.46	12.23	6.76E-03
LOC105378040	5.76E-01	5.29E-01	2.46	12.23	6.76E-03
MIR422A	5.76E-01	5.29E-01	2.46	12.22	6.76E-03
LOC107984654	5.76E-01	5.29E-01	2.46	12.22	6.76E-03
DENND4C	5.76E-01	5.29E-01	2.46	12.22	6.78E-03
XKR6_1	5.76E-01	5.29E-01	2.46	12.21	6.78E-03
LOC105372791	5.76E-01	5.28E-01	2.46	12.21	6.79E-03
LINC01650	5.76E-01	5.28E-01	2.46	12.21	6.79E-03
LOC105371635	5.76E-01	5.28E-01	2.46	12.20	6.79E-03
LOC105377121	5.75E-01	5.28E-01	2.47	12.19	6.82E-03
PTPMT1	5.75E-01	5.28E-01	2.47	12.18	6.83E-03
MIR6512	5.75E-01	5.28E-01	2.47	12.18	6.83E-03
TRK.CTT7.1	5.75E-01	5.28E-01	2.47	12.18	6.83E-03
LOC105375789	5.75E-01	5.28E-01	2.47	12.18	6.83E-03
LOC105372602	5.75E-01	5.28E-01	2.47	12.17	6.84E-03
LOC105369809	5.75E-01	5.28E-01	2.47	12.17	6.84E-03
LOC101929536	5.75E-01	5.27E-01	2.47	12.16	6.86E-03
MRPL52	5.75E-01	5.27E-01	2.47	12.16	6.87E-03
NAPEPLD_1	5.75E-01	5.27E-01	2.47	12.16	6.87E-03

FRMPD2B	5.75E-01	5.27E-01	2.47	12.15	6.87E-03
TMC3.AS1	5.74E-01	5.27E-01	2.47	12.15	6.88E-03
LOC105369969	5.74E-01	5.27E-01	2.47	12.14	6.89E-03
TRC.GCA2.3	5.74E-01	5.27E-01	2.47	12.14	6.90E-03
WWTR1.AS1	5.74E-01	5.27E-01	2.47	12.13	6.91E-03
TRS.GCT6.1	5.74E-01	5.27E-01	2.47	12.12	6.92E-03
LOC105378603	5.74E-01	5.26E-01	2.47	12.11	6.93E-03
MIR4689	5.74E-01	5.26E-01	2.47	12.11	6.93E-03
RNVU1.19	5.74E-01	5.26E-01	2.47	12.11	6.93E-03
MIR190B	5.74E-01	5.26E-01	2.47	12.11	6.93E-03
HAGLROS	5.74E-01	5.26E-01	2.47	12.11	6.93E-03
MIR7845	5.74E-01	5.26E-01	2.47	12.11	6.93E-03
MIR572	5.74E-01	5.26E-01	2.47	12.11	6.93E-03
MIR548T	5.74E-01	5.26E-01	2.47	12.11	6.93E-03
MIR3145	5.74E-01	5.26E-01	2.47	12.11	6.93E-03
MIR4655	5.74E-01	5.26E-01	2.47	12.11	6.93E-03
MIR5692A1	5.74E-01	5.26E-01	2.47	12.11	6.93E-03
MIR3666	5.74E-01	5.26E-01	2.47	12.11	6.93E-03
TRBV6.5	5.74E-01	5.26E-01	2.47	12.11	6.93E-03
ARHGEF34P	5.74E-01	5.26E-01	2.47	12.11	6.93E-03
LOC101929128	5.74E-01	5.26E-01	2.47	12.11	6.93E-03
FAM86B2	5.74E-01	5.26E-01	2.47	12.11	6.93E-03
MIR30D	5.74E-01	5.26E-01	2.47	12.11	6.93E-03
C8orf31	5.74E-01	5.26E-01	2.47	12.11	6.93E-03
LOC107987066	5.74E-01	5.26E-01	2.47	12.11	6.93E-03
LOC105379453	5.74E-01	5.26E-01	2.47	12.11	6.93E-03
MIR6081	5.74E-01	5.26E-01	2.47	12.11	6.93E-03
MIR3155A	5.74E-01	5.26E-01	2.47	12.11	6.93E-03
DRD4	5.74E-01	5.26E-01	2.47	12.11	6.93E-03
TRR.TCT3.2	5.74E-01	5.26E-01	2.47	12.11	6.93E-03
TRS.GCT3.1	5.74E-01	5.26E-01	2.47	12.11	6.93E-03
SNORD14D	5.74E-01	5.26E-01	2.47	12.11	6.93E-03
SNORD14C	5.74E-01	5.26E-01	2.47	12.11	6.93E-03
C1RL	5.74E-01	5.26E-01	2.47	12.11	6.93E-03
MIR4498	5.74E-01	5.26E-01	2.47	12.11	6.93E-03
MIR4305	5.74E-01	5.26E-01	2.47	12.11	6.93E-03
TRAJ38	5.74E-01	5.26E-01	2.47	12.11	6.93E-03
TRAJ8	5.74E-01	5.26E-01	2.47	12.11	6.93E-03
TRAJ3	5.74E-01	5.26E-01	2.47	12.11	6.93E-03
OR4M2	5.74E-01	5.26E-01	2.47	12.11	6.93E-03
SNORD115.1	5.74E-01	5.26E-01	2.47	12.11	6.93E-03

LOC112268159	5.74E-01	5.26E-01	2.47	12.11	6.93E-03
LOC105370926	5.74E-01	5.26E-01	2.47	12.11	6.93E-03
LINC00254	5.74E-01	5.26E-01	2.47	12.11	6.93E-03
SLX1A.SULT1A3	5.74E-01	5.26E-01	2.47	12.11	6.93E-03
MIR195	5.74E-01	5.26E-01	2.47	12.11	6.93E-03
MIR6883	5.74E-01	5.26E-01	2.47	12.11	6.93E-03
LOC105371754	5.74E-01	5.26E-01	2.47	12.11	6.93E-03
MAPT.IT1	5.74E-01	5.26E-01	2.47	12.11	6.93E-03
LOC107985023	5.74E-01	5.26E-01	2.47	12.11	6.93E-03
MIR1268B	5.74E-01	5.26E-01	2.47	12.11	6.93E-03
MIR4322	5.74E-01	5.26E-01	2.47	12.11	6.93E-03
ZNF100	5.74E-01	5.26E-01	2.47	12.11	6.93E-03
MIR296	5.74E-01	5.26E-01	2.47	12.11	6.93E-03
MIR124.3	5.74E-01	5.26E-01	2.47	12.11	6.93E-03
LOC107987288	5.74E-01	5.26E-01	2.47	12.11	6.93E-03
MIR12114	5.74E-01	5.26E-01	2.47	12.11	6.93E-03
MIR500A	5.74E-01	5.26E-01	2.47	12.11	6.93E-03
MAGED4	5.74E-01	5.26E-01	2.47	12.11	6.93E-03
TTTY2B	5.74E-01	5.26E-01	2.47	12.11	6.93E-03
C8orf74_1	5.74E-01	5.26E-01	2.47	12.11	6.93E-03
PRSS22_1	5.74E-01	5.26E-01	2.47	12.11	6.93E-03
LGALS7_1	5.74E-01	5.26E-01	2.47	12.11	6.93E-03
KIR3DL3_9	5.74E-01	5.26E-01	2.47	12.11	6.93E-03
PRAMEF18_3	5.74E-01	5.26E-01	2.47	12.11	6.93E-03
LOC105374167_1	5.74E-01	5.26E-01	2.47	12.11	6.93E-03
LOC107986177_1	5.74E-01	5.26E-01	2.47	12.11	6.93E-03
TRBV12.2_1	5.74E-01	5.26E-01	2.47	12.11	6.93E-03
TAS2R19_1	5.74E-01	5.26E-01	2.47	12.11	6.93E-03
TAS2R46_1	5.74E-01	5.26E-01	2.47	12.11	6.93E-03
CYFIP1_2	5.74E-01	5.26E-01	2.47	12.11	6.93E-03
NTAN1_1	5.74E-01	5.26E-01	2.47	12.11	6.93E-03
LOC105379605	5.74E-01	5.26E-01	2.47	12.11	6.93E-03
GUSBP15_1	5.74E-01	5.26E-01	2.47	12.11	6.93E-03
HLA.DQA1_1	5.74E-01	5.26E-01	2.47	12.11	6.93E-03
HLA.DQA2_2	5.74E-01	5.26E-01	2.47	12.11	6.93E-03
ZNRD1ASP_4	5.74E-01	5.26E-01	2.47	12.11	6.93E-03
OR12D2_5	5.74E-01	5.26E-01	2.47	12.11	6.93E-03
HLA.DQA1_6	5.74E-01	5.26E-01	2.47	12.11	6.93E-03
HLA.DQA2_7	5.74E-01	5.26E-01	2.47	12.11	6.93E-03
LILRP2_18	5.74E-01	5.26E-01	2.47	12.11	6.93E-03
LOC105377784_1	5.74E-01	5.26E-01	2.47	12.11	6.93E-03

GPR179	5.74E-01	5.26E-01	2.47	12.11	6.95E-03
PCDHGA8	5.74E-01	5.26E-01	2.47	12.10	6.95E-03
PNKY	5.73E-01	5.26E-01	2.47	12.09	6.97E-03
LOC107986068	5.73E-01	5.25E-01	2.47	12.07	7.00E-03
HOXC4	5.73E-01	5.25E-01	2.47	12.07	7.01E-03
LOC112268270	5.73E-01	5.25E-01	2.47	12.06	7.01E-03
SNORD13I	5.73E-01	5.25E-01	2.47	12.06	7.02E-03
SNORD115.32	5.73E-01	5.25E-01	2.47	12.06	7.02E-03
TRV.CAC1.5	5.73E-01	5.25E-01	2.47	12.06	7.02E-03
MIR4513	5.73E-01	5.25E-01	2.47	12.06	7.02E-03
IGLVIV.53	5.73E-01	5.25E-01	2.47	12.05	7.03E-03
BATF2	5.72E-01	5.24E-01	2.47	12.03	7.07E-03
LINC00421	5.72E-01	5.24E-01	2.48	12.01	7.09E-03
PCDHGC4	5.72E-01	5.24E-01	2.48	12.01	7.10E-03
MIR449A	5.71E-01	5.24E-01	2.48	12.00	7.11E-03
SNORA25	5.71E-01	5.24E-01	2.48	11.99	7.13E-03
LOC102723530	5.71E-01	5.24E-01	2.48	11.99	7.13E-03
LOC105376286	5.71E-01	5.24E-01	2.48	11.99	7.13E-03
LOC112268195	5.71E-01	5.23E-01	2.48	11.97	7.16E-03
LINC01089	5.71E-01	5.23E-01	2.48	11.96	7.17E-03
MIR6079	5.71E-01	5.23E-01	2.48	11.96	7.19E-03
TRAJ36	5.71E-01	5.23E-01	2.48	11.96	7.19E-03
TRAJ47	5.70E-01	5.22E-01	2.48	11.93	7.23E-03
H4C11	5.70E-01	5.22E-01	2.48	11.93	7.23E-03
LOC105376316	5.70E-01	5.22E-01	2.48	11.93	7.23E-03
OR5V1_3	5.70E-01	5.22E-01	2.48	11.92	7.25E-03
MIR519D	5.70E-01	5.22E-01	2.48	11.91	7.27E-03
LOC105378250	5.69E-01	5.22E-01	2.48	11.90	7.27E-03
JSRP1	5.69E-01	5.22E-01	2.48	11.90	7.27E-03
LOC107986757	5.69E-01	5.22E-01	2.48	11.90	7.28E-03
TRK.CTT2.2	5.69E-01	5.21E-01	2.48	11.90	7.29E-03
TIMP1	5.69E-01	5.21E-01	2.48	11.89	7.29E-03
TRDJ1	5.69E-01	5.21E-01	2.48	11.87	7.32E-03
MIR12126	5.68E-01	5.20E-01	2.49	11.84	7.37E-03
KDM4A.AS1	5.68E-01	5.20E-01	2.49	11.84	7.38E-03
FOXP4.AS1	5.68E-01	5.20E-01	2.49	11.84	7.38E-03
ACTB	5.68E-01	5.20E-01	2.49	11.81	7.43E-03
MICA_3	5.67E-01	5.19E-01	2.49	11.80	7.45E-03
LOC105374402	5.67E-01	5.19E-01	2.49	11.80	7.45E-03
SOCS1	5.67E-01	5.19E-01	2.49	11.79	7.46E-03
TRK.TTT5.1	5.67E-01	5.19E-01	2.49	11.79	7.47E-03

TRK.TTT3.5	5.67E-01	5.19E-01	2.49	11.79	7.47E-03
TRAJ54	5.67E-01	5.19E-01	2.49	11.79	7.47E-03
LOC105377733	5.67E-01	5.19E-01	2.49	11.78	7.47E-03
PDHA2	5.67E-01	5.19E-01	2.49	11.77	7.50E-03
MIR653	5.67E-01	5.18E-01	2.49	11.77	7.50E-03
C1orf122	5.67E-01	5.18E-01	2.49	11.76	7.51E-03
WBP1	5.66E-01	5.18E-01	2.49	11.76	7.52E-03
CTSW	5.66E-01	5.18E-01	2.49	11.75	7.53E-03
CD160	5.66E-01	5.18E-01	2.49	11.75	7.53E-03
MEA1	5.66E-01	5.18E-01	2.49	11.75	7.53E-03
LINC02398	5.66E-01	5.18E-01	2.49	11.74	7.55E-03
LOC652276	5.66E-01	5.18E-01	2.49	11.74	7.56E-03
CRCT1	5.66E-01	5.18E-01	2.49	11.73	7.57E-03
TRY.GTA6.1	5.65E-01	5.17E-01	2.49	11.71	7.60E-03
CHODL.AS1	5.65E-01	5.17E-01	2.49	11.71	7.61E-03
SNORD116.17	5.65E-01	5.17E-01	2.49	11.70	7.62E-03
OARD1	5.65E-01	5.17E-01	2.49	11.70	7.62E-03
SIGMAR1	5.65E-01	5.17E-01	2.50	11.69	7.65E-03
GNGT1	5.65E-01	5.16E-01	2.50	11.67	7.68E-03
LOC107986204	5.64E-01	5.16E-01	2.50	11.66	7.69E-03
LOC101928953	5.64E-01	5.15E-01	2.50	11.64	7.73E-03
SNORD114.14	5.64E-01	5.15E-01	2.50	11.62	7.76E-03
LOC105376154	5.63E-01	5.15E-01	2.50	11.62	7.77E-03
IGLV5.48	5.63E-01	5.15E-01	2.50	11.61	7.78E-03
NAP1L4	5.63E-01	5.15E-01	2.50	11.60	7.79E-03
LOC112268355	5.63E-01	5.15E-01	2.50	11.60	7.80E-03
GPR151	5.63E-01	5.14E-01	2.50	11.60	7.81E-03
MMP24OS	5.63E-01	5.14E-01	2.50	11.60	7.81E-03
LOC101928475	5.62E-01	5.14E-01	2.50	11.57	7.86E-03
MIR501	5.62E-01	5.14E-01	2.50	11.57	7.86E-03
TRAJ19	5.62E-01	5.14E-01	2.50	11.57	7.86E-03
ABR_2	5.62E-01	5.14E-01	2.50	11.57	7.86E-03
PLA2G1B	5.62E-01	5.14E-01	2.50	11.56	7.88E-03
SNORD114.25	5.61E-01	5.12E-01	2.51	11.50	7.98E-03
LOC105376341	5.61E-01	5.12E-01	2.51	11.50	7.98E-03
LOC101928718	5.61E-01	5.12E-01	2.51	11.49	7.99E-03
PIAS1	5.60E-01	5.11E-01	2.51	11.45	8.09E-03
TRK.TTT1.1	5.60E-01	5.11E-01	2.51	11.44	8.10E-03
LOC105372246	5.59E-01	5.11E-01	2.51	11.43	8.12E-03
LOC107984609	5.59E-01	5.10E-01	2.51	11.41	8.16E-03
CES5A_1	5.59E-01	5.10E-01	2.51	11.41	8.16E-03

GPC3.AS1	5.59E-01	5.10E-01	2.51	11.40	8.18E-03
LOC105370689	5.59E-01	5.10E-01	2.51	11.40	8.18E-03
NR2C2AP	5.58E-01	5.09E-01	2.51	11.39	8.20E-03
LINC01574	5.58E-01	5.09E-01	2.51	11.38	8.21E-03
LOC107986948	5.58E-01	5.09E-01	2.51	11.38	8.22E-03
NADK	5.58E-01	5.09E-01	2.51	11.37	8.22E-03
MTMR9_1	5.58E-01	5.09E-01	2.51	11.37	8.23E-03
LRMDA	5.58E-01	5.09E-01	2.51	11.37	8.23E-03
ARMCX2	5.58E-01	5.09E-01	2.51	11.37	8.24E-03
KRTAP6.3	5.58E-01	5.09E-01	2.51	11.36	8.24E-03
MRPS28	5.58E-01	5.09E-01	2.51	11.36	8.25E-03
LOC107984124	5.58E-01	5.09E-01	2.52	11.35	8.26E-03
MIR2052	5.58E-01	5.09E-01	2.52	11.35	8.27E-03
LOC105374054	5.57E-01	5.08E-01	2.52	11.34	8.29E-03
LOC107986944	5.57E-01	5.08E-01	2.52	11.33	8.31E-03
LOC107986578	5.57E-01	5.08E-01	2.52	11.32	8.33E-03
AREG	5.57E-01	5.08E-01	2.52	11.31	8.34E-03
MYRIP	5.57E-01	5.08E-01	2.52	11.31	8.34E-03
GPC4	5.57E-01	5.08E-01	2.52	11.31	8.35E-03
RDH8	5.57E-01	5.07E-01	2.52	11.30	8.37E-03
LOC105371276	5.56E-01	5.07E-01	2.52	11.26	8.44E-03
LOC107986747	5.56E-01	5.06E-01	2.52	11.26	8.45E-03
LOC107984188	5.56E-01	5.06E-01	2.52	11.26	8.45E-03
ARL11	5.56E-01	5.06E-01	2.52	11.25	8.47E-03
CMTR2	5.56E-01	5.06E-01	2.52	11.25	8.47E-03
RPRM	5.55E-01	5.06E-01	2.52	11.23	8.51E-03
MUC4_3	5.55E-01	5.06E-01	2.52	11.23	8.51E-03
P2RX1	5.55E-01	5.05E-01	2.52	11.22	8.53E-03
C11orf94	5.54E-01	5.05E-01	2.53	11.19	8.58E-03
LYPLAL1.DT	5.54E-01	5.04E-01	2.53	11.18	8.61E-03
PRSS57	5.54E-01	5.04E-01	2.53	11.17	8.63E-03
MIR548H5	5.53E-01	5.04E-01	2.53	11.16	8.66E-03
LOC105371167	5.53E-01	5.04E-01	2.53	11.15	8.66E-03
WFDC6	5.53E-01	5.04E-01	2.53	11.15	8.67E-03
LOC105370871	5.53E-01	5.04E-01	2.53	11.14	8.68E-03
LOC105373493	5.53E-01	5.03E-01	2.53	11.14	8.70E-03
PRRX2.AS1	5.53E-01	5.03E-01	2.53	11.13	8.72E-03
PDLIM1	5.53E-01	5.03E-01	2.53	11.12	8.73E-03
LOC107986453	5.52E-01	5.03E-01	2.53	11.10	8.77E-03
LINC01093	5.52E-01	5.02E-01	2.53	11.09	8.80E-03
LINC00052	5.52E-01	5.02E-01	2.53	11.08	8.81E-03

LOC107985500	5.52E-01	5.02E-01	2.53	11.08	8.81E-03
LOC105378866	5.51E-01	5.02E-01	2.53	11.07	8.85E-03
LOC105372920	5.51E-01	5.02E-01	2.53	11.06	8.85E-03
LOC105373408	5.51E-01	5.01E-01	2.53	11.05	8.88E-03
LOC102723760	5.51E-01	5.01E-01	2.53	11.04	8.90E-03
LOC100128288	5.51E-01	5.01E-01	2.53	11.04	8.91E-03
MIR223	5.51E-01	5.01E-01	2.54	11.04	8.91E-03
LOC105371318	5.50E-01	5.00E-01	2.54	11.01	8.96E-03
LOC105370002	5.50E-01	5.00E-01	2.54	11.01	8.98E-03
MIR548Q	5.50E-01	5.00E-01	2.54	11.00	8.98E-03
MIR5590	5.50E-01	5.00E-01	2.54	10.99	9.01E-03
SNORD53	5.50E-01	5.00E-01	2.54	10.98	9.03E-03
LINC01194	5.50E-01	5.00E-01	2.54	10.98	9.03E-03
TRBV7.4	5.50E-01	4.99E-01	2.54	10.98	9.03E-03
LOC107987417	5.50E-01	4.99E-01	2.54	10.98	9.03E-03
LOC105377137	5.50E-01	4.99E-01	2.54	10.98	9.03E-03
LOC105371468	5.50E-01	4.99E-01	2.54	10.98	9.04E-03
GJD4	5.49E-01	4.99E-01	2.54	10.98	9.04E-03
LOC102723811	5.49E-01	4.99E-01	2.54	10.96	9.08E-03
TRAV12.3	5.49E-01	4.99E-01	2.54	10.94	9.11E-03
LOC100130172	5.48E-01	4.98E-01	2.54	10.91	9.18E-03
TTC8	5.48E-01	4.98E-01	2.54	10.91	9.18E-03
LOC101929154	5.48E-01	4.97E-01	2.54	10.89	9.23E-03
LOC105372508	5.47E-01	4.97E-01	2.54	10.89	9.23E-03
LOC105375775	5.47E-01	4.97E-01	2.54	10.89	9.24E-03
LOC107986639	5.47E-01	4.97E-01	2.55	10.86	9.29E-03
SPTBN1	5.47E-01	4.96E-01	2.55	10.86	9.30E-03
LINC02409	5.47E-01	4.96E-01	2.55	10.86	9.30E-03
RNF185	5.46E-01	4.96E-01	2.55	10.85	9.33E-03
LOC107984273	5.46E-01	4.96E-01	2.55	10.84	9.34E-03
SLIRP	5.46E-01	4.96E-01	2.55	10.83	9.37E-03
TMSB15A	5.46E-01	4.95E-01	2.55	10.82	9.39E-03
RERG.AS1	5.46E-01	4.95E-01	2.55	10.81	9.41E-03
LOC101927839	5.45E-01	4.95E-01	2.55	10.80	9.43E-03
LOC105371989	5.45E-01	4.95E-01	2.55	10.80	9.44E-03
MIR1269A	5.45E-01	4.95E-01	2.55	10.80	9.44E-03
FMC1	5.45E-01	4.95E-01	2.55	10.80	9.44E-03
ZNF292	5.45E-01	4.94E-01	2.55	10.78	9.48E-03
LOC102724719	5.45E-01	4.94E-01	2.55	10.78	9.48E-03
MIR4791	5.44E-01	4.94E-01	2.55	10.75	9.55E-03
LOC105379466	5.44E-01	4.94E-01	2.55	10.75	9.55E-03

PRSS1	5.44E-01	4.94E-01	2.55	10.75	9.55E-03
HLA.DPB1_2	5.44E-01	4.93E-01	2.55	10.74	9.57E-03
SYNJ2.IT1	5.44E-01	4.93E-01	2.55	10.73	9.58E-03
RIPPLY1	5.43E-01	4.93E-01	2.56	10.71	9.63E-03
EFNA4	5.43E-01	4.93E-01	2.56	10.71	9.64E-03
ALOX12B	5.43E-01	4.93E-01	2.56	10.71	9.65E-03
TRAV6	5.43E-01	4.93E-01	2.56	10.71	9.65E-03
SLC16A3	5.43E-01	4.92E-01	2.56	10.70	9.67E-03
LOC105378319	5.43E-01	4.92E-01	2.56	10.69	9.68E-03
LOC105378271	5.43E-01	4.92E-01	2.56	10.69	9.69E-03
CDK4	5.43E-01	4.92E-01	2.56	10.68	9.70E-03
LOC105369339	5.42E-01	4.91E-01	2.56	10.64	9.80E-03
LOC102546299	5.42E-01	4.91E-01	2.56	10.64	9.81E-03
LOC389765	5.42E-01	4.91E-01	2.56	10.64	9.82E-03
IGKV2D.19	5.42E-01	4.91E-01	2.56	10.63	9.83E-03
CALML3	5.41E-01	4.90E-01	2.56	10.62	9.85E-03
MRPL53	5.41E-01	4.90E-01	2.56	10.62	9.85E-03
LOC105373973	5.41E-01	4.90E-01	2.56	10.61	9.89E-03
CDRT15L2	5.41E-01	4.90E-01	2.56	10.60	9.90E-03
LOC105369673	5.41E-01	4.90E-01	2.56	10.59	9.93E-03
TRP.CGG1.1	5.40E-01	4.89E-01	2.56	10.58	9.96E-03
LOC107987433	5.40E-01	4.89E-01	2.57	10.57	9.98E-03
RNU5E.1	5.40E-01	4.89E-01	2.57	10.56	1.00E-02
HSPB9	5.40E-01	4.89E-01	2.57	10.56	1.00E-02
SPRR1B	5.40E-01	4.88E-01	2.57	10.55	1.00E-02
ADM2	5.40E-01	4.88E-01	2.57	10.54	1.00E-02
LOC101928417	5.39E-01	4.88E-01	2.57	10.54	1.00E-02
PAGE1	5.39E-01	4.88E-01	2.57	10.54	1.01E-02
LOC107986411	5.38E-01	4.87E-01	2.57	10.50	1.02E-02
CDIPT	5.38E-01	4.87E-01	2.57	10.49	1.02E-02
OR2A2_1	5.38E-01	4.87E-01	2.57	10.48	1.02E-02
KDM8	5.38E-01	4.86E-01	2.57	10.47	1.02E-02
LOC105369182	5.38E-01	4.86E-01	2.57	10.47	1.02E-02
PIR.FIGF	5.37E-01	4.86E-01	2.57	10.46	1.03E-02
SNORD3B.2	5.37E-01	4.86E-01	2.57	10.45	1.03E-02
LOC107986329	5.37E-01	4.86E-01	2.57	10.44	1.03E-02
LOC107986621	5.37E-01	4.85E-01	2.57	10.43	1.03E-02
FAM221A	5.37E-01	4.85E-01	2.57	10.43	1.03E-02
LINC02259	5.36E-01	4.85E-01	2.58	10.40	1.04E-02
PRAMEF19	5.36E-01	4.84E-01	2.58	10.40	1.04E-02
HRCT1	5.35E-01	4.84E-01	2.58	10.36	1.05E-02

LOC107987223	5.35E-01	4.84E-01	2.58	10.36	1.05E-02
SNORD50B	5.35E-01	4.83E-01	2.58	10.36	1.05E-02
LOC102724153	5.35E-01	4.83E-01	2.58	10.35	1.05E-02
C11orf86	5.35E-01	4.83E-01	2.58	10.34	1.06E-02
NAA80	5.34E-01	4.83E-01	2.58	10.33	1.06E-02
SNORD115.10	5.34E-01	4.82E-01	2.58	10.30	1.07E-02
LOC107984222	5.33E-01	4.81E-01	2.58	10.29	1.07E-02
CENPV	5.33E-01	4.81E-01	2.58	10.28	1.07E-02
PIGH	5.33E-01	4.81E-01	2.58	10.27	1.07E-02
LOC105370195	5.33E-01	4.81E-01	2.58	10.27	1.07E-02
LOC105369383	5.33E-01	4.81E-01	2.59	10.25	1.08E-02
LOC105373053	5.33E-01	4.81E-01	2.59	10.25	1.08E-02
IGLV3.1	5.32E-01	4.81E-01	2.59	10.25	1.08E-02
MIR4268	5.32E-01	4.81E-01	2.59	10.25	1.08E-02
MIR4320	5.32E-01	4.81E-01	2.59	10.25	1.08E-02
LINC02400	5.32E-01	4.80E-01	2.59	10.23	1.09E-02
LOC101930100	5.31E-01	4.79E-01	2.59	10.20	1.09E-02
LINC00672	5.31E-01	4.79E-01	2.59	10.20	1.09E-02
TSC22D3	5.31E-01	4.79E-01	2.59	10.19	1.10E-02
LOC105373937	5.31E-01	4.79E-01	2.59	10.19	1.10E-02
LOC728660	5.31E-01	4.79E-01	2.59	10.19	1.10E-02
POT1.AS1	5.31E-01	4.78E-01	2.59	10.17	1.10E-02
LOC107984716	5.31E-01	4.78E-01	2.59	10.17	1.10E-02
MCM4	5.30E-01	4.78E-01	2.59	10.17	1.10E-02
LY6E.DT	5.30E-01	4.78E-01	2.59	10.16	1.11E-02
CERS3.AS1	5.30E-01	4.78E-01	2.59	10.15	1.11E-02
PHACTR3.AS1	5.30E-01	4.78E-01	2.59	10.14	1.11E-02
LOC105373770	5.30E-01	4.77E-01	2.59	10.13	1.11E-02
GRAP2	5.29E-01	4.77E-01	2.59	10.13	1.11E-02
LOC105373250	5.29E-01	4.77E-01	2.60	10.12	1.12E-02
LOC105377501	5.29E-01	4.77E-01	2.60	10.12	1.12E-02
PRL	5.29E-01	4.77E-01	2.60	10.12	1.12E-02
PEMT	5.29E-01	4.77E-01	2.60	10.11	1.12E-02
LOC105369686	5.29E-01	4.76E-01	2.60	10.09	1.12E-02
KIR3DS1	5.29E-01	4.76E-01	2.60	10.09	1.12E-02
CDX1	5.29E-01	4.76E-01	2.60	10.09	1.13E-02
LOC112268325	5.28E-01	4.76E-01	2.60	10.08	1.13E-02
OR3A4P	5.28E-01	4.75E-01	2.60	10.06	1.13E-02
CAPSL	5.28E-01	4.75E-01	2.60	10.06	1.13E-02
IGKV3D.31	5.27E-01	4.74E-01	2.60	10.02	1.14E-02
CCDC105	5.27E-01	4.74E-01	2.60	10.02	1.14E-02

SNORD103C	5.27E-01	4.74E-01	2.60	10.01	1.15E-02
LOC105371311	5.25E-01	4.73E-01	2.61	9.97	1.16E-02
PRSS38	5.25E-01	4.73E-01	2.61	9.96	1.16E-02
AP3S2	5.25E-01	4.72E-01	2.61	9.94	1.17E-02
LINC01958	5.25E-01	4.72E-01	2.61	9.94	1.17E-02
SLC12A5.AS1	5.25E-01	4.72E-01	2.61	9.94	1.17E-02
LOC105378906	5.25E-01	4.72E-01	2.61	9.93	1.17E-02
LINC01484	5.24E-01	4.72E-01	2.61	9.93	1.17E-02
APOA2	5.24E-01	4.72E-01	2.61	9.92	1.17E-02
LOC107985945	5.24E-01	4.71E-01	2.61	9.92	1.17E-02
AGAP13P	5.24E-01	4.71E-01	2.61	9.91	1.18E-02
IGLV3.17	5.24E-01	4.71E-01	2.61	9.91	1.18E-02
LOC105369976	5.24E-01	4.71E-01	2.61	9.90	1.18E-02
OVOS2	5.23E-01	4.70E-01	2.61	9.87	1.19E-02
LOC729609	5.23E-01	4.70E-01	2.61	9.87	1.19E-02
LOC149684	5.23E-01	4.70E-01	2.61	9.86	1.19E-02
LOC105377342	5.23E-01	4.70E-01	2.61	9.86	1.19E-02
C17orf58	5.23E-01	4.70E-01	2.61	9.86	1.19E-02
LOC105374811	5.23E-01	4.70E-01	2.61	9.86	1.19E-02
LINC01164	5.23E-01	4.70E-01	2.61	9.85	1.19E-02
PET117	5.23E-01	4.70E-01	2.61	9.85	1.19E-02
RBMXL2	5.22E-01	4.69E-01	2.61	9.85	1.20E-02
PRKAG2.AS1	5.22E-01	4.69E-01	2.61	9.84	1.20E-02
CSF1	5.22E-01	4.69E-01	2.62	9.83	1.20E-02
LOC105370957	5.22E-01	4.69E-01	2.62	9.82	1.20E-02
LINC02128	5.22E-01	4.69E-01	2.62	9.82	1.21E-02
CCL3L1	5.22E-01	4.68E-01	2.62	9.81	1.21E-02
LOC107984833	5.21E-01	4.68E-01	2.62	9.80	1.21E-02
TMPRSS2	5.21E-01	4.68E-01	2.62	9.78	1.22E-02
LINC02541	5.21E-01	4.68E-01	2.62	9.78	1.22E-02
ZNF326	5.21E-01	4.67E-01	2.62	9.78	1.22E-02
LOC107985203	5.20E-01	4.67E-01	2.62	9.77	1.22E-02
LOC107984625	5.20E-01	4.67E-01	2.62	9.76	1.22E-02
NOTO	5.20E-01	4.67E-01	2.62	9.75	1.23E-02
LOC105372686	5.20E-01	4.67E-01	2.62	9.75	1.23E-02
LOC105369975	5.20E-01	4.67E-01	2.62	9.75	1.23E-02
RNF113B	5.20E-01	4.66E-01	2.62	9.74	1.23E-02
LINC02755	5.19E-01	4.66E-01	2.62	9.73	1.23E-02
LOC107984202	5.19E-01	4.66E-01	2.62	9.73	1.23E-02
ARFIP2	5.19E-01	4.66E-01	2.62	9.72	1.24E-02
MIR8054	5.19E-01	4.66E-01	2.62	9.72	1.24E-02

LOC102723996	5.19E-01	4.65E-01	2.62	9.71	1.24E-02
SNORA95	5.19E-01	4.65E-01	2.62	9.70	1.24E-02
FKBP1A	5.19E-01	4.65E-01	2.62	9.69	1.25E-02
NEUROD6	5.18E-01	4.65E-01	2.63	9.68	1.25E-02
PDZD4	5.18E-01	4.65E-01	2.63	9.68	1.25E-02
LOC105376353	5.18E-01	4.65E-01	2.63	9.68	1.25E-02
CKS1B	5.18E-01	4.64E-01	2.63	9.67	1.25E-02
CORO1A	5.18E-01	4.64E-01	2.63	9.66	1.26E-02
LOC105372236	5.18E-01	4.64E-01	2.63	9.65	1.26E-02
MIR4694	5.18E-01	4.64E-01	2.63	9.65	1.26E-02
LINC01381	5.17E-01	4.64E-01	2.63	9.65	1.26E-02
CTD.2350J17.1	5.17E-01	4.64E-01	2.63	9.65	1.26E-02
PNMA8B	5.17E-01	4.64E-01	2.63	9.65	1.26E-02
LOC105372084	5.17E-01	4.64E-01	2.63	9.64	1.26E-02
LOC105370600	5.17E-01	4.64E-01	2.63	9.64	1.26E-02
LOC107986097	5.17E-01	4.63E-01	2.63	9.63	1.26E-02
LOC107986178	5.17E-01	4.63E-01	2.63	9.63	1.27E-02
SMPX	5.17E-01	4.63E-01	2.63	9.62	1.27E-02
TSPAN32	5.17E-01	4.63E-01	2.63	9.62	1.27E-02
NDUFB8	5.16E-01	4.63E-01	2.63	9.61	1.27E-02
PXMP2	5.16E-01	4.63E-01	2.63	9.60	1.27E-02
MIR4282	5.16E-01	4.62E-01	2.63	9.60	1.28E-02
CA5BP1.CA5B	5.16E-01	4.62E-01	2.63	9.60	1.28E-02
LOC105373856	5.16E-01	4.62E-01	2.63	9.59	1.28E-02
LOC100506100	5.16E-01	4.62E-01	2.63	9.59	1.28E-02
DPP3	5.16E-01	4.62E-01	2.63	9.59	1.28E-02
DEFB106A	5.16E-01	4.62E-01	2.63	9.59	1.28E-02
OCIAD1.AS1	5.15E-01	4.62E-01	2.63	9.57	1.29E-02
MIPEPP3	5.15E-01	4.61E-01	2.63	9.57	1.29E-02
DKFZp434L192	5.15E-01	4.61E-01	2.63	9.56	1.29E-02
CDAN1	5.15E-01	4.61E-01	2.63	9.56	1.29E-02
PSME3	5.15E-01	4.61E-01	2.63	9.55	1.29E-02
MAPK11	5.15E-01	4.61E-01	2.63	9.55	1.29E-02
LOC102723536	5.15E-01	4.61E-01	2.64	9.55	1.29E-02
RELB	5.15E-01	4.61E-01	2.64	9.54	1.29E-02
LOC105379083	5.15E-01	4.61E-01	2.64	9.54	1.30E-02
LOC102723665	5.14E-01	4.61E-01	2.64	9.54	1.30E-02
LOC112268243	5.14E-01	4.60E-01	2.64	9.51	1.31E-02
LOC107987143	5.14E-01	4.60E-01	2.64	9.51	1.31E-02
LOC283299_2	5.14E-01	4.60E-01	2.64	9.50	1.31E-02
LOC105379435	5.14E-01	4.60E-01	2.64	9.50	1.31E-02

RPL10L	5.13E-01	4.59E-01	2.64	9.48	1.31E-02
C4B_2_1	5.13E-01	4.59E-01	2.64	9.48	1.32E-02
C4B_3	5.13E-01	4.59E-01	2.64	9.48	1.32E-02
CADM2.AS2	5.13E-01	4.59E-01	2.64	9.48	1.32E-02
LINC00499	5.13E-01	4.59E-01	2.64	9.47	1.32E-02
RRN3P3	5.12E-01	4.58E-01	2.64	9.45	1.32E-02
LOC102723564	5.12E-01	4.58E-01	2.64	9.45	1.32E-02
SNORD38B	5.12E-01	4.58E-01	2.64	9.45	1.32E-02
GUSBP16	5.12E-01	4.58E-01	2.64	9.45	1.32E-02
LOC105372470	5.12E-01	4.58E-01	2.64	9.45	1.33E-02
LRIT2	5.12E-01	4.58E-01	2.64	9.44	1.33E-02
MFAP4	5.12E-01	4.58E-01	2.64	9.44	1.33E-02
LOC101928855	5.12E-01	4.57E-01	2.64	9.43	1.33E-02
MIDN	5.12E-01	4.57E-01	2.64	9.43	1.33E-02
LOC107986817	5.12E-01	4.57E-01	2.64	9.42	1.34E-02
HSD17B3.AS1	5.11E-01	4.57E-01	2.64	9.41	1.34E-02
ALDH3B2	5.11E-01	4.57E-01	2.65	9.41	1.34E-02
LINC02126	5.11E-01	4.57E-01	2.65	9.40	1.34E-02
EPN2.IT1	5.11E-01	4.57E-01	2.65	9.40	1.34E-02
LOC105372283	5.11E-01	4.56E-01	2.65	9.39	1.35E-02
NAA25	5.11E-01	4.56E-01	2.65	9.39	1.35E-02
LOC105378883	5.10E-01	4.56E-01	2.65	9.37	1.35E-02
LOC105376876	5.10E-01	4.56E-01	2.65	9.37	1.35E-02
LOC107985579	5.10E-01	4.56E-01	2.65	9.37	1.35E-02
TMEM52	5.10E-01	4.56E-01	2.65	9.37	1.35E-02
H2AZ1.DT	5.10E-01	4.56E-01	2.65	9.37	1.35E-02
S100A1	5.09E-01	4.55E-01	2.65	9.35	1.36E-02
TRV.CAC1.1	5.09E-01	4.55E-01	2.65	9.35	1.36E-02
MIR802	5.09E-01	4.55E-01	2.65	9.35	1.36E-02
LILRB4_2	5.09E-01	4.55E-01	2.65	9.35	1.36E-02
TSN	5.09E-01	4.55E-01	2.65	9.35	1.36E-02
MIR3713	5.09E-01	4.55E-01	2.65	9.34	1.37E-02
DOCK8.AS1	5.09E-01	4.55E-01	2.65	9.34	1.37E-02
SCNM1	5.09E-01	4.54E-01	2.65	9.32	1.37E-02
LOC105377940	5.09E-01	4.54E-01	2.65	9.32	1.37E-02
PPIAL4C	5.09E-01	4.54E-01	2.65	9.31	1.38E-02
BCL2L10	5.08E-01	4.54E-01	2.65	9.31	1.38E-02
NADK2	5.08E-01	4.53E-01	2.65	9.29	1.38E-02
DONSON	5.08E-01	4.53E-01	2.65	9.28	1.39E-02
SPRR3	5.08E-01	4.53E-01	2.65	9.28	1.39E-02
TMEM30CP	5.08E-01	4.53E-01	2.65	9.28	1.39E-02

LOC105371382	5.08E-01	4.53E-01	2.65	9.28	1.39E-02
CDHR5	5.08E-01	4.53E-01	2.65	9.28	1.39E-02
FUT8	5.07E-01	4.53E-01	2.65	9.27	1.39E-02
LOC102724363	5.07E-01	4.53E-01	2.65	9.27	1.39E-02
IPW	5.07E-01	4.52E-01	2.66	9.25	1.40E-02
LOC100132078	5.06E-01	4.51E-01	2.66	9.23	1.41E-02
GDPD2	5.06E-01	4.51E-01	2.66	9.23	1.41E-02
LOC107987384	5.06E-01	4.51E-01	2.66	9.21	1.41E-02
LOC105370340	5.05E-01	4.50E-01	2.66	9.19	1.42E-02
SLC25A2	5.05E-01	4.50E-01	2.66	9.18	1.42E-02
LOC105377178	5.05E-01	4.50E-01	2.66	9.18	1.42E-02
LOC105375107	5.05E-01	4.50E-01	2.66	9.18	1.42E-02
RPL35	5.05E-01	4.50E-01	2.66	9.18	1.43E-02
AGAP6	5.05E-01	4.50E-01	2.66	9.17	1.43E-02
CCL18_1	5.04E-01	4.49E-01	2.66	9.15	1.44E-02
CAMTA1	5.04E-01	4.49E-01	2.66	9.15	1.44E-02
LOC105375701	5.04E-01	4.49E-01	2.66	9.14	1.44E-02
LOC102723714	5.04E-01	4.49E-01	2.66	9.14	1.44E-02
LOC105370738	5.04E-01	4.49E-01	2.66	9.14	1.44E-02
PIP	5.04E-01	4.49E-01	2.66	9.14	1.44E-02
LOC101927623	5.04E-01	4.48E-01	2.67	9.13	1.44E-02
TAS1R3	5.03E-01	4.48E-01	2.67	9.12	1.45E-02
LOC105377411	5.03E-01	4.48E-01	2.67	9.12	1.45E-02
LOC105378728	5.03E-01	4.48E-01	2.67	9.11	1.45E-02
RPL13	5.03E-01	4.48E-01	2.67	9.11	1.45E-02
LOC105373060	5.03E-01	4.47E-01	2.67	9.10	1.46E-02
KRT73.AS1	5.02E-01	4.47E-01	2.67	9.09	1.46E-02
ZMYND10	5.02E-01	4.47E-01	2.67	9.08	1.46E-02
LINC00571	5.02E-01	4.47E-01	2.67	9.08	1.46E-02
LOC101928397	5.02E-01	4.47E-01	2.67	9.07	1.47E-02
LOC100128993	5.02E-01	4.47E-01	2.67	9.07	1.47E-02
LOC105373887	5.01E-01	4.46E-01	2.67	9.05	1.48E-02
MACROH2A2	5.01E-01	4.45E-01	2.67	9.03	1.49E-02
LOC105371378	5.01E-01	4.45E-01	2.67	9.03	1.49E-02
LOC105378394	5.01E-01	4.45E-01	2.67	9.03	1.49E-02
HSF2BP	5.01E-01	4.45E-01	2.67	9.02	1.49E-02
FADD	5.00E-01	4.45E-01	2.67	9.01	1.49E-02
LRRC37A6P	5.00E-01	4.45E-01	2.67	9.01	1.49E-02
SIK1B	5.00E-01	4.45E-01	2.67	9.01	1.49E-02
LOC101928881	5.00E-01	4.45E-01	2.67	9.00	1.49E-02
LOC107986892	5.00E-01	4.44E-01	2.67	9.00	1.50E-02

PCOLCE2	5.00E-01	4.44E-01	2.67	9.00	1.50E-02
ARL14	5.00E-01	4.44E-01	2.68	8.99	1.50E-02
LOC105377814	5.00E-01	4.44E-01	2.68	8.99	1.50E-02
MAGEB6	4.99E-01	4.44E-01	2.68	8.98	1.50E-02
PPM1M	4.99E-01	4.44E-01	2.68	8.97	1.51E-02
LOC101927248	4.99E-01	4.44E-01	2.68	8.97	1.51E-02
SDCBP2.AS1	4.99E-01	4.43E-01	2.68	8.96	1.51E-02
MIR202HG	4.99E-01	4.43E-01	2.68	8.96	1.51E-02
YRDC	4.99E-01	4.43E-01	2.68	8.95	1.52E-02
LOC105378971	4.97E-01	4.42E-01	2.68	8.91	1.53E-02
LOC105378132	4.97E-01	4.42E-01	2.68	8.91	1.53E-02
LOC105379513	4.97E-01	4.41E-01	2.68	8.90	1.54E-02
LOC102724768	4.97E-01	4.41E-01	2.68	8.90	1.54E-02
LOC105371380	4.97E-01	4.41E-01	2.68	8.89	1.54E-02
LOC101927548	4.97E-01	4.41E-01	2.68	8.88	1.54E-02
LOC105376400	4.97E-01	4.41E-01	2.68	8.88	1.54E-02
LAIR1	4.96E-01	4.40E-01	2.68	8.87	1.55E-02
LOC100272217	4.96E-01	4.40E-01	2.69	8.85	1.56E-02
FLJ42351	4.96E-01	4.40E-01	2.69	8.85	1.56E-02
LOC101926977	4.96E-01	4.40E-01	2.69	8.84	1.56E-02
ATF7IP	4.96E-01	4.40E-01	2.69	8.84	1.56E-02
RAB25	4.96E-01	4.39E-01	2.69	8.84	1.56E-02
FLJ37453	4.95E-01	4.39E-01	2.69	8.83	1.57E-02
LOC105373096	4.95E-01	4.39E-01	2.69	8.82	1.57E-02
LOC107985646	4.95E-01	4.38E-01	2.69	8.81	1.58E-02
LOC105369405	4.94E-01	4.38E-01	2.69	8.80	1.58E-02
CFAP99	4.94E-01	4.38E-01	2.69	8.80	1.58E-02
MYO5C	4.94E-01	4.38E-01	2.69	8.80	1.58E-02
LOC105374974	4.94E-01	4.38E-01	2.69	8.79	1.58E-02
GMPPA	4.94E-01	4.38E-01	2.69	8.79	1.59E-02
LINC02033	4.94E-01	4.38E-01	2.69	8.78	1.59E-02
DLGAP1.AS4	4.94E-01	4.37E-01	2.69	8.77	1.59E-02
LOC107987262	4.94E-01	4.37E-01	2.69	8.77	1.59E-02
CLEC18C	4.93E-01	4.37E-01	2.69	8.76	1.60E-02
ARNTL2.AS1	4.93E-01	4.37E-01	2.69	8.76	1.60E-02
TRK.CTT1.1	4.93E-01	4.37E-01	2.69	8.75	1.60E-02
FBXL16	4.93E-01	4.36E-01	2.69	8.74	1.60E-02
SERTAD3	4.93E-01	4.36E-01	2.69	8.74	1.61E-02
MRAP	4.93E-01	4.36E-01	2.69	8.74	1.61E-02
LOC105371554	4.92E-01	4.36E-01	2.69	8.73	1.61E-02
LOC105373894	4.92E-01	4.36E-01	2.70	8.73	1.61E-02

LOC105375038	4.92E-01	4.36E-01	2.70	8.72	1.61E-02
TRNW	4.92E-01	4.35E-01	2.70	8.71	1.62E-02
LOC105370525	4.91E-01	4.35E-01	2.70	8.69	1.63E-02
LOC107986758	4.91E-01	4.35E-01	2.70	8.69	1.63E-02
LOC644277	4.91E-01	4.34E-01	2.70	8.67	1.64E-02
LINC01609	4.91E-01	4.34E-01	2.70	8.67	1.64E-02
MICA.AS1_2	4.91E-01	4.34E-01	2.70	8.67	1.64E-02
IGKV1D.43	4.91E-01	4.34E-01	2.70	8.66	1.64E-02
LOC105374295	4.90E-01	4.33E-01	2.70	8.65	1.65E-02
MIR4765	4.89E-01	4.32E-01	2.70	8.62	1.66E-02
MIR548V	4.89E-01	4.32E-01	2.70	8.61	1.66E-02
LOC112267931	4.89E-01	4.32E-01	2.70	8.61	1.66E-02
LOC107987069	4.89E-01	4.32E-01	2.70	8.61	1.67E-02
LOC105370613	4.89E-01	4.32E-01	2.71	8.60	1.67E-02
MCEE	4.88E-01	4.32E-01	2.71	8.59	1.67E-02
SNORA117	4.88E-01	4.31E-01	2.71	8.58	1.68E-02
LOC105375799_1	4.88E-01	4.31E-01	2.71	8.58	1.68E-02
LINC00280	4.88E-01	4.31E-01	2.71	8.58	1.68E-02
ADIRF	4.88E-01	4.31E-01	2.71	8.58	1.68E-02
TMC4	4.88E-01	4.31E-01	2.71	8.58	1.68E-02
LOC107984714	4.88E-01	4.31E-01	2.71	8.58	1.68E-02
ZNF676	4.88E-01	4.31E-01	2.71	8.57	1.68E-02
LINC02123	4.88E-01	4.31E-01	2.71	8.57	1.68E-02
PABPC1L2B	4.87E-01	4.30E-01	2.71	8.55	1.69E-02
MUC12.AS1	4.87E-01	4.30E-01	2.71	8.54	1.70E-02
LOC105377479	4.87E-01	4.30E-01	2.71	8.53	1.70E-02
LOC107984469	4.86E-01	4.29E-01	2.71	8.52	1.71E-02
LOC105375571	4.86E-01	4.29E-01	2.71	8.51	1.71E-02
CLEC12A.AS1	4.86E-01	4.29E-01	2.71	8.51	1.71E-02
SNORD140	4.86E-01	4.29E-01	2.71	8.51	1.71E-02
LOC107984876	4.86E-01	4.29E-01	2.71	8.50	1.71E-02
LOC107984855	4.86E-01	4.29E-01	2.71	8.50	1.72E-02
SNORD58A	4.86E-01	4.29E-01	2.71	8.50	1.72E-02
LOC107986630	4.86E-01	4.28E-01	2.71	8.50	1.72E-02
LOC102723313	4.86E-01	4.28E-01	2.71	8.49	1.72E-02
NIPA1	4.85E-01	4.28E-01	2.71	8.49	1.72E-02
LOC101929348	4.85E-01	4.28E-01	2.71	8.48	1.73E-02
GPR149	4.85E-01	4.27E-01	2.72	8.46	1.73E-02
LOC105379539	4.84E-01	4.27E-01	2.72	8.45	1.74E-02
LOC101927727	4.84E-01	4.27E-01	2.72	8.45	1.74E-02
HHLA3	4.84E-01	4.27E-01	2.72	8.45	1.74E-02

XIST	4.84E-01	4.27E-01	2.72	8.44	1.74E-02
LINC01698	4.84E-01	4.27E-01	2.72	8.44	1.75E-02
CTSD	4.83E-01	4.25E-01	2.72	8.40	1.77E-02
FBXL12	4.82E-01	4.25E-01	2.72	8.38	1.77E-02
LOC105377975	4.82E-01	4.25E-01	2.72	8.38	1.77E-02
NOC2L	4.82E-01	4.25E-01	2.72	8.38	1.78E-02
RPL7L1	4.82E-01	4.24E-01	2.72	8.38	1.78E-02
LOC105371106	4.82E-01	4.24E-01	2.72	8.37	1.78E-02
GACAT2	4.81E-01	4.24E-01	2.72	8.36	1.79E-02
FLJ36000	4.81E-01	4.24E-01	2.72	8.35	1.79E-02
RYR3.DT	4.81E-01	4.24E-01	2.72	8.35	1.79E-02
LOC100287015	4.81E-01	4.24E-01	2.72	8.35	1.79E-02
LOC105370066	4.81E-01	4.23E-01	2.72	8.34	1.79E-02
LOC107986935	4.81E-01	4.23E-01	2.72	8.34	1.79E-02
LOC105377106	4.81E-01	4.23E-01	2.73	8.34	1.80E-02
HERC2P2_1	4.81E-01	4.23E-01	2.73	8.33	1.80E-02
LOC100335030	4.81E-01	4.23E-01	2.73	8.33	1.80E-02
CLDN5	4.80E-01	4.23E-01	2.73	8.32	1.80E-02
LOC112268172	4.80E-01	4.22E-01	2.73	8.31	1.81E-02
LINC00885	4.80E-01	4.22E-01	2.73	8.31	1.81E-02
KIFC3	4.80E-01	4.22E-01	2.73	8.31	1.81E-02
NPIPA1_1	4.80E-01	4.22E-01	2.73	8.30	1.81E-02
GSTTP2	4.80E-01	4.22E-01	2.73	8.30	1.82E-02
LOC100506178	4.80E-01	4.22E-01	2.73	8.30	1.82E-02
MIR609	4.79E-01	4.22E-01	2.73	8.29	1.82E-02
MIR320E	4.79E-01	4.22E-01	2.73	8.29	1.82E-02
LINC02086	4.79E-01	4.21E-01	2.73	8.29	1.82E-02
MBOAT2	4.79E-01	4.21E-01	2.73	8.28	1.82E-02
LOC105378550	4.79E-01	4.21E-01	2.73	8.28	1.82E-02
LOC554206	4.79E-01	4.21E-01	2.73	8.28	1.82E-02
SF3A2	4.79E-01	4.21E-01	2.73	8.28	1.83E-02
FBXW7	4.79E-01	4.21E-01	2.73	8.27	1.83E-02
LOC102724151	4.79E-01	4.21E-01	2.73	8.27	1.83E-02
SNORD13F	4.79E-01	4.21E-01	2.73	8.27	1.83E-02
TMEM265	4.78E-01	4.20E-01	2.73	8.26	1.84E-02
LINC00977	4.78E-01	4.20E-01	2.73	8.25	1.84E-02
G0S2	4.78E-01	4.20E-01	2.73	8.25	1.84E-02
SNTB2	4.78E-01	4.20E-01	2.73	8.25	1.84E-02
LOC105370958	4.78E-01	4.20E-01	2.73	8.24	1.85E-02
ZNF630.AS1	4.78E-01	4.20E-01	2.73	8.23	1.85E-02
SPINK2	4.78E-01	4.20E-01	2.73	8.23	1.85E-02

CNIH2	4.77E-01	4.19E-01	2.73	8.22	1.85E-02
RPS4X	4.77E-01	4.19E-01	2.73	8.22	1.86E-02
NSG1	4.77E-01	4.19E-01	2.73	8.22	1.86E-02
DRAIC	4.76E-01	4.18E-01	2.74	8.19	1.87E-02
SPOCD1	4.76E-01	4.18E-01	2.74	8.19	1.87E-02
S100A11	4.76E-01	4.18E-01	2.74	8.18	1.88E-02
LOC105377721	4.76E-01	4.17E-01	2.74	8.17	1.88E-02
ENTPD8	4.76E-01	4.17E-01	2.74	8.17	1.88E-02
TAS1R2	4.75E-01	4.17E-01	2.74	8.15	1.89E-02
EFCAB3	4.75E-01	4.17E-01	2.74	8.15	1.89E-02
RAB9BP1	4.75E-01	4.17E-01	2.74	8.15	1.89E-02
SPNS2	4.75E-01	4.16E-01	2.74	8.13	1.90E-02
LINC01297	4.74E-01	4.16E-01	2.74	8.12	1.91E-02
TRIM74	4.74E-01	4.16E-01	2.74	8.12	1.91E-02
LOC105373742	4.74E-01	4.15E-01	2.74	8.11	1.92E-02
LOC100422212	4.74E-01	4.15E-01	2.74	8.11	1.92E-02
THOC7	4.74E-01	4.15E-01	2.74	8.10	1.92E-02
TNNC2	4.74E-01	4.15E-01	2.74	8.10	1.92E-02
IFIT1B	4.74E-01	4.15E-01	2.74	8.10	1.92E-02
SHISA5	4.73E-01	4.15E-01	2.74	8.09	1.93E-02
LHX1_1	4.73E-01	4.15E-01	2.74	8.09	1.93E-02
ZSCAN16.AS1	4.73E-01	4.15E-01	2.75	8.08	1.93E-02
PITPNA	4.73E-01	4.14E-01	2.75	8.07	1.94E-02
LOC107984019	4.73E-01	4.14E-01	2.75	8.07	1.94E-02
H3P6	4.72E-01	4.14E-01	2.75	8.05	1.95E-02
LOC112267959	4.72E-01	4.14E-01	2.75	8.05	1.95E-02
SIX2	4.72E-01	4.14E-01	2.75	8.05	1.95E-02
LINC01919	4.72E-01	4.13E-01	2.75	8.05	1.95E-02
TGFB3	4.72E-01	4.13E-01	2.75	8.04	1.96E-02
OR2L8	4.72E-01	4.13E-01	2.75	8.04	1.96E-02
LOC107987078	4.72E-01	4.13E-01	2.75	8.04	1.96E-02
LEFTY1	4.72E-01	4.13E-01	2.75	8.03	1.96E-02
H2BP1	4.72E-01	4.13E-01	2.75	8.03	1.96E-02
LOC107985694	4.71E-01	4.13E-01	2.75	8.03	1.96E-02
IDI2.AS1	4.71E-01	4.12E-01	2.75	8.02	1.97E-02
DPH3P1	4.71E-01	4.12E-01	2.75	8.01	1.97E-02
FAM197Y7	4.71E-01	4.12E-01	2.75	8.00	1.98E-02
BCO1	4.70E-01	4.12E-01	2.75	8.00	1.98E-02
LOC105377166	4.70E-01	4.12E-01	2.75	7.99	1.98E-02
GALNT4	4.70E-01	4.11E-01	2.75	7.99	1.98E-02
DRD4_1	4.70E-01	4.11E-01	2.75	7.99	1.98E-02

ATOH1	4.70E-01	4.11E-01	2.75	7.98	1.99E-02
ENTPD1	4.70E-01	4.11E-01	2.75	7.97	1.99E-02
LINC01849	4.70E-01	4.11E-01	2.75	7.97	1.99E-02
LOC101059954	4.70E-01	4.11E-01	2.75	7.97	2.00E-02
LINC01586	4.70E-01	4.11E-01	2.75	7.97	2.00E-02
LOC105369647	4.69E-01	4.10E-01	2.76	7.96	2.00E-02
ST7.OT4	4.69E-01	4.10E-01	2.76	7.95	2.00E-02
LRIF1	4.69E-01	4.10E-01	2.76	7.94	2.01E-02
HULC	4.69E-01	4.09E-01	2.76	7.93	2.02E-02
LOC105369329	4.67E-01	4.07E-01	2.76	7.87	2.05E-02
ETV7	4.66E-01	4.07E-01	2.76	7.87	2.06E-02
LOC105374797	4.66E-01	4.07E-01	2.76	7.86	2.06E-02
LOC105371116	4.66E-01	4.07E-01	2.76	7.86	2.06E-02
LINC02772	4.66E-01	4.07E-01	2.76	7.86	2.06E-02
LOC105371024	4.66E-01	4.07E-01	2.76	7.85	2.07E-02
NINJ2.AS1	4.66E-01	4.06E-01	2.76	7.85	2.07E-02
LOC107984381	4.65E-01	4.06E-01	2.77	7.83	2.08E-02
LINC02082	4.65E-01	4.06E-01	2.77	7.82	2.08E-02
DLX4	4.65E-01	4.05E-01	2.77	7.82	2.08E-02
APTR	4.64E-01	4.05E-01	2.77	7.80	2.09E-02
SNRPD2P2	4.64E-01	4.04E-01	2.77	7.79	2.10E-02
WIPI1	4.64E-01	4.04E-01	2.77	7.79	2.10E-02
LOC105371878	4.63E-01	4.04E-01	2.77	7.77	2.11E-02
LINC00548	4.63E-01	4.04E-01	2.77	7.77	2.11E-02
VSNL1	4.63E-01	4.04E-01	2.77	7.76	2.12E-02
CD3D	4.63E-01	4.03E-01	2.77	7.75	2.12E-02
LOC105375050	4.62E-01	4.03E-01	2.77	7.74	2.13E-02
PANO1	4.62E-01	4.02E-01	2.77	7.74	2.14E-02
C15orf39	4.62E-01	4.02E-01	2.77	7.73	2.14E-02
LOC105378515	4.62E-01	4.02E-01	2.77	7.73	2.14E-02
NKRF	4.62E-01	4.02E-01	2.77	7.73	2.14E-02
AMH	4.61E-01	4.01E-01	2.78	7.69	2.16E-02
GASK1A	4.60E-01	4.01E-01	2.78	7.68	2.17E-02
LOC102546229	4.60E-01	4.00E-01	2.78	7.68	2.17E-02
MTFR1L	4.60E-01	4.00E-01	2.78	7.67	2.18E-02
KIRREL1	4.60E-01	4.00E-01	2.78	7.66	2.18E-02
LOC105369202	4.60E-01	4.00E-01	2.78	7.66	2.18E-02
MIR8070	4.59E-01	3.99E-01	2.78	7.65	2.19E-02
GUCA1B	4.59E-01	3.99E-01	2.78	7.65	2.19E-02
LOC105377499	4.59E-01	3.99E-01	2.78	7.64	2.20E-02
LINC01419	4.59E-01	3.99E-01	2.78	7.63	2.21E-02

LOC107985158	4.59E-01	3.99E-01	2.78	7.63	2.21E-02
LINC01657	4.58E-01	3.98E-01	2.78	7.62	2.21E-02
MRPL12	4.58E-01	3.98E-01	2.78	7.62	2.21E-02
INHBA	4.58E-01	3.98E-01	2.78	7.62	2.21E-02
DAAM2.AS1	4.58E-01	3.98E-01	2.78	7.61	2.22E-02
HSPA1L_3	4.58E-01	3.98E-01	2.78	7.61	2.22E-02
KIR2DL1_4	4.58E-01	3.98E-01	2.78	7.61	2.22E-02
LOC112267881	4.58E-01	3.98E-01	2.78	7.61	2.22E-02
GNAI3	4.58E-01	3.98E-01	2.79	7.60	2.22E-02
ZNF189	4.58E-01	3.97E-01	2.79	7.60	2.23E-02
IGIP	4.58E-01	3.97E-01	2.79	7.60	2.23E-02
HSPE1	4.58E-01	3.97E-01	2.79	7.59	2.23E-02
TOP1P1	4.58E-01	3.97E-01	2.79	7.59	2.23E-02
TP53BP1	4.57E-01	3.97E-01	2.79	7.58	2.23E-02
PENK	4.57E-01	3.97E-01	2.79	7.58	2.24E-02
TMEM9	4.57E-01	3.97E-01	2.79	7.57	2.24E-02
LOC100129455	4.57E-01	3.97E-01	2.79	7.57	2.24E-02
GPR135	4.57E-01	3.96E-01	2.79	7.57	2.24E-02
LOC105372928	4.57E-01	3.96E-01	2.79	7.57	2.25E-02
LOC101928386	4.56E-01	3.96E-01	2.79	7.55	2.25E-02
DEDD	4.56E-01	3.96E-01	2.79	7.55	2.26E-02
NELFB	4.56E-01	3.96E-01	2.79	7.55	2.26E-02
EIF1	4.56E-01	3.95E-01	2.79	7.54	2.26E-02
LOC105374804	4.55E-01	3.95E-01	2.79	7.53	2.27E-02
ATR	4.55E-01	3.95E-01	2.79	7.53	2.27E-02
NOTCH4_2	4.55E-01	3.94E-01	2.79	7.51	2.28E-02
LOC105378476	4.55E-01	3.94E-01	2.79	7.51	2.29E-02
LINC01290	4.54E-01	3.94E-01	2.79	7.50	2.29E-02
OR4C3	4.54E-01	3.94E-01	2.79	7.49	2.29E-02
LINC01761	4.54E-01	3.93E-01	2.79	7.49	2.30E-02
LOC105379123	4.54E-01	3.93E-01	2.80	7.48	2.31E-02
NSUN4	4.54E-01	3.93E-01	2.80	7.48	2.31E-02
TNFSF12	4.54E-01	3.93E-01	2.80	7.47	2.31E-02
LINC01872	4.53E-01	3.93E-01	2.80	7.47	2.31E-02
LOC105377461	4.53E-01	3.93E-01	2.80	7.46	2.32E-02
LOC100129503	4.53E-01	3.93E-01	2.80	7.46	2.32E-02
LOC105378688	4.53E-01	3.92E-01	2.80	7.45	2.32E-02
ZNF460.AS1	4.53E-01	3.92E-01	2.80	7.45	2.33E-02
RBMY2EP	4.53E-01	3.92E-01	2.80	7.45	2.33E-02
LINC02408	4.53E-01	3.92E-01	2.80	7.45	2.33E-02
LOC107986359	4.53E-01	3.92E-01	2.80	7.44	2.33E-02

SNRPN	4.53E-01	3.92E-01	2.80	7.44	2.33E-02
LOC107987061	4.52E-01	3.92E-01	2.80	7.43	2.34E-02
ANKRD54	4.52E-01	3.91E-01	2.80	7.43	2.34E-02
MARS2	4.52E-01	3.91E-01	2.80	7.42	2.35E-02
TRPM7	4.51E-01	3.91E-01	2.80	7.41	2.35E-02
LOC105375716	4.51E-01	3.90E-01	2.80	7.40	2.36E-02
LOC107986887	4.51E-01	3.90E-01	2.80	7.40	2.36E-02
SLC18A3	4.51E-01	3.90E-01	2.80	7.40	2.36E-02
RASL11B	4.51E-01	3.90E-01	2.80	7.39	2.37E-02
CD300LD	4.51E-01	3.90E-01	2.80	7.39	2.37E-02
IGKV2D.29	4.51E-01	3.90E-01	2.80	7.39	2.37E-02
JUND	4.50E-01	3.89E-01	2.80	7.37	2.38E-02
SNORD13H	4.50E-01	3.89E-01	2.80	7.37	2.38E-02
MRPS36	4.50E-01	3.89E-01	2.80	7.37	2.38E-02
LOC101927890	4.50E-01	3.89E-01	2.80	7.37	2.38E-02
NBPF22P	4.50E-01	3.89E-01	2.81	7.36	2.39E-02
LOC105372511	4.50E-01	3.89E-01	2.81	7.36	2.39E-02
LOC105376345	4.50E-01	3.89E-01	2.81	7.36	2.39E-02
PIWIL2	4.50E-01	3.89E-01	2.81	7.36	2.39E-02
LOC107986897	4.50E-01	3.89E-01	2.81	7.36	2.39E-02
LOC105370964	4.49E-01	3.88E-01	2.81	7.35	2.40E-02
ERICH6B	4.49E-01	3.88E-01	2.81	7.34	2.41E-02
CTDSP2	4.49E-01	3.88E-01	2.81	7.33	2.41E-02
LOC101928168	4.49E-01	3.88E-01	2.81	7.33	2.41E-02
IGKV3D.11	4.48E-01	3.87E-01	2.81	7.31	2.42E-02
LOC107986962	4.48E-01	3.87E-01	2.81	7.31	2.43E-02
LILRA2	4.48E-01	3.87E-01	2.81	7.31	2.43E-02
SNORD126	4.48E-01	3.87E-01	2.81	7.31	2.43E-02
LOC105375875	4.48E-01	3.86E-01	2.81	7.30	2.43E-02
HS6ST2.AS1	4.48E-01	3.86E-01	2.81	7.29	2.44E-02
LOC105377235	4.48E-01	3.86E-01	2.81	7.29	2.44E-02
HBD	4.47E-01	3.86E-01	2.81	7.28	2.45E-02
IFNL2	4.47E-01	3.85E-01	2.81	7.27	2.45E-02
LOC105378323	4.46E-01	3.85E-01	2.81	7.26	2.46E-02
TCTA	4.46E-01	3.85E-01	2.81	7.25	2.47E-02
LRRC52.AS1	4.46E-01	3.85E-01	2.82	7.25	2.47E-02
RPS28	4.46E-01	3.84E-01	2.82	7.25	2.47E-02
LOC105374721	4.46E-01	3.84E-01	2.82	7.25	2.47E-02
LSM10	4.46E-01	3.84E-01	2.82	7.24	2.48E-02
RAB44	4.45E-01	3.84E-01	2.82	7.23	2.48E-02
MTMR3	4.45E-01	3.84E-01	2.82	7.23	2.49E-02

WASH3P_1	4.45E-01	3.84E-01	2.82	7.23	2.49E-02
CEP192	4.45E-01	3.83E-01	2.82	7.22	2.49E-02
MGC16275	4.45E-01	3.83E-01	2.82	7.21	2.50E-02
OCEL1	4.45E-01	3.83E-01	2.82	7.21	2.50E-02
PLP2	4.45E-01	3.83E-01	2.82	7.21	2.50E-02
LOC105376237	4.44E-01	3.83E-01	2.82	7.20	2.51E-02
LINC01916	4.44E-01	3.82E-01	2.82	7.19	2.51E-02
LOC644669	4.44E-01	3.82E-01	2.82	7.18	2.52E-02
TNKS2.AS1	4.44E-01	3.82E-01	2.82	7.18	2.52E-02
LOC107984670	4.44E-01	3.82E-01	2.82	7.18	2.52E-02
LOC100288123	4.44E-01	3.82E-01	2.82	7.18	2.53E-02
BPIFB2	4.44E-01	3.82E-01	2.82	7.17	2.53E-02
TOMM40L	4.44E-01	3.82E-01	2.82	7.17	2.53E-02
REPIN1	4.43E-01	3.82E-01	2.82	7.17	2.53E-02
GATA6.AS1	4.43E-01	3.81E-01	2.82	7.16	2.53E-02
NUDT9P1	4.43E-01	3.81E-01	2.82	7.16	2.54E-02
LOC100506446	4.43E-01	3.81E-01	2.82	7.16	2.54E-02
RPS16P5	4.43E-01	3.81E-01	2.82	7.15	2.54E-02
GAGE13	4.43E-01	3.81E-01	2.82	7.15	2.55E-02
ANKRD6	4.43E-01	3.81E-01	2.82	7.15	2.55E-02
LINC01350	4.42E-01	3.80E-01	2.82	7.14	2.56E-02
HERC2P2_2	4.42E-01	3.80E-01	2.83	7.13	2.56E-02
LOC107986959	4.42E-01	3.80E-01	2.83	7.13	2.56E-02
SNORA70B	4.42E-01	3.80E-01	2.83	7.13	2.56E-02
CSKMT	4.42E-01	3.80E-01	2.83	7.12	2.57E-02
TONSL.AS1	4.42E-01	3.80E-01	2.83	7.12	2.57E-02
LOC105378457	4.42E-01	3.79E-01	2.83	7.11	2.57E-02
IL20RA	4.41E-01	3.79E-01	2.83	7.11	2.58E-02
RLN2	4.41E-01	3.79E-01	2.83	7.10	2.58E-02
WNT2	4.41E-01	3.79E-01	2.83	7.10	2.59E-02
CASQ1	4.41E-01	3.79E-01	2.83	7.10	2.59E-02
COL26A1	4.41E-01	3.79E-01	2.83	7.09	2.59E-02
ZDHHC23	4.40E-01	3.78E-01	2.83	7.08	2.60E-02
LOC105369851	4.40E-01	3.78E-01	2.83	7.07	2.61E-02
LOC101927213	4.40E-01	3.78E-01	2.83	7.07	2.61E-02
TRQ.CTG1.4	4.40E-01	3.77E-01	2.83	7.06	2.61E-02
POLR2K	4.40E-01	3.77E-01	2.83	7.06	2.62E-02
FAM216B	4.40E-01	3.77E-01	2.83	7.06	2.62E-02
LOC102723339	4.40E-01	3.77E-01	2.83	7.06	2.62E-02
HSD52	4.40E-01	3.77E-01	2.83	7.06	2.62E-02
LOC107986597	4.39E-01	3.77E-01	2.83	7.06	2.62E-02

LOC105376197	4.39E-01	3.77E-01	2.83	7.05	2.62E-02
LOC112268042	4.39E-01	3.77E-01	2.83	7.05	2.62E-02
LINC01490	4.39E-01	3.77E-01	2.83	7.05	2.63E-02
LOC101929341	4.39E-01	3.77E-01	2.83	7.04	2.63E-02
TRG.AS1	4.39E-01	3.77E-01	2.83	7.04	2.63E-02
GET1	4.39E-01	3.77E-01	2.83	7.04	2.63E-02
YWHAEP7	4.39E-01	3.77E-01	2.83	7.04	2.63E-02
SDK1.AS1	4.39E-01	3.76E-01	2.83	7.04	2.63E-02
TMPRSS11F	4.39E-01	3.76E-01	2.83	7.03	2.64E-02
KLHDC7B.DT	4.39E-01	3.76E-01	2.83	7.03	2.64E-02
MIR4435.2HG	4.38E-01	3.76E-01	2.83	7.02	2.65E-02
LINC02626	4.38E-01	3.76E-01	2.84	7.02	2.65E-02
LOC105377879	4.38E-01	3.76E-01	2.84	7.02	2.65E-02
FOXN3.AS1	4.38E-01	3.76E-01	2.84	7.02	2.65E-02
LOC100132686	4.38E-01	3.76E-01	2.84	7.02	2.65E-02
MS4A18	4.38E-01	3.76E-01	2.84	7.02	2.65E-02
GTF3C2.AS1	4.38E-01	3.76E-01	2.84	7.02	2.65E-02
GAGE12B	4.38E-01	3.76E-01	2.84	7.02	2.65E-02
TBC1D3E	4.38E-01	3.75E-01	2.84	7.01	2.66E-02
LOC105374172	4.38E-01	3.75E-01	2.84	7.01	2.66E-02
OR4P4	4.38E-01	3.75E-01	2.84	7.00	2.67E-02
C8orf48	4.38E-01	3.75E-01	2.84	7.00	2.67E-02
LOC101927979	4.37E-01	3.75E-01	2.84	7.00	2.67E-02
LOC107986921	4.37E-01	3.75E-01	2.84	6.99	2.67E-02
MRPS31P5	4.37E-01	3.74E-01	2.84	6.99	2.68E-02
LGALS13	4.37E-01	3.74E-01	2.84	6.99	2.68E-02
LOC107987116	4.37E-01	3.74E-01	2.84	6.99	2.68E-02
TSPEAR.AS1	4.37E-01	3.74E-01	2.84	6.98	2.69E-02
LOC105370080	4.37E-01	3.74E-01	2.84	6.98	2.69E-02
TAGAP	4.37E-01	3.74E-01	2.84	6.98	2.69E-02
PIAS3	4.37E-01	3.74E-01	2.84	6.97	2.69E-02
LOC108783654	4.36E-01	3.74E-01	2.84	6.97	2.69E-02
TRAC	4.36E-01	3.74E-01	2.84	6.96	2.70E-02
SDC4	4.36E-01	3.73E-01	2.84	6.95	2.71E-02
IGLV3.29	4.36E-01	3.73E-01	2.84	6.95	2.71E-02
TRBV7.6_1	4.36E-01	3.73E-01	2.84	6.95	2.71E-02
CNTFR.AS1	4.35E-01	3.73E-01	2.84	6.94	2.72E-02
LOC101929551	4.35E-01	3.73E-01	2.84	6.94	2.72E-02
ADGRE4P	4.35E-01	3.72E-01	2.84	6.92	2.73E-02
LOC105378327	4.34E-01	3.72E-01	2.84	6.91	2.74E-02
RPL24	4.34E-01	3.72E-01	2.84	6.91	2.74E-02

LOC105371901	4.34E-01	3.71E-01	2.84	6.91	2.74E-02
COL28A1	4.34E-01	3.71E-01	2.85	6.90	2.75E-02
GPR176	4.34E-01	3.71E-01	2.85	6.90	2.75E-02
TM2D1	4.34E-01	3.71E-01	2.85	6.89	2.76E-02
LOC105377055	4.34E-01	3.71E-01	2.85	6.89	2.76E-02
LOC100506016	4.33E-01	3.71E-01	2.85	6.89	2.76E-02
LOC105377437	4.33E-01	3.70E-01	2.85	6.88	2.77E-02
LOC105379329	4.33E-01	3.70E-01	2.85	6.88	2.77E-02
LINC02206	4.33E-01	3.70E-01	2.85	6.88	2.77E-02
LOC105369960	4.33E-01	3.70E-01	2.85	6.88	2.77E-02
LOC105370873	4.33E-01	3.70E-01	2.85	6.87	2.77E-02
CARD9	4.33E-01	3.70E-01	2.85	6.87	2.78E-02
PDGFRL	4.32E-01	3.69E-01	2.85	6.85	2.79E-02
OR1D5	4.32E-01	3.69E-01	2.85	6.85	2.80E-02
MIR548F2	4.32E-01	3.68E-01	2.85	6.83	2.81E-02
CYB561D2	4.31E-01	3.68E-01	2.85	6.83	2.81E-02
CRYGS	4.31E-01	3.68E-01	2.85	6.83	2.81E-02
WDR13	4.31E-01	3.68E-01	2.85	6.83	2.81E-02
LOC107984770	4.31E-01	3.68E-01	2.85	6.82	2.82E-02
LOC107984369	4.31E-01	3.68E-01	2.85	6.82	2.82E-02
LOC105377762	4.31E-01	3.68E-01	2.85	6.82	2.82E-02
LOC105370350	4.31E-01	3.68E-01	2.85	6.82	2.82E-02
LOC107984650	4.31E-01	3.68E-01	2.85	6.81	2.83E-02
MIR181A1	4.31E-01	3.68E-01	2.85	6.81	2.83E-02
CXCL10	4.31E-01	3.67E-01	2.85	6.81	2.83E-02
BARX1.DT	4.31E-01	3.67E-01	2.85	6.81	2.83E-02
KATNBL1P6	4.31E-01	3.67E-01	2.85	6.81	2.83E-02
LINC02521	4.31E-01	3.67E-01	2.85	6.81	2.83E-02
BRPF1	4.30E-01	3.67E-01	2.86	6.79	2.85E-02
RFX2	4.30E-01	3.66E-01	2.86	6.78	2.85E-02
LINC01358	4.30E-01	3.66E-01	2.86	6.78	2.85E-02
LINC00635	4.30E-01	3.66E-01	2.86	6.78	2.85E-02
LOC105369658	4.30E-01	3.66E-01	2.86	6.78	2.86E-02
IGHA2	4.29E-01	3.66E-01	2.86	6.78	2.86E-02
SNORD65C	4.29E-01	3.66E-01	2.86	6.77	2.86E-02
LOC105376302	4.29E-01	3.66E-01	2.86	6.77	2.86E-02
LZTFL1	4.29E-01	3.66E-01	2.86	6.77	2.86E-02
CCT6P1	4.29E-01	3.66E-01	2.86	6.77	2.86E-02
TSPAN33	4.29E-01	3.66E-01	2.86	6.77	2.87E-02
GOLGA2P10	4.29E-01	3.66E-01	2.86	6.77	2.87E-02
ART1	4.29E-01	3.66E-01	2.86	6.76	2.87E-02

LOC105378098	4.29E-01	3.65E-01	2.86	6.76	2.87E-02
RPS9	4.29E-01	3.65E-01	2.86	6.76	2.88E-02
RABL2B	4.29E-01	3.65E-01	2.86	6.75	2.88E-02
DPPA2P3	4.29E-01	3.65E-01	2.86	6.75	2.88E-02
MIR6130	4.29E-01	3.65E-01	2.86	6.75	2.88E-02
LOC107985279	4.29E-01	3.65E-01	2.86	6.75	2.88E-02
AZGP1P1	4.28E-01	3.65E-01	2.86	6.75	2.89E-02
PYGO2	4.28E-01	3.65E-01	2.86	6.74	2.89E-02
LOC112268214	4.28E-01	3.64E-01	2.86	6.73	2.90E-02
LOC107985551	4.28E-01	3.64E-01	2.86	6.72	2.91E-02
CCDC140	4.27E-01	3.64E-01	2.86	6.72	2.91E-02
GPD2	4.27E-01	3.64E-01	2.86	6.72	2.91E-02
LOC285422	4.27E-01	3.64E-01	2.86	6.71	2.92E-02
LOC285804	4.27E-01	3.63E-01	2.86	6.71	2.92E-02
LOC105379149	4.27E-01	3.63E-01	2.86	6.71	2.92E-02
LOC105375462	4.27E-01	3.63E-01	2.86	6.71	2.92E-02
KRTAP19.5	4.27E-01	3.63E-01	2.86	6.70	2.93E-02
LOC105370982	4.26E-01	3.62E-01	2.87	6.69	2.94E-02
LOC105372117	4.26E-01	3.62E-01	2.87	6.68	2.95E-02
LOC105371818	4.26E-01	3.62E-01	2.87	6.68	2.95E-02
EID1	4.26E-01	3.62E-01	2.87	6.67	2.95E-02
EXOSC7	4.26E-01	3.62E-01	2.87	6.67	2.96E-02
LOC107984358	4.25E-01	3.62E-01	2.87	6.66	2.96E-02
LOC101930071	4.25E-01	3.61E-01	2.87	6.66	2.97E-02
GTPBP3	4.25E-01	3.61E-01	2.87	6.65	2.97E-02
TRAV12.1	4.25E-01	3.61E-01	2.87	6.64	2.98E-02
LINC00244	4.24E-01	3.60E-01	2.87	6.64	2.99E-02
LOC105374986	4.24E-01	3.60E-01	2.87	6.63	2.99E-02
CDYL.AS1	4.24E-01	3.60E-01	2.87	6.63	3.00E-02
RIOK1	4.24E-01	3.60E-01	2.87	6.63	3.00E-02
PRPSAP2	4.24E-01	3.60E-01	2.87	6.63	3.00E-02
UBE2NL	4.24E-01	3.60E-01	2.87	6.62	3.00E-02
ACACA_1	4.24E-01	3.60E-01	2.87	6.62	3.00E-02
LOC105379194	4.24E-01	3.60E-01	2.87	6.62	3.01E-02
SCARF2	4.24E-01	3.60E-01	2.87	6.62	3.01E-02
NOA1	4.24E-01	3.59E-01	2.87	6.61	3.01E-02
DVL1	4.23E-01	3.59E-01	2.87	6.61	3.02E-02
CBX8	4.23E-01	3.59E-01	2.87	6.60	3.02E-02
LOC107986920	4.23E-01	3.59E-01	2.87	6.60	3.02E-02
LOC105377932	4.23E-01	3.59E-01	2.87	6.60	3.02E-02
LOC105376586	4.23E-01	3.59E-01	2.87	6.60	3.02E-02

FBXO48	4.22E-01	3.58E-01	2.87	6.58	3.04E-02
LOC107984169	4.22E-01	3.58E-01	2.88	6.57	3.05E-02
RFC5	4.22E-01	3.57E-01	2.88	6.56	3.06E-02
LOC105369318	4.22E-01	3.57E-01	2.88	6.56	3.06E-02
MYOSLID.AS1	4.21E-01	3.57E-01	2.88	6.55	3.07E-02
RP9P	4.21E-01	3.57E-01	2.88	6.55	3.07E-02
IGLVI.70	4.21E-01	3.57E-01	2.88	6.54	3.08E-02
PKMYT1	4.21E-01	3.56E-01	2.88	6.54	3.08E-02
FCGBP	4.21E-01	3.56E-01	2.88	6.54	3.08E-02
LOC400710	4.21E-01	3.56E-01	2.88	6.53	3.09E-02
TBXT	4.20E-01	3.56E-01	2.88	6.53	3.09E-02
UBTD1	4.20E-01	3.56E-01	2.88	6.52	3.10E-02
LOC105374798	4.20E-01	3.55E-01	2.88	6.51	3.11E-02
PEX11G	4.20E-01	3.55E-01	2.88	6.51	3.12E-02
LOC105370674	4.19E-01	3.55E-01	2.88	6.50	3.12E-02
LOC105376035	4.19E-01	3.55E-01	2.88	6.50	3.12E-02
DHX37	4.19E-01	3.55E-01	2.88	6.50	3.13E-02
UGT1A3	4.19E-01	3.54E-01	2.88	6.49	3.13E-02
SON	4.19E-01	3.54E-01	2.88	6.49	3.13E-02
CENPT	4.19E-01	3.54E-01	2.88	6.49	3.14E-02
LOC105373966	4.19E-01	3.54E-01	2.88	6.48	3.14E-02
LINC02587	4.18E-01	3.53E-01	2.89	6.47	3.15E-02
PANCR	4.18E-01	3.53E-01	2.89	6.47	3.16E-02
LOC105375689	4.18E-01	3.53E-01	2.89	6.45	3.17E-02
LOC107986938	4.18E-01	3.53E-01	2.89	6.45	3.17E-02
ZNF321P	4.17E-01	3.53E-01	2.89	6.45	3.17E-02
FGF12.AS1	4.17E-01	3.53E-01	2.89	6.45	3.17E-02
TRG.CCC2.1	4.17E-01	3.53E-01	2.89	6.45	3.18E-02
LOC100128253	4.17E-01	3.53E-01	2.89	6.44	3.18E-02
XKR4	4.17E-01	3.52E-01	2.89	6.44	3.19E-02
FCRL1	4.17E-01	3.52E-01	2.89	6.43	3.19E-02
LOC112267888	4.17E-01	3.52E-01	2.89	6.43	3.19E-02
LINC00412	4.17E-01	3.52E-01	2.89	6.43	3.20E-02
LINC02757	4.16E-01	3.52E-01	2.89	6.42	3.20E-02
MIR2276	4.16E-01	3.51E-01	2.89	6.41	3.21E-02
LOC105369811	4.16E-01	3.51E-01	2.89	6.41	3.22E-02
LOC105375272	4.16E-01	3.51E-01	2.89	6.40	3.22E-02
LINC01221	4.16E-01	3.51E-01	2.89	6.40	3.23E-02
TBPL2	4.15E-01	3.50E-01	2.89	6.40	3.23E-02
NKAPP1	4.15E-01	3.50E-01	2.89	6.40	3.23E-02
TMEM99	4.15E-01	3.50E-01	2.89	6.39	3.23E-02

SLC38A5	4.15E-01	3.50E-01	2.89	6.39	3.23E-02
LOC105375772	4.15E-01	3.50E-01	2.89	6.39	3.24E-02
LOC112268413	4.15E-01	3.50E-01	2.89	6.39	3.24E-02
LOC105377459	4.15E-01	3.50E-01	2.89	6.39	3.24E-02
PMEL	4.15E-01	3.50E-01	2.89	6.38	3.24E-02
SLC29A3	4.15E-01	3.50E-01	2.89	6.38	3.25E-02
CSRP1	4.15E-01	3.50E-01	2.89	6.38	3.25E-02
HLA.C_3	4.14E-01	3.49E-01	2.89	6.37	3.26E-02
PRAP1	4.14E-01	3.49E-01	2.89	6.37	3.26E-02
MIR8086	4.14E-01	3.49E-01	2.89	6.37	3.26E-02
GFOD2	4.14E-01	3.49E-01	2.89	6.37	3.26E-02
UBE2D1	4.14E-01	3.49E-01	2.89	6.36	3.26E-02
DDX27	4.14E-01	3.49E-01	2.90	6.36	3.27E-02
RSPH1	4.14E-01	3.49E-01	2.90	6.36	3.27E-02
C6orf120	4.14E-01	3.49E-01	2.90	6.35	3.27E-02
HSD11B2	4.14E-01	3.49E-01	2.90	6.35	3.28E-02
LOC101929653	4.14E-01	3.48E-01	2.90	6.35	3.28E-02
PMVK	4.14E-01	3.48E-01	2.90	6.35	3.28E-02
LINC02607	4.13E-01	3.48E-01	2.90	6.35	3.28E-02
SETD1A	4.13E-01	3.48E-01	2.90	6.34	3.28E-02
RNF217.AS1	4.13E-01	3.48E-01	2.90	6.34	3.29E-02
LOC105375879	4.13E-01	3.48E-01	2.90	6.33	3.29E-02
CCDC180	4.13E-01	3.48E-01	2.90	6.33	3.30E-02
NUP62	4.13E-01	3.48E-01	2.90	6.33	3.30E-02
RPS26P11	4.13E-01	3.48E-01	2.90	6.33	3.30E-02
LOC105371279	4.13E-01	3.47E-01	2.90	6.32	3.31E-02
TNFRSF10B	4.12E-01	3.47E-01	2.90	6.31	3.32E-02
LANCL3	4.12E-01	3.47E-01	2.90	6.31	3.32E-02
LOC102724463	4.12E-01	3.47E-01	2.90	6.31	3.32E-02
CRYZL2P.SEC16B	4.12E-01	3.46E-01	2.90	6.30	3.33E-02
PPP3CB.AS1	4.11E-01	3.46E-01	2.90	6.29	3.35E-02
LOC105374591	4.11E-01	3.46E-01	2.90	6.28	3.35E-02
CSF2	4.11E-01	3.45E-01	2.90	6.27	3.36E-02
LOC107985235	4.11E-01	3.45E-01	2.90	6.27	3.36E-02
LOC101927359	4.11E-01	3.45E-01	2.90	6.27	3.36E-02
TCEAL5	4.11E-01	3.45E-01	2.90	6.27	3.36E-02
LOC105378512	4.11E-01	3.45E-01	2.90	6.27	3.36E-02
NBPF7	4.10E-01	3.45E-01	2.91	6.26	3.38E-02
CTCF	4.10E-01	3.44E-01	2.91	6.25	3.39E-02
SLC33A1	4.10E-01	3.44E-01	2.91	6.24	3.39E-02
LOC105376070	4.10E-01	3.44E-01	2.91	6.24	3.40E-02

LOC105369149	4.09E-01	3.44E-01	2.91	6.24	3.40E-02
LOC105370846	4.09E-01	3.43E-01	2.91	6.23	3.41E-02
MAN2A1	4.09E-01	3.43E-01	2.91	6.23	3.41E-02
LOC107985981	4.09E-01	3.43E-01	2.91	6.23	3.41E-02
LOC105373075	4.09E-01	3.43E-01	2.91	6.23	3.41E-02
LOC107986843	4.09E-01	3.43E-01	2.91	6.22	3.42E-02
LOC107987027	4.09E-01	3.43E-01	2.91	6.22	3.42E-02
RRP12	4.09E-01	3.43E-01	2.91	6.22	3.42E-02
TRNG	4.08E-01	3.42E-01	2.91	6.21	3.43E-02
LOC105375481	4.08E-01	3.42E-01	2.91	6.21	3.44E-02
LINC00871_1	4.08E-01	3.42E-01	2.91	6.20	3.44E-02
SH2D7	4.08E-01	3.42E-01	2.91	6.20	3.44E-02
TRPV6_1	4.08E-01	3.42E-01	2.91	6.20	3.44E-02
LOC105372770	4.08E-01	3.42E-01	2.91	6.19	3.45E-02
MRPS30.DT	4.08E-01	3.42E-01	2.91	6.19	3.45E-02
MRI1	4.07E-01	3.42E-01	2.91	6.19	3.46E-02
HAGHL	4.07E-01	3.41E-01	2.91	6.18	3.46E-02
NAGK	4.07E-01	3.41E-01	2.91	6.18	3.47E-02
LAMC1.AS1	4.07E-01	3.41E-01	2.91	6.18	3.47E-02
ADGRF5.AS1	4.07E-01	3.41E-01	2.91	6.17	3.47E-02
LOC105375672	4.07E-01	3.41E-01	2.91	6.17	3.48E-02
LOC105373743	4.06E-01	3.41E-01	2.91	6.16	3.48E-02
LOC107984214	4.06E-01	3.40E-01	2.91	6.16	3.49E-02
LOC105371414	4.06E-01	3.40E-01	2.91	6.16	3.49E-02
LOC105376030	4.06E-01	3.40E-01	2.91	6.16	3.49E-02
EMILIN1	4.06E-01	3.40E-01	2.91	6.16	3.49E-02
LOC101927452	4.06E-01	3.40E-01	2.91	6.16	3.49E-02
TTLL4	4.06E-01	3.40E-01	2.91	6.16	3.49E-02
LOC105374616	4.06E-01	3.40E-01	2.92	6.15	3.50E-02
SH3BGRL	4.06E-01	3.40E-01	2.92	6.14	3.51E-02
TMEM191B	4.06E-01	3.40E-01	2.92	6.14	3.51E-02
SNHG17	4.06E-01	3.40E-01	2.92	6.14	3.51E-02
GCC2.AS1	4.06E-01	3.40E-01	2.92	6.14	3.51E-02
LOC105370789	4.05E-01	3.39E-01	2.92	6.14	3.52E-02
LINC01798	4.05E-01	3.38E-01	2.92	6.11	3.54E-02
MIR2392	4.04E-01	3.38E-01	2.92	6.10	3.56E-02
FBXL3	4.04E-01	3.38E-01	2.92	6.10	3.56E-02
PYCR1	4.04E-01	3.37E-01	2.92	6.09	3.57E-02
AACSP1	4.04E-01	3.37E-01	2.92	6.09	3.57E-02
LINC01232	4.03E-01	3.37E-01	2.92	6.09	3.57E-02
LOC100506675	4.03E-01	3.37E-01	2.92	6.09	3.57E-02

LOC105370563	4.03E-01	3.37E-01	2.92	6.09	3.57E-02
LOC107986662	4.03E-01	3.37E-01	2.92	6.09	3.57E-02
LOC107984374	4.03E-01	3.37E-01	2.92	6.09	3.58E-02
LOC105374952	4.03E-01	3.37E-01	2.92	6.08	3.58E-02
LOC105373211	4.03E-01	3.37E-01	2.92	6.08	3.58E-02
LOC105379179	4.03E-01	3.37E-01	2.92	6.08	3.59E-02
LOC107984362	4.03E-01	3.36E-01	2.92	6.07	3.60E-02
LOC107985699	4.03E-01	3.36E-01	2.92	6.07	3.60E-02
RABGAP1L	4.03E-01	3.36E-01	2.92	6.06	3.60E-02
LAMB2P1	4.02E-01	3.36E-01	2.92	6.06	3.60E-02
CCL8	4.02E-01	3.36E-01	2.92	6.06	3.61E-02
TRNP	4.02E-01	3.36E-01	2.92	6.06	3.61E-02
LOC105371732	4.02E-01	3.36E-01	2.92	6.06	3.61E-02
SLC25A32	4.02E-01	3.36E-01	2.92	6.06	3.61E-02
SMPD5	4.02E-01	3.36E-01	2.92	6.05	3.61E-02
LINC00851	4.02E-01	3.36E-01	2.92	6.05	3.61E-02
RBM38.AS1	4.02E-01	3.35E-01	2.93	6.05	3.62E-02
MIR620	4.01E-01	3.35E-01	2.93	6.04	3.63E-02
KRT24	4.01E-01	3.35E-01	2.93	6.04	3.63E-02
LOC105372448	4.01E-01	3.35E-01	2.93	6.03	3.64E-02
CREBZF	4.01E-01	3.35E-01	2.93	6.03	3.64E-02
LOC107986969	4.01E-01	3.35E-01	2.93	6.03	3.64E-02
LOC105378864	4.01E-01	3.35E-01	2.93	6.03	3.64E-02
LOC105372862	4.01E-01	3.35E-01	2.93	6.03	3.65E-02
LOC105374052	4.01E-01	3.34E-01	2.93	6.02	3.66E-02
NDUFC2	4.01E-01	3.34E-01	2.93	6.02	3.66E-02
LOC105378658	4.00E-01	3.34E-01	2.93	6.01	3.67E-02
LOC105377971	4.00E-01	3.34E-01	2.93	6.01	3.67E-02
IMP3	4.00E-01	3.34E-01	2.93	6.01	3.67E-02
PPP4R2	4.00E-01	3.34E-01	2.93	6.01	3.67E-02
LOC100653049	4.00E-01	3.33E-01	2.93	6.00	3.68E-02
LOC105375845	4.00E-01	3.33E-01	2.93	6.00	3.68E-02
LOC105373985	4.00E-01	3.33E-01	2.93	6.00	3.68E-02
KRT34_1	4.00E-01	3.33E-01	2.93	5.99	3.69E-02
VMO1	4.00E-01	3.33E-01	2.93	5.99	3.69E-02
OR7C1	3.99E-01	3.33E-01	2.93	5.99	3.70E-02
LOC101929418	3.99E-01	3.33E-01	2.93	5.98	3.70E-02
LACTBL1	3.99E-01	3.33E-01	2.93	5.98	3.70E-02
MYOM3.AS1	3.99E-01	3.32E-01	2.93	5.98	3.70E-02
RAVER2	3.99E-01	3.32E-01	2.93	5.98	3.71E-02
DHRS2	3.99E-01	3.32E-01	2.93	5.98	3.71E-02

LOC105369302	3.99E-01	3.32E-01	2.93	5.97	3.71E-02
LOC105374096	3.99E-01	3.32E-01	2.93	5.97	3.71E-02
OR2M4	3.99E-01	3.32E-01	2.93	5.97	3.71E-02
LOC105373283	3.99E-01	3.32E-01	2.93	5.97	3.71E-02
KLK15	3.99E-01	3.32E-01	2.93	5.97	3.71E-02
LOC112268471	3.99E-01	3.32E-01	2.93	5.97	3.72E-02
TAF9B	3.99E-01	3.32E-01	2.93	5.97	3.72E-02
C6orf99	3.99E-01	3.32E-01	2.93	5.96	3.72E-02
EFEMP1	3.99E-01	3.32E-01	2.93	5.96	3.73E-02
LINC01512	3.98E-01	3.32E-01	2.93	5.96	3.73E-02
MIR5695	3.98E-01	3.32E-01	2.93	5.96	3.73E-02
NCOA5	3.98E-01	3.32E-01	2.93	5.96	3.73E-02
ZNF474	3.98E-01	3.32E-01	2.93	5.96	3.73E-02
TRGV11	3.98E-01	3.31E-01	2.93	5.95	3.74E-02
LOC107984379	3.98E-01	3.31E-01	2.93	5.95	3.74E-02
LOC105377927	3.98E-01	3.31E-01	2.94	5.94	3.75E-02
LINC02112	3.97E-01	3.31E-01	2.94	5.94	3.76E-02
LOC105372757	3.97E-01	3.30E-01	2.94	5.94	3.76E-02
TSPAN5	3.97E-01	3.30E-01	2.94	5.93	3.76E-02
KCTD18	3.97E-01	3.30E-01	2.94	5.93	3.76E-02
LOC105369783	3.97E-01	3.30E-01	2.94	5.92	3.78E-02
LOC107984778	3.96E-01	3.29E-01	2.94	5.91	3.79E-02
INKA2	3.96E-01	3.29E-01	2.94	5.91	3.80E-02
LOC107986148	3.96E-01	3.29E-01	2.94	5.90	3.80E-02
DPP4.DT	3.96E-01	3.29E-01	2.94	5.90	3.81E-02
LOC107985021	3.96E-01	3.29E-01	2.94	5.90	3.81E-02
SLC25A45	3.96E-01	3.29E-01	2.94	5.90	3.81E-02
LOC105374901	3.96E-01	3.29E-01	2.94	5.90	3.81E-02
LOC107986179	3.96E-01	3.29E-01	2.94	5.89	3.81E-02
DPT	3.96E-01	3.29E-01	2.94	5.89	3.81E-02
RBM3	3.96E-01	3.28E-01	2.94	5.89	3.82E-02
HSPC324	3.96E-01	3.28E-01	2.94	5.89	3.82E-02
LOC107984590	3.95E-01	3.28E-01	2.94	5.89	3.82E-02
BLID	3.95E-01	3.28E-01	2.94	5.88	3.83E-02
LINC01903	3.95E-01	3.28E-01	2.94	5.88	3.83E-02
LINC02316	3.95E-01	3.28E-01	2.94	5.88	3.83E-02
ILF2	3.95E-01	3.28E-01	2.94	5.88	3.83E-02
LOC107986105	3.95E-01	3.28E-01	2.94	5.88	3.84E-02
RHCE	3.95E-01	3.27E-01	2.94	5.87	3.84E-02
GPR32	3.95E-01	3.27E-01	2.94	5.87	3.85E-02
LOC105378416	3.94E-01	3.27E-01	2.94	5.86	3.86E-02

LOC102724194	3.94E-01	3.27E-01	2.94	5.85	3.87E-02
FAM247D	3.94E-01	3.27E-01	2.94	5.85	3.87E-02
LOC105377495	3.94E-01	3.26E-01	2.95	5.84	3.88E-02
LOC440313	3.94E-01	3.26E-01	2.95	5.84	3.88E-02
MGC15885	3.93E-01	3.26E-01	2.95	5.84	3.88E-02
ECI2	3.93E-01	3.26E-01	2.95	5.84	3.88E-02
LOC105379393	3.93E-01	3.26E-01	2.95	5.84	3.89E-02
LOC102724700	3.93E-01	3.26E-01	2.95	5.84	3.89E-02
NUDT18	3.93E-01	3.26E-01	2.95	5.83	3.90E-02
TAS2R50_2	3.93E-01	3.25E-01	2.95	5.82	3.91E-02
LOC107987304	3.93E-01	3.25E-01	2.95	5.82	3.91E-02
LOC105374643	3.93E-01	3.25E-01	2.95	5.82	3.91E-02
LINC01893	3.93E-01	3.25E-01	2.95	5.82	3.91E-02
AFDN	3.93E-01	3.25E-01	2.95	5.81	3.92E-02
VPS9D1.AS1	3.92E-01	3.25E-01	2.95	5.81	3.92E-02
LOC107985809	3.92E-01	3.25E-01	2.95	5.81	3.93E-02
LOC102467217	3.92E-01	3.25E-01	2.95	5.80	3.93E-02
TRT.AGT4.1	3.92E-01	3.24E-01	2.95	5.80	3.94E-02
LINC02557_1	3.92E-01	3.24E-01	2.95	5.79	3.94E-02
MIR210HG	3.92E-01	3.24E-01	2.95	5.79	3.94E-02
EML2.AS1	3.92E-01	3.24E-01	2.95	5.79	3.94E-02
CCDC107	3.92E-01	3.24E-01	2.95	5.79	3.95E-02
LINC01711	3.92E-01	3.24E-01	2.95	5.79	3.95E-02
MTHFD2P1	3.92E-01	3.24E-01	2.95	5.79	3.95E-02
MIR1.1HG	3.91E-01	3.24E-01	2.95	5.79	3.95E-02
LINC02442	3.91E-01	3.24E-01	2.95	5.79	3.95E-02
LINC02260	3.91E-01	3.24E-01	2.95	5.79	3.95E-02
LOC100287944	3.91E-01	3.24E-01	2.95	5.79	3.95E-02
LOC105373644	3.91E-01	3.24E-01	2.95	5.78	3.96E-02
LINC02633	3.91E-01	3.23E-01	2.95	5.78	3.96E-02
LOC105373347	3.91E-01	3.23E-01	2.95	5.78	3.97E-02
LOC105379314	3.91E-01	3.23E-01	2.95	5.77	3.97E-02
HAUS2	3.90E-01	3.23E-01	2.95	5.76	3.98E-02
C8G	3.90E-01	3.22E-01	2.95	5.76	4.00E-02
LINC00265	3.90E-01	3.22E-01	2.95	5.75	4.00E-02
GS1.279B7.1	3.90E-01	3.22E-01	2.95	5.75	4.00E-02
SNORD1A	3.90E-01	3.22E-01	2.95	5.75	4.00E-02
GOLGA8EP	3.90E-01	3.22E-01	2.95	5.75	4.00E-02
LOC105376428	3.90E-01	3.22E-01	2.95	5.75	4.01E-02
NIP7	3.90E-01	3.22E-01	2.95	5.75	4.01E-02
LOC105377697	3.90E-01	3.22E-01	2.95	5.75	4.01E-02

FUZ	3.89E-01	3.22E-01	2.96	5.74	4.02E-02
TAS1R1	3.89E-01	3.22E-01	2.96	5.74	4.02E-02
MYH7	3.89E-01	3.22E-01	2.96	5.74	4.02E-02
IRGM	3.89E-01	3.21E-01	2.96	5.74	4.02E-02
PLSCR5	3.89E-01	3.21E-01	2.96	5.74	4.02E-02
SNORD114.4	3.89E-01	3.21E-01	2.96	5.74	4.02E-02
LOC105374681	3.89E-01	3.21E-01	2.96	5.73	4.02E-02
CASC19	3.89E-01	3.21E-01	2.96	5.73	4.03E-02
PPP4R3B	3.89E-01	3.21E-01	2.96	5.72	4.04E-02
LINC02216	3.88E-01	3.20E-01	2.96	5.72	4.05E-02
FRY	3.88E-01	3.20E-01	2.96	5.71	4.05E-02
CHRNA3	3.88E-01	3.20E-01	2.96	5.71	4.06E-02
KAT7	3.88E-01	3.20E-01	2.96	5.71	4.06E-02
LOC105369725	3.88E-01	3.20E-01	2.96	5.71	4.06E-02
IGLVI.63	3.88E-01	3.20E-01	2.96	5.70	4.07E-02
LINC00921	3.88E-01	3.20E-01	2.96	5.70	4.07E-02
HEXA	3.88E-01	3.20E-01	2.96	5.70	4.08E-02
ZNF564	3.88E-01	3.20E-01	2.96	5.70	4.08E-02
PRKCZ.AS1	3.87E-01	3.19E-01	2.96	5.69	4.08E-02
LOC105377703	3.87E-01	3.19E-01	2.96	5.69	4.08E-02
LOC101928782	3.87E-01	3.19E-01	2.96	5.69	4.09E-02
CTLA4	3.87E-01	3.19E-01	2.96	5.69	4.09E-02
LOC105370262	3.87E-01	3.19E-01	2.96	5.69	4.09E-02
LOC101409256	3.87E-01	3.19E-01	2.96	5.68	4.09E-02
LINC02728	3.87E-01	3.19E-01	2.96	5.68	4.10E-02
PNPLA5	3.87E-01	3.19E-01	2.96	5.68	4.10E-02
LOC105377469	3.87E-01	3.19E-01	2.96	5.68	4.10E-02
LOC107985305	3.87E-01	3.19E-01	2.96	5.67	4.11E-02
C14orf28	3.86E-01	3.18E-01	2.96	5.67	4.12E-02
LOC105373855	3.86E-01	3.18E-01	2.96	5.66	4.12E-02
LOC105369808	3.86E-01	3.18E-01	2.96	5.66	4.12E-02
BMPR1A	3.86E-01	3.18E-01	2.96	5.66	4.13E-02
OR14J1_5	3.86E-01	3.18E-01	2.96	5.66	4.13E-02
LINC.PINT	3.86E-01	3.18E-01	2.96	5.66	4.14E-02
LINC00884	3.86E-01	3.18E-01	2.96	5.65	4.14E-02
DPY19L2	3.86E-01	3.17E-01	2.96	5.65	4.14E-02
LOC105378626	3.86E-01	3.17E-01	2.96	5.65	4.14E-02
MACIR	3.86E-01	3.17E-01	2.97	5.65	4.15E-02
CASC22	3.85E-01	3.17E-01	2.97	5.64	4.15E-02
CMAHP	3.85E-01	3.17E-01	2.97	5.64	4.15E-02
NBPF15	3.85E-01	3.17E-01	2.97	5.64	4.16E-02

ELOVL2.AS1	3.85E-01	3.17E-01	2.97	5.64	4.16E-02
LOC105374009	3.85E-01	3.17E-01	2.97	5.64	4.16E-02
FZD5	3.85E-01	3.17E-01	2.97	5.64	4.16E-02
CABP2	3.85E-01	3.17E-01	2.97	5.64	4.16E-02
DSPP	3.85E-01	3.17E-01	2.97	5.63	4.17E-02
RGS19	3.85E-01	3.16E-01	2.97	5.63	4.17E-02
SDHB	3.85E-01	3.16E-01	2.97	5.63	4.18E-02
LINC01031	3.84E-01	3.16E-01	2.97	5.62	4.18E-02
LOC105372004	3.84E-01	3.16E-01	2.97	5.62	4.19E-02
LOC107985437	3.84E-01	3.16E-01	2.97	5.61	4.20E-02
KHSRP	3.84E-01	3.15E-01	2.97	5.61	4.21E-02
LOC105377796	3.84E-01	3.15E-01	2.97	5.60	4.21E-02
LINC02050	3.84E-01	3.15E-01	2.97	5.60	4.21E-02
LOC107987004	3.84E-01	3.15E-01	2.97	5.60	4.21E-02
MAPK8IP1P2	3.84E-01	3.15E-01	2.97	5.60	4.21E-02
ST6GALNAC6	3.84E-01	3.15E-01	2.97	5.60	4.22E-02
ZMAT3	3.83E-01	3.15E-01	2.97	5.60	4.22E-02
HIGD1A	3.83E-01	3.15E-01	2.97	5.60	4.22E-02
C2CD4D.AS1	3.83E-01	3.15E-01	2.97	5.59	4.23E-02
SUPT4H1	3.83E-01	3.15E-01	2.97	5.59	4.23E-02
HMGB1	3.83E-01	3.15E-01	2.97	5.59	4.23E-02
LINC01136	3.83E-01	3.15E-01	2.97	5.59	4.23E-02
LOC105379355	3.83E-01	3.15E-01	2.97	5.59	4.23E-02
TLR4	3.83E-01	3.14E-01	2.97	5.58	4.25E-02
LOC107984932	3.83E-01	3.14E-01	2.97	5.58	4.25E-02
TRIM67.AS1	3.82E-01	3.14E-01	2.97	5.57	4.25E-02
RAB27B	3.82E-01	3.14E-01	2.97	5.57	4.26E-02
LOC105371312	3.82E-01	3.14E-01	2.97	5.57	4.26E-02
CZIB	3.82E-01	3.14E-01	2.97	5.57	4.26E-02
PCDHGB6	3.82E-01	3.14E-01	2.97	5.57	4.26E-02
CNIH4	3.82E-01	3.13E-01	2.97	5.57	4.27E-02
MS4A3	3.82E-01	3.13E-01	2.97	5.55	4.28E-02
BHLHE40.AS1	3.82E-01	3.13E-01	2.97	5.55	4.29E-02
TRAF7	3.82E-01	3.13E-01	2.97	5.55	4.29E-02
C22orf31	3.81E-01	3.13E-01	2.98	5.55	4.29E-02
LOC112268254	3.81E-01	3.13E-01	2.98	5.55	4.30E-02
LRRC4	3.81E-01	3.12E-01	2.98	5.54	4.30E-02
SLC4A9	3.81E-01	3.12E-01	2.98	5.54	4.30E-02
LUC7L	3.81E-01	3.12E-01	2.98	5.54	4.30E-02
LOC105376105	3.81E-01	3.12E-01	2.98	5.54	4.31E-02
PAGE5	3.81E-01	3.12E-01	2.98	5.54	4.31E-02

NGLY1	3.81E-01	3.12E-01	2.98	5.54	4.31E-02
TMEM181	3.81E-01	3.12E-01	2.98	5.54	4.31E-02
PHF24	3.81E-01	3.12E-01	2.98	5.54	4.31E-02
LINC01632	3.81E-01	3.12E-01	2.98	5.53	4.31E-02
TEX36.AS1	3.81E-01	3.12E-01	2.98	5.53	4.31E-02
MIR563	3.81E-01	3.12E-01	2.98	5.53	4.32E-02
ZNF733P	3.81E-01	3.12E-01	2.98	5.53	4.32E-02
ETDC	3.81E-01	3.12E-01	2.98	5.53	4.32E-02
HSPA1L	3.81E-01	3.12E-01	2.98	5.53	4.32E-02
TCF15	3.80E-01	3.12E-01	2.98	5.53	4.32E-02
LINC02852	3.80E-01	3.12E-01	2.98	5.52	4.33E-02
GPR156	3.80E-01	3.11E-01	2.98	5.52	4.33E-02
LOC107985941	3.80E-01	3.11E-01	2.98	5.52	4.33E-02
LOC105370325	3.80E-01	3.11E-01	2.98	5.52	4.33E-02
BMS1P2.AGAP9	3.80E-01	3.11E-01	2.98	5.52	4.33E-02
EPO	3.80E-01	3.11E-01	2.98	5.52	4.33E-02
OBP2A	3.80E-01	3.11E-01	2.98	5.52	4.34E-02
USP46.DT	3.80E-01	3.11E-01	2.98	5.52	4.34E-02
LOC105373748	3.80E-01	3.11E-01	2.98	5.52	4.34E-02
LINC01374	3.80E-01	3.11E-01	2.98	5.51	4.35E-02
RASGRP3.AS1	3.80E-01	3.11E-01	2.98	5.51	4.36E-02
LINC01256	3.80E-01	3.11E-01	2.98	5.51	4.36E-02
ZC3H12D	3.80E-01	3.11E-01	2.98	5.50	4.36E-02
CNNM3.DT	3.79E-01	3.11E-01	2.98	5.50	4.36E-02
FAM210A	3.79E-01	3.10E-01	2.98	5.50	4.36E-02
LOC112268127	3.79E-01	3.10E-01	2.98	5.50	4.36E-02
TAAR3P	3.79E-01	3.10E-01	2.98	5.50	4.36E-02
CCM2	3.79E-01	3.10E-01	2.98	5.50	4.36E-02
STK38	3.79E-01	3.10E-01	2.98	5.50	4.37E-02
BUD23	3.79E-01	3.10E-01	2.98	5.50	4.37E-02
LOC101928972	3.79E-01	3.10E-01	2.98	5.50	4.37E-02
LOC102724992	3.79E-01	3.10E-01	2.98	5.50	4.37E-02
THEM4	3.79E-01	3.10E-01	2.98	5.49	4.37E-02
LINC02110	3.79E-01	3.10E-01	2.98	5.49	4.38E-02
LOC105371912	3.79E-01	3.10E-01	2.98	5.49	4.38E-02
LOC440084	3.78E-01	3.09E-01	2.98	5.48	4.40E-02
CYTH2	3.78E-01	3.09E-01	2.98	5.47	4.41E-02
LOC105376631	3.78E-01	3.09E-01	2.98	5.47	4.41E-02
TRT.AGT1.3	3.77E-01	3.08E-01	2.98	5.45	4.43E-02
LOC105370077	3.77E-01	3.08E-01	2.98	5.45	4.44E-02
LOC105373769	3.77E-01	3.08E-01	2.98	5.45	4.44E-02

TENT5B	3.77E-01	3.08E-01	2.98	5.45	4.44E-02
H2BC1	3.77E-01	3.08E-01	2.98	5.45	4.44E-02
DCLK2	3.77E-01	3.08E-01	2.98	5.45	4.44E-02
LOC107984351	3.77E-01	3.08E-01	2.99	5.45	4.44E-02
LOC105371403	3.77E-01	3.08E-01	2.99	5.45	4.44E-02
STOX1	3.77E-01	3.08E-01	2.99	5.45	4.44E-02
SNORD113.9	3.77E-01	3.08E-01	2.99	5.45	4.45E-02
LOC107984480	3.77E-01	3.08E-01	2.99	5.44	4.45E-02
LOC107984548	3.77E-01	3.07E-01	2.99	5.44	4.46E-02
LOC105375765	3.77E-01	3.07E-01	2.99	5.44	4.46E-02
KRTAP10.8	3.77E-01	3.07E-01	2.99	5.44	4.46E-02
LINC02289	3.76E-01	3.07E-01	2.99	5.43	4.47E-02
LOC105379224	3.76E-01	3.07E-01	2.99	5.43	4.47E-02
LOC105374735	3.76E-01	3.07E-01	2.99	5.43	4.47E-02
GRIK1.AS2	3.76E-01	3.07E-01	2.99	5.43	4.47E-02
LOC107986478	3.76E-01	3.07E-01	2.99	5.43	4.47E-02
ESX1	3.76E-01	3.07E-01	2.99	5.43	4.47E-02
LOC399900	3.76E-01	3.07E-01	2.99	5.43	4.48E-02
FSCN3	3.76E-01	3.07E-01	2.99	5.43	4.48E-02
LOC107984964	3.76E-01	3.07E-01	2.99	5.43	4.48E-02
LINC02762	3.76E-01	3.07E-01	2.99	5.43	4.48E-02
LOC105373890	3.76E-01	3.07E-01	2.99	5.42	4.49E-02
IL24	3.76E-01	3.06E-01	2.99	5.42	4.49E-02
OR5AS1	3.76E-01	3.06E-01	2.99	5.41	4.50E-02
LOC107985486	3.75E-01	3.06E-01	2.99	5.41	4.50E-02
LOC105374573	3.75E-01	3.06E-01	2.99	5.41	4.50E-02
LOC105373269	3.75E-01	3.05E-01	2.99	5.39	4.53E-02
LOC105370210	3.75E-01	3.05E-01	2.99	5.39	4.53E-02
LOC101241902	3.75E-01	3.05E-01	2.99	5.39	4.54E-02
MAGEA6.DT	3.75E-01	3.05E-01	2.99	5.39	4.54E-02
LOC105377017	3.75E-01	3.05E-01	2.99	5.39	4.54E-02
NECAB3	3.74E-01	3.04E-01	2.99	5.38	4.56E-02
LOC105377209	3.74E-01	3.04E-01	2.99	5.38	4.56E-02
NEUROG2.AS1	3.74E-01	3.04E-01	2.99	5.38	4.56E-02
LOC105375377	3.74E-01	3.04E-01	2.99	5.37	4.56E-02
RAB31	3.74E-01	3.04E-01	2.99	5.37	4.56E-02
LOC105375748	3.74E-01	3.04E-01	2.99	5.37	4.57E-02
LOC105377666	3.74E-01	3.04E-01	2.99	5.37	4.57E-02
SLC34A2	3.74E-01	3.04E-01	2.99	5.37	4.57E-02
KIAA1328	3.74E-01	3.04E-01	2.99	5.37	4.57E-02
ZFP57_2	3.74E-01	3.04E-01	2.99	5.37	4.57E-02

LOC107987282	3.73E-01	3.04E-01	2.99	5.37	4.58E-02
FAS.AS1	3.73E-01	3.04E-01	2.99	5.37	4.58E-02
SRRM2	3.73E-01	3.04E-01	2.99	5.36	4.58E-02
LOC107984219	3.73E-01	3.03E-01	2.99	5.36	4.59E-02
LOC105371725	3.73E-01	3.03E-01	3.00	5.35	4.59E-02
HAUS8	3.73E-01	3.03E-01	3.00	5.35	4.60E-02
LOC107984220	3.73E-01	3.03E-01	3.00	5.35	4.60E-02
PEX7	3.73E-01	3.03E-01	3.00	5.35	4.60E-02
PLD6	3.73E-01	3.03E-01	3.00	5.35	4.61E-02
ENPP1	3.73E-01	3.03E-01	3.00	5.35	4.61E-02
SDHAP3	3.73E-01	3.03E-01	3.00	5.35	4.61E-02
HS6ST1	3.72E-01	3.03E-01	3.00	5.34	4.61E-02
HTR3E	3.72E-01	3.03E-01	3.00	5.34	4.62E-02
NPHP1	3.72E-01	3.02E-01	3.00	5.34	4.62E-02
FAM225B	3.72E-01	3.02E-01	3.00	5.33	4.63E-02
H1.2	3.72E-01	3.02E-01	3.00	5.33	4.63E-02
C19orf53	3.72E-01	3.02E-01	3.00	5.33	4.63E-02
LOC105373521	3.72E-01	3.02E-01	3.00	5.33	4.63E-02
LOC112267905	3.72E-01	3.02E-01	3.00	5.32	4.65E-02
LOC105372498	3.71E-01	3.01E-01	3.00	5.31	4.67E-02
ZBED3.AS1	3.71E-01	3.01E-01	3.00	5.31	4.67E-02
PTPRM	3.71E-01	3.01E-01	3.00	5.31	4.67E-02
NKX6.3	3.71E-01	3.01E-01	3.00	5.31	4.67E-02
STAMBPL1	3.71E-01	3.01E-01	3.00	5.30	4.68E-02
LINC01493	3.71E-01	3.01E-01	3.00	5.30	4.68E-02
SLC5A8_1	3.70E-01	3.01E-01	3.00	5.30	4.69E-02
MVP	3.70E-01	3.00E-01	3.00	5.29	4.69E-02
RNF14	3.70E-01	3.00E-01	3.00	5.29	4.70E-02
PNPT1	3.70E-01	3.00E-01	3.00	5.29	4.71E-02
ZEB2	3.70E-01	3.00E-01	3.00	5.29	4.71E-02
SIGIRR	3.70E-01	3.00E-01	3.00	5.29	4.71E-02
LOC105369925	3.70E-01	3.00E-01	3.00	5.28	4.71E-02
UCP2	3.70E-01	3.00E-01	3.00	5.28	4.72E-02
LOC153684	3.69E-01	2.99E-01	3.00	5.27	4.73E-02
LOC105373707	3.69E-01	2.99E-01	3.00	5.27	4.74E-02
LOC112268313	3.69E-01	2.99E-01	3.00	5.27	4.74E-02
LINC01348	3.69E-01	2.99E-01	3.00	5.26	4.75E-02
LOC105371056	3.69E-01	2.99E-01	3.00	5.26	4.75E-02
GABARAPL3	3.69E-01	2.99E-01	3.00	5.26	4.75E-02
HOXD1	3.69E-01	2.99E-01	3.01	5.26	4.75E-02
C1orf210	3.69E-01	2.98E-01	3.01	5.25	4.76E-02

TRAV34	3.69E-01	2.98E-01	3.01	5.25	4.76E-02
DNM3.IT1	3.68E-01	2.98E-01	3.01	5.25	4.77E-02
LINC01778	3.68E-01	2.98E-01	3.01	5.25	4.77E-02
PUSL1	3.68E-01	2.98E-01	3.01	5.25	4.77E-02
INMT	3.68E-01	2.98E-01	3.01	5.25	4.77E-02
CRH	3.68E-01	2.98E-01	3.01	5.24	4.78E-02
MCMBP	3.68E-01	2.98E-01	3.01	5.24	4.78E-02
UBE2C	3.68E-01	2.97E-01	3.01	5.23	4.79E-02
HES2	3.68E-01	2.97E-01	3.01	5.23	4.80E-02
COX4I1	3.67E-01	2.97E-01	3.01	5.23	4.80E-02
LPIN2	3.67E-01	2.97E-01	3.01	5.23	4.80E-02
LOC107986418	3.67E-01	2.97E-01	3.01	5.23	4.81E-02
LOC105377989	3.67E-01	2.97E-01	3.01	5.22	4.81E-02
TUBGCP6	3.67E-01	2.97E-01	3.01	5.22	4.82E-02
C8orf88	3.67E-01	2.97E-01	3.01	5.22	4.82E-02
SYT10	3.67E-01	2.97E-01	3.01	5.22	4.82E-02
LOC101926960	3.67E-01	2.97E-01	3.01	5.22	4.82E-02
LOC105377740	3.67E-01	2.97E-01	3.01	5.22	4.83E-02
SELP	3.67E-01	2.97E-01	3.01	5.22	4.83E-02
MANCR	3.67E-01	2.97E-01	3.01	5.21	4.83E-02
LINC01107	3.67E-01	2.96E-01	3.01	5.21	4.83E-02
ATP1A2	3.67E-01	2.96E-01	3.01	5.21	4.83E-02
ABI1	3.67E-01	2.96E-01	3.01	5.21	4.84E-02
LOC105378468	3.67E-01	2.96E-01	3.01	5.21	4.84E-02
REPIN1.AS1	3.67E-01	2.96E-01	3.01	5.21	4.84E-02
MPST	3.66E-01	2.96E-01	3.01	5.21	4.84E-02
SOCS2	3.66E-01	2.96E-01	3.01	5.20	4.85E-02
ARMC3	3.66E-01	2.96E-01	3.01	5.20	4.85E-02
PASD1	3.66E-01	2.96E-01	3.01	5.20	4.85E-02
PLXNA4	3.66E-01	2.96E-01	3.01	5.20	4.85E-02
LOC105370847	3.66E-01	2.96E-01	3.01	5.20	4.85E-02
BACH1.IT3	3.66E-01	2.96E-01	3.01	5.20	4.86E-02
LOC105376558	3.66E-01	2.96E-01	3.01	5.20	4.86E-02
SHD	3.66E-01	2.96E-01	3.01	5.20	4.86E-02
OR52E8	3.66E-01	2.95E-01	3.01	5.19	4.87E-02
LOC107986492	3.66E-01	2.95E-01	3.01	5.19	4.87E-02
LOC107987110	3.66E-01	2.95E-01	3.01	5.19	4.87E-02
SHC4	3.66E-01	2.95E-01	3.01	5.19	4.88E-02
TRBV6.9	3.65E-01	2.95E-01	3.01	5.18	4.88E-02
LOC105370513	3.65E-01	2.95E-01	3.01	5.18	4.89E-02
PABPC5	3.65E-01	2.95E-01	3.01	5.18	4.89E-02

LOC105379283	3.65E-01	2.95E-01	3.01	5.18	4.89E-02
LOC107987249	3.65E-01	2.95E-01	3.01	5.18	4.89E-02
C1orf185	3.65E-01	2.94E-01	3.02	5.17	4.91E-02
TRPV3	3.65E-01	2.94E-01	3.02	5.16	4.92E-02
LOC105375001	3.64E-01	2.94E-01	3.02	5.16	4.92E-02
LOC107986896	3.64E-01	2.94E-01	3.02	5.16	4.93E-02
LINC00260	3.64E-01	2.94E-01	3.02	5.16	4.93E-02
ADAM7	3.64E-01	2.94E-01	3.02	5.15	4.93E-02
GSTA2	3.64E-01	2.93E-01	3.02	5.15	4.93E-02
CHRNA4	3.64E-01	2.93E-01	3.02	5.15	4.94E-02
MFSD9	3.64E-01	2.93E-01	3.02	5.15	4.94E-02
OR2B6	3.64E-01	2.93E-01	3.02	5.15	4.95E-02
FOXE3	3.64E-01	2.93E-01	3.02	5.15	4.95E-02
LOC102724214	3.64E-01	2.93E-01	3.02	5.14	4.95E-02
LINC00354	3.64E-01	2.93E-01	3.02	5.14	4.96E-02
LOC100506405	3.63E-01	2.93E-01	3.02	5.14	4.96E-02
MIR5698	3.63E-01	2.93E-01	3.02	5.14	4.97E-02
FZD7	3.63E-01	2.92E-01	3.02	5.13	4.97E-02
TMEM67	3.63E-01	2.92E-01	3.02	5.13	4.97E-02
LOC105373830	3.63E-01	2.92E-01	3.02	5.13	4.97E-02
IGLVVI.25.1	3.63E-01	2.92E-01	3.02	5.13	4.98E-02
DHX57	3.63E-01	2.92E-01	3.02	5.13	4.98E-02
LOC107986169	3.63E-01	2.92E-01	3.02	5.13	4.99E-02
CA2	3.63E-01	2.92E-01	3.02	5.12	4.99E-02
FAM156A	3.63E-01	2.92E-01	3.02	5.12	4.99E-02
FAXDC2	3.63E-01	2.92E-01	3.02	5.12	4.99E-02
LOC107986277	3.63E-01	2.92E-01	3.02	5.12	5.00E-02
HOXB.AS1	3.63E-01	2.92E-01	3.02	5.12	5.00E-02
LOC107986285	3.63E-01	2.92E-01	3.02	5.12	5.00E-02
