# Supplementary material for: RNA Sequencing of Intestinal Enterocytes Pre- and Post-Roux-en-Y Gastric Bypass Reveals Alteration in Gene Expression Related to Enterocyte Differentiation, Restitution, and Obesity with Regulation by Schlafen 12
Source: Cells. 2022 Oct 18;11(20):3283. doi: 10.3390/cells11203283 (PMC9601224; doi:10.3390/cells11203283)
Supplement: Supplementary file 1 [file cells-11-03283-s001.zip › Table S3.pdf]

	RYGB (Pre vs Post)				BMI				Interaction			
	Estimate	Std. Error	t value	Pval	Estimate	Std. Error	t value	Pval	Estimate	Std. Error	t value	Pval
SCGB3A1	-22.014	4.456	-4.941	1.06E-04	-0.598	0.062	-9.709	1.40E-08	0.598	0.104	5.748	1.90E-05
MIR6503	-101.561	24.566	-4.134	6.23E-04	-2.775	0.340	-8.166	1.83E-07	2.775	0.574	4.834	1.33E-04
LOC1079841	-23.684	5.794	-4.088	6.91E-04	-0.643	0.080	-8.017	2.38E-07	0.643	0.135	4.746	1.61E-04
LOC1079854	-237.996	62.090	-3.833	1.22E-03	-7.369	0.859	-8.580	8.92E-08	6.697	1.451	4.616	2.14E-04
MIR548P	-174.994	46.415	-3.770	1.40E-03	-4.743	0.642	-7.388	7.47E-07	4.743	1.084	4.374	3.66E-04
MIR4645	-91.594	24.475	-3.742	1.49E-03	-2.491	0.339	-7.357	7.92E-07	2.491	0.572	4.355	3.82E-04
LOC1122680	-23.152	6.190	-3.740	1.50E-03	-0.624	0.086	-7.291	8.95E-07	0.625	0.145	4.324	4.09E-04
SNORA58B	-131.266	36.123	-3.634	1.90E-03	-3.495	0.500	-6.996	1.57E-06	3.495	0.844	4.141	6.13E-04
SNORD5	-127.871	36.862	-3.469	2.74E-03	-3.510	0.510	-6.884	1.94E-06	3.510	0.861	4.075	7.10E-04
CYP2D7_2	-4.107	1.184	-3.469	2.74E-03	-0.113	0.016	-6.884	1.94E-06	0.113	0.028	4.075	7.10E-04
LINC01740	-158.558	46.210	-3.431	2.98E-03	-4.567	0.639	-7.145	1.18E-06	4.381	1.080	4.058	7.38E-04
DCLRE1CP1	-15.920	4.654	-3.421	3.05E-03	-0.437	0.064	-6.792	2.32E-06	0.437	0.109	4.021	8.02E-04
LOC1053781	-4.428	1.311	-3.378	3.35E-03	-0.121	0.018	-6.697	2.79E-06	0.121	0.031	3.965	9.09E-04
LOC389831	-1.170	0.346	-3.378	3.35E-03	-0.032	0.005	-6.697	2.79E-06	0.032	0.008	3.965	9.09E-04
IGKC	-16.299	4.825	-3.378	3.35E-03	-0.447	0.067	-6.697	2.79E-06	0.447	0.113	3.965	9.09E-04
GLIS2_1	-3.393	1.004	-3.378	3.35E-03	-0.093	0.014	-6.697	2.79E-06	0.093	0.023	3.965	9.09E-04
FOXL3.OT1	-8.162	2.416	-3.378	3.35E-03	-0.224	0.033	-6.697	2.79E-06	0.224	0.056	3.965	9.09E-04
SNORD165	-83.563	24.739	-3.378	3.35E-03	-2.292	0.342	-6.697	2.79E-06	2.292	0.578	3.965	9.09E-04
LOC1079874	-4.019	1.190	-3.378	3.35E-03	-0.110	0.016	-6.697	2.79E-06	0.110	0.028	3.965	9.09E-04
MIR29A	-41.129	12.176	-3.378	3.35E-03	-1.128	0.168	-6.697	2.79E-06	1.128	0.284	3.965	9.09E-04
SLC2A4RG	-24.195	6.751	-3.584	2.12E-03	-0.499	0.093	-5.347	4.41E-05	0.623	0.158	3.949	9.41E-04
CPEB1	-2.898	0.872	-3.325	3.77E-03	-0.078	0.012	-6.443	4.61E-06	0.078	0.020	3.814	1.27E-03
PARN_1	-1.325	0.418	-3.169	5.31E-03	-0.036	0.006	-6.297	6.17E-06	0.036	0.010	3.728	1.54E-03
TTTY9B	-4.973	1.569	-3.169	5.31E-03	-0.137	0.022	-6.297	6.17E-06	0.137	0.037	3.728	1.54E-03
TRR.ACG2.4	-111.510	35.189	-3.169	5.31E-03	-3.065	0.487	-6.297	6.17E-06	3.065	0.822	3.728	1.54E-03
B3GALT4_4	-9.560	3.017	-3.169	5.31E-03	-0.263	0.042	-6.297	6.17E-06	0.263	0.070	3.728	1.54E-03
TRBV2	-23.459	7.403	-3.169	5.31E-03	-0.645	0.102	-6.297	6.17E-06	0.645	0.173	3.728	1.54E-03
MIR593	-81.403	25.688	-3.169	5.31E-03	-2.237	0.355	-6.297	6.17E-06	2.237	0.600	3.728	1.54E-03
DEFB103B_2	-7.857	2.480	-3.169	5.31E-03	-0.216	0.034	-6.297	6.17E-06	0.216	0.058	3.728	1.54E-03
LOC1053794	-14.281	4.507	-3.169	5.31E-03	-0.393	0.062	-6.297	6.17E-06	0.393	0.105	3.728	1.54E-03
MIR3606	-64.605	20.387	-3.169	5.31E-03	-1.776	0.282	-6.297	6.17E-06	1.776	0.476	3.728	1.54E-03
LOC1079873	-0.799	0.252	-3.169	5.31E-03	-0.022	0.003	-6.297	6.17E-06	0.022	0.006	3.728	1.54E-03
LOC1122682	-26.515	8.367	-3.169	5.31E-03	-0.729	0.116	-6.297	6.17E-06	0.729	0.196	3.728	1.54E-03
LOC1019297	-15.634	4.659	-3.355	3.52E-03	-0.277	0.064	-4.296	4.35E-04	0.402	0.109	3.692	1.67E-03
CENPS.CORT	-4.621	1.470	-3.143	5.62E-03	-0.125	0.020	-6.163	8.09E-06	0.125	0.034	3.648	1.84E-03
LOC1079847	-68.732	21.928	-3.134	5.73E-03	-1.701	0.303	-5.607	2.55E-05	1.865	0.512	3.640	1.87E-03
LOC1019287	106.023	25.767	4.115	6.51E-04	-0.136	0.356	-0.383	7.06E-01	-2.174	0.602	-3.611	2.00E-03
FRMPD1	82.198	23.210	3.541	2.33E-03	0.478	0.321	1.489	1.54E-01	-1.957	0.542	-3.609	2.00E-03
PAFAH1B3	-32.856	10.214	-3.217	4.78E-03	-0.778	0.141	-5.508	3.14E-05	0.860	0.239	3.604	2.03E-03
LOC1009968	-26.047	8.683	-3.000	7.69E-03	-0.770	0.120	-6.410	4.92E-06	0.725	0.203	3.574	2.17E-03
MAFA	-99.195	27.082	-3.663	1.78E-03	-0.154	0.375	-0.410	6.87E-01	2.259	0.633	3.570	2.19E-03
IGLV1.62	-46.602	16.123	-2.890	9.74E-03	-1.544	0.223	-6.926	1.79E-06	1.345	0.377	3.569	2.19E-03
FAM197Y6	-38.934	12.794	-3.043	7.00E-03	-1.067	0.177	-6.027	1.07E-05	1.067	0.299	3.568	2.20E-03
MIR4517	-167.872	53.804	-3.120	5.91E-03	-3.837	0.744	-5.155	6.64E-05	4.476	1.257	3.560	2.24E-03
LOC1027237	-251.754	70.889	-3.551	2.28E-03	-1.186	0.981	-1.209	2.42E-01	5.837	1.656	3.524	2.42E-03
TRG.CCC4.1	-455.647	146.070	-3.119	5.92E-03	-9.840	2.020	-4.870	1.23E-04	11.974	3.413	3.508	2.51E-03
LINC02017	-18.786	6.113	-3.073	6.56E-03	-0.499	0.085	-5.905	1.37E-05	0.499	0.143	3.496	2.58E-03
MIR382	-997.883	291.375	-3.425	3.02E-03	-7.121	4.030	-1.767	9.42E-02	23.706	6.808	3.482	2.66E-03
GON4L	93.016	27.925	3.331	3.72E-03	0.699	0.386	1.810	8.70E-02	-2.259	0.652	-3.462	2.78E-03
SRPK2	-129.520	36.649	-3.534	2.37E-03	-0.219	0.507	-0.431	6.71E-01	2.963	0.856	3.460	2.80E-03
MIR493	-526.831	176.500	-2.985	7.94E-03	-14.266	2.441	-5.844	1.55E-05	14.266	4.124	3.459	2.80E-03

MIR1285.1	-147.316	49.114	-3.000	7.69E-03	-3.939	0.679	-5.799	1.71E-05	3.939	1.148	3.433	2.97E-03
LOC1053706	-123.697	38.415	-3.220	4.75E-03	-1.419	0.531	-2.670	1.56E-02	3.071	0.898	3.421	3.05E-03
ANKRD26	55.275	15.932	3.469	2.74E-03	0.120	0.220	0.544	5.93E-01	-1.271	0.372	-3.415	3.08E-03
PKD1L2	64.139	18.930	3.388	3.28E-03	0.310	0.262	1.185	2.51E-01	-1.510	0.442	-3.415	3.09E-03
LOC1053759	243.360	66.055	3.684	1.70E-03	0.707	0.914	0.773	4.49E-01	-5.259	1.543	-3.408	3.14E-03
MIR548AG1	-224.410	75.613	-2.968	8.24E-03	-6.009	1.046	-5.746	1.91E-05	6.009	1.767	3.401	3.18E-03
LOC1053719	-16.514	5.608	-2.945	8.66E-03	-0.445	0.078	-5.737	1.94E-05	0.445	0.131	3.397	3.22E-03
NEK8	-308.388	89.479	-3.447	2.88E-03	-0.542	1.238	-0.438	6.67E-01	6.975	2.091	3.336	3.68E-03
CHRFAM7A	21.241	5.662	3.752	1.46E-03	0.001	0.078	0.008	9.94E-01	-0.440	0.132	-3.327	3.75E-03
IFNL3	199.891	53.863	3.711	1.60E-03	-0.001	0.745	-0.001	9.99E-01	-4.170	1.259	-3.314	3.86E-03
PYDC2	-51.886	19.812	-2.619	1.74E-02	-1.844	0.274	-6.730	2.62E-06	1.529	0.463	3.303	3.95E-03
NF1	80.257	26.958	2.977	8.08E-03	0.818	0.373	2.195	4.16E-02	-2.068	0.630	-3.283	4.13E-03
AASS	-218.435	66.049	-3.307	3.92E-03	-0.785	0.914	-0.860	4.01E-01	5.059	1.543	3.278	4.18E-03
LOC1053709	334.167	90.278	3.702	1.63E-03	-0.118	1.249	-0.095	9.26E-01	-6.878	2.109	-3.261	4.34E-03
TRAJ17	-293.277	110.473	-2.655	1.61E-02	-9.394	1.528	-6.148	8.34E-06	8.414	2.581	3.260	4.35E-03
GPRC5A	57.069	19.147	2.981	8.02E-03	0.563	0.265	2.124	4.78E-02	-1.451	0.447	-3.244	4.50E-03
ACSBG1	-77.061	23.890	-3.226	4.69E-03	-0.270	0.330	-0.817	4.25E-01	1.804	0.558	3.233	4.62E-03
LOC1079846	-170.091	52.757	-3.224	4.71E-03	-1.010	0.730	-1.384	1.83E-01	3.983	1.233	3.231	4.64E-03
MIR124.1HG	-4.279	1.477	-2.897	9.61E-03	-0.077	0.020	-3.748	1.47E-03	0.111	0.035	3.229	4.65E-03
LINC01911	135.614	43.821	3.095	6.25E-03	0.921	0.606	1.520	1.46E-01	-3.305	1.024	-3.228	4.67E-03
RIMBP3C	-10.058	3.341	-3.011	7.51E-03	-0.147	0.046	-3.189	5.08E-03	0.252	0.078	3.228	4.67E-03
RNU6.7	-51.477	18.793	-2.739	1.35E-02	-1.416	0.260	-5.449	3.55E-05	1.416	0.439	3.226	4.69E-03
RASSF7	-10.134	3.700	-2.739	1.35E-02	-0.279	0.051	-5.449	3.55E-05	0.279	0.086	3.226	4.69E-03
HCG18_2	-0.619	0.226	-2.739	1.35E-02	-0.017	0.003	-5.449	3.55E-05	0.017	0.005	3.226	4.69E-03
DHODH	84.056	25.849	3.252	4.43E-03	0.442	0.358	1.236	2.32E-01	-1.947	0.604	-3.223	4.72E-03
MIR105.2	1327.416	373.271	3.556	2.26E-03	0.154	5.163	0.030	9.77E-01	-27.810	8.721	-3.189	5.09E-03
SYP	58.065	16.636	3.490	2.61E-03	0.059	0.230	0.257	8.00E-01	-1.239	0.389	-3.188	5.10E-03
ZNF460.AS1	162.951	49.029	3.324	3.78E-03	0.619	0.678	0.913	3.73E-01	-3.645	1.146	-3.182	5.16E-03
LOC1079846	-83.799	30.198	-2.775	1.25E-02	-1.898	0.418	-4.545	2.51E-04	2.243	0.706	3.179	5.20E-03
LRIG2.DT	-17.080	5.739	-2.976	8.09E-03	-0.261	0.079	-3.284	4.12E-03	0.426	0.134	3.177	5.22E-03
OVOL1.AS1	-20.239	7.448	-2.717	1.41E-02	-0.552	0.103	-5.359	4.29E-05	0.552	0.174	3.173	5.27E-03
TMEM127	-91.725	28.444	-3.225	4.70E-03	-0.157	0.393	-0.400	6.94E-01	2.100	0.665	3.159	5.43E-03
LOC1053700	144.895	37.976	3.815	1.27E-03	-0.594	0.525	-1.130	2.73E-01	-2.794	0.887	-3.149	5.55E-03
LOC1125775	156.092	43.100	3.622	1.95E-03	-0.239	0.596	-0.401	6.93E-01	-3.167	1.007	-3.145	5.60E-03
OCEL1	79.953	23.680	3.376	3.36E-03	0.262	0.328	0.800	4.34E-01	-1.738	0.553	-3.141	5.65E-03
LOC1053720	80.002	21.863	3.659	1.79E-03	-0.177	0.302	-0.585	5.66E-01	-1.601	0.511	-3.134	5.73E-03
ERCC8.AS1	-312.403	97.248	-3.212	4.83E-03	-0.786	1.345	-0.584	5.66E-01	7.122	2.272	3.134	5.73E-03
LOC1122681	-45.026	15.911	-2.830	1.11E-02	-0.934	0.220	-4.245	4.87E-04	1.165	0.372	3.134	5.74E-03
FAM247D	21.837	6.834	3.195	5.01E-03	0.104	0.095	1.104	2.84E-01	-0.498	0.160	-3.120	5.91E-03
DDAH1	127.617	42.004	3.038	7.07E-03	1.043	0.581	1.795	8.95E-02	-3.059	0.981	-3.117	5.95E-03
TRQ.CTG4.1	-376.620	141.503	-2.662	1.59E-02	-10.290	1.957	-5.257	5.34E-05	10.290	3.306	3.112	6.01E-03
GPR22	3.826	1.080	3.542	2.33E-03	0.000	0.015	0.000	1.00E+00	-0.078	0.025	-3.104	6.13E-03
KCNIP3	59.590	16.452	3.622	1.95E-03	-0.159	0.228	-0.699	4.94E-01	-1.193	0.384	-3.103	6.13E-03
LOC1079845	-180.167	67.919	-2.653	1.62E-02	-5.147	0.939	-5.478	3.34E-05	4.918	1.587	3.099	6.19E-03
SORBS1	-195.194	61.915	-3.153	5.51E-03	-0.351	0.856	-0.409	6.87E-01	4.481	1.447	3.098	6.21E-03
GRAPL	-10.516	3.849	-2.732	1.37E-02	-0.201	0.053	-3.777	1.38E-03	0.278	0.090	3.089	6.33E-03
KRTAP19.2	-80.615	31.062	-2.595	1.83E-02	-2.224	0.430	-5.176	6.35E-05	2.233	0.726	3.077	6.50E-03
GGH	-151.350	51.506	-2.939	8.78E-03	-1.368	0.712	-1.920	7.08E-02	3.703	1.203	3.077	6.50E-03
LOC149684	263.472	82.801	3.182	5.16E-03	1.274	1.145	1.112	2.81E-01	-5.931	1.935	-3.066	6.66E-03
TRR.TCT3.2	1661.210	522.471	3.180	5.19E-03	8.727	7.227	1.208	2.43E-01	-37.424	12.207	-3.066	6.66E-03
MIR554	-202.240	72.321	-2.796	1.19E-02	-3.395	1.000	-3.393	3.24E-03	5.177	1.690	3.064	6.69E-03
LOC1009966	-266.524	84.928	-3.138	5.68E-03	-0.046	1.175	-0.039	9.69E-01	6.053	1.984	3.050	6.89E-03
SLC38A1	-83.711	27.597	-3.033	7.15E-03	-0.160	0.382	-0.419	6.80E-01	1.965	0.645	3.047	6.94E-03

TRAV13.1	189.153	55.640	3.400	3.19E-03	0.097	0.770	0.126	9.01E-01	-3.954	1.300	-3.042	7.02E-03
TRNAV.CAC_1	262.186	76.420	3.431	2.98E-03	0.000	1.057	0.000	1.00E+00	-5.425	1.786	-3.038	7.07E-03
LINC01901	324.675	92.025	3.528	2.40E-03	-0.514	1.273	-0.404	6.91E-01	-6.531	2.150	-3.038	7.08E-03
SNORA71D	292.347	86.173	3.393	3.24E-03	0.073	1.192	0.061	9.52E-01	-6.115	2.013	-3.037	7.09E-03
ETDA	43.943	13.153	3.341	3.64E-03	-0.013	0.182	-0.071	9.44E-01	-0.933	0.307	-3.034	7.13E-03
HTT.AS	187.412	55.001	3.407	3.14E-03	0.013	0.761	0.017	9.87E-01	-3.895	1.285	-3.031	7.18E-03
IBA57.DT	49.435	13.549	3.649	1.84E-03	-0.225	0.187	-1.202	2.45E-01	-0.959	0.317	-3.029	7.21E-03
MIR599	-193.877	67.924	-2.854	1.05E-02	-2.674	0.940	-2.846	1.07E-02	4.801	1.587	3.025	7.28E-03
TSBP1_1	-54.631	17.313	-3.156	5.47E-03	0.000	0.239	0.000	1.00E+00	1.222	0.405	3.022	7.33E-03
MAP3K8	73.797	23.296	3.168	5.33E-03	0.196	0.322	0.607	5.51E-01	-1.644	0.544	-3.020	7.36E-03
LOC10798539	-249.101	88.166	-2.825	1.12E-02	-3.979	1.220	-3.263	4.32E-03	6.221	2.060	3.020	7.36E-03
DOCK8	81.199	27.438	2.959	8.39E-03	0.502	0.380	1.324	2.02E-01	-1.936	0.641	-3.020	7.36E-03
CYP21A2_5	2.681	0.784	3.419	3.06E-03	0.000	0.011	0.000	1.00E+00	-0.055	0.018	-3.016	7.42E-03
SNORA71C	78.564	22.979	3.419	3.06E-03	0.000	0.318	0.000	1.00E+00	-1.619	0.537	-3.016	7.42E-03
LINC01903	137.788	46.423	2.968	8.24E-03	0.875	0.642	1.362	1.90E-01	-3.270	1.085	-3.015	7.44E-03
LOC10272360	123.316	37.034	3.330	3.73E-03	0.071	0.512	0.139	8.91E-01	-2.607	0.865	-3.013	7.46E-03
LOC10798482	-266.391	86.956	-3.064	6.69E-03	-0.195	1.203	-0.162	8.73E-01	6.109	2.032	3.007	7.57E-03
INSYN1.AS1	187.065	55.331	3.381	3.33E-03	-0.309	0.765	-0.404	6.91E-01	-3.886	1.293	-3.006	7.59E-03
NOTCH4_2	-1.493	0.562	-2.654	1.61E-02	-0.039	0.008	-5.075	7.89E-05	0.039	0.013	3.005	7.61E-03
HSBP1L1	-65.718	25.245	-2.603	1.80E-02	-1.860	0.349	-5.328	4.59E-05	1.772	0.590	3.004	7.61E-03
LOC10537578	51.643	17.008	3.036	7.10E-03	0.393	0.235	1.670	1.12E-01	-1.193	0.397	-3.002	7.66E-03
IRAK1	-165.505	54.167	-3.055	6.81E-03	-0.643	0.749	-0.858	4.02E-01	3.792	1.266	2.996	7.75E-03
SNORD126	328.897	99.799	3.296	4.02E-03	0.326	1.380	0.236	8.16E-01	-6.979	2.332	-2.993	7.81E-03
LOC10537262	-236.956	88.185	-2.687	1.51E-02	-4.744	1.220	-3.889	1.08E-03	6.152	2.060	2.986	7.92E-03
LINC00471	139.180	42.500	3.275	4.21E-03	0.140	0.588	0.238	8.14E-01	-2.954	0.993	-2.975	8.11E-03
AUH	71.736	26.716	2.685	1.51E-02	0.586	0.370	1.585	1.30E-01	-1.856	0.624	-2.973	8.15E-03
MUTYH	-236.088	77.590	-3.043	7.00E-03	-0.479	1.073	-0.446	6.61E-01	5.377	1.813	2.966	8.27E-03
MIR125B1	932.207	276.105	3.376	3.36E-03	-1.166	3.819	-0.305	7.64E-01	-19.096	6.451	-2.960	8.38E-03
USF3	58.946	18.371	3.209	4.87E-03	-0.048	0.254	-0.189	8.52E-01	-1.268	0.429	-2.954	8.49E-03
LOC10537257	169.397	52.243	3.242	4.52E-03	0.163	0.723	0.226	8.24E-01	-3.605	1.221	-2.953	8.50E-03
SNORD116.7	-334.671	134.337	-2.491	2.27E-02	-9.583	1.858	-5.157	6.62E-05	9.264	3.139	2.952	8.54E-03
ALOX12P2	-183.336	59.865	-3.063	6.71E-03	-0.218	0.828	-0.264	7.95E-01	4.128	1.399	2.951	8.54E-03
PRAMEF18	70.920	26.173	2.710	1.44E-02	0.806	0.362	2.225	3.91E-02	-1.804	0.612	-2.950	8.56E-03
SNORD62A	-110.675	48.489	-2.282	3.48E-02	-4.280	0.671	-6.382	5.20E-06	3.340	1.133	2.948	8.60E-03
GOLGA8EP_2	-1.337	0.521	-2.567	1.94E-02	-0.036	0.007	-4.980	9.70E-05	0.036	0.012	2.948	8.60E-03
CACNG8	-117.072	40.369	-2.900	9.54E-03	-0.662	0.558	-1.185	2.51E-01	2.777	0.943	2.944	8.68E-03
RPF1	138.841	44.688	3.107	6.09E-03	0.046	0.618	0.075	9.41E-01	-3.071	1.044	-2.941	8.73E-03
WSCD1	61.258	19.015	3.222	4.73E-03	-0.076	0.263	-0.287	7.77E-01	-1.306	0.444	-2.940	8.76E-03
DOC2B_1	-2.292	0.921	-2.489	2.28E-02	-0.063	0.013	-4.954	1.03E-04	0.063	0.022	2.933	8.89E-03
IGKJ5	-365.576	146.881	-2.489	2.28E-02	-10.065	2.032	-4.954	1.03E-04	10.065	3.432	2.933	8.89E-03
MIR4705	-97.830	39.306	-2.489	2.28E-02	-2.693	0.544	-4.954	1.03E-04	2.693	0.918	2.933	8.89E-03
ZNF800	-39.457	13.708	-2.878	1.00E-02	-0.302	0.190	-1.595	1.28E-01	0.939	0.320	2.932	8.91E-03
LETM2	-194.020	66.253	-2.928	8.98E-03	-0.762	0.916	-0.831	4.17E-01	4.533	1.548	2.929	8.97E-03
PAQR8	-81.807	30.094	-2.718	1.41E-02	-0.857	0.416	-2.058	5.44E-02	2.059	0.703	2.928	8.99E-03
SUCLG1	180.374	56.583	3.188	5.10E-03	0.095	0.783	0.122	9.05E-01	-3.862	1.322	-2.921	9.12E-03
RASGRF2.AS1	200.980	68.467	2.935	8.84E-03	1.045	0.947	1.103	2.84E-01	-4.661	1.600	-2.913	9.27E-03
LOC10798487	-3.316	1.334	-2.485	2.30E-02	-0.091	0.018	-4.915	1.12E-04	0.091	0.031	2.910	9.34E-03
LINC01193	-5.737	2.194	-2.615	1.75E-02	-0.101	0.030	-3.320	3.81E-03	0.149	0.051	2.908	9.38E-03
LOC10537392	-70.629	30.362	-2.326	3.19E-02	-2.341	0.420	-5.574	2.73E-05	2.062	0.709	2.906	9.42E-03
FAM74A4	-31.385	12.202	-2.572	1.92E-02	-0.611	0.169	-3.622	1.95E-03	0.828	0.285	2.905	9.44E-03
PRADC1	-99.027	35.253	-2.809	1.16E-02	-0.908	0.488	-1.862	7.89E-02	2.392	0.824	2.904	9.46E-03
PKD1P6.NPIP	9.093	2.781	3.270	4.25E-03	0.000	0.038	0.000	1.00E+00	-0.188	0.065	-2.901	9.53E-03
LOC10192957	98.954	29.660	3.336	3.67E-03	-0.189	0.410	-0.462	6.50E-01	-2.000	0.693	-2.886	9.85E-03

SMG6	59.328	18.387	3.227	4.68E-03	-0.048	0.254	-0.189	8.52E-01	-1.238	0.430	-2.881	9.95E-03
RRN3	9.709	3.116	3.116	5.97E-03	0.011	0.043	0.263	7.95E-01	-0.210	0.073	-2.879	9.98E-03
BICD1	72.642	24.088	3.016	7.43E-03	0.008	0.333	0.024	9.81E-01	-1.617	0.563	-2.872	1.01E-02
LOC1079867	149.982	48.090	3.119	5.93E-03	0.321	0.665	0.482	6.35E-01	-3.218	1.124	-2.864	1.03E-02
LOC1053749	-159.739	67.037	-2.383	2.84E-02	-5.067	0.927	-5.465	3.44E-05	4.482	1.566	2.861	1.04E-02
LOC1053738	-43.552	17.943	-2.427	2.59E-02	-1.271	0.248	-5.121	7.15E-05	1.198	0.419	2.859	1.04E-02
LOC1027233	488.965	172.890	2.828	1.11E-02	3.708	2.391	1.550	1.38E-01	-11.547	4.040	-2.858	1.04E-02
CTB.1I21.1	-205.004	74.307	-2.759	1.29E-02	-1.365	1.028	-1.328	2.01E-01	4.949	1.736	2.850	1.06E-02
SNORD15B	-122.941	50.283	-2.445	2.50E-02	-3.348	0.696	-4.814	1.39E-04	3.348	1.175	2.850	1.06E-02
LINC01093	151.650	50.269	3.017	7.41E-03	0.501	0.695	0.721	4.80E-01	-3.347	1.175	-2.849	1.06E-02
IGKV2.4_1	-15.877	6.043	-2.627	1.71E-02	-0.262	0.084	-3.132	5.76E-03	0.402	0.141	2.848	1.07E-02
IGSF21	-228.589	77.696	-2.942	8.71E-03	-0.139	1.075	-0.129	8.99E-01	5.166	1.815	2.846	1.07E-02
LOC1079860	-134.512	51.096	-2.633	1.69E-02	-2.175	0.707	-3.077	6.49E-03	3.395	1.194	2.844	1.08E-02
NAP1L4_1	19.892	6.629	3.001	7.67E-03	0.061	0.092	0.660	5.17E-01	-0.440	0.155	-2.842	1.08E-02
CCL23_1	-14.086	5.681	-2.479	2.33E-02	-0.377	0.079	-4.798	1.44E-04	0.377	0.133	2.841	1.08E-02
LINC01741	159.097	60.871	2.614	1.76E-02	1.684	0.842	2.000	6.08E-02	-4.039	1.422	-2.840	1.09E-02
LOC1079861	423.162	131.978	3.206	4.89E-03	0.208	1.826	0.114	9.11E-01	-8.756	3.084	-2.840	1.09E-02
H2BW1	100.537	30.403	3.307	3.92E-03	-0.258	0.421	-0.614	5.47E-01	-2.016	0.710	-2.839	1.09E-02
CLEC10A	-140.510	48.472	-2.899	9.57E-03	-0.347	0.670	-0.518	6.11E-01	3.210	1.133	2.834	1.10E-02
LY9	-160.259	56.037	-2.860	1.04E-02	-0.444	0.775	-0.573	5.73E-01	3.708	1.309	2.832	1.10E-02
C4orf48	62.350	20.536	3.036	7.11E-03	0.015	0.284	0.051	9.60E-01	-1.359	0.480	-2.832	1.11E-02
FAM197Y4	-94.945	35.508	-2.674	1.55E-02	-1.267	0.491	-2.579	1.89E-02	2.348	0.830	2.831	1.11E-02
TACO1	-50.635	21.911	-2.311	3.29E-02	-1.583	0.303	-5.224	5.74E-05	1.448	0.512	2.829	1.11E-02
LINC02526	209.274	64.608	3.239	4.55E-03	-0.468	0.894	-0.523	6.07E-01	-4.269	1.510	-2.828	1.11E-02
LOC1053756	-147.511	58.720	-2.512	2.18E-02	-3.099	0.812	-3.815	1.27E-03	3.879	1.372	2.827	1.12E-02
IGLJ3	587.341	188.256	3.120	5.92E-03	0.665	2.604	0.255	8.01E-01	-12.427	4.399	-2.825	1.12E-02
MIR5695	425.289	154.984	2.744	1.33E-02	3.790	2.144	1.768	9.40E-02	-10.224	3.621	-2.823	1.13E-02
MMP24.AS1.	-17.811	7.108	-2.506	2.20E-02	-0.397	0.098	-4.034	7.78E-04	0.468	0.166	2.817	1.14E-02
UBOX5.AS1	145.472	45.985	3.163	5.38E-03	-0.057	0.636	-0.090	9.29E-01	-3.026	1.074	-2.816	1.14E-02
LINC01494	199.168	68.620	2.902	9.49E-03	0.575	0.949	0.606	5.52E-01	-4.515	1.603	-2.816	1.14E-02
LOC1053749	254.385	82.228	3.094	6.27E-03	0.320	1.137	0.281	7.82E-01	-5.408	1.921	-2.815	1.15E-02
OR14J1_5	19.231	6.204	3.100	6.18E-03	0.015	0.086	0.179	8.60E-01	-0.408	0.145	-2.815	1.15E-02
KIAA0100	88.223	33.330	2.647	1.64E-02	1.004	0.461	2.178	4.29E-02	-2.189	0.779	-2.811	1.16E-02
LINC01730	-161.823	56.541	-2.862	1.04E-02	-0.781	0.782	-0.998	3.31E-01	3.711	1.321	2.809	1.16E-02
KRTAP6.3	207.278	69.190	2.996	7.76E-03	0.503	0.957	0.525	6.06E-01	-4.535	1.617	-2.805	1.17E-02
HNRNPCL3	9.004	2.852	3.157	5.45E-03	0.000	0.039	0.000	1.00E+00	-0.187	0.067	-2.803	1.18E-02
PGAM1P5	72.600	26.053	2.787	1.22E-02	0.453	0.360	1.257	2.25E-01	-1.706	0.609	-2.803	1.18E-02
PRSS1	40.167	13.196	3.044	6.98E-03	0.074	0.183	0.405	6.90E-01	-0.863	0.308	-2.799	1.19E-02
ZCCHC18	52.537	17.488	3.004	7.62E-03	0.194	0.242	0.802	4.33E-01	-1.141	0.409	-2.793	1.20E-02
GMCL1	157.193	53.899	2.916	9.21E-03	0.687	0.746	0.922	3.69E-01	-3.515	1.259	-2.791	1.21E-02
PITPNM1	102.321	37.389	2.737	1.36E-02	0.690	0.517	1.335	1.99E-01	-2.438	0.874	-2.791	1.21E-02
LOC1001287	-80.652	30.007	-2.688	1.50E-02	-0.758	0.415	-1.827	8.44E-02	1.956	0.701	2.790	1.21E-02
SLX1A.SULT1	10.479	3.624	2.891	9.72E-03	0.054	0.050	1.074	2.97E-01	-0.236	0.085	-2.788	1.21E-02
LOC1079839	-92.154	38.217	-2.411	2.68E-02	-2.284	0.529	-4.321	4.12E-04	2.489	0.893	2.788	1.22E-02
SPEF2	77.346	22.693	3.408	3.13E-03	-0.463	0.314	-1.476	1.57E-01	-1.477	0.530	-2.786	1.22E-02
LOC1053729	134.126	43.012	3.118	5.94E-03	0.034	0.595	0.057	9.55E-01	-2.799	1.005	-2.785	1.22E-02
LOC1079853	-256.550	90.917	-2.822	1.13E-02	-1.005	1.258	-0.799	4.35E-01	5.903	2.124	2.779	1.24E-02
KIF2B	101.324	35.595	2.847	1.07E-02	0.389	0.492	0.789	4.40E-01	-2.308	0.832	-2.775	1.25E-02
LOC1079849	123.629	39.088	3.163	5.38E-03	-0.238	0.541	-0.439	6.66E-01	-2.530	0.913	-2.770	1.26E-02
LOC1079857	58.166	19.739	2.947	8.63E-03	-0.035	0.273	-0.130	8.98E-01	-1.276	0.461	-2.767	1.27E-02
MIR4314	-182.257	78.032	-2.336	3.13E-02	-5.020	1.079	-4.651	1.99E-04	5.020	1.823	2.753	1.31E-02
MIR3927	-118.082	50.556	-2.336	3.13E-02	-3.252	0.699	-4.651	1.99E-04	3.252	1.181	2.753	1.31E-02
EFCAB12	82.890	29.410	2.818	1.14E-02	0.570	0.407	1.401	1.78E-01	-1.892	0.687	-2.753	1.31E-02

IGHEP1_1	15.736	5.181	3.037	7.09E-03	0.017	0.072	0.232	8.19E-01	-0.333	0.121	-2.752	1.31E-02
SNORD121A	194.124	62.043	3.129	5.80E-03	-0.250	0.858	-0.292	7.74E-01	-3.982	1.450	-2.747	1.32E-02
RARA.AS1	-17.326	7.607	-2.278	3.52E-02	-0.533	0.105	-5.066	8.04E-05	0.488	0.178	2.745	1.33E-02
LINC00578	-217.553	79.090	-2.751	1.32E-02	-1.085	1.094	-0.992	3.34E-01	5.064	1.848	2.740	1.34E-02
LOC1027238	34.967	12.331	2.836	1.10E-02	0.199	0.171	1.168	2.58E-01	-0.789	0.288	-2.740	1.35E-02
LOC1053786	257.803	84.858	3.038	7.07E-03	0.166	1.174	0.142	8.89E-01	-5.429	1.983	-2.738	1.35E-02
LOC1079842	-68.146	26.626	-2.559	1.97E-02	-0.959	0.368	-2.604	1.79E-02	1.703	0.622	2.738	1.35E-02
GGCT	133.934	40.717	3.289	4.08E-03	-0.559	0.563	-0.992	3.34E-01	-2.604	0.951	-2.737	1.35E-02
LOC1053743	-106.481	44.292	-2.404	2.72E-02	-2.360	0.613	-3.852	1.17E-03	2.832	1.035	2.736	1.36E-02
GSE1	36.647	15.226	2.407	2.70E-02	0.468	0.211	2.224	3.92E-02	-0.973	0.356	-2.735	1.36E-02
SLC25A32	81.276	29.620	2.744	1.33E-02	0.366	0.410	0.894	3.83E-01	-1.892	0.692	-2.734	1.36E-02
SYNC	32.507	12.904	2.519	2.14E-02	0.355	0.178	1.991	6.19E-02	-0.823	0.302	-2.731	1.37E-02
LL22NC01.81	95.289	33.195	2.871	1.02E-02	0.209	0.459	0.456	6.54E-01	-2.112	0.776	-2.723	1.39E-02
LOC1079851	125.104	48.091	2.601	1.80E-02	0.886	0.665	1.332	2.00E-01	-3.058	1.124	-2.722	1.40E-02
MIR193B	536.016	169.857	3.156	5.47E-03	-1.175	2.349	-0.500	6.23E-01	-10.799	3.969	-2.721	1.40E-02
LOC1053761	-104.997	46.925	-2.238	3.81E-02	-3.068	0.649	-4.727	1.68E-04	2.983	1.096	2.720	1.40E-02
RBMX2	102.127	34.072	2.997	7.73E-03	-0.121	0.471	-0.257	8.00E-01	-2.163	0.796	-2.717	1.41E-02
CACNG6	102.944	38.173	2.697	1.48E-02	0.669	0.528	1.267	2.21E-01	-2.418	0.892	-2.711	1.43E-02
FAM197Y9	198.304	62.685	3.163	5.38E-03	-0.575	0.867	-0.663	5.16E-01	-3.969	1.465	-2.710	1.43E-02
LOC1053754	146.640	55.034	2.665	1.58E-02	0.837	0.761	1.099	2.86E-01	-3.484	1.286	-2.709	1.44E-02
LINC02039	198.110	64.660	3.064	6.69E-03	-0.424	0.894	-0.474	6.42E-01	-4.092	1.511	-2.708	1.44E-02
LOC1053784	-93.636	34.575	-2.708	1.44E-02	-0.623	0.478	-1.303	2.09E-01	2.186	0.808	2.706	1.45E-02
LOC1053724	-3.206	1.290	-2.485	2.30E-02	-0.062	0.018	-3.471	2.72E-03	0.082	0.030	2.704	1.45E-02
HPF1	181.622	67.212	2.702	1.46E-02	0.988	0.930	1.063	3.02E-01	-4.244	1.570	-2.703	1.46E-02
HLA.C_3	60.026	20.557	2.920	9.14E-03	0.141	0.284	0.495	6.27E-01	-1.297	0.480	-2.700	1.46E-02
BIN1	106.194	36.010	2.949	8.58E-03	0.059	0.498	0.119	9.07E-01	-2.270	0.841	-2.698	1.47E-02
TCL1B	129.867	45.598	2.848	1.07E-02	0.249	0.631	0.395	6.98E-01	-2.872	1.065	-2.696	1.48E-02
MIR5692A1	831.467	323.075	2.574	1.91E-02	9.232	4.469	2.066	5.35E-02	-20.339	7.549	-2.694	1.48E-02
DENND3	44.495	17.017	2.615	1.75E-02	0.240	0.235	1.018	3.22E-01	-1.070	0.398	-2.692	1.49E-02
LINC02027	329.984	116.153	2.841	1.08E-02	1.406	1.607	0.875	3.93E-01	-7.305	2.714	-2.692	1.49E-02
LINC01058	-181.588	73.167	-2.482	2.32E-02	-3.331	1.012	-3.292	4.05E-03	4.600	1.710	2.691	1.49E-02
MIR3149	299.450	98.966	3.026	7.27E-03	0.000	1.369	0.000	1.00E+00	-6.217	2.312	-2.689	1.50E-02
ABHD13	86.385	29.695	2.909	9.36E-03	0.134	0.411	0.327	7.47E-01	-1.865	0.694	-2.688	1.50E-02
TAF9B	114.209	41.950	2.722	1.40E-02	0.654	0.580	1.126	2.75E-01	-2.635	0.980	-2.688	1.50E-02
LOC1027248	801.501	265.929	3.014	7.46E-03	0.060	3.678	0.016	9.87E-01	-16.675	6.213	-2.684	1.52E-02
BAIAP3	52.341	20.434	2.561	1.96E-02	0.402	0.283	1.421	1.73E-01	-1.280	0.477	-2.680	1.53E-02
LOC1122683	-8.864	3.709	-2.390	2.80E-02	-0.187	0.051	-3.642	1.86E-03	0.232	0.087	2.678	1.53E-02
LINC00337	122.933	40.781	3.014	7.45E-03	0.013	0.564	0.023	9.82E-01	-2.550	0.953	-2.676	1.54E-02
IGLV10.67	94.005	35.929	2.616	1.75E-02	0.767	0.497	1.544	1.40E-01	-2.246	0.839	-2.675	1.54E-02
BARX2	-138.794	53.645	-2.587	1.86E-02	-0.984	0.742	-1.326	2.02E-01	3.350	1.253	2.673	1.55E-02
LOC1019296	-22.898	8.679	-2.639	1.67E-02	-0.065	0.120	-0.540	5.96E-01	0.542	0.203	2.672	1.55E-02
CPEB1.AS1	67.541	23.373	2.890	9.76E-03	0.063	0.323	0.195	8.47E-01	-1.459	0.546	-2.671	1.56E-02
LOC1079846	150.146	52.302	2.871	1.02E-02	0.274	0.723	0.378	7.10E-01	-3.262	1.222	-2.670	1.56E-02
LOC1079842	108.869	38.380	2.837	1.09E-02	0.385	0.531	0.726	4.77E-01	-2.394	0.897	-2.669	1.56E-02
RDH8	95.432	33.845	2.820	1.13E-02	0.252	0.468	0.538	5.97E-01	-2.110	0.791	-2.669	1.57E-02
MIR5197	664.716	221.386	3.003	7.64E-03	-0.296	3.062	-0.097	9.24E-01	-13.802	5.173	-2.668	1.57E-02
ABCA12	103.761	38.308	2.709	1.44E-02	0.349	0.530	0.658	5.19E-01	-2.387	0.895	-2.667	1.57E-02
LINC01978	61.970	21.503	2.882	9.93E-03	0.048	0.297	0.162	8.73E-01	-1.339	0.502	-2.666	1.58E-02
LINC02624	-168.876	62.891	-2.685	1.51E-02	-0.867	0.870	-0.997	3.32E-01	3.916	1.469	2.665	1.58E-02
LOC1053727	120.052	42.417	2.830	1.11E-02	0.212	0.587	0.362	7.22E-01	-2.640	0.991	-2.663	1.58E-02
NECTIN3.AS1	-381.438	142.560	-2.676	1.54E-02	-0.609	1.972	-0.309	7.61E-01	8.869	3.331	2.663	1.59E-02
MIR1197	-68.246	32.013	-2.132	4.71E-02	-2.342	0.443	-5.290	4.98E-05	1.991	0.748	2.662	1.59E-02
ZNF345	-167.542	63.295	-2.647	1.64E-02	-0.539	0.875	-0.615	5.46E-01	3.933	1.479	2.660	1.60E-02

LINC01151	-611.461	224.014	-2.730	1.38E-02	-2.448	3.099	-0.790	4.40E-01	13.921	5.234	2.660	1.60E-02
LOC1019273	-408.392	149.589	-2.730	1.37E-02	-1.006	2.069	-0.486	6.33E-01	9.278	3.495	2.655	1.61E-02
AKIRIN1	113.126	36.335	3.113	6.00E-03	-0.423	0.503	-0.841	4.11E-01	-2.253	0.849	-2.654	1.61E-02
TARM1	22.956	7.766	2.956	8.45E-03	0.004	0.107	0.033	9.74E-01	-0.481	0.181	-2.654	1.62E-02
MIR4477B	2136.573	707.261	3.021	7.34E-03	-3.353	9.783	-0.343	7.36E-01	-43.804	16.525	-2.651	1.63E-02
TCF21	66.392	22.338	2.972	8.16E-03	-0.003	0.309	-0.011	9.92E-01	-1.383	0.522	-2.650	1.63E-02
BCDIN3D.AS1	85.651	30.317	2.825	1.12E-02	0.253	0.419	0.604	5.53E-01	-1.877	0.708	-2.650	1.63E-02
S100A10	105.600	35.210	2.999	7.70E-03	-0.163	0.487	-0.335	7.41E-01	-2.179	0.823	-2.649	1.63E-02
DDX27	118.348	44.786	2.642	1.65E-02	0.586	0.619	0.947	3.56E-01	-2.771	1.046	-2.648	1.64E-02
ZNF806	81.444	27.545	2.957	8.44E-03	-0.088	0.381	-0.231	8.20E-01	-1.703	0.644	-2.646	1.64E-02
TMEM265	163.141	59.575	2.738	1.35E-02	0.882	0.824	1.070	2.99E-01	-3.681	1.392	-2.644	1.65E-02
LOC1053701	-729.944	264.199	-2.763	1.28E-02	0.414	3.654	0.113	9.11E-01	16.322	6.173	2.644	1.65E-02
IP6K3	61.419	20.603	2.981	8.01E-03	-0.007	0.285	-0.024	9.81E-01	-1.272	0.481	-2.642	1.65E-02
SCTR.AS1	-135.519	50.661	-2.675	1.54E-02	-0.669	0.701	-0.955	3.52E-01	3.124	1.184	2.639	1.67E-02
FLT3	85.235	34.055	2.503	2.22E-02	0.438	0.471	0.930	3.65E-01	-2.098	0.796	-2.637	1.68E-02
BTN2A1	84.328	32.902	2.563	1.96E-02	0.549	0.455	1.206	2.44E-01	-2.027	0.769	-2.636	1.68E-02
OR5F1	-371.104	136.424	-2.720	1.40E-02	-0.689	1.887	-0.365	7.19E-01	8.399	3.187	2.635	1.68E-02
TRBV11.1_1	-28.305	12.671	-2.234	3.84E-02	-0.780	0.175	-4.449	3.10E-04	0.780	0.296	2.634	1.69E-02
LOC1005068	-84.138	40.510	-2.077	5.24E-02	-3.169	0.560	-5.656	2.30E-05	2.492	0.946	2.632	1.69E-02
MIR4297	235.654	79.644	2.959	8.40E-03	-0.172	1.102	-0.156	8.78E-01	-4.896	1.861	-2.631	1.69E-02
SNORA66	307.048	101.439	3.027	7.25E-03	-0.543	1.403	-0.387	7.03E-01	-6.233	2.370	-2.630	1.70E-02
LOC1122682	414.369	136.438	3.037	7.09E-03	-0.180	1.887	-0.095	9.25E-01	-8.383	3.188	-2.630	1.70E-02
TTLL10.AS1	61.120	24.415	2.503	2.22E-02	0.513	0.338	1.518	1.46E-01	-1.499	0.570	-2.627	1.71E-02
KRTAP9.4_1	-26.563	11.842	-2.243	3.77E-02	-0.823	0.164	-5.022	8.85E-05	0.727	0.277	2.627	1.71E-02
PRAMEF19	133.527	53.772	2.483	2.31E-02	1.164	0.744	1.566	1.35E-01	-3.300	1.256	-2.627	1.71E-02
TBCE_1	2.928	0.982	2.981	8.01E-03	-0.003	0.014	-0.195	8.48E-01	-0.060	0.023	-2.626	1.71E-02
TMEFF2	69.428	23.729	2.926	9.03E-03	-0.183	0.328	-0.556	5.85E-01	-1.455	0.554	-2.624	1.72E-02
LOC1079873	174.142	59.340	2.935	8.86E-03	0.052	0.821	0.063	9.50E-01	-3.638	1.386	-2.624	1.72E-02
MIR30E	-134.322	62.341	-2.155	4.50E-02	-4.157	0.862	-4.821	1.37E-04	3.821	1.457	2.624	1.72E-02
LINC01894	95.160	36.568	2.602	1.80E-02	0.522	0.506	1.033	3.15E-01	-2.240	0.854	-2.622	1.73E-02
ERCC4	81.933	27.625	2.966	8.28E-03	-0.144	0.382	-0.377	7.10E-01	-1.689	0.645	-2.617	1.75E-02
BTN1A1	139.363	47.288	2.947	8.62E-03	0.011	0.654	0.016	9.87E-01	-2.888	1.105	-2.614	1.76E-02
LOC1053784	78.573	28.515	2.756	1.30E-02	0.274	0.394	0.694	4.97E-01	-1.741	0.666	-2.613	1.76E-02
LOC1053719	78.004	26.509	2.943	8.71E-03	0.012	0.367	0.033	9.74E-01	-1.618	0.619	-2.613	1.76E-02
LOC1079873	175.426	70.220	2.498	2.24E-02	1.601	0.971	1.648	1.17E-01	-4.278	1.641	-2.608	1.78E-02
SNORD46	427.836	145.688	2.937	8.82E-03	-0.320	2.015	-0.159	8.76E-01	-8.876	3.404	-2.608	1.78E-02
SNORD63	536.976	183.278	2.930	8.95E-03	0.000	2.535	0.000	1.00E+00	-11.155	4.282	-2.605	1.79E-02
OR2J3	15.365	5.343	2.876	1.01E-02	0.004	0.074	0.052	9.59E-01	-0.325	0.125	-2.603	1.80E-02
MIR181A1	274.909	113.942	2.413	2.67E-02	3.426	1.576	2.174	4.33E-02	-6.928	2.662	-2.602	1.80E-02
ERP29	74.537	30.160	2.471	2.37E-02	0.702	0.417	1.682	1.10E-01	-1.834	0.705	-2.602	1.80E-02
LOC1079863	-63.901	23.471	-2.723	1.40E-02	0.002	0.325	0.006	9.96E-01	1.426	0.548	2.601	1.81E-02
VPS52_5	-2.897	1.235	-2.345	3.07E-02	-0.075	0.017	-4.391	3.52E-04	0.075	0.029	2.599	1.81E-02
LOC1053701	-436.379	163.560	-2.668	1.57E-02	0.026	2.262	0.012	9.91E-01	9.930	3.822	2.598	1.82E-02
XRCC5	-303.019	110.729	-2.737	1.36E-02	0.055	1.532	0.036	9.72E-01	6.720	2.587	2.598	1.82E-02
DNAH3	-72.017	25.704	-2.802	1.18E-02	0.054	0.356	0.153	8.80E-01	1.560	0.601	2.597	1.82E-02
LOC1079865	-364.533	145.196	-2.511	2.18E-02	-2.533	2.008	-1.261	2.23E-01	8.810	3.392	2.597	1.82E-02
LOC1053766	-99.328	37.956	-2.617	1.75E-02	-0.455	0.525	-0.866	3.98E-01	2.301	0.887	2.594	1.83E-02
ABI1	78.301	28.510	2.746	1.33E-02	0.123	0.394	0.311	7.60E-01	-1.728	0.666	-2.594	1.83E-02
TMEM183B	-103.559	40.171	-2.578	1.90E-02	-0.689	0.556	-1.240	2.31E-01	2.434	0.939	2.594	1.83E-02
LOC1053795	-27.536	11.526	-2.389	2.81E-02	-0.427	0.159	-2.676	1.54E-02	0.698	0.269	2.593	1.84E-02
TRA.CGC5.1_	-213.401	89.330	-2.389	2.81E-02	-3.307	1.236	-2.676	1.54E-02	5.411	2.087	2.593	1.84E-02
MIR4268	1174.850	420.305	2.795	1.20E-02	2.717	5.814	0.467	6.46E-01	-25.457	9.820	-2.592	1.84E-02
LOC1122682	68.776	25.188	2.731	1.37E-02	0.083	0.348	0.237	8.15E-01	-1.525	0.589	-2.592	1.84E-02

IL18RAP	74.543	28.105	2.652	1.62E-02	0.227	0.389	0.584	5.67E-01	-1.701	0.657	-2.591	1.85E-02
PTPMT1	66.408	25.908	2.563	1.96E-02	0.585	0.358	1.632	1.20E-01	-1.568	0.605	-2.590	1.85E-02
LOC1053706	-577.942	212.350	-2.722	1.40E-02	0.136	2.937	0.046	9.64E-01	12.843	4.961	2.588	1.85E-02
RDH12	-110.696	44.534	-2.486	2.30E-02	-1.057	0.616	-1.715	1.03E-01	2.693	1.041	2.588	1.86E-02
TRV.CAC1.5	639.414	255.109	2.506	2.20E-02	6.137	3.529	1.739	9.91E-02	-15.421	5.961	-2.587	1.86E-02
LOC1053754	-206.648	79.452	-2.601	1.81E-02	-0.330	1.099	-0.301	7.67E-01	4.802	1.856	2.587	1.86E-02
ULK4P1	-33.119	14.576	-2.272	3.56E-02	-0.757	0.202	-3.753	1.46E-03	0.880	0.341	2.585	1.87E-02
LOC1053786	127.622	46.526	2.743	1.34E-02	0.365	0.644	0.568	5.77E-01	-2.809	1.087	-2.584	1.87E-02
DHDH	71.852	29.546	2.432	2.57E-02	0.606	0.409	1.482	1.56E-01	-1.784	0.690	-2.584	1.87E-02
CCP110	70.488	26.603	2.650	1.63E-02	0.155	0.368	0.420	6.79E-01	-1.606	0.622	-2.583	1.87E-02
MGAT5	72.945	30.327	2.405	2.71E-02	0.530	0.419	1.263	2.23E-01	-1.830	0.709	-2.583	1.88E-02
POP1	-239.103	92.267	-2.591	1.84E-02	-0.407	1.276	-0.319	7.54E-01	5.566	2.156	2.582	1.88E-02
LOC1053739	-107.658	41.056	-2.622	1.73E-02	-0.317	0.568	-0.559	5.83E-01	2.474	0.959	2.579	1.89E-02
C13orf46_1	14.867	5.092	2.920	9.15E-03	-0.009	0.070	-0.122	9.04E-01	-0.307	0.119	-2.578	1.90E-02
LOC1053718	-18.582	8.720	-2.131	4.71E-02	-0.568	0.121	-4.712	1.74E-04	0.525	0.204	2.577	1.90E-02
MIR4780	737.243	250.216	2.946	8.63E-03	-0.041	3.461	-0.012	9.91E-01	-15.060	5.846	-2.576	1.90E-02
OR4K1	471.450	164.080	2.873	1.01E-02	-0.703	2.270	-0.310	7.60E-01	-9.874	3.834	-2.576	1.90E-02
LOC1027250	-122.024	57.063	-2.138	4.64E-02	-3.277	0.789	-4.152	5.99E-04	3.433	1.333	2.575	1.91E-02
LOC1019287	33.396	13.083	2.553	2.00E-02	0.280	0.181	1.545	1.40E-01	-0.787	0.306	-2.575	1.91E-02
LOC1027240	-641.717	245.444	-2.615	1.76E-02	-0.622	3.395	-0.183	8.57E-01	14.762	5.735	2.574	1.91E-02
ZDHHC13	-177.682	70.797	-2.510	2.19E-02	-1.565	0.979	-1.599	1.27E-01	4.256	1.654	2.573	1.92E-02
MIR378E	-310.254	131.188	-2.365	2.95E-02	-4.430	1.815	-2.441	2.52E-02	7.886	3.065	2.573	1.92E-02
LOC1053735	-51.138	23.379	-2.187	4.22E-02	-1.577	0.323	-4.876	1.21E-04	1.405	0.546	2.572	1.92E-02
TRUB2	69.978	24.051	2.910	9.35E-03	-0.038	0.333	-0.115	9.10E-01	-1.444	0.562	-2.570	1.93E-02
LINC02644	178.229	64.266	2.773	1.25E-02	0.350	0.889	0.394	6.98E-01	-3.859	1.502	-2.570	1.93E-02
LOC1053706	-98.050	40.174	-2.441	2.52E-02	-1.413	0.556	-2.542	2.04E-02	2.411	0.939	2.569	1.93E-02
LOC1079851	-48.225	23.553	-2.048	5.55E-02	-1.639	0.326	-5.032	8.67E-05	1.413	0.550	2.567	1.94E-02
LOC1079856	99.239	34.838	2.849	1.07E-02	-0.037	0.482	-0.076	9.40E-01	-2.090	0.814	-2.567	1.94E-02
EPM2AIP1	49.248	19.967	2.466	2.39E-02	0.280	0.276	1.013	3.24E-01	-1.198	0.467	-2.567	1.94E-02
PRPF40A	67.452	26.585	2.537	2.06E-02	0.308	0.368	0.838	4.13E-01	-1.594	0.621	-2.566	1.94E-02
TMEM158	62.526	22.442	2.786	1.22E-02	0.022	0.310	0.071	9.44E-01	-1.345	0.524	-2.566	1.95E-02
LOC1053765	-349.806	135.472	-2.582	1.88E-02	-0.838	1.874	-0.447	6.60E-01	8.119	3.165	2.565	1.95E-02
AVIL	75.659	25.704	2.943	8.69E-03	-0.137	0.356	-0.387	7.04E-01	-1.540	0.601	-2.564	1.95E-02
ROR1	-112.646	42.863	-2.628	1.71E-02	-0.294	0.593	-0.496	6.26E-01	2.566	1.001	2.562	1.96E-02
TRAJ26	-127.870	62.988	-2.030	5.74E-02	-4.271	0.871	-4.902	1.15E-04	3.771	1.472	2.562	1.96E-02
LOC1053695	260.481	95.155	2.737	1.35E-02	0.701	1.316	0.532	6.01E-01	-5.693	2.223	-2.561	1.96E-02
LOC1053742	-228.493	92.571	-2.468	2.38E-02	-1.607	1.280	-1.255	2.25E-01	5.534	2.163	2.559	1.97E-02
ESYT3	-53.684	22.014	-2.439	2.53E-02	-0.371	0.304	-1.219	2.38E-01	1.315	0.514	2.557	1.98E-02
KRT75	-228.351	90.158	-2.533	2.08E-02	-0.773	1.247	-0.620	5.43E-01	5.385	2.107	2.556	1.98E-02
MARK1	85.808	33.339	2.574	1.91E-02	0.447	0.461	0.970	3.45E-01	-1.991	0.779	-2.556	1.99E-02
MIR1286	547.752	226.763	2.416	2.66E-02	6.282	3.137	2.003	6.05E-02	-13.538	5.298	-2.555	1.99E-02
C5AR2	-80.217	32.429	-2.474	2.36E-02	-0.517	0.449	-1.153	2.64E-01	1.935	0.758	2.554	1.99E-02
PYDC1	210.169	84.723	2.481	2.32E-02	1.822	1.172	1.555	1.37E-01	-5.052	1.980	-2.552	2.00E-02
RAB27B	66.665	25.513	2.613	1.76E-02	0.271	0.353	0.768	4.52E-01	-1.521	0.596	-2.551	2.01E-02
LINC01522	115.213	38.158	3.019	7.37E-03	-0.592	0.528	-1.121	2.77E-01	-2.273	0.892	-2.550	2.01E-02
LRRC19	113.678	40.859	2.782	1.23E-02	0.202	0.565	0.357	7.25E-01	-2.431	0.955	-2.547	2.02E-02
LOC1053731	-120.383	44.562	-2.701	1.46E-02	-0.148	0.616	-0.240	8.13E-01	2.650	1.041	2.545	2.03E-02
MIR548AN	-114.279	55.973	-2.042	5.61E-02	-4.073	0.774	-5.260	5.30E-05	3.328	1.308	2.545	2.03E-02
LOC1053753	18.710	7.859	2.381	2.85E-02	0.202	0.109	1.857	7.98E-02	-0.467	0.184	-2.544	2.03E-02
PLA2G1B	274.721	99.745	2.754	1.31E-02	1.233	1.380	0.893	3.83E-01	-5.929	2.331	-2.544	2.03E-02
RPS18P9	-200.117	76.829	-2.605	1.79E-02	-1.368	1.063	-1.288	2.14E-01	4.564	1.795	2.543	2.04E-02
LOC1079859	18.548	6.601	2.810	1.16E-02	-0.002	0.091	-0.027	9.79E-01	-0.392	0.154	-2.543	2.04E-02
LRMDA	103.956	40.346	2.577	1.90E-02	0.422	0.558	0.756	4.60E-01	-2.395	0.943	-2.541	2.05E-02

LRG1	80.936	27.098	2.987	7.91E-03	-0.293	0.375	-0.782	4.44E-01	-1.608	0.633	-2.540	2.05E-02
CDRT15P3_1	32.961	11.373	2.898	9.58E-03	-0.046	0.157	-0.293	7.73E-01	-0.675	0.266	-2.539	2.06E-02
LUNAR1	124.606	41.586	2.996	7.75E-03	-0.477	0.575	-0.829	4.18E-01	-2.467	0.972	-2.538	2.06E-02
DACT1	103.431	36.500	2.834	1.10E-02	-0.009	0.505	-0.017	9.86E-01	-2.165	0.853	-2.538	2.06E-02
LINC00244	199.392	76.920	2.592	1.84E-02	1.024	1.064	0.962	3.49E-01	-4.557	1.797	-2.535	2.07E-02
TRP.AGG2.6	677.788	234.816	2.886	9.83E-03	0.000	3.248	0.000	1.00E+00	-13.908	5.486	-2.535	2.07E-02
SNORA50B	-207.109	93.256	-2.221	3.94E-02	-4.629	1.290	-3.589	2.10E-03	5.523	2.179	2.535	2.07E-02
MAS1	42.435	17.150	2.474	2.35E-02	0.081	0.237	0.340	7.37E-01	-1.015	0.401	-2.533	2.08E-02
ANKRD34C	82.497	34.738	2.375	2.89E-02	0.898	0.480	1.869	7.80E-02	-2.056	0.812	-2.533	2.08E-02
TRAJ46	1263.717	442.260	2.857	1.05E-02	-0.473	6.117	-0.077	9.39E-01	-26.176	10.333	-2.533	2.08E-02
NCR3LG1	-70.081	28.157	-2.489	2.28E-02	-0.558	0.389	-1.432	1.69E-01	1.666	0.658	2.533	2.08E-02
MIR186	1343.274	499.433	2.690	1.50E-02	2.949	6.908	0.427	6.75E-01	-29.548	11.669	-2.532	2.09E-02
LINC00545	220.260	78.067	2.821	1.13E-02	0.060	1.080	0.056	9.56E-01	-4.618	1.824	-2.532	2.09E-02
LOC1079851	75.398	29.521	2.554	1.99E-02	0.443	0.408	1.085	2.92E-01	-1.746	0.690	-2.532	2.09E-02
MIR3146	-489.044	209.099	-2.339	3.11E-02	-7.636	2.892	-2.640	1.66E-02	12.359	4.886	2.530	2.10E-02
LOC1079863	-80.160	32.243	-2.486	2.30E-02	-0.573	0.446	-1.284	2.15E-01	1.905	0.753	2.528	2.10E-02
LOC1053761	81.311	29.503	2.756	1.30E-02	0.014	0.408	0.035	9.73E-01	-1.743	0.689	-2.528	2.10E-02
PRPH2	-301.953	113.514	-2.660	1.59E-02	0.309	1.570	0.197	8.46E-01	6.701	2.652	2.527	2.11E-02
SREBF2	-110.907	42.914	-2.584	1.87E-02	-0.411	0.594	-0.692	4.98E-01	2.532	1.003	2.526	2.11E-02
RPA1	116.696	44.809	2.604	1.79E-02	0.374	0.620	0.603	5.54E-01	-2.643	1.047	-2.525	2.12E-02
LOC1079853	98.292	35.923	2.736	1.36E-02	0.201	0.497	0.404	6.91E-01	-2.114	0.839	-2.518	2.15E-02
PROSER3	-134.469	51.928	-2.590	1.85E-02	-0.215	0.718	-0.299	7.68E-01	3.054	1.213	2.517	2.15E-02
SCARNA8	-209.948	93.794	-2.238	3.81E-02	-5.514	1.297	-4.250	4.82E-04	5.514	2.191	2.516	2.16E-02
HBA1	163.502	60.577	2.699	1.47E-02	0.365	0.838	0.435	6.68E-01	-3.560	1.415	-2.515	2.16E-02
LOC1079860	163.193	56.690	2.879	1.00E-02	-0.546	0.784	-0.696	4.95E-01	-3.330	1.325	-2.514	2.17E-02
LINC02057	-80.934	38.028	-2.128	4.74E-02	-1.973	0.526	-3.751	1.46E-03	2.232	0.889	2.512	2.18E-02
RASSF9	-195.806	72.974	-2.683	1.52E-02	-0.256	1.009	-0.254	8.03E-01	4.282	1.705	2.511	2.18E-02
LOC1079841	82.623	35.506	2.327	3.18E-02	0.923	0.491	1.879	7.65E-02	-2.081	0.830	-2.508	2.19E-02
C11orf49	57.304	24.121	2.376	2.88E-02	0.323	0.334	0.969	3.45E-01	-1.413	0.564	-2.508	2.19E-02
LINC02360	-452.958	175.786	-2.577	1.90E-02	-0.911	2.431	-0.375	7.12E-01	10.295	4.107	2.507	2.20E-02
LOC1027239	121.336	42.809	2.834	1.10E-02	-0.090	0.592	-0.152	8.81E-01	-2.507	1.000	-2.507	2.20E-02
NDFIP2.AS1	-34.501	17.012	-2.028	5.76E-02	-1.139	0.235	-4.841	1.31E-04	0.996	0.397	2.505	2.21E-02
FBXW8	70.036	27.425	2.554	1.99E-02	0.282	0.379	0.744	4.67E-01	-1.605	0.641	-2.505	2.21E-02
VTRNA1.3	-77.377	35.861	-2.158	4.47E-02	-2.098	0.496	-4.229	5.05E-04	2.098	0.838	2.504	2.21E-02
RNF225	-15.327	7.036	-2.178	4.29E-02	-0.411	0.097	-4.227	5.07E-04	0.411	0.164	2.503	2.22E-02
LOC1079848	160.774	55.927	2.875	1.01E-02	-0.315	0.774	-0.407	6.89E-01	-3.270	1.307	-2.502	2.22E-02
PHRF1_1	-10.792	4.584	-2.354	3.01E-02	-0.140	0.063	-2.212	4.02E-02	0.268	0.107	2.501	2.22E-02
ZNF589	-125.198	48.313	-2.591	1.84E-02	-0.154	0.668	-0.230	8.21E-01	2.822	1.129	2.500	2.23E-02
SLCO5A1	-391.336	151.377	-2.585	1.87E-02	0.189	2.094	0.090	9.29E-01	8.839	3.537	2.499	2.24E-02
LOC1053776	48.151	16.500	2.918	9.18E-03	-0.096	0.228	-0.422	6.78E-01	-0.963	0.386	-2.498	2.24E-02
SMIM2.AS1	101.898	38.254	2.664	1.58E-02	0.098	0.529	0.185	8.55E-01	-2.232	0.894	-2.497	2.24E-02
FSD1	94.275	40.394	2.334	3.14E-02	0.824	0.559	1.475	1.58E-01	-2.357	0.944	-2.497	2.25E-02
LOC1053718	-123.340	55.182	-2.235	3.83E-02	-2.353	0.763	-3.082	6.43E-03	3.219	1.289	2.496	2.25E-02
LPL	-210.313	87.737	-2.397	2.76E-02	-0.666	1.214	-0.549	5.90E-01	5.115	2.050	2.495	2.25E-02
LOC1079855	134.357	47.587	2.823	1.13E-02	-0.154	0.658	-0.234	8.17E-01	-2.774	1.112	-2.495	2.25E-02
LOC1079840	125.299	51.704	2.423	2.61E-02	1.019	0.715	1.425	1.71E-01	-3.012	1.208	-2.494	2.26E-02
DEFB127	-110.567	53.238	-2.077	5.24E-02	-2.932	0.736	-3.982	8.74E-04	3.101	1.244	2.493	2.26E-02
SUCLG2.AS1	103.095	40.333	2.556	1.98E-02	0.353	0.558	0.633	5.35E-01	-2.349	0.942	-2.493	2.26E-02
C1orf54	296.817	117.187	2.533	2.08E-02	1.709	1.621	1.055	3.06E-01	-6.824	2.738	-2.492	2.27E-02
FAM155A	-155.197	58.576	-2.649	1.63E-02	0.236	0.810	0.291	7.74E-01	3.407	1.369	2.490	2.28E-02
CAPN5	-84.774	33.406	-2.538	2.06E-02	-0.065	0.462	-0.142	8.89E-01	1.943	0.781	2.489	2.28E-02
CXCR4	-103.972	40.497	-2.567	1.94E-02	-0.351	0.560	-0.626	5.39E-01	2.355	0.946	2.489	2.28E-02
IGFL1	313.623	107.101	2.928	8.98E-03	-0.434	1.481	-0.293	7.73E-01	-6.228	2.502	-2.489	2.28E-02

LOC1053755	-84.942	33.530	-2.533	2.08E-02	-0.576	0.464	-1.241	2.30E-01	1.947	0.783	2.486	2.30E-02
RBM19	113.178	44.529	2.542	2.05E-02	0.475	0.616	0.771	4.51E-01	-2.586	1.040	-2.485	2.30E-02
LOC1053702	152.962	57.063	2.681	1.53E-02	0.256	0.789	0.325	7.49E-01	-3.313	1.333	-2.485	2.30E-02
RETNLB	138.163	49.250	2.805	1.17E-02	-0.121	0.681	-0.178	8.61E-01	-2.859	1.151	-2.485	2.30E-02
LOC1053753	-213.648	84.387	-2.532	2.09E-02	-1.424	1.167	-1.220	2.38E-01	4.899	1.972	2.485	2.30E-02
PRR30	130.368	49.736	2.621	1.73E-02	0.578	0.688	0.841	4.11E-01	-2.885	1.162	-2.483	2.31E-02
LOC1053732	-264.167	113.783	-2.322	3.22E-02	-2.612	1.574	-1.660	1.14E-01	6.598	2.659	2.482	2.32E-02
VPS13D	55.495	24.932	2.226	3.90E-02	0.386	0.345	1.118	2.78E-01	-1.444	0.583	-2.479	2.33E-02
MIR660	-93.919	43.134	-2.177	4.30E-02	-2.499	0.597	-4.188	5.53E-04	2.499	1.008	2.479	2.33E-02
LOC1053762	105.908	37.054	2.858	1.04E-02	-0.308	0.513	-0.600	5.56E-01	-2.144	0.866	-2.477	2.34E-02
LOC1079860	-172.445	68.262	-2.526	2.11E-02	-0.723	0.944	-0.766	4.54E-01	3.949	1.595	2.476	2.34E-02
ABCC11	-148.933	58.051	-2.566	1.95E-02	0.053	0.803	0.066	9.48E-01	3.357	1.356	2.475	2.35E-02
SLC37A1	47.490	19.474	2.439	2.53E-02	0.305	0.269	1.131	2.73E-01	-1.125	0.455	-2.472	2.36E-02
LOC1053775	124.705	50.214	2.483	2.31E-02	0.772	0.695	1.111	2.81E-01	-2.896	1.173	-2.469	2.38E-02
SNORA25B	248.151	89.461	2.774	1.25E-02	-0.177	1.237	-0.143	8.88E-01	-5.159	2.090	-2.468	2.38E-02
FGF12.AS2	125.533	44.340	2.831	1.11E-02	-0.162	0.613	-0.264	7.95E-01	-2.554	1.036	-2.465	2.40E-02
SLC25A40	-108.025	41.844	-2.582	1.88E-02	-0.382	0.579	-0.660	5.17E-01	2.409	0.978	2.464	2.40E-02
LOC1009962	-32.055	14.993	-2.138	4.65E-02	-0.935	0.207	-4.510	2.71E-04	0.863	0.350	2.463	2.41E-02
LOC1053741	76.923	30.724	2.504	2.21E-02	0.197	0.425	0.463	6.49E-01	-1.768	0.718	-2.463	2.41E-02
SDCBP2.AS1	79.407	29.465	2.695	1.48E-02	0.107	0.408	0.263	7.96E-01	-1.695	0.688	-2.462	2.41E-02
ZNF826P	-513.797	198.618	-2.587	1.86E-02	-0.679	2.747	-0.247	8.08E-01	11.427	4.641	2.462	2.41E-02
ANKRD61	167.554	64.046	2.616	1.75E-02	0.451	0.886	0.509	6.17E-01	-3.685	1.496	-2.462	2.41E-02
MIR4296	795.979	284.137	2.801	1.18E-02	-0.908	3.930	-0.231	8.20E-01	-16.332	6.639	-2.460	2.42E-02
LINC01632	3.555	1.394	2.551	2.00E-02	0.014	0.019	0.710	4.87E-01	-0.080	0.033	-2.459	2.43E-02
MIS18A	76.775	31.436	2.442	2.51E-02	0.404	0.435	0.928	3.66E-01	-1.806	0.734	-2.459	2.43E-02
LOC1053740	-104.847	43.881	-2.389	2.80E-02	-0.596	0.607	-0.981	3.39E-01	2.520	1.025	2.458	2.43E-02
DDN.AS1	-14.717	7.195	-2.046	5.57E-02	-0.401	0.100	-4.031	7.83E-04	0.413	0.168	2.457	2.44E-02
ANKRD36C	108.942	47.647	2.286	3.46E-02	1.109	0.659	1.682	1.10E-01	-2.733	1.113	-2.455	2.45E-02
LOC1019295	258.746	90.934	2.845	1.07E-02	-0.031	1.258	-0.024	9.81E-01	-5.215	2.125	-2.455	2.45E-02
LOC1079859	-82.348	32.912	-2.502	2.22E-02	-0.173	0.455	-0.380	7.08E-01	1.886	0.769	2.453	2.46E-02
LOC1079843	122.759	45.232	2.714	1.42E-02	0.214	0.626	0.343	7.36E-01	-2.592	1.057	-2.452	2.46E-02
LOC1053703	115.783	44.716	2.589	1.85E-02	0.257	0.619	0.415	6.83E-01	-2.562	1.045	-2.452	2.46E-02
LINC00583	-127.262	61.520	-2.069	5.33E-02	-4.006	0.851	-4.708	1.75E-04	3.523	1.437	2.451	2.47E-02
CROT	-100.068	40.562	-2.467	2.39E-02	-0.464	0.561	-0.827	4.19E-01	2.323	0.948	2.451	2.47E-02
CDKAL1	68.035	30.089	2.261	3.64E-02	0.671	0.416	1.612	1.24E-01	-1.723	0.703	-2.450	2.47E-02
LOC1053773	78.272	31.573	2.479	2.33E-02	0.311	0.437	0.712	4.86E-01	-1.807	0.738	-2.450	2.48E-02
LOC1079862	193.677	100.509	1.927	6.99E-02	3.726	1.390	2.680	1.53E-02	-5.751	2.348	-2.449	2.48E-02
B3GAT3	-222.010	89.930	-2.469	2.38E-02	-0.718	1.244	-0.577	5.71E-01	5.144	2.101	2.448	2.48E-02
LOC1122682	-36.859	17.545	-2.101	5.00E-02	-0.872	0.243	-3.595	2.07E-03	1.003	0.410	2.448	2.48E-02
TRC.GCA10.1	1560.140	557.496	2.798	1.19E-02	-0.451	7.711	-0.059	9.54E-01	-31.875	13.026	-2.447	2.49E-02
ABCC8	-54.674	23.436	-2.333	3.15E-02	-0.251	0.324	-0.776	4.48E-01	1.339	0.548	2.446	2.49E-02
NRGN	135.634	60.970	2.225	3.91E-02	1.962	0.843	2.327	3.19E-02	-3.483	1.425	-2.445	2.50E-02
LOC1079853	182.863	76.513	2.390	2.80E-02	1.405	1.058	1.327	2.01E-01	-4.371	1.788	-2.445	2.50E-02
NRK	68.752	25.777	2.667	1.57E-02	-0.096	0.357	-0.268	7.91E-01	-1.472	0.602	-2.445	2.50E-02
UNC13A	-87.819	35.616	-2.466	2.40E-02	-0.256	0.493	-0.520	6.09E-01	2.033	0.832	2.443	2.51E-02
LOC1005065	-142.150	57.947	-2.453	2.46E-02	-0.614	0.802	-0.766	4.54E-01	3.307	1.354	2.442	2.51E-02
LOC1053777	43.045	15.890	2.709	1.44E-02	0.024	0.220	0.107	9.16E-01	-0.907	0.371	-2.442	2.51E-02
CLEC17A	79.999	28.978	2.761	1.29E-02	-0.233	0.401	-0.582	5.68E-01	-1.653	0.677	-2.442	2.52E-02
NSD3	75.491	31.900	2.366	2.94E-02	0.484	0.441	1.096	2.88E-01	-1.820	0.745	-2.441	2.52E-02
CDRT15	-40.776	19.152	-2.129	4.73E-02	-0.876	0.265	-3.306	3.93E-03	1.092	0.447	2.441	2.52E-02
FOXC2.AS1	49.502	18.334	2.700	1.47E-02	0.030	0.254	0.120	9.06E-01	-1.044	0.428	-2.438	2.54E-02
EDA2R	-143.047	56.125	-2.549	2.02E-02	-0.340	0.776	-0.437	6.67E-01	3.197	1.311	2.438	2.54E-02
LOC1079861	64.299	26.126	2.461	2.42E-02	0.160	0.361	0.443	6.63E-01	-1.487	0.610	-2.437	2.54E-02

SLC39A4	-10.645	4.469	-2.382	2.85E-02	-0.093	0.062	-1.502	1.50E-01	0.254	0.104	2.436	2.55E-02
LOC1002887	-108.429	47.582	-2.279	3.51E-02	-1.643	0.658	-2.497	2.25E-02	2.706	1.112	2.434	2.56E-02
TSBP1_7	-15.653	6.653	-2.353	3.02E-02	-0.197	0.092	-2.146	4.58E-02	0.378	0.155	2.434	2.56E-02
TRG.CCC3.1	708.068	256.315	2.762	1.28E-02	-0.668	3.545	-0.188	8.53E-01	-14.572	5.989	-2.433	2.56E-02
LINC00933	140.637	51.096	2.752	1.31E-02	-0.203	0.707	-0.287	7.78E-01	-2.899	1.194	-2.428	2.59E-02
IL20RA	70.998	30.450	2.332	3.15E-02	0.455	0.421	1.081	2.94E-01	-1.727	0.711	-2.428	2.59E-02
B3GALT1	60.277	26.444	2.279	3.51E-02	0.265	0.366	0.726	4.77E-01	-1.500	0.618	-2.428	2.59E-02
HLA.DQA2_5	29.068	10.373	2.802	1.18E-02	-0.065	0.143	-0.451	6.57E-01	-0.588	0.242	-2.427	2.60E-02
LOC1053720	-935.549	371.900	-2.516	2.16E-02	-1.240	5.144	-0.241	8.12E-01	21.080	8.689	2.426	2.60E-02
RBMXL2	77.257	30.595	2.525	2.12E-02	0.337	0.423	0.797	4.36E-01	-1.734	0.715	-2.426	2.60E-02
IGHV3.21	11.010	4.043	2.724	1.39E-02	0.000	0.056	0.000	1.00E+00	-0.229	0.094	-2.425	2.61E-02
IGHV3.48	11.010	4.043	2.724	1.39E-02	0.000	0.056	0.000	1.00E+00	-0.229	0.094	-2.425	2.61E-02
IGHV3.52	11.010	4.043	2.724	1.39E-02	0.000	0.056	0.000	1.00E+00	-0.229	0.094	-2.425	2.61E-02
IGHV3.64	11.010	4.043	2.724	1.39E-02	0.000	0.056	0.000	1.00E+00	-0.229	0.094	-2.425	2.61E-02
PRAMEF34P_	1.756	0.645	2.724	1.39E-02	0.000	0.009	0.000	1.00E+00	-0.037	0.015	-2.425	2.61E-02
KIR2DL2_10	12.245	4.496	2.724	1.39E-02	0.000	0.062	0.000	1.00E+00	-0.255	0.105	-2.425	2.61E-02
MIR92B	80.973	29.730	2.724	1.39E-02	0.000	0.411	0.000	1.00E+00	-1.684	0.695	-2.425	2.61E-02
MIR664A	94.797	34.806	2.724	1.39E-02	0.000	0.481	0.000	1.00E+00	-1.972	0.813	-2.425	2.61E-02
MIR10B	35.333	12.973	2.724	1.39E-02	0.000	0.179	0.000	1.00E+00	-0.735	0.303	-2.425	2.61E-02
MIR4440	118.980	43.685	2.724	1.39E-02	0.000	0.604	0.000	1.00E+00	-2.475	1.021	-2.425	2.61E-02
EEFSEC	0.956	0.351	2.724	1.39E-02	0.000	0.005	0.000	1.00E+00	-0.020	0.008	-2.425	2.61E-02
LOC1079861	14.694	5.395	2.724	1.39E-02	0.000	0.075	0.000	1.00E+00	-0.306	0.126	-2.425	2.61E-02
RUFY1.AS1	3.954	1.452	2.724	1.39E-02	0.000	0.020	0.000	1.00E+00	-0.082	0.034	-2.425	2.61E-02
TRBV25.1	22.597	8.297	2.724	1.39E-02	0.000	0.115	0.000	1.00E+00	-0.470	0.194	-2.425	2.61E-02
LOC1053792	54.283	19.931	2.724	1.39E-02	0.000	0.276	0.000	1.00E+00	-1.129	0.466	-2.425	2.61E-02
MIR3926.1	53.242	19.549	2.724	1.39E-02	0.000	0.270	0.000	1.00E+00	-1.108	0.457	-2.425	2.61E-02
MIR4471	187.310	68.774	2.724	1.39E-02	0.000	0.951	0.000	1.00E+00	-3.897	1.607	-2.425	2.61E-02
MIR6845	254.864	93.578	2.724	1.39E-02	0.000	1.294	0.000	1.00E+00	-5.302	2.186	-2.425	2.61E-02
MIR3689A	49.829	18.296	2.724	1.39E-02	0.000	0.253	0.000	1.00E+00	-1.037	0.427	-2.425	2.61E-02
FNBP4	2.263	0.831	2.724	1.39E-02	0.000	0.011	0.000	1.00E+00	-0.047	0.019	-2.425	2.61E-02
MIR3167	91.451	33.578	2.724	1.39E-02	0.000	0.464	0.000	1.00E+00	-1.902	0.785	-2.425	2.61E-02
BLOC1S1.RDH	6.408	2.353	2.724	1.39E-02	0.000	0.033	0.000	1.00E+00	-0.133	0.055	-2.425	2.61E-02
TRAJ7	790.512	290.249	2.724	1.39E-02	0.000	4.015	0.000	1.00E+00	-16.445	6.782	-2.425	2.61E-02
IGHV4.30.2	21.835	8.017	2.724	1.39E-02	0.000	0.111	0.000	1.00E+00	-0.454	0.187	-2.425	2.61E-02
SNORD116.2	413.477	151.815	2.724	1.39E-02	0.000	2.100	0.000	1.00E+00	-8.601	3.547	-2.425	2.61E-02
TRH.GTG1.9	377.872	138.742	2.724	1.39E-02	0.000	1.919	0.000	1.00E+00	-7.861	3.242	-2.425	2.61E-02
SNORA78	306.038	112.367	2.724	1.39E-02	0.000	1.554	0.000	1.00E+00	-6.366	2.625	-2.425	2.61E-02
LOC388242	36.791	13.508	2.724	1.39E-02	0.000	0.187	0.000	1.00E+00	-0.765	0.316	-2.425	2.61E-02
MIR4730	51.141	18.777	2.724	1.39E-02	0.000	0.260	0.000	1.00E+00	-1.064	0.439	-2.425	2.61E-02
SNORD35B	132.501	48.650	2.724	1.39E-02	0.000	0.673	0.000	1.00E+00	-2.756	1.137	-2.425	2.61E-02
RDH13	0.660	0.242	2.724	1.39E-02	0.000	0.003	0.000	1.00E+00	-0.014	0.006	-2.425	2.61E-02
SHANK3	1.016	0.373	2.724	1.39E-02	0.000	0.005	0.000	1.00E+00	-0.021	0.009	-2.425	2.61E-02
MIR12129	409.124	150.217	2.724	1.39E-02	0.000	2.078	0.000	1.00E+00	-8.511	3.510	-2.425	2.61E-02
LOC1053772	2.805	1.030	2.724	1.39E-02	0.000	0.014	0.000	1.00E+00	-0.058	0.024	-2.425	2.61E-02
OR2A12_1	0.934	0.343	2.724	1.39E-02	0.000	0.005	0.000	1.00E+00	-0.019	0.008	-2.425	2.61E-02
SMG1P3_1	4.625	1.698	2.724	1.39E-02	0.000	0.023	0.000	1.00E+00	-0.096	0.040	-2.425	2.61E-02
LOC1053781	27.663	10.157	2.724	1.39E-02	0.000	0.140	0.000	1.00E+00	-0.575	0.237	-2.425	2.61E-02
LOC1053777	53.106	19.499	2.724	1.39E-02	0.000	0.270	0.000	1.00E+00	-1.105	0.456	-2.425	2.61E-02
ABCC6P1_1	14.667	5.385	2.724	1.39E-02	0.000	0.074	0.000	1.00E+00	-0.305	0.126	-2.425	2.61E-02
MIR378J_1	213.946	78.554	2.724	1.39E-02	0.000	1.087	0.000	1.00E+00	-4.451	1.835	-2.425	2.61E-02
LOC1019294	9.796	3.597	2.724	1.39E-02	0.000	0.050	0.000	1.00E+00	-0.204	0.084	-2.425	2.61E-02
CRHR1_1	2.961	1.087	2.724	1.39E-02	0.000	0.015	0.000	1.00E+00	-0.062	0.025	-2.425	2.61E-02
LENG8.AS1_1	4.357	1.600	2.724	1.39E-02	0.000	0.022	0.000	1.00E+00	-0.091	0.037	-2.425	2.61E-02

TBX22_1	3.172	1.164	2.724	1.39E-02	0.000	0.016	0.000	1.00E+00	-0.066	0.027	-2.425	2.61E-02
HLA.DQA1_4	12.300	4.516	2.724	1.39E-02	0.000	0.062	0.000	1.00E+00	-0.256	0.106	-2.425	2.61E-02
NOTCH4_5	5.676	2.084	2.724	1.39E-02	0.000	0.029	0.000	1.00E+00	-0.118	0.049	-2.425	2.61E-02
LOC10537965	32.799	12.043	2.724	1.39E-02	0.000	0.167	0.000	1.00E+00	-0.682	0.281	-2.425	2.61E-02
TRF.GAA1.1_	638.907	234.585	2.724	1.39E-02	0.000	3.245	0.000	1.00E+00	-13.291	5.481	-2.425	2.61E-02
LOC10029414	1.053	0.387	2.724	1.39E-02	0.000	0.005	0.000	1.00E+00	-0.022	0.009	-2.425	2.61E-02
LINC02028	-120.106	49.858	-2.409	2.69E-02	-0.497	0.690	-0.721	4.80E-01	2.824	1.165	2.424	2.61E-02
LMNTD2_1	54.611	20.922	2.610	1.77E-02	0.084	0.289	0.290	7.75E-01	-1.185	0.489	-2.423	2.61E-02
POPDC2	-84.964	37.624	-2.258	3.66E-02	-1.019	0.520	-1.958	6.59E-02	2.129	0.879	2.422	2.62E-02
NET1	86.368	37.193	2.322	3.22E-02	0.628	0.514	1.221	2.38E-01	-2.105	0.869	-2.422	2.62E-02
NPPA	126.194	48.326	2.611	1.77E-02	0.140	0.668	0.210	8.36E-01	-2.734	1.129	-2.421	2.63E-02
LOC10798602	166.558	65.461	2.544	2.03E-02	0.765	0.905	0.845	4.09E-01	-3.703	1.529	-2.421	2.63E-02
CLEC4A	94.892	37.949	2.501	2.23E-02	-0.009	0.525	-0.016	9.87E-01	-2.146	0.887	-2.420	2.63E-02
GREB1	51.062	18.831	2.712	1.43E-02	-0.201	0.260	-0.772	4.50E-01	-1.064	0.440	-2.419	2.64E-02
RPRD2	60.388	24.095	2.506	2.20E-02	0.168	0.333	0.505	6.20E-01	-1.362	0.563	-2.419	2.64E-02
TOMM40L	58.173	25.174	2.311	3.29E-02	0.447	0.348	1.284	2.15E-01	-1.423	0.588	-2.419	2.64E-02
CRBN	-138.757	58.488	-2.372	2.90E-02	-0.754	0.809	-0.932	3.64E-01	3.305	1.367	2.419	2.64E-02
LRRC4	76.770	27.820	2.760	1.29E-02	0.055	0.385	0.143	8.88E-01	-1.572	0.650	-2.418	2.64E-02
LOC10537516	86.096	32.191	2.675	1.55E-02	-0.015	0.445	-0.034	9.73E-01	-1.819	0.752	-2.418	2.64E-02
ZNF208	-110.521	44.315	-2.494	2.26E-02	-0.320	0.613	-0.522	6.08E-01	2.503	1.035	2.418	2.65E-02
TRBC1_1	22.335	8.380	2.665	1.58E-02	0.018	0.116	0.157	8.77E-01	-0.473	0.196	-2.417	2.65E-02
MIR149	133.758	50.185	2.665	1.58E-02	0.109	0.694	0.157	8.77E-01	-2.835	1.173	-2.417	2.65E-02
LOC10536989	86.393	33.868	2.551	2.01E-02	0.078	0.468	0.165	8.70E-01	-1.913	0.791	-2.417	2.65E-02
GRAMD1C	-126.591	52.048	-2.432	2.57E-02	-0.496	0.720	-0.688	5.00E-01	2.940	1.216	2.417	2.65E-02
GOLGA8M	-0.406	0.196	-2.070	5.32E-02	-0.011	0.003	-4.083	6.98E-04	0.011	0.005	2.417	2.65E-02
LOC10537667	142.271	51.840	2.744	1.33E-02	-0.216	0.717	-0.302	7.66E-01	-2.924	1.211	-2.414	2.66E-02
LOC11226823	227.403	109.704	2.073	5.28E-02	3.709	1.517	2.444	2.51E-02	-6.186	2.563	-2.414	2.67E-02
LINC01370	-302.156	125.910	-2.400	2.74E-02	-2.079	1.742	-1.194	2.48E-01	7.095	2.942	2.412	2.68E-02
LOC10537320	-38.642	19.415	-1.990	6.20E-02	-1.262	0.269	-4.701	1.78E-04	1.094	0.454	2.411	2.68E-02
AAR2	67.162	23.982	2.801	1.18E-02	-0.113	0.332	-0.342	7.36E-01	-1.351	0.560	-2.411	2.68E-02
LOC10537374	181.564	72.719	2.497	2.25E-02	0.853	1.006	0.848	4.08E-01	-4.095	1.699	-2.410	2.69E-02
LOC10537039	-142.250	55.451	-2.565	1.95E-02	0.186	0.767	0.243	8.11E-01	3.123	1.296	2.410	2.69E-02
LOC10050706	-111.770	46.819	-2.387	2.82E-02	-0.610	0.648	-0.942	3.59E-01	2.635	1.094	2.408	2.70E-02
RIMBP3B	-1.938	0.923	-2.099	5.02E-02	-0.052	0.013	-4.066	7.25E-04	0.052	0.022	2.407	2.70E-02
IGLC4	64.974	23.097	2.813	1.15E-02	-0.224	0.319	-0.700	4.93E-01	-1.299	0.540	-2.407	2.70E-02
LOC10798689	-123.928	55.286	-2.242	3.78E-02	-2.094	0.765	-2.738	1.35E-02	3.109	1.292	2.407	2.71E-02
GREB1L	75.501	32.937	2.292	3.42E-02	0.581	0.456	1.275	2.19E-01	-1.852	0.770	-2.406	2.71E-02
LINC02646	112.190	48.324	2.322	3.22E-02	0.896	0.668	1.340	1.97E-01	-2.717	1.129	-2.406	2.71E-02
FAF1	67.261	25.836	2.603	1.80E-02	0.126	0.357	0.354	7.28E-01	-1.452	0.604	-2.406	2.71E-02
QPCT	-371.130	153.282	-2.421	2.63E-02	-0.894	2.120	-0.422	6.78E-01	8.612	3.581	2.405	2.72E-02
GRHL1	-176.477	70.845	-2.491	2.27E-02	-0.145	0.980	-0.148	8.84E-01	3.979	1.655	2.404	2.72E-02
LOC10537694	-135.965	64.448	-2.110	4.91E-02	-2.947	0.891	-3.306	3.93E-03	3.620	1.506	2.404	2.72E-02
MEGF9	-97.527	40.645	-2.399	2.75E-02	-0.457	0.562	-0.812	4.27E-01	2.282	0.950	2.403	2.72E-02
FAM167A.AS	20.810	7.349	2.832	1.11E-02	-0.053	0.102	-0.523	6.07E-01	-0.413	0.172	-2.403	2.72E-02
MT2A	249.140	90.036	2.767	1.27E-02	-0.014	1.245	-0.011	9.91E-01	-5.050	2.104	-2.401	2.74E-02
LOC10537252	-183.478	74.806	-2.453	2.46E-02	-0.382	1.035	-0.369	7.17E-01	4.196	1.748	2.400	2.74E-02
LOC10798690	-4.175	2.208	-1.891	7.48E-02	-0.150	0.031	-4.910	1.13E-04	0.124	0.052	2.399	2.75E-02
COQ4	161.605	62.582	2.582	1.88E-02	0.282	0.866	0.326	7.49E-01	-3.508	1.462	-2.399	2.75E-02
LOC10798627	123.951	45.008	2.754	1.31E-02	-0.393	0.623	-0.631	5.36E-01	-2.522	1.052	-2.398	2.75E-02
LOC10537524	-112.886	48.113	-2.346	3.06E-02	-0.549	0.665	-0.825	4.20E-01	2.695	1.124	2.397	2.76E-02
TMEM87B	131.368	50.688	2.592	1.84E-02	0.487	0.701	0.695	4.96E-01	-2.839	1.184	-2.397	2.76E-02
LOC10537553	-451.126	186.131	-2.424	2.61E-02	-0.906	2.575	-0.352	7.29E-01	10.421	4.349	2.396	2.76E-02
STYXL2	69.158	26.357	2.624	1.72E-02	-0.124	0.365	-0.339	7.38E-01	-1.476	0.616	-2.396	2.76E-02

VWA1	-92.981	38.434	-2.419	2.64E-02	-0.364	0.532	-0.684	5.02E-01	2.151	0.898	2.396	2.77E-02
LOC1053737	155.258	60.831	2.552	2.00E-02	0.278	0.841	0.330	7.45E-01	-3.404	1.421	-2.395	2.77E-02
MIR7843	-377.462	196.442	-1.921	7.06E-02	-13.038	2.717	-4.799	1.44E-04	10.991	4.590	2.395	2.77E-02
SPOPL	83.631	35.703	2.342	3.09E-02	0.523	0.494	1.059	3.04E-01	-1.998	0.834	-2.395	2.77E-02
LOC1053795	-7.745	3.761	-2.059	5.42E-02	-0.210	0.052	-4.044	7.61E-04	0.210	0.088	2.394	2.78E-02
MIR4493	-95.063	46.161	-2.059	5.42E-02	-2.582	0.638	-4.044	7.61E-04	2.582	1.079	2.394	2.78E-02
IGKV2.40_1	-9.559	4.641	-2.059	5.42E-02	-0.260	0.064	-4.044	7.61E-04	0.260	0.108	2.394	2.78E-02
AP2A2_2	-4.750	2.306	-2.059	5.42E-02	-0.129	0.032	-4.044	7.61E-04	0.129	0.054	2.394	2.78E-02
MIR622	-216.863	105.304	-2.059	5.42E-02	-5.891	1.457	-4.044	7.61E-04	5.891	2.460	2.394	2.78E-02
LOC1053711	-4.819	2.340	-2.059	5.42E-02	-0.131	0.032	-4.044	7.61E-04	0.131	0.055	2.394	2.78E-02
NTMT1	-28.048	13.168	-2.130	4.72E-02	-0.517	0.182	-2.838	1.09E-02	0.737	0.308	2.394	2.78E-02
LOC1053725	-165.735	70.084	-2.365	2.95E-02	-1.119	0.969	-1.154	2.63E-01	3.920	1.637	2.394	2.78E-02
IFIT5	70.445	26.604	2.648	1.64E-02	0.004	0.368	0.011	9.91E-01	-1.488	0.622	-2.394	2.78E-02
LOC1122684	73.239	27.376	2.675	1.54E-02	0.013	0.379	0.034	9.74E-01	-1.531	0.640	-2.393	2.78E-02
PDZD4	46.497	21.102	2.203	4.08E-02	0.558	0.292	1.912	7.19E-02	-1.179	0.493	-2.392	2.79E-02
LOC1053737	480.527	179.821	2.672	1.55E-02	0.430	2.487	0.173	8.65E-01	-10.045	4.201	-2.391	2.79E-02
SLC35A2	-146.515	59.612	-2.458	2.43E-02	-0.294	0.825	-0.357	7.25E-01	3.325	1.393	2.388	2.81E-02
MDH1	228.355	95.578	2.389	2.80E-02	1.091	1.322	0.826	4.20E-01	-5.327	2.233	-2.385	2.83E-02
PCDH18	-112.341	47.369	-2.372	2.91E-02	-0.468	0.655	-0.714	4.84E-01	2.640	1.107	2.385	2.83E-02
HGD	-257.715	117.137	-2.200	4.11E-02	-3.460	1.620	-2.135	4.67E-02	6.520	2.737	2.382	2.84E-02
ZZZ3	-172.091	71.005	-2.424	2.61E-02	-0.478	0.982	-0.486	6.33E-01	3.952	1.659	2.382	2.85E-02
ALS2	65.548	25.299	2.591	1.84E-02	-0.055	0.350	-0.156	8.77E-01	-1.408	0.591	-2.382	2.85E-02
LINC00491	-249.555	99.107	-2.518	2.15E-02	-0.259	1.371	-0.189	8.52E-01	5.515	2.316	2.382	2.85E-02
ARHGEF1	-163.036	69.069	-2.360	2.97E-02	-0.466	0.955	-0.488	6.32E-01	3.842	1.614	2.381	2.85E-02
C4BPB	-232.461	92.887	-2.503	2.22E-02	0.121	1.285	0.095	9.26E-01	5.167	2.170	2.381	2.85E-02
C20orf141	-25.217	10.999	-2.293	3.41E-02	-0.343	0.152	-2.252	3.71E-02	0.612	0.257	2.380	2.86E-02
TNFAIP3	55.844	24.275	2.301	3.36E-02	0.409	0.336	1.219	2.39E-01	-1.350	0.567	-2.380	2.86E-02
ST6GALNAC4	-175.142	70.341	-2.490	2.28E-02	-0.013	0.973	-0.014	9.89E-01	3.911	1.643	2.380	2.86E-02
CLCA4	122.333	43.888	2.787	1.22E-02	-0.452	0.607	-0.745	4.66E-01	-2.439	1.025	-2.379	2.86E-02
LOC1005062	133.527	56.888	2.347	3.06E-02	0.937	0.787	1.191	2.49E-01	-3.162	1.329	-2.379	2.86E-02
LOC1079850	-40.037	20.730	-1.931	6.93E-02	-1.365	0.287	-4.760	1.57E-04	1.152	0.484	2.379	2.86E-02
MIR6500	-73.786	36.324	-2.031	5.73E-02	-2.019	0.502	-4.018	8.07E-04	2.019	0.849	2.379	2.87E-02
TRBV19	-36.893	18.162	-2.031	5.73E-02	-1.009	0.251	-4.018	8.07E-04	1.009	0.424	2.379	2.87E-02
MIR589	-128.194	63.108	-2.031	5.73E-02	-3.507	0.873	-4.018	8.07E-04	3.507	1.474	2.379	2.87E-02
LOC1053739	105.814	40.358	2.622	1.73E-02	0.077	0.558	0.137	8.92E-01	-2.243	0.943	-2.378	2.87E-02
PDE4DIPP7	-45.555	19.430	-2.345	3.07E-02	-0.198	0.269	-0.735	4.72E-01	1.079	0.454	2.378	2.87E-02
AQP7	50.561	18.920	2.672	1.55E-02	-0.126	0.262	-0.483	6.35E-01	-1.051	0.442	-2.377	2.87E-02
LOC1019281	152.343	58.491	2.605	1.79E-02	0.210	0.809	0.259	7.98E-01	-3.246	1.367	-2.376	2.88E-02
C9orf62	-107.185	43.053	-2.490	2.28E-02	-0.026	0.596	-0.043	9.66E-01	2.390	1.006	2.375	2.88E-02
OR8B3	77.442	36.740	2.108	4.93E-02	0.978	0.508	1.925	7.02E-02	-2.039	0.858	-2.375	2.89E-02
LOC1079866	-1181.542	485.044	-2.436	2.55E-02	-2.115	6.709	-0.315	7.56E-01	26.898	11.333	2.373	2.90E-02
TAMALIN.AS1	-7.984	4.494	-1.776	9.26E-02	-0.351	0.062	-5.641	2.37E-05	0.249	0.105	2.373	2.90E-02
SNORD160	1048.571	392.244	2.673	1.55E-02	-0.408	5.425	-0.075	9.41E-01	-21.744	9.165	-2.373	2.90E-02
LINC01186	-37.252	19.119	-1.948	6.71E-02	-1.195	0.264	-4.519	2.66E-04	1.060	0.447	2.372	2.90E-02
LOC1079870	5.671	2.197	2.581	1.88E-02	0.009	0.030	0.304	7.64E-01	-0.122	0.051	-2.372	2.90E-02
LOC1053741	-109.044	53.359	-2.044	5.59E-02	-2.437	0.738	-3.303	3.96E-03	2.956	1.247	2.371	2.91E-02
LOC1053708	-313.993	140.059	-2.242	3.78E-02	-2.468	1.937	-1.274	2.19E-01	7.748	3.272	2.368	2.93E-02
ZNF785	56.470	26.052	2.168	4.38E-02	0.599	0.360	1.662	1.14E-01	-1.441	0.609	-2.367	2.93E-02
LOC1027247	63.242	24.914	2.538	2.06E-02	-0.060	0.345	-0.173	8.64E-01	-1.378	0.582	-2.367	2.93E-02
MIR325	699.359	261.382	2.676	1.54E-02	0.000	3.615	0.000	1.00E+00	-14.450	6.107	-2.366	2.94E-02
TSC22D1	-111.673	44.073	-2.534	2.08E-02	0.147	0.610	0.240	8.13E-01	2.435	1.030	2.365	2.95E-02
LOC1053774	162.087	62.992	2.573	1.91E-02	0.246	0.871	0.282	7.81E-01	-3.480	1.472	-2.365	2.95E-02
FAM71F1	-167.699	70.229	-2.388	2.81E-02	-0.472	0.971	-0.486	6.33E-01	3.880	1.641	2.364	2.95E-02

LOC284950	-373.156	156.183	-2.389	2.80E-02	-0.764	2.160	-0.354	7.28E-01	8.625	3.649	2.364	2.95E-02
LOC10537849	400.421	165.469	2.420	2.63E-02	3.125	2.289	1.366	1.89E-01	-9.138	3.866	-2.364	2.95E-02
PTPRQ	75.238	29.617	2.540	2.05E-02	-0.052	0.410	-0.126	9.01E-01	-1.636	0.692	-2.363	2.96E-02
OR6C76	-123.561	57.740	-2.140	4.63E-02	-2.010	0.799	-2.516	2.16E-02	3.186	1.349	2.362	2.97E-02
DENND6A.DT	-89.764	37.989	-2.363	2.96E-02	-0.795	0.525	-1.513	1.48E-01	2.096	0.888	2.361	2.97E-02
LINC01052	275.547	96.107	2.867	1.02E-02	-0.984	1.329	-0.740	4.69E-01	-5.302	2.246	-2.361	2.97E-02
TRBV11.2_1	-76.436	39.238	-1.948	6.72E-02	-2.322	0.543	-4.278	4.53E-04	2.164	0.917	2.360	2.98E-02
OMP	-126.683	56.462	-2.244	3.77E-02	-1.445	0.781	-1.850	8.08E-02	3.113	1.319	2.359	2.98E-02
PIWIL2	77.539	30.785	2.519	2.15E-02	0.300	0.426	0.706	4.89E-01	-1.697	0.719	-2.359	2.98E-02
IMP3	74.620	31.623	2.360	2.98E-02	0.452	0.437	1.034	3.15E-01	-1.743	0.739	-2.359	2.98E-02
CEBPB	71.920	28.587	2.516	2.16E-02	0.218	0.395	0.552	5.88E-01	-1.576	0.668	-2.359	2.98E-02
MAP3K20	77.672	35.122	2.212	4.02E-02	0.668	0.486	1.376	1.86E-01	-1.935	0.821	-2.358	2.99E-02
MIR4324	423.604	159.366	2.658	1.60E-02	-0.207	2.204	-0.094	9.26E-01	-8.777	3.724	-2.357	2.99E-02
MUC15	-161.871	67.862	-2.385	2.83E-02	-0.452	0.939	-0.482	6.36E-01	3.736	1.586	2.356	3.00E-02
FAM167A.AS	43.525	16.007	2.719	1.41E-02	-0.080	0.221	-0.360	7.23E-01	-0.881	0.374	-2.356	3.00E-02
LOC10537643	170.235	65.758	2.589	1.85E-02	0.094	0.910	0.104	9.19E-01	-3.619	1.536	-2.355	3.01E-02
LINC01525	179.791	65.956	2.726	1.39E-02	-0.640	0.912	-0.701	4.92E-01	-3.627	1.541	-2.354	3.01E-02
LOC10537497	351.325	130.016	2.702	1.46E-02	0.530	1.798	0.294	7.72E-01	-7.146	3.038	-2.352	3.02E-02
LINC02113	101.923	40.856	2.495	2.26E-02	0.270	0.565	0.478	6.38E-01	-2.245	0.955	-2.352	3.02E-02
FAM74A3	-49.545	23.759	-2.085	5.15E-02	-1.052	0.329	-3.200	4.96E-03	1.305	0.555	2.352	3.03E-02
RHOU_1	-2.874	1.219	-2.359	2.99E-02	-0.015	0.017	-0.885	3.88E-01	0.067	0.028	2.348	3.05E-02
RNU7.1	857.967	406.138	2.112	4.89E-02	13.103	5.618	2.332	3.15E-02	-22.274	9.489	-2.347	3.06E-02
MOGAT2	96.082	44.611	2.154	4.51E-02	1.089	0.617	1.765	9.45E-02	-2.446	1.042	-2.347	3.06E-02
GALNT5	86.916	39.157	2.220	3.95E-02	0.751	0.542	1.387	1.82E-01	-2.146	0.915	-2.346	3.06E-02
LINC00260	-331.130	143.080	-2.314	3.27E-02	-2.237	1.979	-1.130	2.73E-01	7.838	3.343	2.345	3.07E-02
CA5BP1	65.823	28.329	2.324	3.21E-02	0.487	0.392	1.243	2.30E-01	-1.552	0.662	-2.344	3.07E-02
LOC10192799	70.874	27.520	2.575	1.91E-02	0.068	0.381	0.179	8.60E-01	-1.507	0.643	-2.344	3.07E-02
LOC10798743	40.710	15.225	2.674	1.55E-02	-0.103	0.211	-0.489	6.31E-01	-0.834	0.356	-2.344	3.08E-02
CRCT1	217.557	95.774	2.272	3.56E-02	2.254	1.325	1.701	1.06E-01	-5.245	2.238	-2.344	3.08E-02
LOC10798579	-19.078	10.002	-1.907	7.25E-02	-0.627	0.138	-4.530	2.59E-04	0.548	0.234	2.343	3.08E-02
LOC10537780	52.710	20.923	2.519	2.14E-02	0.112	0.289	0.387	7.03E-01	-1.145	0.489	-2.343	3.08E-02
MIR5047	277.025	104.764	2.644	1.65E-02	0.158	1.449	0.109	9.14E-01	-5.735	2.448	-2.343	3.08E-02
PRODH2	-405.217	170.181	-2.381	2.85E-02	-1.090	2.354	-0.463	6.49E-01	9.311	3.976	2.342	3.09E-02
LOC10798540	-194.099	79.855	-2.431	2.58E-02	-0.189	1.105	-0.171	8.66E-01	4.366	1.866	2.340	3.10E-02
LINC00924	145.447	58.606	2.482	2.32E-02	0.262	0.811	0.323	7.50E-01	-3.202	1.369	-2.338	3.11E-02
MIR7156	-259.451	130.150	-1.993	6.16E-02	-7.110	1.800	-3.949	9.40E-04	7.110	3.041	2.338	3.11E-02
LOC10537810	108.875	42.327	2.572	1.92E-02	0.011	0.585	0.019	9.85E-01	-2.310	0.989	-2.336	3.12E-02
LOC10537383	137.827	66.204	2.082	5.19E-02	2.069	0.916	2.259	3.65E-02	-3.614	1.547	-2.336	3.12E-02
LOC10050655	-132.626	54.450	-2.436	2.55E-02	0.044	0.753	0.059	9.54E-01	2.970	1.272	2.335	3.13E-02
LOC10192719	91.375	36.692	2.490	2.28E-02	0.184	0.508	0.362	7.22E-01	-2.001	0.857	-2.335	3.14E-02
LOC10798697	-238.788	104.113	-2.294	3.41E-02	-1.929	1.440	-1.339	1.97E-01	5.678	2.433	2.334	3.14E-02
GJB6	-270.372	108.605	-2.489	2.28E-02	-0.279	1.502	-0.186	8.55E-01	5.920	2.538	2.333	3.14E-02
FAM193A	75.012	32.302	2.322	3.21E-02	0.473	0.447	1.058	3.04E-01	-1.761	0.755	-2.333	3.15E-02
NDUFA2	-51.210	22.308	-2.296	3.39E-02	-0.597	0.309	-1.935	6.89E-02	1.216	0.521	2.333	3.15E-02
HNRNPAB	-275.877	115.482	-2.389	2.81E-02	-0.750	1.597	-0.469	6.44E-01	6.294	2.698	2.333	3.15E-02
C1orf52	47.318	19.632	2.410	2.69E-02	-0.049	0.272	-0.181	8.59E-01	-1.069	0.459	-2.331	3.16E-02
LINC01543	-269.568	121.574	-2.217	3.97E-02	-1.192	1.682	-0.709	4.87E-01	6.620	2.841	2.330	3.16E-02
RCSD1	59.220	23.450	2.525	2.12E-02	0.073	0.324	0.225	8.24E-01	-1.277	0.548	-2.330	3.16E-02
PPP1R2B	-266.748	113.432	-2.352	3.03E-02	-0.406	1.569	-0.259	7.99E-01	6.175	2.650	2.330	3.16E-02
MIR6801	287.672	109.359	2.631	1.70E-02	-0.188	1.513	-0.125	9.02E-01	-5.952	2.555	-2.330	3.17E-02
OR10P1	-321.450	132.163	-2.432	2.57E-02	-0.259	1.828	-0.142	8.89E-01	7.191	3.088	2.329	3.17E-02
LOC10798490	167.193	66.224	2.525	2.12E-02	0.033	0.916	0.036	9.72E-01	-3.602	1.547	-2.328	3.18E-02
LOC10192834	-484.954	203.015	-2.389	2.81E-02	-0.586	2.808	-0.209	8.37E-01	11.041	4.743	2.328	3.18E-02

BTNL9	79.998	31.002	2.580	1.89E-02	-0.106	0.429	-0.246	8.08E-01	-1.685	0.724	-2.327	3.19E-02
LOC10537585	-184.471	78.721	-2.343	3.08E-02	-0.827	1.089	-0.759	4.57E-01	4.278	1.839	2.326	3.19E-02
RTL1	-130.668	55.669	-2.347	3.06E-02	-0.262	0.770	-0.340	7.38E-01	3.026	1.301	2.326	3.19E-02
SLC44A3	137.071	55.813	2.456	2.44E-02	0.393	0.772	0.509	6.17E-01	-3.031	1.304	-2.324	3.20E-02
LOC10537440	-633.770	260.001	-2.438	2.54E-02	-0.122	3.596	-0.034	9.73E-01	14.114	6.075	2.323	3.21E-02
MIR1243	516.055	198.601	2.598	1.82E-02	-0.241	2.747	-0.088	9.31E-01	-10.780	4.640	-2.323	3.21E-02
LOC10537569	179.759	70.339	2.556	1.99E-02	0.125	0.973	0.129	8.99E-01	-3.818	1.643	-2.323	3.21E-02
SNORD115.11	551.247	209.078	2.637	1.68E-02	-0.484	2.892	-0.167	8.69E-01	-11.348	4.885	-2.323	3.21E-02
MIR5705	507.891	192.634	2.637	1.68E-02	-0.446	2.665	-0.167	8.69E-01	-10.456	4.501	-2.323	3.21E-02
SNORA8	120.255	45.035	2.670	1.56E-02	-0.328	0.623	-0.526	6.05E-01	-2.443	1.052	-2.322	3.22E-02
LOC11226814	91.957	39.170	2.348	3.05E-02	0.348	0.542	0.642	5.29E-01	-2.125	0.915	-2.322	3.22E-02
OR1B1	68.907	26.017	2.649	1.63E-02	-0.302	0.360	-0.839	4.12E-01	-1.411	0.608	-2.321	3.22E-02
PELATON	129.398	49.479	2.615	1.75E-02	-0.014	0.684	-0.021	9.84E-01	-2.683	1.156	-2.320	3.23E-02
CDK2AP1	-289.603	120.510	-2.403	2.73E-02	-0.278	1.667	-0.167	8.70E-01	6.526	2.816	2.318	3.24E-02
TRPM2.AS	69.253	29.408	2.355	3.01E-02	0.328	0.407	0.807	4.30E-01	-1.592	0.687	-2.317	3.25E-02
LOC11226824	-128.002	53.057	-2.413	2.67E-02	-0.355	0.734	-0.484	6.34E-01	2.871	1.240	2.316	3.25E-02
LOC10537796	355.389	139.996	2.539	2.06E-02	-0.021	1.936	-0.011	9.92E-01	-7.576	3.271	-2.316	3.26E-02
LOC10798663	-335.293	137.755	-2.434	2.56E-02	0.452	1.905	0.237	8.15E-01	7.448	3.219	2.314	3.27E-02
MIR548S	503.849	191.697	2.628	1.71E-02	-0.484	2.652	-0.182	8.57E-01	-10.362	4.479	-2.314	3.27E-02
MIR135A2	272.660	101.393	2.689	1.50E-02	-0.726	1.402	-0.518	6.11E-01	-5.479	2.369	-2.313	3.28E-02
ZMYM3	48.248	20.311	2.375	2.88E-02	0.239	0.281	0.850	4.06E-01	-1.097	0.475	-2.312	3.28E-02
ZNF812P	-180.209	78.920	-2.283	3.48E-02	-1.347	1.092	-1.234	2.33E-01	4.262	1.844	2.312	3.28E-02
PARP6	112.964	45.485	2.484	2.31E-02	0.279	0.629	0.444	6.62E-01	-2.456	1.063	-2.311	3.29E-02
LOC10192744	-86.336	36.295	-2.379	2.86E-02	-0.379	0.502	-0.755	4.60E-01	1.959	0.848	2.310	3.30E-02
MIR548A1	81.131	29.224	2.776	1.25E-02	-0.409	0.404	-1.012	3.25E-01	-1.576	0.683	-2.309	3.30E-02
GMPPA	112.578	47.432	2.373	2.90E-02	0.506	0.656	0.771	4.51E-01	-2.558	1.108	-2.309	3.31E-02
ZNF487	105.088	43.097	2.438	2.53E-02	0.361	0.596	0.606	5.52E-01	-2.324	1.007	-2.308	3.31E-02
LOC10537132	305.973	116.676	2.622	1.73E-02	-0.272	1.614	-0.169	8.68E-01	-6.293	2.726	-2.308	3.31E-02
LOC10798675	14.351	5.679	2.527	2.11E-02	0.015	0.079	0.187	8.53E-01	-0.306	0.133	-2.308	3.31E-02
LOC10250342	196.567	86.838	2.264	3.62E-02	1.401	1.201	1.167	2.59E-01	-4.683	2.029	-2.308	3.31E-02
LINC01508	-501.474	213.213	-2.352	3.03E-02	-1.302	2.949	-0.441	6.64E-01	11.497	4.982	2.308	3.31E-02
LOC10537074	127.534	56.118	2.273	3.55E-02	0.792	0.776	1.020	3.21E-01	-3.025	1.311	-2.307	3.31E-02
OR7D2	-268.891	112.725	-2.385	2.83E-02	-0.577	1.559	-0.370	7.16E-01	6.074	2.634	2.306	3.32E-02
ZNF92	-126.264	54.408	-2.321	3.22E-02	-0.570	0.753	-0.757	4.59E-01	2.931	1.271	2.305	3.33E-02
FST	-122.108	52.260	-2.337	3.12E-02	-0.078	0.723	-0.108	9.15E-01	2.815	1.221	2.305	3.33E-02
ZNF792	-123.308	54.809	-2.250	3.72E-02	-0.611	0.758	-0.806	4.31E-01	2.952	1.281	2.305	3.33E-02
ATAD3B	41.758	20.922	1.996	6.13E-02	0.544	0.289	1.879	7.65E-02	-1.127	0.489	-2.305	3.33E-02
LINC01126	105.685	40.972	2.579	1.89E-02	-0.003	0.567	-0.006	9.95E-01	-2.206	0.957	-2.305	3.33E-02
SLC35G5	14.326	5.744	2.494	2.26E-02	0.029	0.079	0.363	7.21E-01	-0.309	0.134	-2.304	3.33E-02
OTOGL	-119.448	51.501	-2.319	3.23E-02	-0.189	0.712	-0.266	7.94E-01	2.773	1.203	2.304	3.33E-02
LOC10536983	-74.878	39.708	-1.886	7.56E-02	-2.420	0.549	-4.407	3.40E-04	2.138	0.928	2.304	3.34E-02
PNMA6A	-4.120	2.100	-1.962	6.54E-02	-0.113	0.029	-3.892	1.07E-03	0.113	0.049	2.304	3.34E-02
LOC10272370	-43.773	21.839	-2.004	6.03E-02	-1.175	0.302	-3.891	1.07E-03	1.175	0.510	2.304	3.34E-02
MIR670	-298.905	152.151	-1.965	6.51E-02	-8.504	2.105	-4.041	7.67E-04	8.189	3.555	2.304	3.34E-02
TPH2	86.886	35.916	2.419	2.64E-02	0.287	0.497	0.578	5.70E-01	-1.933	0.839	-2.303	3.34E-02
SNORD45B	-559.677	259.754	-2.155	4.50E-02	-7.734	3.593	-2.153	4.52E-02	13.978	6.069	2.303	3.34E-02
TMSB4X	-75.466	38.426	-1.964	6.52E-02	-1.909	0.532	-3.591	2.09E-03	2.067	0.898	2.302	3.35E-02
TRI.AAT2.1	-160.390	83.509	-1.921	7.08E-02	-4.905	1.155	-4.247	4.85E-04	4.488	1.951	2.300	3.36E-02
LOC10537484	-87.519	43.929	-1.992	6.17E-02	-1.937	0.608	-3.188	5.10E-03	2.361	1.026	2.300	3.36E-02
LOC10537400	214.496	77.320	2.774	1.25E-02	-1.255	1.069	-1.174	2.56E-01	-4.153	1.807	-2.299	3.37E-02
LINC00384	-76.557	40.340	-1.898	7.39E-02	-2.085	0.558	-3.737	1.51E-03	2.166	0.943	2.298	3.37E-02
SNORA9	193.564	73.938	2.618	1.74E-02	-0.128	1.023	-0.125	9.02E-01	-3.970	1.728	-2.298	3.37E-02
PRAMEF13	84.112	36.572	2.300	3.36E-02	0.542	0.506	1.072	2.98E-01	-1.964	0.855	-2.298	3.38E-02

LINC00894	-67.327	29.168	-2.308	3.31E-02	-0.272	0.403	-0.675	5.08E-01	1.566	0.681	2.297	3.38E-02
OARD1	76.970	35.780	2.151	4.53E-02	0.760	0.495	1.536	1.42E-01	-1.920	0.836	-2.297	3.38E-02
LOC1053703	-463.620	188.238	-2.463	2.41E-02	0.043	2.604	0.017	9.87E-01	10.100	4.398	2.296	3.39E-02
ZHX2	-83.884	35.478	-2.364	2.95E-02	-0.307	0.491	-0.625	5.40E-01	1.903	0.829	2.296	3.39E-02
TRAPPC6B	-144.328	61.439	-2.349	3.04E-02	-0.652	0.850	-0.768	4.53E-01	3.295	1.436	2.295	3.39E-02
TRV.CAC1.3	415.465	161.935	2.566	1.95E-02	-0.177	2.240	-0.079	9.38E-01	-8.683	3.784	-2.295	3.40E-02
UTP6	-105.582	46.909	-2.251	3.71E-02	-0.833	0.649	-1.284	2.16E-01	2.514	1.096	2.293	3.41E-02
PACS2	54.996	24.372	2.256	3.67E-02	0.294	0.337	0.872	3.95E-01	-1.306	0.569	-2.293	3.41E-02
GSC	71.796	29.257	2.454	2.45E-02	0.093	0.405	0.230	8.20E-01	-1.566	0.684	-2.291	3.43E-02
SBK1	54.669	21.737	2.515	2.16E-02	-0.012	0.301	-0.039	9.70E-01	-1.163	0.508	-2.290	3.43E-02
PRM2	125.452	50.034	2.507	2.20E-02	0.000	0.692	0.000	1.00E+00	-2.677	1.169	-2.290	3.43E-02
LOC1001322	-7.026	3.505	-2.004	6.03E-02	-0.188	0.048	-3.868	1.13E-03	0.188	0.082	2.290	3.43E-02
MIR567	-77.964	38.896	-2.004	6.03E-02	-2.081	0.538	-3.868	1.13E-03	2.081	0.909	2.290	3.43E-02
SNORA74C.1	-159.097	82.219	-1.935	6.89E-02	-4.794	1.137	-4.215	5.20E-04	4.398	1.921	2.289	3.44E-02
LOC1053772	-215.553	90.121	-2.392	2.79E-02	-0.572	1.247	-0.459	6.52E-01	4.817	2.106	2.288	3.45E-02
LINC02421	-203.282	88.552	-2.296	3.39E-02	-0.589	1.225	-0.481	6.37E-01	4.733	2.069	2.288	3.45E-02
LOC1053758	423.367	162.304	2.608	1.78E-02	-0.027	2.245	-0.012	9.91E-01	-8.672	3.792	-2.287	3.45E-02
LOC1053715	-28.306	15.066	-1.879	7.66E-02	-0.906	0.208	-4.346	3.90E-04	0.805	0.352	2.286	3.46E-02
TFAP2A.AS2	95.486	41.758	2.287	3.45E-02	0.389	0.578	0.673	5.10E-01	-2.230	0.976	-2.286	3.46E-02
PSG8	-318.412	133.891	-2.378	2.87E-02	-0.304	1.852	-0.164	8.71E-01	7.150	3.128	2.285	3.46E-02
SNORA50D	-56.623	25.669	-2.206	4.06E-02	-0.605	0.355	-1.705	1.05E-01	1.371	0.600	2.285	3.46E-02
LOC1053716	-270.683	117.267	-2.308	3.31E-02	-0.520	1.622	-0.320	7.52E-01	6.256	2.740	2.283	3.48E-02
NDUFAF6	39.178	14.351	2.730	1.37E-02	-0.282	0.198	-1.421	1.72E-01	-0.765	0.335	-2.283	3.48E-02
OSCAR	35.910	13.856	2.592	1.84E-02	-0.043	0.192	-0.224	8.26E-01	-0.739	0.324	-2.282	3.49E-02
ESCO1	82.828	34.829	2.378	2.87E-02	0.220	0.482	0.458	6.53E-01	-1.855	0.814	-2.280	3.50E-02
MIR31	688.390	280.387	2.455	2.45E-02	1.268	3.878	0.327	7.47E-01	-14.933	6.551	-2.279	3.51E-02
ATG4C	82.107	39.884	2.059	5.43E-02	0.829	0.552	1.504	1.50E-01	-2.124	0.932	-2.279	3.51E-02
GIMAP5	-3.050	1.549	-1.968	6.46E-02	-0.082	0.021	-3.849	1.18E-03	0.082	0.036	2.279	3.51E-02
DAAM2.AS1	88.492	38.925	2.273	3.55E-02	0.652	0.538	1.211	2.42E-01	-2.072	0.909	-2.278	3.51E-02
PTCHD4	33.305	15.835	2.103	4.98E-02	0.265	0.219	1.211	2.41E-01	-0.843	0.370	-2.278	3.52E-02
LOC1019291	-256.604	104.980	-2.444	2.50E-02	0.199	1.452	0.137	8.93E-01	5.587	2.453	2.278	3.52E-02
LOC155060	-140.211	63.366	-2.213	4.01E-02	-0.736	0.876	-0.840	4.12E-01	3.372	1.481	2.278	3.52E-02
LOC400682	51.830	20.636	2.512	2.18E-02	-0.080	0.285	-0.281	7.82E-01	-1.098	0.482	-2.278	3.52E-02
LOC1053784	-33.003	15.584	-2.118	4.84E-02	-0.690	0.216	-3.199	4.97E-03	0.829	0.364	2.277	3.52E-02
MIR4691	-250.808	129.460	-1.937	6.86E-02	-6.886	1.791	-3.845	1.19E-03	6.886	3.025	2.276	3.53E-02
RAB34	139.615	55.538	2.514	2.17E-02	0.252	0.768	0.328	7.46E-01	-2.954	1.298	-2.276	3.53E-02
LOC1019279	88.421	45.950	1.924	7.03E-02	1.468	0.636	2.309	3.30E-02	-2.444	1.074	-2.276	3.53E-02
LOC1053783	259.054	112.888	2.295	3.40E-02	1.778	1.561	1.139	2.70E-01	-6.000	2.638	-2.275	3.54E-02
MNAT1	-172.609	75.444	-2.288	3.45E-02	-0.728	1.044	-0.698	4.94E-01	4.010	1.763	2.275	3.54E-02
LOC1079841	185.088	77.330	2.393	2.78E-02	0.104	1.070	0.098	9.23E-01	-4.109	1.807	-2.274	3.54E-02
TRL.AAG4.1	634.170	256.895	2.469	2.38E-02	0.688	3.553	0.194	8.49E-01	-13.650	6.002	-2.274	3.54E-02
NPSR1	-162.355	72.139	-2.251	3.72E-02	-0.975	0.998	-0.977	3.42E-01	3.832	1.686	2.273	3.55E-02
GPR68	53.279	21.891	2.434	2.56E-02	0.041	0.303	0.135	8.94E-01	-1.163	0.511	-2.273	3.55E-02
LOC1079860	61.910	23.623	2.621	1.73E-02	-0.062	0.327	-0.189	8.52E-01	-1.255	0.552	-2.273	3.55E-02
CRHR2	-71.147	32.989	-2.157	4.48E-02	-0.903	0.456	-1.979	6.33E-02	1.752	0.771	2.273	3.55E-02
GUCA2A	65.331	24.223	2.697	1.47E-02	-0.243	0.335	-0.724	4.78E-01	-1.286	0.566	-2.272	3.56E-02
KISS1	-65.151	30.478	-2.138	4.65E-02	-0.899	0.422	-2.133	4.70E-02	1.618	0.712	2.272	3.56E-02
ADAMTS16	-92.834	43.012	-2.158	4.47E-02	-0.914	0.595	-1.537	1.42E-01	2.282	1.005	2.271	3.57E-02
LOC1053757	63.322	31.234	2.027	5.77E-02	0.819	0.432	1.896	7.42E-02	-1.657	0.730	-2.270	3.57E-02
NARS1	-164.103	69.927	-2.347	3.06E-02	-0.312	0.967	-0.323	7.51E-01	3.709	1.634	2.270	3.57E-02
LOC1053698	79.596	33.166	2.400	2.74E-02	0.156	0.459	0.339	7.39E-01	-1.759	0.775	-2.270	3.57E-02
LOC1053699	-380.435	160.711	-2.367	2.93E-02	-0.173	2.223	-0.078	9.39E-01	8.519	3.755	2.269	3.58E-02
AGAP1.IT1	64.438	23.610	2.729	1.38E-02	-0.339	0.327	-1.037	3.14E-01	-1.251	0.552	-2.268	3.59E-02

LOC10798568	-75.351	32.606	-2.311	3.29E-02	-0.119	0.451	-0.264	7.95E-01	1.727	0.762	2.268	3.59E-02
OR2A25	-27.053	12.220	-2.214	4.00E-02	-0.202	0.169	-1.197	2.47E-01	0.647	0.286	2.267	3.60E-02
RPS19	67.245	31.718	2.120	4.82E-02	0.705	0.439	1.606	1.26E-01	-1.679	0.741	-2.266	3.60E-02
FAM199X	-100.515	43.011	-2.337	3.12E-02	-0.203	0.595	-0.341	7.37E-01	2.276	1.005	2.265	3.61E-02
LOC10537808	82.889	33.097	2.504	2.21E-02	-0.163	0.458	-0.357	7.25E-01	-1.751	0.773	-2.265	3.61E-02
HMGA1P7	-130.418	57.249	-2.278	3.51E-02	-0.411	0.792	-0.519	6.10E-01	3.029	1.338	2.265	3.61E-02
MIR3923	-94.988	48.095	-1.975	6.38E-02	-2.543	0.665	-3.823	1.25E-03	2.543	1.124	2.263	3.62E-02
TMEM210	-717.153	303.659	-2.362	2.97E-02	-0.865	4.200	-0.206	8.39E-01	16.056	7.095	2.263	3.62E-02
LOC10537305	173.088	71.886	2.408	2.70E-02	0.103	0.994	0.104	9.18E-01	-3.801	1.680	-2.263	3.62E-02
LILRB4_1	4.115	1.678	2.453	2.46E-02	0.006	0.023	0.277	7.85E-01	-0.089	0.039	-2.262	3.63E-02
LOC10192809	56.336	24.464	2.303	3.34E-02	0.177	0.338	0.523	6.07E-01	-1.293	0.572	-2.261	3.63E-02
MIR9900	-279.831	132.347	-2.114	4.87E-02	-3.641	1.831	-1.989	6.21E-02	6.989	3.092	2.260	3.64E-02
LOC10798496	185.066	81.160	2.280	3.50E-02	0.704	1.123	0.627	5.39E-01	-4.285	1.896	-2.260	3.65E-02
BLACAT1	61.819	23.150	2.670	1.56E-02	-0.261	0.320	-0.814	4.26E-01	-1.222	0.541	-2.260	3.65E-02
LOC10192833	96.930	38.136	2.542	2.05E-02	-0.425	0.527	-0.805	4.31E-01	-2.014	0.891	-2.260	3.65E-02
GBF1	83.756	34.435	2.432	2.57E-02	0.107	0.476	0.224	8.25E-01	-1.818	0.805	-2.260	3.65E-02
UQCRC2	8.266	3.322	2.488	2.29E-02	0.010	0.046	0.221	8.28E-01	-0.175	0.078	-2.259	3.65E-02
LOC10537128	142.000	54.896	2.587	1.86E-02	-0.487	0.759	-0.641	5.30E-01	-2.897	1.283	-2.258	3.66E-02
FTHL18	-46.897	23.732	-1.976	6.37E-02	-1.252	0.328	-3.815	1.27E-03	1.252	0.554	2.258	3.66E-02
SNORD114.11	459.779	181.109	2.539	2.06E-02	-0.697	2.505	-0.278	7.84E-01	-9.554	4.232	-2.258	3.66E-02
ZFP90	69.557	32.705	2.127	4.75E-02	0.539	0.452	1.192	2.49E-01	-1.725	0.764	-2.257	3.66E-02
SPRR1A	117.379	49.061	2.393	2.78E-02	0.156	0.679	0.229	8.21E-01	-2.587	1.146	-2.257	3.67E-02
LOC10272490	-113.457	49.381	-2.298	3.38E-02	-0.530	0.683	-0.777	4.47E-01	2.604	1.154	2.257	3.67E-02
MIR629	-361.357	156.916	-2.303	3.34E-02	-1.279	2.170	-0.589	5.63E-01	8.273	3.666	2.257	3.67E-02
NPHP3.ACAD	-3.588	1.767	-2.030	5.74E-02	-0.058	0.024	-2.359	2.98E-02	0.093	0.041	2.254	3.69E-02
CYP51A1.AS1	-229.175	97.466	-2.351	3.03E-02	-0.332	1.348	-0.246	8.08E-01	5.133	2.277	2.254	3.69E-02
EML2	59.185	24.068	2.459	2.43E-02	-0.022	0.333	-0.066	9.48E-01	-1.267	0.562	-2.254	3.69E-02
LOC10272430	-335.719	150.108	-2.237	3.82E-02	-1.501	2.076	-0.723	4.79E-01	7.904	3.507	2.254	3.69E-02
LOC10798645	74.182	32.215	2.303	3.34E-02	0.341	0.446	0.765	4.54E-01	-1.696	0.753	-2.254	3.69E-02
DELEC1	164.730	73.700	2.235	3.83E-02	1.151	1.019	1.129	2.74E-01	-3.881	1.722	-2.254	3.69E-02
LINC00514	102.285	41.488	2.465	2.40E-02	0.188	0.574	0.328	7.47E-01	-2.184	0.969	-2.253	3.69E-02
MBTD1	74.837	28.914	2.588	1.86E-02	-0.068	0.400	-0.170	8.67E-01	-1.522	0.676	-2.253	3.70E-02
LOC10537232	20.068	7.911	2.537	2.07E-02	-0.018	0.109	-0.166	8.70E-01	-0.416	0.185	-2.253	3.70E-02
LINC02586	148.770	83.611	1.779	9.21E-02	3.398	1.156	2.938	8.79E-03	-4.400	1.954	-2.253	3.70E-02
LOC10798502	-4.521	2.272	-1.990	6.20E-02	-0.120	0.031	-3.805	1.30E-03	0.120	0.053	2.252	3.70E-02
SLC20A1	64.128	30.512	2.102	4.99E-02	0.745	0.422	1.766	9.43E-02	-1.606	0.713	-2.252	3.70E-02
TRP.AGG2.2	638.308	229.159	2.785	1.22E-02	-4.416	3.170	-1.393	1.80E-01	-12.058	5.354	-2.252	3.70E-02
LOC10536970	-173.353	76.849	-2.256	3.68E-02	-0.562	1.063	-0.528	6.04E-01	4.043	1.796	2.251	3.71E-02
LOC10798420	-171.748	77.492	-2.216	3.98E-02	-1.314	1.072	-1.226	2.36E-01	4.075	1.811	2.251	3.71E-02
CCDC73	-212.358	87.798	-2.419	2.64E-02	0.090	1.214	0.074	9.42E-01	4.615	2.051	2.250	3.72E-02
HARBI1	63.815	24.988	2.554	1.99E-02	-0.219	0.346	-0.632	5.35E-01	-1.313	0.584	-2.249	3.73E-02
PPL	66.644	26.785	2.488	2.29E-02	-0.065	0.370	-0.177	8.62E-01	-1.407	0.626	-2.249	3.73E-02
LOC10537228	-394.937	173.675	-2.274	3.54E-02	-1.281	2.402	-0.533	6.00E-01	9.124	4.058	2.249	3.73E-02
MIR6764	362.718	141.241	2.568	1.94E-02	-0.533	1.954	-0.273	7.88E-01	-7.420	3.300	-2.248	3.73E-02
UBE2D1	-217.094	96.757	-2.244	3.77E-02	-1.450	1.338	-1.083	2.93E-01	5.083	2.261	2.248	3.73E-02
LOC10537023	-127.799	62.933	-2.031	5.73E-02	-2.694	0.870	-3.095	6.25E-03	3.305	1.470	2.248	3.74E-02
SNAR.C1	216.803	98.626	2.198	4.13E-02	1.601	1.364	1.173	2.56E-01	-5.180	2.304	-2.248	3.74E-02
GABRE	39.054	17.794	2.195	4.15E-02	0.311	0.246	1.265	2.22E-01	-0.935	0.416	-2.248	3.74E-02
LINC00905	-187.645	81.272	-2.309	3.30E-02	-0.351	1.124	-0.312	7.58E-01	4.264	1.899	2.245	3.75E-02
MIR3938	356.705	129.152	2.762	1.28E-02	-2.311	1.786	-1.294	2.12E-01	-6.774	3.018	-2.245	3.76E-02
LOC11226817	15.181	6.219	2.441	2.52E-02	0.020	0.086	0.238	8.14E-01	-0.326	0.145	-2.245	3.76E-02
SOBP	37.109	17.300	2.145	4.58E-02	0.151	0.239	0.631	5.36E-01	-0.907	0.404	-2.245	3.76E-02
OR1N1	90.963	35.090	2.592	1.84E-02	-0.282	0.485	-0.581	5.68E-01	-1.840	0.820	-2.244	3.76E-02

COX17	137.507	59.359	2.317	3.25E-02	0.732	0.821	0.891	3.85E-01	-3.112	1.387	-2.244	3.77E-02
LOC1053780	45.581	19.026	2.396	2.77E-02	-0.076	0.263	-0.289	7.76E-01	-0.997	0.445	-2.244	3.77E-02
CFLAR	32.381	15.293	2.117	4.84E-02	0.280	0.212	1.323	2.03E-01	-0.801	0.357	-2.243	3.77E-02
RAD21L1	65.158	28.544	2.283	3.48E-02	0.039	0.395	0.098	9.23E-01	-1.495	0.667	-2.242	3.78E-02
KIF5B	119.611	50.721	2.358	2.99E-02	0.100	0.702	0.142	8.89E-01	-2.657	1.185	-2.242	3.78E-02
LOC1053785	-66.176	32.294	-2.049	5.53E-02	-0.803	0.447	-1.799	8.88E-02	1.691	0.755	2.242	3.78E-02
RBL2	-132.622	57.404	-2.310	3.29E-02	-0.415	0.794	-0.522	6.08E-01	3.006	1.341	2.241	3.78E-02
LOC1079860	-318.341	140.230	-2.270	3.57E-02	-0.614	1.940	-0.317	7.55E-01	7.341	3.276	2.241	3.79E-02
LOC1053787	122.784	56.368	2.178	4.29E-02	1.044	0.780	1.339	1.97E-01	-2.951	1.317	-2.240	3.79E-02
LOC1053773	135.965	56.986	2.386	2.82E-02	0.300	0.788	0.381	7.08E-01	-2.982	1.331	-2.239	3.80E-02
PCDHB1	98.328	40.563	2.424	2.61E-02	0.033	0.561	0.059	9.54E-01	-2.121	0.948	-2.238	3.81E-02
YPEL3	146.208	66.897	2.186	4.23E-02	1.462	0.925	1.580	1.31E-01	-3.498	1.563	-2.238	3.81E-02
CETN1	-330.980	145.672	-2.272	3.56E-02	-0.682	2.015	-0.338	7.39E-01	7.617	3.404	2.238	3.81E-02
CDK14	104.827	48.202	2.175	4.32E-02	0.592	0.667	0.888	3.86E-01	-2.520	1.126	-2.237	3.82E-02
INS.IGF2	7.029	2.772	2.535	2.07E-02	-0.009	0.038	-0.239	8.14E-01	-0.145	0.065	-2.236	3.83E-02
SNORD18C	-181.683	93.730	-1.938	6.84E-02	-4.895	1.296	-3.776	1.38E-03	4.895	2.190	2.235	3.83E-02
ARRDC3.AS1	-220.605	99.095	-2.226	3.90E-02	-0.539	1.371	-0.393	6.99E-01	5.174	2.315	2.235	3.84E-02
CZIB	113.439	50.601	2.242	3.78E-02	0.609	0.700	0.869	3.96E-01	-2.642	1.182	-2.235	3.84E-02
LOC1053752	-297.843	132.682	-2.245	3.76E-02	-0.769	1.835	-0.419	6.80E-01	6.926	3.100	2.234	3.84E-02
HEMK1	34.045	15.066	2.260	3.65E-02	0.120	0.208	0.576	5.72E-01	-0.786	0.352	-2.234	3.84E-02
HLA.DQB1.AS	148.906	61.082	2.438	2.54E-02	0.098	0.845	0.115	9.09E-01	-3.188	1.427	-2.234	3.84E-02
FAM210A	55.750	23.621	2.360	2.98E-02	0.087	0.327	0.268	7.92E-01	-1.233	0.552	-2.233	3.85E-02
SPDYE7P	119.035	46.680	2.550	2.01E-02	-0.058	0.646	-0.090	9.29E-01	-2.435	1.091	-2.233	3.85E-02
LINC01886	94.023	39.271	2.394	2.78E-02	0.306	0.543	0.563	5.80E-01	-2.048	0.918	-2.232	3.85E-02
LINC00280	40.708	17.875	2.277	3.52E-02	0.210	0.247	0.851	4.06E-01	-0.932	0.418	-2.232	3.86E-02
MYO5B	44.271	18.661	2.372	2.90E-02	-0.067	0.258	-0.260	7.98E-01	-0.973	0.436	-2.232	3.86E-02
SDR16C6P	248.175	99.395	2.497	2.25E-02	-0.428	1.375	-0.312	7.59E-01	-5.183	2.322	-2.232	3.86E-02
GPR42	54.525	22.032	2.475	2.35E-02	-0.101	0.305	-0.331	7.45E-01	-1.149	0.515	-2.232	3.86E-02
STX8	-224.627	95.440	-2.354	3.02E-02	-0.462	1.320	-0.350	7.30E-01	4.972	2.230	2.230	3.88E-02
NOLC1	92.109	40.941	2.250	3.72E-02	0.226	0.566	0.400	6.94E-01	-2.133	0.957	-2.229	3.88E-02
PRR23B	-251.760	109.589	-2.297	3.38E-02	-0.007	1.516	-0.005	9.96E-01	5.708	2.561	2.229	3.88E-02
PATE1	113.670	59.043	1.925	7.02E-02	1.434	0.817	1.756	9.61E-02	-3.074	1.380	-2.229	3.88E-02
LOC1079846	-139.488	68.443	-2.038	5.65E-02	-2.463	0.947	-2.602	1.80E-02	3.559	1.599	2.226	3.91E-02
TLR6	71.619	31.453	2.277	3.52E-02	0.273	0.435	0.627	5.38E-01	-1.635	0.735	-2.225	3.91E-02
LINC02042	-217.720	98.488	-2.211	4.02E-02	-0.880	1.362	-0.646	5.26E-01	5.119	2.301	2.225	3.91E-02
FAM27E4	48.857	19.655	2.486	2.30E-02	-0.052	0.272	-0.190	8.52E-01	-1.022	0.459	-2.225	3.91E-02
NEK6	-167.641	74.158	-2.261	3.64E-02	-0.524	1.026	-0.511	6.16E-01	3.854	1.733	2.224	3.92E-02
LOC1053771	-45.996	21.262	-2.163	4.42E-02	-0.380	0.294	-1.291	2.13E-01	1.105	0.497	2.224	3.92E-02
LOC1053784	52.604	23.111	2.276	3.53E-02	0.265	0.320	0.830	4.17E-01	-1.201	0.540	-2.223	3.92E-02
NKAPL	146.504	61.341	2.388	2.81E-02	0.103	0.848	0.122	9.05E-01	-3.186	1.433	-2.223	3.93E-02
ZNF66_1	48.749	23.364	2.086	5.14E-02	0.393	0.323	1.217	2.39E-01	-1.213	0.546	-2.223	3.93E-02
LOC1019280	128.831	62.448	2.063	5.38E-02	1.608	0.864	1.861	7.91E-02	-3.243	1.459	-2.223	3.93E-02
TSPY10	-15.733	8.613	-1.827	8.44E-02	-0.516	0.119	-4.333	4.01E-04	0.447	0.201	2.222	3.93E-02
LOC1053722	53.163	23.376	2.274	3.54E-02	0.287	0.323	0.888	3.86E-01	-1.214	0.546	-2.222	3.94E-02
LOC1079859	-777.519	338.047	-2.300	3.36E-02	-1.097	4.676	-0.235	8.17E-01	17.545	7.898	2.221	3.94E-02
LOC1053720	243.784	106.985	2.279	3.51E-02	1.338	1.480	0.904	3.78E-01	-5.552	2.500	-2.221	3.94E-02
LOC1027237	-121.461	53.846	-2.256	3.68E-02	-0.486	0.745	-0.652	5.23E-01	2.794	1.258	2.221	3.94E-02
MOXD2P	37.505	15.759	2.380	2.86E-02	0.063	0.218	0.288	7.77E-01	-0.818	0.368	-2.221	3.94E-02
HCP5B	59.827	22.514	2.657	1.60E-02	-0.334	0.311	-1.072	2.98E-01	-1.168	0.526	-2.221	3.95E-02
EDARADD	-107.754	49.663	-2.170	4.37E-02	-1.117	0.687	-1.626	1.21E-01	2.576	1.160	2.220	3.95E-02
MTFP1	85.185	40.132	2.123	4.79E-02	0.854	0.555	1.539	1.41E-01	-2.081	0.938	-2.220	3.95E-02
B3GNTL1	22.976	8.994	2.554	1.99E-02	-0.072	0.124	-0.577	5.71E-01	-0.466	0.210	-2.217	3.97E-02
LOC1079856	-29.890	15.408	-1.940	6.82E-02	-0.736	0.213	-3.452	2.84E-03	0.798	0.360	2.217	3.97E-02

LOC10537544	-161.646	71.180	-2.271	3.57E-02	-0.788	0.985	-0.800	4.34E-01	3.686	1.663	2.216	3.98E-02
TRABD	-91.460	42.669	-2.143	4.60E-02	-0.813	0.590	-1.378	1.85E-01	2.209	0.997	2.216	3.98E-02
RPS27	-104.747	53.818	-1.946	6.74E-02	-2.503	0.744	-3.363	3.46E-03	2.786	1.257	2.216	3.98E-02
LOC10192928	-253.273	108.252	-2.340	3.10E-02	0.002	1.497	0.001	9.99E-01	5.604	2.529	2.216	3.98E-02
CLYBL.AS1	178.643	71.997	2.481	2.32E-02	-0.372	0.996	-0.374	7.13E-01	-3.725	1.682	-2.215	3.99E-02
ACAP3	-58.813	25.762	-2.283	3.48E-02	0.018	0.356	0.050	9.61E-01	1.332	0.602	2.213	4.00E-02
LOC10537657	95.886	44.186	2.170	4.36E-02	0.625	0.611	1.023	3.20E-01	-2.285	1.032	-2.213	4.00E-02
LOC10272438	56.139	22.860	2.456	2.45E-02	-0.037	0.316	-0.117	9.08E-01	-1.182	0.534	-2.213	4.01E-02
SUMO1	-106.717	46.596	-2.290	3.43E-02	-0.384	0.645	-0.596	5.58E-01	2.409	1.089	2.213	4.01E-02
LOC10537860	-55.199	29.568	-1.867	7.83E-02	-1.755	0.409	-4.290	4.40E-04	1.528	0.691	2.212	4.01E-02
LINC01048	-753.700	330.635	-2.280	3.50E-02	-1.284	4.573	-0.281	7.82E-01	17.088	7.725	2.212	4.01E-02
LINC02428	326.657	161.229	2.026	5.78E-02	3.693	2.230	1.656	1.15E-01	-8.331	3.767	-2.212	4.02E-02
CICP24	-15.303	7.815	-1.958	6.59E-02	-0.412	0.108	-3.809	1.29E-03	0.404	0.183	2.211	4.02E-02
ZNHIT6	-108.045	47.665	-2.267	3.60E-02	-0.101	0.659	-0.153	8.80E-01	2.462	1.114	2.210	4.03E-02
STRIP1	59.760	26.440	2.260	3.64E-02	0.068	0.366	0.186	8.55E-01	-1.364	0.618	-2.208	4.04E-02
LOC10536988	-119.902	57.864	-2.072	5.29E-02	-1.308	0.800	-1.635	1.19E-01	2.985	1.352	2.208	4.05E-02
LOC649352	57.997	23.733	2.444	2.51E-02	0.074	0.328	0.227	8.23E-01	-1.224	0.555	-2.208	4.05E-02
ADCYAP1	-352.190	152.932	-2.303	3.34E-02	0.340	2.115	0.161	8.74E-01	7.888	3.573	2.207	4.05E-02
LOC10537620	-89.182	42.795	-2.084	5.17E-02	-0.671	0.592	-1.134	2.72E-01	2.207	1.000	2.207	4.05E-02
LOC10536978	-427.330	192.573	-2.219	3.96E-02	-1.808	2.664	-0.679	5.06E-01	9.931	4.499	2.207	4.05E-02
METTL7A	116.599	52.488	2.221	3.94E-02	0.996	0.726	1.371	1.87E-01	-2.706	1.226	-2.207	4.06E-02
ZNF192P1	-370.978	167.379	-2.216	3.98E-02	-0.787	2.315	-0.340	7.38E-01	8.624	3.911	2.205	4.07E-02
LINC02608	-289.620	135.748	-2.134	4.69E-02	-1.784	1.878	-0.950	3.55E-01	6.994	3.172	2.205	4.07E-02
LOC10537766	-603.623	273.766	-2.205	4.07E-02	-1.887	3.787	-0.498	6.24E-01	14.101	6.396	2.205	4.07E-02
LOC10537162	-108.823	49.024	-2.220	3.95E-02	-0.862	0.678	-1.271	2.20E-01	2.524	1.145	2.204	4.08E-02
LOC10798620	153.483	63.087	2.433	2.56E-02	0.192	0.873	0.220	8.29E-01	-3.248	1.474	-2.204	4.08E-02
PRAMEF14	103.026	43.710	2.357	2.99E-02	0.154	0.605	0.255	8.02E-01	-2.250	1.021	-2.203	4.09E-02
LOC729609	103.389	45.722	2.261	3.64E-02	0.762	0.632	1.204	2.44E-01	-2.353	1.068	-2.203	4.09E-02
MIR4504	1001.165	387.159	2.586	1.86E-02	-2.394	5.355	-0.447	6.60E-01	-19.924	9.046	-2.203	4.09E-02
LOC10537330	383.795	149.297	2.571	1.92E-02	-0.197	2.065	-0.095	9.25E-01	-7.683	3.488	-2.202	4.09E-02
CSMD2	40.692	18.363	2.216	3.98E-02	0.195	0.254	0.768	4.53E-01	-0.945	0.429	-2.202	4.10E-02
UBE3A	-96.913	43.683	-2.219	3.96E-02	-0.344	0.604	-0.569	5.76E-01	2.247	1.021	2.202	4.10E-02
LOC10537318	-121.252	55.529	-2.184	4.25E-02	-0.938	0.768	-1.221	2.38E-01	2.856	1.297	2.201	4.10E-02
LINC01840	-53.728	28.560	-1.881	7.62E-02	-1.562	0.395	-3.953	9.31E-04	1.468	0.667	2.200	4.11E-02
KDM2B	-125.743	56.659	-2.219	3.96E-02	-0.569	0.784	-0.726	4.77E-01	2.912	1.324	2.200	4.11E-02
LOC10798421	-75.014	39.109	-1.918	7.11E-02	-1.836	0.541	-3.394	3.23E-03	2.010	0.914	2.200	4.11E-02
LOC10192897	65.983	39.826	1.657	1.15E-01	1.676	0.551	3.042	7.02E-03	-2.047	0.931	-2.200	4.11E-02
OR14A16	-126.919	57.075	-2.224	3.92E-02	-0.569	0.789	-0.720	4.81E-01	2.931	1.334	2.198	4.13E-02
HIKESHI	-132.187	59.070	-2.238	3.81E-02	-0.444	0.817	-0.544	5.93E-01	3.034	1.380	2.198	4.13E-02
LOC10537811	157.524	63.105	2.496	2.25E-02	-0.212	0.873	-0.243	8.11E-01	-3.241	1.474	-2.198	4.13E-02
TRQ.TTG1.1	153.695	61.571	2.496	2.25E-02	-0.207	0.852	-0.243	8.11E-01	-3.162	1.439	-2.198	4.13E-02
LOC10536992	-454.918	203.626	-2.234	3.84E-02	-0.757	2.817	-0.269	7.91E-01	10.457	4.758	2.198	4.13E-02
PRB1	42.296	19.092	2.215	3.99E-02	0.213	0.264	0.807	4.30E-01	-0.980	0.446	-2.197	4.13E-02
TRE.CTC1.7	622.730	253.938	2.452	2.46E-02	0.339	3.512	0.096	9.24E-01	-13.035	5.933	-2.197	4.14E-02
LOC10798515	162.200	72.905	2.225	3.91E-02	1.062	1.008	1.053	3.06E-01	-3.738	1.703	-2.195	4.16E-02
RAB4A	-164.594	70.168	-2.346	3.06E-02	-0.164	0.971	-0.169	8.68E-01	3.597	1.639	2.194	4.16E-02
LOC10798531	-712.322	305.616	-2.331	3.16E-02	-0.055	4.227	-0.013	9.90E-01	15.668	7.141	2.194	4.16E-02
KRTAP7.1	-84.455	39.992	-2.112	4.89E-02	-0.912	0.553	-1.649	1.17E-01	2.050	0.934	2.194	4.16E-02
FABP5	-552.248	250.480	-2.205	4.07E-02	-2.694	3.465	-0.778	4.47E-01	12.840	5.852	2.194	4.16E-02
LRRC74B	91.862	38.562	2.382	2.84E-02	-0.105	0.533	-0.197	8.46E-01	-1.977	0.901	-2.194	4.16E-02
LINC01849	77.071	35.602	2.165	4.41E-02	0.550	0.492	1.116	2.79E-01	-1.825	0.832	-2.194	4.16E-02
TMX2.CTNND	-3.505	1.885	-1.859	7.94E-02	-0.102	0.026	-3.895	1.06E-03	0.097	0.044	2.194	4.16E-02
MIR365B	-81.394	56.556	-1.439	1.67E-01	-5.084	0.782	-6.499	4.12E-06	2.899	1.321	2.194	4.16E-02

LOC1053780	237.131	100.311	2.364	2.95E-02	1.046	1.388	0.754	4.61E-01	-5.141	2.344	-2.194	4.16E-02
LOC1053728	-184.736	82.255	-2.246	3.75E-02	-0.026	1.138	-0.023	9.82E-01	4.215	1.922	2.193	4.17E-02
LOC1053771	183.187	76.050	2.409	2.69E-02	0.188	1.052	0.179	8.60E-01	-3.896	1.777	-2.193	4.17E-02
LOC1019289	127.063	50.852	2.499	2.24E-02	-0.449	0.703	-0.638	5.31E-01	-2.604	1.188	-2.192	4.18E-02
LOC1079848	-204.599	90.961	-2.249	3.72E-02	-0.746	1.258	-0.593	5.60E-01	4.658	2.125	2.192	4.18E-02
VASH1.AS1	-16.109	8.611	-1.871	7.77E-02	-0.441	0.119	-3.701	1.63E-03	0.441	0.201	2.191	4.18E-02
LOC1079872	-24.729	12.325	-2.006	6.01E-02	-0.358	0.170	-2.102	4.99E-02	0.631	0.288	2.190	4.20E-02
STX11	64.767	27.736	2.335	3.13E-02	-0.033	0.384	-0.087	9.32E-01	-1.419	0.648	-2.190	4.20E-02
LINC01480	-37.697	20.003	-1.885	7.57E-02	-1.023	0.277	-3.698	1.65E-03	1.023	0.467	2.189	4.20E-02
RIC1	-112.742	50.165	-2.247	3.74E-02	-0.436	0.694	-0.629	5.37E-01	2.566	1.172	2.189	4.20E-02
TMEM225	-243.815	112.029	-2.176	4.31E-02	-0.608	1.550	-0.392	6.99E-01	5.729	2.618	2.189	4.21E-02
LOC1053787	-60.731	29.167	-2.082	5.19E-02	-0.643	0.403	-1.594	1.28E-01	1.491	0.681	2.189	4.21E-02
MIR1207	261.694	108.086	2.421	2.63E-02	-0.052	1.495	-0.035	9.73E-01	-5.524	2.525	-2.187	4.21E-02
TMEM39B	77.820	38.942	1.998	6.10E-02	0.681	0.539	1.264	2.22E-01	-1.990	0.910	-2.187	4.21E-02
RHOV	47.978	19.352	2.479	2.33E-02	-0.109	0.268	-0.406	6.89E-01	-0.989	0.452	-2.187	4.22E-02
NBR1	109.531	50.821	2.155	4.49E-02	0.760	0.703	1.081	2.94E-01	-2.596	1.187	-2.186	4.23E-02
OSMR.AS1	226.058	106.173	2.129	4.73E-02	1.776	1.469	1.210	2.42E-01	-5.423	2.481	-2.186	4.23E-02
ERLEC1	85.543	37.761	2.265	3.61E-02	0.336	0.522	0.644	5.28E-01	-1.928	0.882	-2.186	4.23E-02
LOC285762	-161.952	70.703	-2.291	3.43E-02	-0.564	0.978	-0.576	5.71E-01	3.611	1.652	2.186	4.23E-02
CYTH2	45.690	24.013	1.903	7.32E-02	0.679	0.332	2.044	5.59E-02	-1.226	0.561	-2.185	4.23E-02
THEG5	-144.562	63.393	-2.280	3.50E-02	-0.309	0.877	-0.352	7.29E-01	3.236	1.481	2.185	4.24E-02
IGKV3D.25	157.296	80.081	1.964	6.51E-02	1.851	1.108	1.671	1.12E-01	-4.088	1.871	-2.185	4.24E-02
LOC1053747	-74.506	36.447	-2.044	5.58E-02	-0.584	0.504	-1.159	2.61E-01	1.860	0.852	2.184	4.24E-02
FRG2C	128.839	56.293	2.289	3.44E-02	0.350	0.779	0.450	6.58E-01	-2.872	1.315	-2.184	4.24E-02
DEFA1B_1	-77.436	36.009	-2.150	4.54E-02	-0.533	0.498	-1.070	2.99E-01	1.837	0.841	2.184	4.24E-02
KRTAP10.4	-156.370	66.939	-2.336	3.13E-02	-0.250	0.926	-0.270	7.90E-01	3.413	1.564	2.182	4.26E-02
COL10A1	44.757	18.468	2.423	2.61E-02	-0.270	0.255	-1.056	3.05E-01	-0.941	0.432	-2.182	4.26E-02
LOC1019284	-105.442	47.919	-2.200	4.11E-02	-0.508	0.663	-0.766	4.54E-01	2.441	1.120	2.180	4.27E-02
LINC01208	252.220	99.559	2.533	2.08E-02	-0.334	1.377	-0.243	8.11E-01	-5.071	2.326	-2.180	4.28E-02
KIRREL1	-95.248	42.355	-2.249	3.73E-02	-0.392	0.586	-0.669	5.12E-01	2.157	0.990	2.179	4.28E-02
LINC01202	-387.838	178.158	-2.177	4.30E-02	-1.344	2.464	-0.545	5.92E-01	9.069	4.163	2.179	4.29E-02
NAV2.AS5	102.563	38.620	2.656	1.61E-02	-0.623	0.534	-1.167	2.59E-01	-1.966	0.902	-2.179	4.29E-02
LOC1053725	133.070	58.040	2.293	3.41E-02	0.588	0.803	0.732	4.73E-01	-2.954	1.356	-2.178	4.29E-02
LOC1053770	-44.555	25.985	-1.715	1.04E-01	-1.495	0.359	-4.160	5.88E-04	1.322	0.607	2.177	4.30E-02
LOC1053735	279.260	128.251	2.177	4.30E-02	1.788	1.774	1.008	3.27E-01	-6.524	2.997	-2.177	4.30E-02
LINC01893	109.767	47.385	2.316	3.25E-02	0.297	0.655	0.453	6.56E-01	-2.410	1.107	-2.177	4.30E-02
MIR4474	-256.848	120.439	-2.133	4.70E-02	-2.240	1.666	-1.344	1.96E-01	6.125	2.814	2.177	4.31E-02
MS4A3_1	-19.118	8.991	-2.126	4.76E-02	-0.146	0.124	-1.177	2.54E-01	0.457	0.210	2.176	4.31E-02
OBSCN.AS1	-69.117	32.326	-2.138	4.65E-02	-0.694	0.447	-1.553	1.38E-01	1.643	0.755	2.175	4.32E-02
SEZ6L	76.686	33.924	2.261	3.64E-02	0.176	0.469	0.374	7.13E-01	-1.724	0.793	-2.175	4.32E-02
LOC1053789	84.775	41.710	2.032	5.71E-02	0.542	0.577	0.939	3.60E-01	-2.120	0.975	-2.175	4.32E-02
LOC1019272	155.517	66.583	2.336	3.13E-02	0.284	0.921	0.308	7.62E-01	-3.384	1.556	-2.175	4.32E-02
MIR381	576.001	232.881	2.473	2.36E-02	-0.433	3.221	-0.135	8.94E-01	-11.830	5.441	-2.174	4.33E-02
LOC1053794	61.614	24.839	2.481	2.32E-02	-0.133	0.344	-0.386	7.04E-01	-1.262	0.580	-2.174	4.33E-02
MUCL1	265.874	114.592	2.320	3.23E-02	0.575	1.585	0.363	7.21E-01	-5.818	2.677	-2.173	4.34E-02
LOC1053753	157.397	62.626	2.513	2.17E-02	-0.328	0.866	-0.378	7.10E-01	-3.180	1.463	-2.173	4.34E-02
NANOS1	-35.097	17.015	-2.063	5.39E-02	-0.489	0.235	-2.078	5.23E-02	0.864	0.398	2.172	4.34E-02
LOC1079843	103.088	48.960	2.106	4.95E-02	0.878	0.677	1.296	2.11E-01	-2.485	1.144	-2.172	4.34E-02
CHPF	53.185	23.366	2.276	3.53E-02	0.169	0.323	0.523	6.07E-01	-1.186	0.546	-2.171	4.35E-02
LOC1053794	-259.859	119.340	-2.177	4.30E-02	-0.720	1.651	-0.436	6.68E-01	6.055	2.788	2.171	4.35E-02
TRP.TGG3.4	-762.398	332.730	-2.291	3.42E-02	0.236	4.602	0.051	9.60E-01	16.873	7.774	2.170	4.36E-02
SNORA87	-364.604	196.428	-1.856	7.99E-02	-8.207	2.717	-3.021	7.35E-03	9.958	4.589	2.170	4.37E-02
LOC1079845	101.541	45.914	2.212	4.02E-02	0.442	0.635	0.696	4.95E-01	-2.327	1.073	-2.169	4.37E-02

LOC1079842	-141.807	60.068	-2.361	2.97E-02	0.086	0.831	0.103	9.19E-01	3.045	1.403	2.169	4.37E-02
LOC1079868	-161.794	71.332	-2.268	3.59E-02	0.020	0.987	0.020	9.84E-01	3.614	1.667	2.169	4.38E-02
KRTAP20.4	-191.787	98.359	-1.950	6.69E-02	-3.890	1.360	-2.859	1.04E-02	4.983	2.298	2.168	4.38E-02
MIR512.1	-2398.940	1052.116	-2.280	3.50E-02	0.594	14.553	0.041	9.68E-01	53.289	24.582	2.168	4.38E-02
TRC.GCA16.1	502.407	197.725	2.541	2.05E-02	-1.768	2.735	-0.646	5.26E-01	-10.014	4.620	-2.168	4.38E-02
MIR330	520.951	223.274	2.333	3.14E-02	0.986	3.088	0.319	7.53E-01	-11.307	5.217	-2.168	4.38E-02
OR52E2	97.345	41.598	2.340	3.10E-02	0.124	0.575	0.216	8.32E-01	-2.106	0.972	-2.167	4.39E-02
VPS37B	39.305	16.292	2.412	2.67E-02	-0.082	0.225	-0.365	7.19E-01	-0.825	0.381	-2.167	4.39E-02
LOC1053769	83.289	34.303	2.428	2.59E-02	0.167	0.474	0.352	7.29E-01	-1.737	0.801	-2.167	4.39E-02
BID	45.378	19.871	2.284	3.48E-02	0.030	0.275	0.109	9.14E-01	-1.006	0.464	-2.167	4.39E-02
LOC1053716	-388.711	173.689	-2.238	3.81E-02	-0.359	2.402	-0.149	8.83E-01	8.791	4.058	2.166	4.40E-02
NKX3.2	82.411	34.665	2.377	2.87E-02	0.039	0.479	0.081	9.36E-01	-1.755	0.810	-2.166	4.40E-02
LOC1019290	99.318	42.119	2.358	2.99E-02	0.051	0.583	0.088	9.31E-01	-2.131	0.984	-2.165	4.40E-02
FCHO2	-83.406	39.560	-2.108	4.93E-02	-0.487	0.547	-0.891	3.85E-01	2.001	0.924	2.165	4.41E-02
MIR512.2	-2057.274	901.257	-2.283	3.48E-02	0.913	12.466	0.073	9.42E-01	45.589	21.058	2.165	4.41E-02
LINC00693	76.564	31.202	2.454	2.46E-02	-0.349	0.432	-0.808	4.30E-01	-1.578	0.729	-2.164	4.41E-02
LOC1019290	-53.801	28.033	-1.919	7.10E-02	-1.100	0.388	-2.837	1.09E-02	1.417	0.655	2.164	4.42E-02
H1.1	-42.871	23.079	-1.858	7.97E-02	-1.155	0.319	-3.619	1.96E-03	1.166	0.539	2.163	4.42E-02
LOC1053713	-590.591	264.865	-2.230	3.87E-02	-1.171	3.664	-0.320	7.53E-01	13.385	6.188	2.163	4.43E-02
LOC1079850	-191.679	85.846	-2.233	3.85E-02	-0.284	1.187	-0.239	8.14E-01	4.337	2.006	2.162	4.43E-02
LGR5	-91.648	43.593	-2.102	4.99E-02	-0.454	0.603	-0.752	4.62E-01	2.202	1.019	2.162	4.43E-02
HSF2	-247.469	112.049	-2.209	4.04E-02	-0.196	1.550	-0.126	9.01E-01	5.659	2.618	2.161	4.44E-02
LOC1053729	116.144	44.310	2.621	1.73E-02	-0.522	0.613	-0.851	4.06E-01	-2.238	1.035	-2.161	4.44E-02
TGM2	-97.025	46.188	-2.101	5.00E-02	-0.640	0.639	-1.002	3.30E-01	2.332	1.079	2.161	4.44E-02
LOC1053776	-628.500	284.339	-2.210	4.03E-02	-1.208	3.933	-0.307	7.62E-01	14.356	6.643	2.161	4.44E-02
LOC1009967	3.940	1.851	2.129	4.74E-02	0.029	0.026	1.150	2.65E-01	-0.093	0.043	-2.160	4.45E-02
GOLGA8T	5.777	2.340	2.468	2.38E-02	-0.007	0.032	-0.216	8.32E-01	-0.118	0.055	-2.159	4.46E-02
LOC1053747	223.172	94.363	2.365	2.95E-02	-0.117	1.305	-0.090	9.29E-01	-4.760	2.205	-2.159	4.46E-02
MIR3936	-713.718	332.431	-2.147	4.57E-02	-2.886	4.598	-0.628	5.38E-01	16.770	7.767	2.159	4.46E-02
LOC1053768	-483.169	214.531	-2.252	3.70E-02	-0.604	2.967	-0.203	8.41E-01	10.817	5.012	2.158	4.47E-02
OR2J1	-1.964	1.067	-1.841	8.22E-02	-0.045	0.015	-3.070	6.60E-03	0.054	0.025	2.158	4.47E-02
SNTG2.AS1	-57.584	32.022	-1.798	8.89E-02	-1.778	0.443	-4.015	8.12E-04	1.614	0.748	2.157	4.47E-02
SHISA7	49.179	19.414	2.533	2.08E-02	-0.168	0.269	-0.625	5.40E-01	-0.979	0.454	-2.157	4.47E-02
TENM2	-85.773	40.372	-2.125	4.77E-02	-0.275	0.558	-0.492	6.29E-01	2.035	0.943	2.157	4.48E-02
MIR3177	544.136	222.542	2.445	2.50E-02	-0.993	3.078	-0.323	7.51E-01	-11.216	5.200	-2.157	4.48E-02
LOC1053727	-78.248	39.691	-1.971	6.42E-02	-1.066	0.549	-1.942	6.79E-02	2.000	0.927	2.157	4.48E-02
ADIG	-61.094	32.127	-1.902	7.33E-02	-1.552	0.444	-3.494	2.59E-03	1.619	0.751	2.157	4.48E-02
LOC644090	-185.356	83.489	-2.220	3.95E-02	-0.195	1.155	-0.169	8.68E-01	4.207	1.951	2.157	4.48E-02
LOC1053741	125.050	57.176	2.187	4.22E-02	0.592	0.791	0.749	4.64E-01	-2.881	1.336	-2.156	4.48E-02
LOC1027240	-148.832	68.165	-2.183	4.25E-02	-0.218	0.943	-0.232	8.19E-01	3.434	1.593	2.156	4.49E-02
SNORA101A	-143.604	78.960	-1.819	8.56E-02	-4.231	1.092	-3.874	1.11E-03	3.976	1.845	2.155	4.49E-02
WIF1	113.344	53.638	2.113	4.88E-02	0.809	0.742	1.091	2.90E-01	-2.701	1.253	-2.155	4.49E-02
LOC1053699	-163.126	85.698	-1.903	7.31E-02	-3.175	1.185	-2.679	1.53E-02	4.314	2.002	2.155	4.50E-02
BAG5	66.632	30.862	2.159	4.46E-02	0.348	0.427	0.815	4.26E-01	-1.554	0.721	-2.154	4.50E-02
LOC1079851	42.251	16.960	2.491	2.27E-02	-0.104	0.235	-0.444	6.62E-01	-0.854	0.396	-2.154	4.50E-02
LOC1079864	85.225	33.705	2.529	2.10E-02	-0.124	0.466	-0.265	7.94E-01	-1.696	0.788	-2.154	4.50E-02
LOC1122681	167.377	73.981	2.262	3.63E-02	0.388	1.023	0.379	7.09E-01	-3.723	1.729	-2.154	4.50E-02
FAM20B	58.336	32.993	1.768	9.40E-02	1.014	0.456	2.222	3.94E-02	-1.659	0.771	-2.153	4.52E-02
LOC1053706	-126.677	62.367	-2.031	5.73E-02	-1.824	0.863	-2.115	4.86E-02	3.136	1.457	2.152	4.52E-02
LOC1053722	67.928	26.329	2.580	1.89E-02	-0.361	0.364	-0.990	3.35E-01	-1.324	0.615	-2.152	4.52E-02
ZNF148	-92.504	41.858	-2.210	4.03E-02	-0.182	0.579	-0.315	7.56E-01	2.104	0.978	2.152	4.52E-02
C1QTNF3.AM	-1.721	0.935	-1.841	8.21E-02	-0.047	0.013	-3.635	1.90E-03	0.047	0.022	2.152	4.52E-02
TRV.CAC7.1	-85.139	46.243	-1.841	8.21E-02	-2.325	0.640	-3.635	1.90E-03	2.325	1.080	2.152	4.52E-02

SEMA6A.AS1	155.119	65.958	2.352	3.03E-02	0.195	0.912	0.214	8.33E-01	-3.315	1.541	-2.151	4.53E-02
ZC3H4	-94.051	43.005	-2.187	4.22E-02	-0.213	0.595	-0.358	7.24E-01	2.162	1.005	2.151	4.53E-02
KRT34	-2.804	1.702	-1.648	1.17E-01	-0.129	0.024	-5.489	3.27E-05	0.086	0.040	2.151	4.53E-02
LOC1053789	211.645	93.171	2.272	3.56E-02	0.389	1.289	0.302	7.66E-01	-4.681	2.177	-2.150	4.54E-02
LOC1122684	61.254	25.151	2.435	2.55E-02	-0.005	0.348	-0.015	9.88E-01	-1.264	0.588	-2.150	4.54E-02
OR2AG1	59.746	27.916	2.140	4.63E-02	0.375	0.386	0.971	3.44E-01	-1.402	0.652	-2.149	4.55E-02
LINC00863	50.755	21.396	2.372	2.90E-02	-0.113	0.296	-0.382	7.07E-01	-1.074	0.500	-2.149	4.55E-02
HIC2	28.542	11.491	2.484	2.31E-02	-0.194	0.159	-1.221	2.38E-01	-0.577	0.268	-2.149	4.55E-02
SLITRK4	-76.280	34.144	-2.234	3.84E-02	-0.064	0.472	-0.136	8.94E-01	1.714	0.798	2.149	4.55E-02
LOC1053742	89.758	38.331	2.342	3.09E-02	0.043	0.530	0.080	9.37E-01	-1.924	0.896	-2.148	4.56E-02
ANKRD34B	-103.614	47.713	-2.172	4.35E-02	-0.387	0.660	-0.587	5.65E-01	2.395	1.115	2.148	4.56E-02
LINC02459	189.412	81.883	2.313	3.27E-02	0.031	1.133	0.027	9.79E-01	-4.109	1.913	-2.148	4.56E-02
PLEKHG6	83.940	36.217	2.318	3.24E-02	0.271	0.501	0.542	5.95E-01	-1.816	0.846	-2.147	4.57E-02
LOC1019293	94.272	51.932	1.815	8.62E-02	1.751	0.718	2.437	2.54E-02	-2.604	1.213	-2.146	4.58E-02
PSD4	53.234	21.269	2.503	2.22E-02	-0.060	0.294	-0.202	8.42E-01	-1.066	0.497	-2.145	4.58E-02
POMT1	-82.658	38.848	-2.128	4.74E-02	-0.495	0.537	-0.922	3.69E-01	1.947	0.908	2.145	4.58E-02
RAB6C	99.255	44.422	2.234	3.84E-02	0.121	0.614	0.196	8.47E-01	-2.225	1.038	-2.144	4.59E-02
MTUS2.AS1	120.779	65.859	1.834	8.33E-02	1.845	0.911	2.025	5.79E-02	-3.299	1.539	-2.144	4.59E-02
SPAG17	84.257	37.654	2.238	3.81E-02	0.217	0.521	0.417	6.82E-01	-1.886	0.880	-2.144	4.60E-02
LOC1079857	-262.385	122.562	-2.141	4.62E-02	-1.262	1.695	-0.745	4.66E-01	6.135	2.864	2.142	4.61E-02
LOC1079858	-63.994	38.306	-1.671	1.12E-01	-2.321	0.530	-4.380	3.61E-04	1.917	0.895	2.142	4.61E-02
PPM1H	82.076	41.391	1.983	6.28E-02	0.798	0.573	1.393	1.81E-01	-2.071	0.967	-2.141	4.62E-02
SNORA24B	-1970.862	887.575	-2.221	3.95E-02	0.000	12.277	0.000	1.00E+00	44.406	20.738	2.141	4.62E-02
KRTAP5.8	78.764	30.841	2.554	1.99E-02	-0.374	0.427	-0.877	3.92E-01	-1.543	0.721	-2.141	4.62E-02
LOC1053745	183.354	70.109	2.615	1.75E-02	-0.889	0.970	-0.917	3.71E-01	-3.507	1.638	-2.141	4.62E-02
DLX4	73.319	31.137	2.355	3.01E-02	0.195	0.431	0.453	6.56E-01	-1.557	0.727	-2.141	4.62E-02
GALNT2	46.222	20.070	2.303	3.34E-02	-0.110	0.278	-0.397	6.96E-01	-1.004	0.469	-2.141	4.62E-02
GPATCH1	107.626	50.108	2.148	4.56E-02	0.494	0.693	0.713	4.85E-01	-2.506	1.171	-2.140	4.63E-02
LOC1053762	429.109	214.206	2.003	6.04E-02	4.554	2.963	1.537	1.42E-01	-10.710	5.005	-2.140	4.63E-02
TEX46	-80.762	38.488	-2.098	5.03E-02	-0.937	0.532	-1.760	9.54E-02	1.923	0.899	2.139	4.64E-02
LINC01832	-144.557	71.788	-2.014	5.92E-02	-1.859	0.993	-1.872	7.75E-02	3.586	1.677	2.138	4.65E-02
ZMIZ1.AS1	158.628	74.635	2.125	4.77E-02	0.890	1.032	0.862	4.00E-01	-3.728	1.744	-2.138	4.65E-02
AGAP13P	142.514	68.096	2.093	5.08E-02	1.196	0.942	1.270	2.20E-01	-3.401	1.591	-2.138	4.65E-02
LOC1001283	79.576	41.633	1.911	7.20E-02	0.958	0.576	1.664	1.13E-01	-2.079	0.973	-2.138	4.65E-02
TRBC2_1	36.373	14.737	2.468	2.38E-02	-0.055	0.204	-0.270	7.90E-01	-0.736	0.344	-2.137	4.66E-02
PNMA8B	42.701	20.873	2.046	5.57E-02	0.424	0.289	1.470	1.59E-01	-1.042	0.488	-2.137	4.66E-02
LINC01640	308.518	166.131	1.857	7.97E-02	5.218	2.298	2.271	3.57E-02	-8.293	3.882	-2.137	4.66E-02
LOC1001309	-31.056	15.298	-2.030	5.74E-02	-0.375	0.212	-1.773	9.32E-02	0.764	0.357	2.136	4.66E-02
LOC1053708	-556.737	255.886	-2.176	4.31E-02	-1.540	3.539	-0.435	6.69E-01	12.771	5.979	2.136	4.67E-02
LINC01166	36.516	15.420	2.368	2.93E-02	0.011	0.213	0.052	9.59E-01	-0.769	0.360	-2.135	4.68E-02
MRPL45P2	-217.682	103.365	-2.106	4.95E-02	-1.006	1.430	-0.704	4.91E-01	5.155	2.415	2.134	4.68E-02
CIBAR1P2	-346.429	157.283	-2.203	4.09E-02	-0.741	2.176	-0.340	7.37E-01	7.844	3.675	2.134	4.68E-02
SLC30A4	56.749	25.593	2.217	3.97E-02	0.164	0.354	0.463	6.49E-01	-1.276	0.598	-2.134	4.68E-02
LOC1053745	-153.611	80.701	-1.903	7.31E-02	-2.727	1.116	-2.443	2.51E-02	4.023	1.886	2.134	4.69E-02
FAM74A1	102.721	41.716	2.462	2.41E-02	-0.257	0.577	-0.445	6.62E-01	-2.080	0.975	-2.134	4.69E-02
LOC1079867	-102.989	49.767	-2.069	5.32E-02	-1.271	0.688	-1.846	8.14E-02	2.480	1.163	2.133	4.69E-02
LOC1027243	134.237	65.424	2.052	5.50E-02	1.130	0.905	1.248	2.28E-01	-3.260	1.529	-2.133	4.70E-02
CRTC1	35.544	15.583	2.281	3.49E-02	0.142	0.216	0.660	5.17E-01	-0.776	0.364	-2.132	4.70E-02
DEFB109B	-32.330	19.277	-1.677	1.11E-01	-1.194	0.267	-4.479	2.90E-04	0.960	0.450	2.132	4.71E-02
DPRX	237.785	109.626	2.169	4.37E-02	1.316	1.516	0.868	3.97E-01	-5.460	2.561	-2.132	4.71E-02
CSAG3	44.528	21.652	2.057	5.45E-02	0.214	0.299	0.715	4.84E-01	-1.078	0.506	-2.131	4.71E-02
SDHAF4	-399.486	177.472	-2.251	3.71E-02	0.224	2.455	0.091	9.28E-01	8.835	4.147	2.131	4.72E-02
LINC02051	183.115	100.477	1.822	8.50E-02	3.165	1.390	2.277	3.52E-02	-5.001	2.348	-2.130	4.72E-02

CACNA1A	-92.952	44.398	-2.094	5.07E-02	-0.299	0.614	-0.488	6.32E-01	2.210	1.037	2.130	4.72E-02
NDUFB1	-80.909	42.870	-1.887	7.54E-02	-1.557	0.593	-2.626	1.71E-02	2.133	1.002	2.130	4.72E-02
LOC1122680	142.631	60.726	2.349	3.05E-02	-0.212	0.840	-0.252	8.04E-01	-3.022	1.419	-2.130	4.73E-02
LOC1079868	-425.493	189.679	-2.243	3.77E-02	0.096	2.624	0.036	9.71E-01	9.437	4.432	2.129	4.73E-02
CD200R1L.AS	-557.543	255.686	-2.181	4.27E-02	-1.429	3.537	-0.404	6.91E-01	12.719	5.974	2.129	4.73E-02
SOX5.AS1	-189.018	93.087	-2.031	5.73E-02	-1.729	1.288	-1.342	1.96E-01	4.630	2.175	2.129	4.74E-02
PNOC	-73.809	35.959	-2.053	5.49E-02	-0.550	0.497	-1.106	2.83E-01	1.788	0.840	2.128	4.74E-02
POU5F1P3	-71.372	34.904	-2.045	5.58E-02	-0.691	0.483	-1.431	1.70E-01	1.735	0.816	2.128	4.74E-02
NME5	-138.860	65.414	-2.123	4.79E-02	-0.490	0.905	-0.542	5.95E-01	3.251	1.528	2.127	4.75E-02
RNF139	-210.338	94.111	-2.235	3.83E-02	-0.358	1.302	-0.275	7.86E-01	4.677	2.199	2.127	4.75E-02
LOC1053695	65.243	30.058	2.171	4.36E-02	0.221	0.416	0.531	6.02E-01	-1.493	0.702	-2.126	4.76E-02
MIR4463	-289.222	155.389	-1.861	7.91E-02	-7.715	2.149	-3.590	2.10E-03	7.715	3.631	2.125	4.77E-02
LOC1079842	74.201	33.639	2.206	4.06E-02	0.260	0.465	0.559	5.83E-01	-1.670	0.786	-2.124	4.77E-02
LOC1053789	124.299	51.701	2.404	2.72E-02	-0.106	0.715	-0.148	8.84E-01	-2.566	1.208	-2.124	4.78E-02
AP1B1	60.394	27.785	2.174	4.33E-02	0.274	0.384	0.714	4.84E-01	-1.379	0.649	-2.123	4.78E-02
BCL2A1	-617.193	280.099	-2.203	4.08E-02	-0.581	3.874	-0.150	8.83E-01	13.896	6.544	2.123	4.78E-02
ZRANB3	77.378	30.173	2.565	1.95E-02	-0.494	0.417	-1.185	2.52E-01	-1.496	0.705	-2.122	4.80E-02
EEF2K	-1.325	0.690	-1.920	7.08E-02	-0.034	0.010	-3.584	2.12E-03	0.034	0.016	2.122	4.80E-02
TM2D1	156.338	72.458	2.158	4.47E-02	0.772	1.002	0.770	4.51E-01	-3.591	1.693	-2.121	4.80E-02
LOC1053785	-294.625	134.211	-2.195	4.15E-02	-0.002	1.856	-0.001	9.99E-01	6.650	3.136	2.121	4.81E-02
SMIM30	-503.356	227.158	-2.216	3.98E-02	-0.734	3.142	-0.234	8.18E-01	11.255	5.307	2.121	4.81E-02
MUC5AC	1.859	0.958	1.941	6.81E-02	0.027	0.013	2.002	6.05E-02	-0.047	0.022	-2.120	4.82E-02
SCARNA26A	512.949	223.423	2.296	3.39E-02	0.685	3.090	0.222	8.27E-01	-11.065	5.220	-2.120	4.82E-02
LOC1122679	-0.638	0.311	-2.052	5.50E-02	-0.006	0.004	-1.402	1.78E-01	0.015	0.007	2.120	4.82E-02
MIR93	-258.010	125.722	-2.052	5.50E-02	-2.438	1.739	-1.402	1.78E-01	6.226	2.937	2.120	4.82E-02
SNORA18	-26.062	12.699	-2.052	5.50E-02	-0.246	0.176	-1.402	1.78E-01	0.629	0.297	2.120	4.82E-02
SMG1P2_1	-2.455	1.196	-2.052	5.50E-02	-0.023	0.017	-1.402	1.78E-01	0.059	0.028	2.120	4.82E-02
HMGN3	-266.575	125.662	-2.121	4.80E-02	-1.111	1.738	-0.639	5.31E-01	6.223	2.936	2.120	4.82E-02
LOC1027237	-28.418	13.756	-2.066	5.35E-02	-0.218	0.190	-1.144	2.68E-01	0.681	0.321	2.119	4.82E-02
TADA1	89.626	35.777	2.505	2.21E-02	-0.399	0.495	-0.807	4.30E-01	-1.772	0.836	-2.119	4.82E-02
SNORD71	-475.928	239.541	-1.987	6.24E-02	-6.704	3.313	-2.023	5.81E-02	11.859	5.597	2.119	4.83E-02
LINC01187	-471.941	214.222	-2.203	4.09E-02	-0.582	2.963	-0.196	8.46E-01	10.604	5.005	2.119	4.83E-02
MIR3125	52.544	23.124	2.272	3.56E-02	0.108	0.320	0.338	7.40E-01	-1.145	0.540	-2.118	4.83E-02
MIR130B	149.943	65.988	2.272	3.56E-02	0.308	0.913	0.338	7.40E-01	-3.266	1.542	-2.118	4.83E-02
LINC02203	4.214	1.855	2.272	3.56E-02	0.009	0.026	0.338	7.40E-01	-0.092	0.043	-2.118	4.83E-02
LINC01621	-32.936	21.305	-1.546	1.40E-01	-1.609	0.295	-5.460	3.47E-05	1.054	0.498	2.118	4.83E-02
LOC1019273	131.185	54.692	2.399	2.75E-02	-0.017	0.756	-0.023	9.82E-01	-2.707	1.278	-2.118	4.83E-02
CDC42SE1	58.608	24.876	2.356	3.00E-02	-0.128	0.344	-0.372	7.15E-01	-1.231	0.581	-2.118	4.83E-02
WDR93	69.943	31.157	2.245	3.76E-02	0.087	0.431	0.201	8.43E-01	-1.541	0.728	-2.117	4.84E-02
TNNT3_1	28.940	11.974	2.417	2.65E-02	-0.046	0.166	-0.280	7.82E-01	-0.592	0.280	-2.117	4.84E-02
MYADM	88.552	37.433	2.366	2.94E-02	0.058	0.518	0.112	9.12E-01	-1.852	0.875	-2.117	4.84E-02
LOC1079853	181.372	71.434	2.539	2.06E-02	-0.918	0.988	-0.929	3.65E-01	-3.532	1.669	-2.116	4.85E-02
DSCC1	68.494	30.391	2.254	3.69E-02	-0.003	0.420	-0.007	9.94E-01	-1.503	0.710	-2.116	4.85E-02
MTHFD1	-177.767	86.967	-2.044	5.59E-02	-1.350	1.203	-1.123	2.76E-01	4.300	2.032	2.116	4.85E-02
LINC00160	116.479	60.069	1.939	6.83E-02	1.352	0.831	1.627	1.21E-01	-2.969	1.403	-2.115	4.86E-02
LOC1079845	-119.701	58.178	-2.057	5.44E-02	-1.072	0.805	-1.332	1.99E-01	2.875	1.359	2.115	4.86E-02
PPIP5K1	67.602	33.886	1.995	6.14E-02	0.679	0.469	1.449	1.65E-01	-1.674	0.792	-2.115	4.87E-02
HAVCR1P1	89.981	35.927	2.505	2.21E-02	-0.501	0.497	-1.009	3.26E-01	-1.775	0.839	-2.114	4.87E-02
LINC01322	345.194	146.675	2.353	3.02E-02	0.787	2.029	0.388	7.03E-01	-7.242	3.427	-2.113	4.88E-02
MARCHF10	-95.000	42.973	-2.211	4.02E-02	-0.195	0.594	-0.328	7.47E-01	2.122	1.004	2.113	4.88E-02
LINC00609	138.295	60.201	2.297	3.38E-02	-0.047	0.833	-0.056	9.56E-01	-2.972	1.407	-2.113	4.89E-02
ALOX12B	49.777	24.642	2.020	5.85E-02	0.438	0.341	1.286	2.15E-01	-1.216	0.576	-2.112	4.89E-02
LOC1053746	187.855	83.195	2.258	3.66E-02	0.569	1.151	0.495	6.27E-01	-4.106	1.944	-2.112	4.89E-02

MAN1B1	103.702	49.146	2.110	4.91E-02	0.448	0.680	0.659	5.18E-01	-2.426	1.148	-2.112	4.89E-02
LOC10537399	-472.014	211.991	-2.227	3.90E-02	-0.245	2.932	-0.084	9.34E-01	10.463	4.953	2.112	4.89E-02
LOC10798490	166.065	71.714	2.316	3.26E-02	0.050	0.992	0.051	9.60E-01	-3.539	1.676	-2.112	4.89E-02
LOC10537805	-391.824	183.181	-2.139	4.64E-02	-1.219	2.534	-0.481	6.36E-01	9.039	4.280	2.112	4.89E-02
GDF6	43.029	17.811	2.416	2.65E-02	-0.207	0.246	-0.841	4.11E-01	-0.879	0.416	-2.112	4.90E-02
LINC02176	126.901	57.291	2.215	3.99E-02	0.579	0.792	0.730	4.75E-01	-2.826	1.339	-2.111	4.90E-02
LOC10537369	-137.351	66.383	-2.069	5.32E-02	-1.106	0.918	-1.205	2.44E-01	3.275	1.551	2.111	4.90E-02
RNF41	-80.321	37.394	-2.148	4.56E-02	-0.139	0.517	-0.268	7.92E-01	1.845	0.874	2.111	4.90E-02
MED7	-153.007	73.067	-2.094	5.07E-02	-1.010	1.011	-1.000	3.31E-01	3.604	1.707	2.111	4.90E-02
ATP6V0CP3	52.035	22.874	2.275	3.54E-02	0.106	0.316	0.336	7.41E-01	-1.128	0.534	-2.110	4.91E-02
TRS.AGA5.1	-455.224	208.679	-2.181	4.27E-02	-0.865	2.886	-0.300	7.68E-01	10.288	4.876	2.110	4.91E-02
LINC02197_1	7.829	3.260	2.401	2.73E-02	-0.019	0.045	-0.428	6.74E-01	-0.161	0.076	-2.110	4.91E-02
AMOTL1	-124.607	58.127	-2.144	4.60E-02	-0.161	0.804	-0.201	8.43E-01	2.865	1.358	2.110	4.91E-02
MIR580	631.580	259.010	2.438	2.53E-02	-2.046	3.583	-0.571	5.75E-01	-12.761	6.052	-2.109	4.93E-02
LOC10537024	-973.473	447.761	-2.174	4.33E-02	-1.670	6.193	-0.270	7.90E-01	22.056	10.462	2.108	4.93E-02
RRAGD	54.306	24.135	2.250	3.72E-02	0.032	0.334	0.096	9.25E-01	-1.189	0.564	-2.108	4.93E-02
LOC10272435	165.830	71.181	2.330	3.17E-02	-0.112	0.985	-0.114	9.10E-01	-3.505	1.663	-2.108	4.93E-02
FIBCD1	88.468	38.707	2.286	3.46E-02	0.256	0.535	0.479	6.38E-01	-1.906	0.904	-2.107	4.94E-02
FMO6P	-399.231	183.299	-2.178	4.29E-02	-0.626	2.535	-0.247	8.08E-01	9.025	4.283	2.107	4.94E-02
LOC10537764	185.944	81.078	2.293	3.41E-02	0.026	1.121	0.023	9.82E-01	-3.991	1.894	-2.107	4.94E-02
LINC02712	252.511	107.166	2.356	3.00E-02	-0.050	1.482	-0.034	9.74E-01	-5.275	2.504	-2.107	4.94E-02
ZSWIM9	-133.881	64.786	-2.067	5.35E-02	-0.311	0.896	-0.347	7.32E-01	3.189	1.514	2.106	4.95E-02
TMEM190	-125.095	59.817	-2.091	5.10E-02	-0.913	0.827	-1.103	2.85E-01	2.943	1.398	2.106	4.95E-02
LOC10537080	46.484	23.104	2.012	5.94E-02	0.243	0.320	0.761	4.57E-01	-1.136	0.540	-2.105	4.96E-02
CPB2	-108.008	52.627	-2.052	5.50E-02	-1.267	0.728	-1.740	9.88E-02	2.588	1.230	2.105	4.96E-02
PCAT7	-123.856	58.145	-2.130	4.72E-02	-0.404	0.804	-0.502	6.22E-01	2.859	1.359	2.104	4.97E-02
RMDN2.AS1	112.755	46.890	2.405	2.72E-02	-0.332	0.649	-0.511	6.15E-01	-2.305	1.096	-2.104	4.97E-02
TCP11	134.050	54.136	2.476	2.34E-02	-0.531	0.749	-0.709	4.88E-01	-2.661	1.265	-2.104	4.97E-02
PLD2	-254.839	117.336	-2.172	4.35E-02	0.173	1.623	0.106	9.16E-01	5.765	2.742	2.103	4.98E-02
LOC10042221	141.325	67.218	2.103	4.98E-02	1.233	0.930	1.327	2.01E-01	-3.302	1.571	-2.103	4.98E-02
TDRKH.AS1	-443.635	202.688	-2.189	4.20E-02	-0.892	2.804	-0.318	7.54E-01	9.958	4.736	2.103	4.98E-02
LOC10537619	-188.590	91.731	-2.056	5.46E-02	-2.173	1.269	-1.713	1.04E-01	4.506	2.143	2.103	4.98E-02
HM13	-179.397	83.286	-2.154	4.50E-02	-0.325	1.152	-0.282	7.81E-01	4.090	1.946	2.102	4.99E-02
LOC10798499	-393.527	182.379	-2.158	4.47E-02	-0.489	2.523	-0.194	8.49E-01	8.957	4.261	2.102	4.99E-02
NOMO1_1	16.564	7.715	2.147	4.57E-02	0.076	0.107	0.715	4.84E-01	-0.379	0.180	-2.101	5.00E-02
MIR378C	-44.224	23.882	-1.852	8.05E-02	-1.172	0.330	-3.549	2.29E-03	1.172	0.558	2.101	5.00E-02
