# Supplementary material for: RNA Sequencing of Intestinal Enterocytes Pre- and Post-Roux-en-Y Gastric Bypass Reveals Alteration in Gene Expression Related to Enterocyte Differentiation, Restitution, and Obesity with Regulation by Schlafen 12
Source: Cells. 2022 Oct 18;11(20):3283. doi: 10.3390/cells11203283 (PMC9601224; doi:10.3390/cells11203283)
Supplement: Supplementary file 1 [file cells-11-03283-s001.zip › Table S4.pdf]

	Fold change and relative BMI Correlation	
Genes	Correlation Coefficient	Correlation_Pval
SSMEM1	9.45E-01	1.12E-05
LOC107985272	9.09E-01	1.06E-04
PRF1	9.09E-01	1.06E-04
GTF2I	9.00E-01	1.60E-04
LOC105376613	9.00E-01	1.60E-04
LOC102723548	9.00E-01	1.60E-04
WNK3	8.91E-01	2.33E-04
P3H2.AS1	8.82E-01	3.30E-04
LOC105378213	8.82E-01	3.30E-04
LOC107984554	8.82E-01	3.30E-04
ADGRG2	8.82E-01	3.30E-04
LOC101929752	8.73E-01	4.55E-04
ZDHHC19	8.73E-01	4.55E-04
CREB3L2.AS1	8.73E-01	4.55E-04
LOC105376088	8.73E-01	4.55E-04
COL5A1.AS1	8.73E-01	4.55E-04
DYRK4	8.73E-01	4.55E-04
CMC4	8.73E-01	4.55E-04
LOC105373484	8.64E-01	6.12E-04
LOC105374759	8.64E-01	6.12E-04
CRISP2	8.64E-01	6.12E-04
LOC101928254	8.64E-01	6.12E-04
LOC112268042	8.64E-01	6.12E-04
IFITM1	8.64E-01	6.12E-04
PCDHAC1	8.55E-01	8.07E-04
USP17L4	8.55E-01	8.07E-04
MKX.AS1	8.55E-01	8.07E-04
LOC105369565	8.55E-01	8.07E-04
TESPA1	8.55E-01	8.07E-04
LOC105370092	8.55E-01	8.07E-04
ACOT1	8.55E-01	8.07E-04
LOC105372233	8.55E-01	8.07E-04
DEFB127	8.55E-01	8.07E-04
LINC01204	8.55E-01	8.07E-04
ARHGEF2.AS1	8.45E-01	1.05E-03
LOC105373924	8.45E-01	1.05E-03
CCR5	8.45E-01	1.05E-03

C3orf85	8.45E-01	1.05E-03
LOC105374645	8.45E-01	1.05E-03
LOC105378211	8.45E-01	1.05E-03
TRY.GTA1.1	8.45E-01	1.05E-03
LOC105379370	8.45E-01	1.05E-03
RC3H2	8.45E-01	1.05E-03
CAND1.11	8.45E-01	1.05E-03
KAT5	8.45E-01	1.05E-03
KLRC4	8.45E-01	1.05E-03
BMS1P18	8.45E-01	1.05E-03
FANCA	8.45E-01	1.05E-03
LOC101929552	8.45E-01	1.05E-03
MIR3156.3	8.45E-01	1.05E-03
LOC105372977	8.45E-01	1.05E-03
PKD1P6.NPIPP1_1	8.45E-01	1.05E-03
CYP4B1	8.36E-01	1.33E-03
LOC105373394	8.36E-01	1.33E-03
SLC2A9.AS1	8.36E-01	1.33E-03
PCDHGA1	8.36E-01	1.33E-03
USP17L1	8.36E-01	1.33E-03
LOC105370088	8.36E-01	1.33E-03
LOC101927864	8.36E-01	1.33E-03
VMP1	8.36E-01	1.33E-03
TNRC6C	8.36E-01	1.33E-03
ZNF567	8.36E-01	1.33E-03
LOC105372497	8.36E-01	1.33E-03
TOP1	8.36E-01	1.33E-03
PABPC1L2A	8.36E-01	1.33E-03
GJC2	8.27E-01	1.68E-03
UHRF1BP1	8.27E-01	1.68E-03
TAX1BP1.AS1	8.27E-01	1.68E-03
LOC105375599	8.27E-01	1.68E-03
LOC100287846	8.27E-01	1.68E-03
C8orf33	8.27E-01	1.68E-03
CARS1.AS1	8.27E-01	1.68E-03
OR2AG2	8.27E-01	1.68E-03
LINC00958	8.27E-01	1.68E-03
CCDC179	8.27E-01	1.68E-03
SNORD6	8.27E-01	1.68E-03
LOC101927437	8.27E-01	1.68E-03
SKAP1	8.27E-01	1.68E-03

LOC105372069	8.27E-01	1.68E-03
UHRF1	8.27E-01	1.68E-03
IFNL1	8.27E-01	1.68E-03
FRG1DP	8.27E-01	1.68E-03
LOC105373083	8.27E-01	1.68E-03
LOC105373223	8.18E-01	2.08E-03
LOC105373616	8.18E-01	2.08E-03
CRYGB	8.18E-01	2.08E-03
TTLL4	8.18E-01	2.08E-03
LOC107986019	8.18E-01	2.08E-03
ASTE1	8.18E-01	2.08E-03
NPHP3.ACAD11	8.18E-01	2.08E-03
TMEM131L	8.18E-01	2.08E-03
LOC105377529	8.18E-01	2.08E-03
LOC107986643	8.18E-01	2.08E-03
LOC105378036	8.18E-01	2.08E-03
LOC100506664	8.18E-01	2.08E-03
MCM4	8.18E-01	2.08E-03
LOC107986961	8.18E-01	2.08E-03
LOC105375952	8.18E-01	2.08E-03
OR5AK4P	8.18E-01	2.08E-03
PGR	8.18E-01	2.08E-03
LOC105370284	8.18E-01	2.08E-03
PARP16	8.18E-01	2.08E-03
LOC107983949	8.18E-01	2.08E-03
LINC01970	8.18E-01	2.08E-03
ZNF516.DT	8.18E-01	2.08E-03
LINC01727	8.18E-01	2.08E-03
LOC107985570	8.18E-01	2.08E-03
DCAF8L1	8.18E-01	2.08E-03
LOC102724491	8.18E-01	2.08E-03
MACO1	8.09E-01	2.56E-03
LOC107985238	8.09E-01	2.56E-03
LOC107985460	8.09E-01	2.56E-03
LOC107985877	8.09E-01	2.56E-03
MIR216A	8.09E-01	2.56E-03
LOC105377033	8.09E-01	2.56E-03
GUCA1C	8.09E-01	2.56E-03
PYDC2	8.09E-01	2.56E-03
MIR548I2	8.09E-01	2.56E-03
LOC285593	8.09E-01	2.56E-03

GMCL2	8.09E-01	2.56E-03
TUBE1	8.09E-01	2.56E-03
HOXA11	8.09E-01	2.56E-03
CLDN12	8.09E-01	2.56E-03
PMS2P1	8.09E-01	2.56E-03
TRS.AGA2.5	8.09E-01	2.56E-03
LOC112268054	8.09E-01	2.56E-03
LOC107987142	8.09E-01	2.56E-03
FAM166A	8.09E-01	2.56E-03
C11orf1	8.09E-01	2.56E-03
LOC105369947	8.09E-01	2.56E-03
SNORD109B	8.09E-01	2.56E-03
MRPL46	8.09E-01	2.56E-03
LOC105370997	8.09E-01	2.56E-03
SNAI3.AS1	8.09E-01	2.56E-03
LINC01428	8.09E-01	2.56E-03
LOC105372782	8.09E-01	2.56E-03
IGLV1.62	8.09E-01	2.56E-03
C22orf15	8.09E-01	2.56E-03
LOC105373293	8.09E-01	2.56E-03
TCEAL5	8.09E-01	2.56E-03
LOC101928832	8.09E-01	2.56E-03
SALL3_1	8.09E-01	2.56E-03
LOC105374866	8.09E-01	2.56E-03
MIR2682	8.00E-01	3.11E-03
LOC107985795	8.00E-01	3.11E-03
ORC2	8.00E-01	3.11E-03
EIF2A	8.00E-01	3.11E-03
MIR551B	8.00E-01	3.11E-03
LOC105374296	8.00E-01	3.11E-03
STPG2.AS1	8.00E-01	3.11E-03
LINC01061	8.00E-01	3.11E-03
ZNF330	8.00E-01	3.11E-03
LOC105377516	8.00E-01	3.11E-03
LOC105374955	8.00E-01	3.11E-03
LOC105377998	8.00E-01	3.11E-03
LOC115308161	8.00E-01	3.11E-03
DEFB109B	8.00E-01	3.11E-03
STK3	8.00E-01	3.11E-03
LOC107986968	8.00E-01	3.11E-03
LOC112268045	8.00E-01	3.11E-03

MIR181B2	8.00E-01	3.11E-03
LOC101448202	8.00E-01	3.11E-03
MIR3611	8.00E-01	3.11E-03
LINC00866	8.00E-01	3.11E-03
LINC02747	8.00E-01	3.11E-03
MIRLET7A2	8.00E-01	3.11E-03
LINC02156	8.00E-01	3.11E-03
MIR1197	8.00E-01	3.11E-03
LOC105376730	8.00E-01	3.11E-03
LOC105371381	8.00E-01	3.11E-03
LINC01926	8.00E-01	3.11E-03
LINC01534	8.00E-01	3.11E-03
LSM14B	8.00E-01	3.11E-03
PIK3IP1	8.00E-01	3.11E-03
OCRL	8.00E-01	3.11E-03
HTATSF1	8.00E-01	3.11E-03
SLC25A24_1	8.00E-01	3.11E-03
LOC112268402	8.00E-01	3.11E-03
PRR23D1_1	8.00E-01	3.11E-03
ROR1.AS1	7.91E-01	3.75E-03
LOC101929788	7.91E-01	3.75E-03
LOC107985996	7.91E-01	3.75E-03
SLC9A9.AS1	7.91E-01	3.75E-03
TMPRSS11GP	7.91E-01	3.75E-03
JCHAIN	7.91E-01	3.75E-03
LINC02057	7.91E-01	3.75E-03
TTC37	7.91E-01	3.75E-03
CSNK1A1	7.91E-01	3.75E-03
FAXDC2	7.91E-01	3.75E-03
LOC107986632	7.91E-01	3.75E-03
STX7	7.91E-01	3.75E-03
SLC22A3	7.91E-01	3.75E-03
LOC105378094	7.91E-01	3.75E-03
LINC01176	7.91E-01	3.75E-03
LOC105375238	7.91E-01	3.75E-03
LOC105375326	7.91E-01	3.75E-03
STC1	7.91E-01	3.75E-03
ARHGAP12	7.91E-01	3.75E-03
LINC02683	7.91E-01	3.75E-03
SHANK2	7.91E-01	3.75E-03
LOC105369552	7.91E-01	3.75E-03

LINC00400	7.91E-01	3.75E-03
LOC107984566	7.91E-01	3.75E-03
LOC101928462	7.91E-01	3.75E-03
LOC107984691	7.91E-01	3.75E-03
SNORD116.3	7.91E-01	3.75E-03
LOC107984727	7.91E-01	3.75E-03
OR2C1	7.91E-01	3.75E-03
SYT17	7.91E-01	3.75E-03
BBS2	7.91E-01	3.75E-03
LOC105376778	7.91E-01	3.75E-03
LOC105372081	7.91E-01	3.75E-03
MIR522	7.91E-01	3.75E-03
ZGPAT	7.91E-01	3.75E-03
LOC107985494	7.91E-01	3.75E-03
LOC105372836	7.91E-01	3.75E-03
LOC107985376	7.82E-01	4.47E-03
MTMR9LP	7.82E-01	4.47E-03
LINC02884	7.82E-01	4.47E-03
RPL21P28	7.82E-01	4.47E-03
LOC107985771	7.82E-01	4.47E-03
LOC105373575	7.82E-01	4.47E-03
TFCP2L1	7.82E-01	4.47E-03
LOC107986067	7.82E-01	4.47E-03
LINC00882	7.82E-01	4.47E-03
LOC105374307	7.82E-01	4.47E-03
DLG1.AS1	7.82E-01	4.47E-03
USP17L6P	7.82E-01	4.47E-03
ARHGAP24	7.82E-01	4.47E-03
LOC107986309	7.82E-01	4.47E-03
LINC01340	7.82E-01	4.47E-03
PCDHB4	7.82E-01	4.47E-03
LOC107986483	7.82E-01	4.47E-03
LOC107986601	7.82E-01	4.47E-03
SNORD166	7.82E-01	4.47E-03
LOC105375363	7.82E-01	4.47E-03
KMT2E	7.82E-01	4.47E-03
LOC105379771	7.82E-01	4.47E-03
LOC107986905	7.82E-01	4.47E-03
BAAT	7.82E-01	4.47E-03
GLUD1P2	7.82E-01	4.47E-03
OR52K2	7.82E-01	4.47E-03

STK33	7.82E-01	4.47E-03
OR1S1	7.82E-01	4.47E-03
LOC107984334	7.82E-01	4.47E-03
LOC105378956	7.82E-01	4.47E-03
LINC01500	7.82E-01	4.47E-03
LOC100129540	7.82E-01	4.47E-03
LOC105371060	7.82E-01	4.47E-03
LOC105379460	7.82E-01	4.47E-03
VKORC1	7.82E-01	4.47E-03
MT1B	7.82E-01	4.47E-03
LOC105371413	7.82E-01	4.47E-03
TCF4	7.82E-01	4.47E-03
LINC01529	7.82E-01	4.47E-03
LL21NC02.21A1.1	7.82E-01	4.47E-03
MRTFA	7.82E-01	4.47E-03
TSIX	7.82E-01	4.47E-03
PABPC5.AS1	7.82E-01	4.47E-03
PSMD10	7.82E-01	4.47E-03
SOX3	7.82E-01	4.47E-03
MAGEA12	7.82E-01	4.47E-03
LOC105371625	7.73E-01	5.30E-03
LOC105373504	7.73E-01	5.30E-03
LOC105377099	7.73E-01	5.30E-03
NR1I2	7.73E-01	5.30E-03
LINC02496	7.73E-01	5.30E-03
LOC101928819	7.73E-01	5.30E-03
MSX2	7.73E-01	5.30E-03
LOC105377882	7.73E-01	5.30E-03
TRQ.TTG4.1	7.73E-01	5.30E-03
LOC105375155	7.73E-01	5.30E-03
DDX56	7.73E-01	5.30E-03
MIR548O	7.73E-01	5.30E-03
LOC102724407	7.73E-01	5.30E-03
TMEM139.AS1	7.73E-01	5.30E-03
LOC105376369	7.73E-01	5.30E-03
ANK3.DT	7.73E-01	5.30E-03
NUDT9P1	7.73E-01	5.30E-03
TIAL1	7.73E-01	5.30E-03
LOC105376642	7.73E-01	5.30E-03
ARHGAP42	7.73E-01	5.30E-03
DAZAP2	7.73E-01	5.30E-03

LOC105369811	7.73E-01	5.30E-03
TRAV22	7.73E-01	5.30E-03
PRORP	7.73E-01	5.30E-03
ACOT2	7.73E-01	5.30E-03
NDUFB1	7.73E-01	5.30E-03
CTF1	7.73E-01	5.30E-03
LOC107985063	7.73E-01	5.30E-03
LOC105371934	7.73E-01	5.30E-03
CCDC103	7.73E-01	5.30E-03
VEZF1	7.73E-01	5.30E-03
MTMR4	7.73E-01	5.30E-03
MIR3975	7.73E-01	5.30E-03
LOC105372074	7.73E-01	5.30E-03
LOC107985434	7.73E-01	5.30E-03
TCF20	7.73E-01	5.30E-03
KLHL34	7.73E-01	5.30E-03
MAGEB4	7.73E-01	5.30E-03
TAS2R19_2	7.73E-01	5.30E-03
FUCA1	7.64E-01	6.23E-03
MPL	7.64E-01	6.23E-03
LOC647070	7.64E-01	6.23E-03
LINC01740	7.64E-01	6.23E-03
LOC107985372	7.64E-01	6.23E-03
UGT1A6	7.64E-01	6.23E-03
QDPR	7.64E-01	6.23E-03
STIM2	7.64E-01	6.23E-03
LOC101930370	7.64E-01	6.23E-03
LINC01554	7.64E-01	6.23E-03
SLC36A2	7.64E-01	6.23E-03
TNFRSF10C	7.64E-01	6.23E-03
TCEA1	7.64E-01	6.23E-03
CYP7B1	7.64E-01	6.23E-03
C9orf64	7.64E-01	6.23E-03
SLC16A12.AS1	7.64E-01	6.23E-03
SPX	7.64E-01	6.23E-03
LINC02305	7.64E-01	6.23E-03
LINC00637	7.64E-01	6.23E-03
LOC105371301	7.64E-01	6.23E-03
CRYBA1	7.64E-01	6.23E-03
STXBP4	7.64E-01	6.23E-03
LOC112267896	7.64E-01	6.23E-03

DNAJB1	7.64E-01	6.23E-03
VN1R4	7.64E-01	6.23E-03
RBM39	7.64E-01	6.23E-03
CYP4F29P	7.64E-01	6.23E-03
KRTAP25.1	7.64E-01	6.23E-03
SIK1	7.64E-01	6.23E-03
LINC01637	7.64E-01	6.23E-03
LINC01282	7.64E-01	6.23E-03
SMARCA1	7.64E-01	6.23E-03
UQCRHL	7.55E-01	7.28E-03
LOC105376892	7.55E-01	7.28E-03
RGS2.AS1	7.55E-01	7.28E-03
LRRN2	7.55E-01	7.28E-03
AURKAP1	7.55E-01	7.28E-03
QPCT	7.55E-01	7.28E-03
CCT7	7.55E-01	7.28E-03
ACAA1	7.55E-01	7.28E-03
LINC01997	7.55E-01	7.28E-03
ELOVL7	7.55E-01	7.28E-03
GUSBP3	7.55E-01	7.28E-03
LINC01338	7.55E-01	7.28E-03
GLP1R	7.55E-01	7.28E-03
LOC107986633	7.55E-01	7.28E-03
LOC105375202	7.55E-01	7.28E-03
LOC112267986	7.55E-01	7.28E-03
LOC107986921	7.55E-01	7.28E-03
KCNU1	7.55E-01	7.28E-03
SAMD12.AS1	7.55E-01	7.28E-03
HACD4	7.55E-01	7.28E-03
OR1J1	7.55E-01	7.28E-03
LOC105376325	7.55E-01	7.28E-03
ACTA2.AS1	7.55E-01	7.28E-03
IDE	7.55E-01	7.28E-03
LOC107984258	7.55E-01	7.28E-03
NSMCE4A	7.55E-01	7.28E-03
SPRNP1	7.55E-01	7.28E-03
OTUB1	7.55E-01	7.28E-03
MAP6	7.55E-01	7.28E-03
LINC02711	7.55E-01	7.28E-03
LOC645485	7.55E-01	7.28E-03
LINC00333	7.55E-01	7.28E-03

LINC00397	7.55E-01	7.28E-03
LOC107984655	7.55E-01	7.28E-03
LOC105371069	7.55E-01	7.28E-03
LOC107984831	7.55E-01	7.28E-03
OR2Z1	7.55E-01	7.28E-03
ADIG	7.55E-01	7.28E-03
KRTAP20.4	7.55E-01	7.28E-03
SSR4P1	7.55E-01	7.28E-03
LOC105372985	7.55E-01	7.28E-03
SCUBE1.AS2	7.55E-01	7.28E-03
PIGA	7.55E-01	7.28E-03
OPHN1	7.55E-01	7.28E-03
UPRT	7.55E-01	7.28E-03
RAB39B	7.55E-01	7.28E-03
LOC102723722_3	7.55E-01	7.28E-03
LINC02810	7.45E-01	8.45E-03
NSUN4	7.45E-01	8.45E-03
LOC101929935	7.45E-01	8.45E-03
PIN1P1	7.45E-01	8.45E-03
LINC02795	7.45E-01	8.45E-03
PKN2.AS1	7.45E-01	8.45E-03
LINC02806	7.45E-01	8.45E-03
ERO1B	7.45E-01	8.45E-03
LOC105374761	7.45E-01	8.45E-03
LOC101926959	7.45E-01	8.45E-03
GRM7.AS2	7.45E-01	8.45E-03
LOC107986187	7.45E-01	8.45E-03
CHRNA9	7.45E-01	8.45E-03
PARM1	7.45E-01	8.45E-03
LOC100506858	7.45E-01	8.45E-03
MIR378E	7.45E-01	8.45E-03
EFCAB9	7.45E-01	8.45E-03
TRIM52.AS1	7.45E-01	8.45E-03
LY86	7.45E-01	8.45E-03
LOC101927588	7.45E-01	8.45E-03
VIM.AS1	7.45E-01	8.45E-03
BUB3	7.45E-01	8.45E-03
PLAAT3	7.45E-01	8.45E-03
ALG9	7.45E-01	8.45E-03
OR10S1	7.45E-01	8.45E-03
LOC100130075	7.45E-01	8.45E-03

LINC02820	7.45E-01	8.45E-03
LINC02258	7.45E-01	8.45E-03
LOC101928300	7.45E-01	8.45E-03
MPHOSPH8	7.45E-01	8.45E-03
MIR548AS	7.45E-01	8.45E-03
LOC107984652	7.45E-01	8.45E-03
RAD51.AS1	7.45E-01	8.45E-03
CA12	7.45E-01	8.45E-03
MEX3B	7.45E-01	8.45E-03
LOC105370955	7.45E-01	8.45E-03
LOC105371089	7.45E-01	8.45E-03
TMEM11	7.45E-01	8.45E-03
LOC112268202	7.45E-01	8.45E-03
LINC00673	7.45E-01	8.45E-03
LOC105372185	7.45E-01	8.45E-03
CD320	7.45E-01	8.45E-03
JUNB	7.45E-01	8.45E-03
ZNF675	7.45E-01	8.45E-03
ZNF582.AS1	7.45E-01	8.45E-03
LOC105372510	7.45E-01	8.45E-03
JAG1	7.45E-01	8.45E-03
FRG1CP	7.45E-01	8.45E-03
ACTR5	7.45E-01	8.45E-03
CTSZ	7.45E-01	8.45E-03
TST	7.45E-01	8.45E-03
ERAS	7.45E-01	8.45E-03
OTUD6A	7.45E-01	8.45E-03
VGLL1	7.45E-01	8.45E-03
BGN	7.45E-01	8.45E-03
LOC101927441	7.36E-01	9.76E-03
OLFM3	7.36E-01	9.76E-03
TAF13	7.36E-01	9.76E-03
LOC107985454	7.36E-01	9.76E-03
LOC102723543	7.36E-01	9.76E-03
LOC107985357	7.36E-01	9.76E-03
LINC00570	7.36E-01	9.76E-03
LOC105373545	7.36E-01	9.76E-03
LRRC58	7.36E-01	9.76E-03
NUDT16L2P	7.36E-01	9.76E-03
LINC01327	7.36E-01	9.76E-03
PIGY.DT	7.36E-01	9.76E-03

LOC105377382	7.36E-01	9.76E-03
LINC01033	7.36E-01	9.76E-03
NOP16	7.36E-01	9.76E-03
TRK.TTT9.1	7.36E-01	9.76E-03
ZNF292	7.36E-01	9.76E-03
CALHM5	7.36E-01	9.76E-03
LOC105377977	7.36E-01	9.76E-03
ADAT2	7.36E-01	9.76E-03
STMP1	7.36E-01	9.76E-03
LOC101928095	7.36E-01	9.76E-03
LOC105379303	7.36E-01	9.76E-03
LOC107986886	7.36E-01	9.76E-03
ZFAND1	7.36E-01	9.76E-03
LOC105375733	7.36E-01	9.76E-03
LINC01388	7.36E-01	9.76E-03
LOC107984279	7.36E-01	9.76E-03
LOC105378567	7.36E-01	9.76E-03
OR4A47	7.36E-01	9.76E-03
LOC105369309	7.36E-01	9.76E-03
PRPF19	7.36E-01	9.76E-03
LOC101927583	7.36E-01	9.76E-03
LOC107984438	7.36E-01	9.76E-03
TRAV31	7.36E-01	9.76E-03
LOC105370706	7.36E-01	9.76E-03
SNURF	7.36E-01	9.76E-03
LINC02205	7.36E-01	9.76E-03
LOC101927227	7.36E-01	9.76E-03
LOC105371344	7.36E-01	9.76E-03
RPL27	7.36E-01	9.76E-03
LOC105371840	7.36E-01	9.76E-03
ZNF726	7.36E-01	9.76E-03
LOC105372694	7.36E-01	9.76E-03
ADRM1	7.36E-01	9.76E-03
LOC107987324	7.36E-01	9.76E-03
LOC101929372	7.36E-01	9.76E-03
FAM239A	7.36E-01	9.76E-03
TCEANC	7.36E-01	9.76E-03
FAM47A	7.36E-01	9.76E-03
LOC105373178	7.36E-01	9.76E-03
FLICR	7.36E-01	9.76E-03
SPANXN5	7.36E-01	9.76E-03

HPRT1	7.36E-01	9.76E-03
LOC105373373	7.36E-01	9.76E-03
REG4	7.27E-01	1.12E-02
LINC02591	7.27E-01	1.12E-02
H2AC19	7.27E-01	1.12E-02
KPRP	7.27E-01	1.12E-02
FAM78B	7.27E-01	1.12E-02
LOC105373117	7.27E-01	1.12E-02
LOC105374842	7.27E-01	1.12E-02
ECRG4	7.27E-01	1.12E-02
LOC105373539	7.27E-01	1.12E-02
LOC105373876	7.27E-01	1.12E-02
LOC729968	7.27E-01	1.12E-02
SRGAP3.AS3	7.27E-01	1.12E-02
GOLGA4	7.27E-01	1.12E-02
TWF2	7.27E-01	1.12E-02
KBTBD12	7.27E-01	1.12E-02
LOC105377275	7.27E-01	1.12E-02
PCAT4	7.27E-01	1.12E-02
MAD2L1	7.27E-01	1.12E-02
LOC105377503	7.27E-01	1.12E-02
LINC02162	7.27E-01	1.12E-02
LOC101929200	7.27E-01	1.12E-02
LINC01957	7.27E-01	1.12E-02
PCDHGA4	7.27E-01	1.12E-02
TRX.CAT1.2	7.27E-01	1.12E-02
LOC107986543	7.27E-01	1.12E-02
WASF1	7.27E-01	1.12E-02
INHBA	7.27E-01	1.12E-02
PCLO	7.27E-01	1.12E-02
SRI	7.27E-01	1.12E-02
LINC02847	7.27E-01	1.12E-02
LOC105375723	7.27E-01	1.12E-02
CCAT1	7.27E-01	1.12E-02
LOC107987065	7.27E-01	1.12E-02
FKBP15	7.27E-01	1.12E-02
POU5F1P5	7.27E-01	1.12E-02
LOC107984182	7.27E-01	1.12E-02
EDRF1.AS1	7.27E-01	1.12E-02
ELF5	7.27E-01	1.12E-02
RBM4	7.27E-01	1.12E-02

LOC107984536	7.27E-01	1.12E-02
MIR1827	7.27E-01	1.12E-02
ALDH1L2	7.27E-01	1.12E-02
LOC107984440	7.27E-01	1.12E-02
LOC105370050	7.27E-01	1.12E-02
LOC254028	7.27E-01	1.12E-02
LOC112268129	7.27E-01	1.12E-02
LOC105370687	7.27E-01	1.12E-02
PPP1R14D	7.27E-01	1.12E-02
FEM1B	7.27E-01	1.12E-02
EFL1	7.27E-01	1.12E-02
SLC5A10	7.27E-01	1.12E-02
LOC105371789	7.27E-01	1.12E-02
HOXB5	7.27E-01	1.12E-02
LINC01909	7.27E-01	1.12E-02
EVI5L	7.27E-01	1.12E-02
NCCRP1	7.27E-01	1.12E-02
MIR644A	7.27E-01	1.12E-02
TRAPPC2	7.27E-01	1.12E-02
CLCN5	7.27E-01	1.12E-02
LOC105373205	7.27E-01	1.12E-02
SLC16A2	7.27E-01	1.12E-02
MIR548AN	7.27E-01	1.12E-02
LOC105371750_1	7.27E-01	1.12E-02
AUNIP	7.18E-01	1.28E-02
LOC107984962	7.18E-01	1.28E-02
LINC02791	7.18E-01	1.28E-02
H2BC21	7.18E-01	1.28E-02
CENPF	7.18E-01	1.28E-02
LOC107985359	7.18E-01	1.28E-02
TOMM20	7.18E-01	1.28E-02
LOC105373587	7.18E-01	1.28E-02
LOC105373812	7.18E-01	1.28E-02
SNRK.AS1	7.18E-01	1.28E-02
LINC02042	7.18E-01	1.28E-02
MIR9900	7.18E-01	1.28E-02
IGF2BP2.AS1	7.18E-01	1.28E-02
LOC105374359	7.18E-01	1.28E-02
FBXL5	7.18E-01	1.28E-02
PHOX2B.AS1	7.18E-01	1.28E-02
LOC105377255	7.18E-01	1.28E-02

F11.AS1	7.18E-01	1.28E-02
BRIX1	7.18E-01	1.28E-02
LOC105378965	7.18E-01	1.28E-02
LOC105374929	7.18E-01	1.28E-02
UBE2J1	7.18E-01	1.28E-02
BACH2	7.18E-01	1.28E-02
RBAK	7.18E-01	1.28E-02
LOC105375253	7.18E-01	1.28E-02
ARMC10	7.18E-01	1.28E-02
DPY19L4	7.18E-01	1.28E-02
LOC107986979	7.18E-01	1.28E-02
MIR101.2	7.18E-01	1.28E-02
LOC107987102	7.18E-01	1.28E-02
XPA	7.18E-01	1.28E-02
LOC107987117	7.18E-01	1.28E-02
GRIN1	7.18E-01	1.28E-02
LOC105376467	7.18E-01	1.28E-02
OR51B2	7.18E-01	1.28E-02
LOC105376655	7.18E-01	1.28E-02
EEF1G	7.18E-01	1.28E-02
RAB38	7.18E-01	1.28E-02
LINC02719	7.18E-01	1.28E-02
SLC35F2	7.18E-01	1.28E-02
LOC101928030	7.18E-01	1.28E-02
LOC105369668	7.18E-01	1.28E-02
MGP	7.18E-01	1.28E-02
APPL2	7.18E-01	1.28E-02
LOC107984577	7.18E-01	1.28E-02
LOC107984551	7.18E-01	1.28E-02
CPB2.AS1	7.18E-01	1.28E-02
TRAJ26	7.18E-01	1.28E-02
LOC105370440	7.18E-01	1.28E-02
TRMT5	7.18E-01	1.28E-02
FBLN5	7.18E-01	1.28E-02
TUNAR	7.18E-01	1.28E-02
ZNF75A	7.18E-01	1.28E-02
LOC101928708	7.18E-01	1.28E-02
LOC105371520	7.18E-01	1.28E-02
LINC02093	7.18E-01	1.28E-02
LOC105371850	7.18E-01	1.28E-02
PIAS2	7.18E-01	1.28E-02

RPS15	7.18E-01	1.28E-02
SNORA68B	7.18E-01	1.28E-02
EMC10	7.18E-01	1.28E-02
LOC105372699	7.18E-01	1.28E-02
KRTAP19.2	7.18E-01	1.28E-02
LOC101927635	7.18E-01	1.28E-02
ZC3H12B	7.18E-01	1.28E-02
TNMD	7.18E-01	1.28E-02
KLHL13	7.18E-01	1.28E-02
DUSP5P1_1	7.18E-01	1.28E-02
LOC105371446	7.09E-01	1.46E-02
FDPS	7.09E-01	1.46E-02
KIRREL1	7.09E-01	1.46E-02
C4BPB	7.09E-01	1.46E-02
LINC02767	7.09E-01	1.46E-02
CNIH3	7.09E-01	1.46E-02
LOC107985466	7.09E-01	1.46E-02
LOC105374589	7.09E-01	1.46E-02
FAM138B	7.09E-01	1.46E-02
LSM3	7.09E-01	1.46E-02
LOC102724104	7.09E-01	1.46E-02
SPINK8	7.09E-01	1.46E-02
LSMEM2	7.09E-01	1.46E-02
LOC105377114	7.09E-01	1.46E-02
PRICKLE2.AS3	7.09E-01	1.46E-02
LINC00635	7.09E-01	1.46E-02
BCHE	7.09E-01	1.46E-02
LINC02013	7.09E-01	1.46E-02
LOC107986253	7.09E-01	1.46E-02
LINC00504	7.09E-01	1.46E-02
MIR573	7.09E-01	1.46E-02
LOC102724210	7.09E-01	1.46E-02
SNORA105A	7.09E-01	1.46E-02
LOC105374715	7.09E-01	1.46E-02
LOC105379052	7.09E-01	1.46E-02
LOC101929710	7.09E-01	1.46E-02
TAF7	7.09E-01	1.46E-02
PCDHGB7	7.09E-01	1.46E-02
LOC107983980	7.09E-01	1.46E-02
LINC02159	7.09E-01	1.46E-02
ZFP2	7.09E-01	1.46E-02

LINC00847	7.09E-01	1.46E-02
DOP1A	7.09E-01	1.46E-02
LOC105378023	7.09E-01	1.46E-02
LOC107986663	7.09E-01	1.46E-02
CREB5	7.09E-01	1.46E-02
LOC107986789	7.09E-01	1.46E-02
LSMEM1	7.09E-01	1.46E-02
LOC101928782	7.09E-01	1.46E-02
LOC102723322	7.09E-01	1.46E-02
ELAVL2	7.09E-01	1.46E-02
PRKACG	7.09E-01	1.46E-02
ZNF782	7.09E-01	1.46E-02
KLF4	7.09E-01	1.46E-02
LOC107987118	7.09E-01	1.46E-02
LOC105378475	7.09E-01	1.46E-02
OR5J2	7.09E-01	1.46E-02
LOC101928940	7.09E-01	1.46E-02
LOC101929432	7.09E-01	1.46E-02
HOXC11	7.09E-01	1.46E-02
EIF2B1	7.09E-01	1.46E-02
LINC00462	7.09E-01	1.46E-02
LOC105370481	7.09E-01	1.46E-02
LOC105370842	7.09E-01	1.46E-02
LOC106660606	7.09E-01	1.46E-02
NPIPB2	7.09E-01	1.46E-02
OR1E3	7.09E-01	1.46E-02
LINC02096	7.09E-01	1.46E-02
CCL2	7.09E-01	1.46E-02
PRKCA.AS1	7.09E-01	1.46E-02
LOC112268199	7.09E-01	1.46E-02
NPTX1	7.09E-01	1.46E-02
LOC105379280	7.09E-01	1.46E-02
LINC02837	7.09E-01	1.46E-02
HSBP1L1	7.09E-01	1.46E-02
LOC105372242	7.09E-01	1.46E-02
LOC107987269	7.09E-01	1.46E-02
KCNJ6.AS1	7.09E-01	1.46E-02
RTN4R	7.09E-01	1.46E-02
APOL1	7.09E-01	1.46E-02
LOC101927393	7.09E-01	1.46E-02
PGAM4	7.09E-01	1.46E-02

LOC105373333	7.09E-01	1.46E-02
CPTP	7.00E-01	1.65E-02
TPRG1L	7.00E-01	1.65E-02
LOC105376717	7.00E-01	1.65E-02
MIR3659HG	7.00E-01	1.65E-02
PPIEL	7.00E-01	1.65E-02
LOC105378771	7.00E-01	1.65E-02
DIRAS3	7.00E-01	1.65E-02
LINC01709	7.00E-01	1.65E-02
C1orf56	7.00E-01	1.65E-02
FLG.AS1	7.00E-01	1.65E-02
GPA33	7.00E-01	1.65E-02
SLC19A2	7.00E-01	1.65E-02
LOC105373159	7.00E-01	1.65E-02
LOC105373275	7.00E-01	1.65E-02
MYT1L.AS1	7.00E-01	1.65E-02
SUPT7L	7.00E-01	1.65E-02
FUNDC2P2	7.00E-01	1.65E-02
ANKRD36BP2	7.00E-01	1.65E-02
LOC105373501	7.00E-01	1.65E-02
PSMD14	7.00E-01	1.65E-02
GMPPB	7.00E-01	1.65E-02
LINC02050	7.00E-01	1.65E-02
LOC105374260	7.00E-01	1.65E-02
YIPF7	7.00E-01	1.65E-02
LOC105377661	7.00E-01	1.65E-02
CSN3	7.00E-01	1.65E-02
CABS1	7.00E-01	1.65E-02
CXCL2	7.00E-01	1.65E-02
SNORD144	7.00E-01	1.65E-02
MCUB	7.00E-01	1.65E-02
LOC105377462	7.00E-01	1.65E-02
LOC105377568	7.00E-01	1.65E-02
LOC105377607	7.00E-01	1.65E-02
LOC105374659	7.00E-01	1.65E-02
LOC107986423	7.00E-01	1.65E-02
LOC107986438	7.00E-01	1.65E-02
FAM53C	7.00E-01	1.65E-02
PCDHGA9	7.00E-01	1.65E-02
TRIM52	7.00E-01	1.65E-02
H4C4	7.00E-01	1.65E-02

MIR548B	7.00E-01	1.65E-02
PKIB	7.00E-01	1.65E-02
MAP3K5.AS1	7.00E-01	1.65E-02
LOC102723758	7.00E-01	1.65E-02
WIPI2	7.00E-01	1.65E-02
PRPS1L1	7.00E-01	1.65E-02
LAT2	7.00E-01	1.65E-02
PRKAR2B.AS1	7.00E-01	1.65E-02
IDO1	7.00E-01	1.65E-02
RNF170	7.00E-01	1.65E-02
PABPC1	7.00E-01	1.65E-02
LOC105375759	7.00E-01	1.65E-02
LOC105379447	7.00E-01	1.65E-02
TNFSF8	7.00E-01	1.65E-02
LOC101930421	7.00E-01	1.65E-02
MIR6078	7.00E-01	1.65E-02
LINC01517	7.00E-01	1.65E-02
LINC01375	7.00E-01	1.65E-02
HABP2	7.00E-01	1.65E-02
TRIM34	7.00E-01	1.65E-02
LOC105376571	7.00E-01	1.65E-02
COX8A	7.00E-01	1.65E-02
LOC105369526	7.00E-01	1.65E-02
HYLS1	7.00E-01	1.65E-02
SOX5.AS1	7.00E-01	1.65E-02
TEX49	7.00E-01	1.65E-02
NTS	7.00E-01	1.65E-02
LOC102723381	7.00E-01	1.65E-02
LOC107984601	7.00E-01	1.65E-02
OR5AU1	7.00E-01	1.65E-02
TRAV24	7.00E-01	1.65E-02
SNORD56B	7.00E-01	1.65E-02
ACSM5	7.00E-01	1.65E-02
SMG1P2	7.00E-01	1.65E-02
LOC105371351	7.00E-01	1.65E-02
LOC100288728	7.00E-01	1.65E-02
XAF1	7.00E-01	1.65E-02
TBX21	7.00E-01	1.65E-02
OTOP2	7.00E-01	1.65E-02
CENPX	7.00E-01	1.65E-02
KLHL14	7.00E-01	1.65E-02

LOC100505549	7.00E-01	1.65E-02
LOC107985179	7.00E-01	1.65E-02
LOC100996288	7.00E-01	1.65E-02
PSG5	7.00E-01	1.65E-02
MIR518E	7.00E-01	1.65E-02
MIR548AG2	7.00E-01	1.65E-02
LINC01718	7.00E-01	1.65E-02
BCL2L13	7.00E-01	1.65E-02
LOC105373016	7.00E-01	1.65E-02
SREBF2.AS1	7.00E-01	1.65E-02
ATP6AP2	7.00E-01	1.65E-02
LOC107985665	7.00E-01	1.65E-02
SATL1	7.00E-01	1.65E-02
ALG13	7.00E-01	1.65E-02
RNF220	-7.00E-01	1.65E-02
LOC105373945	-7.00E-01	1.65E-02
DKFZp434L192	-7.00E-01	1.65E-02
LOC105376271	-7.00E-01	1.65E-02
LINC01872	-7.00E-01	1.65E-02
LOC107985279	-7.00E-01	1.65E-02
XIST	-7.00E-01	1.65E-02
RNA45SN5	-7.00E-01	1.65E-02
LOC112268313	-7.00E-01	1.65E-02
INAVA	-7.09E-01	1.46E-02
KIF5C.AS1	-7.09E-01	1.46E-02
TRV.AAC1.3	-7.09E-01	1.46E-02
TSPYL4	-7.09E-01	1.46E-02
DKK1	-7.09E-01	1.46E-02
C11orf86	-7.09E-01	1.46E-02
ZNF335	-7.09E-01	1.46E-02
SSX2	-7.09E-01	1.46E-02
LOC107986830	-7.18E-01	1.28E-02
LOC105375805	-7.18E-01	1.28E-02
KIF20B	-7.18E-01	1.28E-02
LOC105378569	-7.18E-01	1.28E-02
MARVELD2_1	-7.18E-01	1.28E-02
GJA9.MYCBP	-7.27E-01	1.12E-02
FLVCR1	-7.27E-01	1.12E-02
HSPC324	-7.27E-01	1.12E-02
LOXL4	-7.27E-01	1.12E-02
DNMBP	-7.27E-01	1.12E-02

LOC102723392	-7.27E-01	1.12E-02
SLIRP	-7.27E-01	1.12E-02
MIR10A	-7.27E-01	1.12E-02
TMED1	-7.27E-01	1.12E-02
LOC100128593	-7.36E-01	9.76E-03
PRPF31	-7.36E-01	9.76E-03
PISRT1	-7.45E-01	8.45E-03
GFUS	-7.45E-01	8.45E-03
HSD17B3.AS1	-7.45E-01	8.45E-03
SLC46A3	-7.45E-01	8.45E-03
MYBL2	-7.45E-01	8.45E-03
MOSMO_1	-7.45E-01	8.45E-03
TEX10	-7.55E-01	7.28E-03
TRAFD1	-7.55E-01	7.28E-03
KLK11	-7.55E-01	7.28E-03
RPS9	-7.55E-01	7.28E-03
LRP5L	-7.55E-01	7.28E-03
DHX36_1	-7.55E-01	7.28E-03
CRP	-7.64E-01	6.23E-03
PPP1R14B.AS1	-7.64E-01	6.23E-03
PPP1R9B	-7.64E-01	6.23E-03
MIR5008	-7.73E-01	5.30E-03
LOC105373582	-7.73E-01	5.30E-03
CXXC5	-7.73E-01	5.30E-03
LINC00965	-7.73E-01	5.30E-03
POLR2A	-7.73E-01	5.30E-03
LOC105371781	-7.73E-01	5.30E-03
LOXHD1	-7.73E-01	5.30E-03
LOC105372727	-7.73E-01	5.30E-03
LOC107987289	-7.73E-01	5.30E-03
LOC105378536_1	-7.73E-01	5.30E-03
POLR2A_1	-7.73E-01	5.30E-03
KRTAP5.6_1	-7.73E-01	5.30E-03
MPIG6B_5	-7.73E-01	5.30E-03
CYP21A2_4	-7.73E-01	5.30E-03
KIFC1_1	-7.73E-01	5.30E-03
KIR3DX1_2	-7.73E-01	5.30E-03
GOLGA6L3_1	-7.82E-01	4.47E-03
DLX4	-7.91E-01	3.75E-03
ITGA3	-7.91E-01	3.75E-03
LOC105372268	-7.91E-01	3.75E-03

CEBPB	-7.91E-01	3.75E-03
PIP	-8.00E-01	3.11E-03
LOC105379533	-8.00E-01	3.11E-03
POLR3E_1	-8.00E-01	3.11E-03
LOC102723750	-8.09E-01	2.56E-03
ZNF213	-8.18E-01	2.08E-03
TTTY7B	-8.18E-01	2.08E-03
LOC105372451	-8.27E-01	1.68E-03
DCD	-8.55E-01	8.07E-04
ENDOU	-8.64E-01	6.12E-04
