# Supplementary material for: RNA Sequencing of Intestinal Enterocytes Pre- and Post-Roux-en-Y Gastric Bypass Reveals Alteration in Gene Expression Related to Enterocyte Differentiation, Restitution, and Obesity with Regulation by Schlafen 12
Source: Cells. 2022 Oct 18;11(20):3283. doi: 10.3390/cells11203283 (PMC9601224; doi:10.3390/cells11203283)
Supplement: Supplementary file 1 [file cells-11-03283-s001.zip › Table S5. BMIPercent_Change_FC_19gene_linerRes.pdf]

		Linear Relation with change of BMI				Correlation with Change of BMI	
Gene Name	Detail	Estimate	Std.Error	t.value	Pvalue	Correlation Value	Correlation Pval
C11orf86	chromosome 11 open reading frame 86	-2.63E+01	7.33E+00	-3.59E+00	5.82E-03	-7.09E-01	1.46E-02
LINC01061	long intergenic non-protein coding RNA 1061	2.13E-01	6.44E-02	3.30E+00	9.21E-03	8.00E-01	3.11E-03
LOC101928254	uncharacterized LOC101928254	1.40E-01	3.52E-02	3.97E+00	3.27E-03	8.64E-01	6.12E-04
LOC105371840	uncharacterized LOC105371840	1.91E-01	5.70E-02	3.36E+00	8.45E-03	7.36E-01	9.76E-03
LOC105373945	uncharacterized LOC105373945	-1.33E-01	3.91E-02	-3.40E+00	7.87E-03	-7.00E-01	1.65E-02
LOC105375952	uncharacterized LOC105375952	6.63E-02	1.83E-02	3.62E+00	5.61E-03	8.18E-01	2.08E-03
LOC105377607	uncharacterized LOC105377607	1.56E-01	4.50E-02	3.48E+00	6.98E-03	7.00E-01	1.65E-02
LOC105379533	translation initiation factor IF-2-like	-4.63E-02	1.35E-02	-3.43E+00	7.52E-03	-8.00E-01	3.11E-03
LOC107984334	uncharacterized LOC107984334	2.07E+00	5.78E-01	3.59E+00	5.86E-03	7.82E-01	4.47E-03
LOC107986830	uncharacterized LOC107986830	-6.54E+01	1.86E+01	-3.52E+00	6.54E-03	-7.18E-01	1.28E-02
LOC112268129	uncharacterized LOC112268129	1.04E+01	2.97E+00	3.50E+00	6.76E-03	7.27E-01	1.12E-02
LOC112268313	collagen alpha-1(I) chain-like	-1.94E-01	5.30E-02	-3.66E+00	5.21E-03	-7.00E-01	1.65E-02
PISRT1	PISRT1 lncRNA	-1.08E+02	2.33E+01	-4.63E+00	1.23E-03	-7.45E-01	8.45E-03
PPP1R14B-AS1	PPP1R14B antisense RNA 1	-1.23E+01	3.49E+00	-3.53E+00	6.41E-03	-7.64E-01	6.23E-03
QPCT	glutaminyl-peptide cyclotransferase	1.37E-01	3.47E-02	3.94E+00	3.39E-03	7.55E-01	7.28E-03
RNA45SN5	RNA, 45S pre-ribosomal N5	-1.59E-01	4.42E-02	-3.61E+00	5.67E-03	-7.00E-01	1.65E-02
RNF170	ring finger protein 170	8.80E-02	2.52E-02	3.50E+00	6.75E-03	7.00E-01	1.65E-02
RPS9	ribosomal protein S9	-8.07E+00	2.43E+00	-3.32E+00	9.00E-03	-7.55E-01	7.28E-03
ZNF213	zinc finger protein 213	-1.06E-01	2.30E-02	-4.61E+00	1.27E-03	-8.18E-01	2.08E-03
